# All-metal aromaticity of cyclo-Bi_3_^3−^ in diuranium and dithorium inverse-sandwich-type complexes

**DOI:** 10.1038/s41557-026-02123-8

**Published:** 2026-04-20

**Authors:** Junru Ding, John A. Seed, Katrin Beuthert, Benjamin Peerless, Julia Rienmüller, Andreas Schmidt, Ashley J. Wooles, Louise S. Natrajan, Chuan-Ling Chen, Zhong-Ming Sun, Florian Weigend, Stefanie Dehnen, Jingzhen Du, Stephen T. Liddle

**Affiliations:** 1 College of Chemistry, Zhengzhou University, Zhengzhou, China; 2 Department of Chemistry and Centre for Radiochemistry Research, University of Manchester, Manchester, UK; 3 Institute of Nanotechnology, Karlsruhe Institute of Technology, Eggenstein-Leopoldshafen, Germany; 4 State Key Laboratory of Elemento-Organic Chemistry, Tianjin Key Lab for Rare Earth Materials and Applications, School of Materials Science and Engineering, Nankai University, Tianjin, China; 5 Department of Chemistry, Fudan University, Shanghai, China; 6 Institute for Quantum Materials and Technologies, Karlsruhe Institute of Technology, Eggenstein-Leopoldshafen, Germany

**Keywords:** Inorganic chemistry, Coordination chemistry

## Abstract

Delocalized [4*n* + 2]π-aromaticity in cyclic planar unsaturated organic molecules conceptually underpins organic chemistry. Recently, the study of all-metal aromaticity has burgeoned, but although there has been interest in cyclo-{E_3_} (E = P, As, Sb, Bi) species as cyclopropenium analogues, the formation of cyclo-{Bi_3_} remains rare. Thus, the potential aromaticity of 2/6π-cyclo-Bi_3_^+/3−^, as the heaviest 6*p* analogue of cyclopropylium, has remained open to different interpretations. Here we report the formation of diuranium and dithorium 6π-cyclo-Bi_3_^3−^ inverse sandwich complexes, complementing the small number of acyclic- and cyclic-Bi_*n*_ (*n* = 3–5) compounds. The 6π-cyclo-Bi_3_^3−^ ring exhibits substantial ring currents, similar to 6π-benzene, 2π-(C_3_H_3_)^+^ or 6π-(C_3_H_3_)^3−^. Calculations reveal similar ring currents for 6π-cyclo-Bi_3_^3−^, 2π-cyclo-Bi_3_^+^ and 0π-cyclo-Bi_3_^3+^, demonstrating σ-aromaticity that is dominant over π-aromaticity in cyclo-Bi_3_^3−^, despite the favourability of describing cyclo-Bi_3_^3−^ with localized rather than delocalized bond descriptions. Confirmation of 6π-cyclo-Bi_3_^3−^ σ-aromaticity provides the heaviest all-metal 6*p* analogue to π-aromatic (C_3_H_3_)^+/3−^, leading to organic–inorganic aromaticity benchmarking.

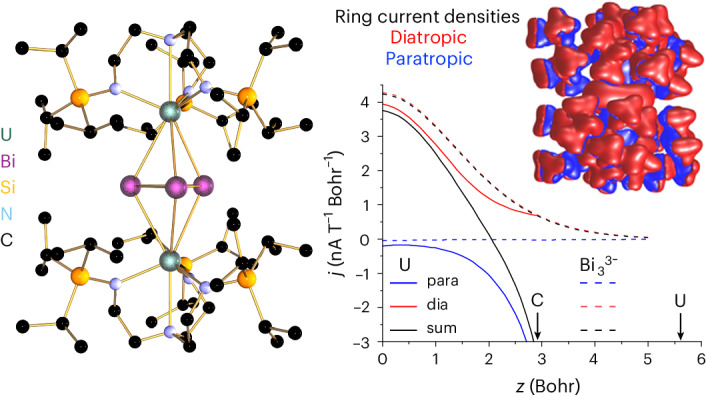

## Abstract (web summary)

Classical understanding of aromaticity has been challenged by its emergence in rings featuring only metal atoms. Now, diuranium and dithorium inverse sandwich complexes featuring a central cyclo-Bi_3_^3−^ unit have been synthesized. These tribismuth rings exhibit exalted diamagnetism and are σ-aromatic, in contrast to the π-aromaticity in analogous tricarbon rings, providing benchmark data for comparisons between organic and inorganic aromaticity.

## Main

**Fig. 1:**
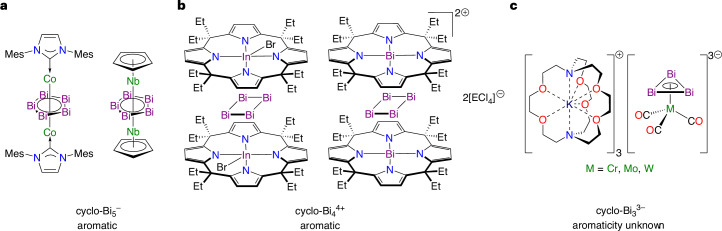
Examples of aromatic and potentially aromatic cyclic Bi_*n*_ (*n* = 3–5) rings. **a**–**c**, Cyclo-Bi_5_^−^ (**a**), cyclo-Bi_4_^4+^ (**b**) and cyclo-Bi_3_^3−^ (**c**).

Delocalized Hückel [4*n* + 2]π-aromaticity in classical cyclic planar unsaturated organic molecules^1,2^ such as benzene (C_6_H_6_) or the cyclopropenium cation ((C_3_H_3_)^+^), which occur terrestrially and in interstellar space^3,4^, conceptually underpins organic chemistry. Recently, the study of molecules exhibiting all-metal aromatic bonding^5–8^ has become a burgeoning, yet intensively debated field^9–52^, because whether it is appropriate to apply Hückel’s model to systems beyond second-row elements remains an open question. Indeed, in the absence of consensus on the definition of aromaticity^53^, discussions can only be valid within their own arbitrary criteria. Accordingly, the study of potentially aromatic all-metal species is necessary to develop further insight, and, ultimately a consensus. Examples now span inorganic heterocycles with three-, four- and five-membered rings with a range of metals from lithium to thorium providing complementary or contrasting types of inorganic aromaticity to established organic aromaticity^1–52^. However, although there has been interest in cyclo-{E_3_} (E = P, As, Sb, Bi) species as heavy potentially aromatic 2/6π-(C_3_H_3_)^+/3−^ analogues^54–61^, the formation of discrete Bi_3_ units in any charge state is rare^62–67^ due to the tendency of bismuth to form large catenated clusters^68–71^. Discrete cyclic poly-bismuth species are comparatively unusual, with examples including cyclo-Bi_5_^−^ in [{(IMes)Co}_2_(μ,η^5^:η^5^-cyclo-Bi_5_)] (IMes = C(NMesCH)_2_; Mes = 2,4,6-trimethylphenyl) and [{(η^5^-C_5_H_5_)Nb}(μ,η^5^:η^5^-cyclo-Bi_5_)]^50,51^, cyclo*-*Bi_4_^4+^ in [cyclo-Bi_4_][InBr(OEP)]_2_ (OEP = octaethylporphyrinogen) and [cyclo-Bi_4_][Bi(OEP)]_2_[ECl_4_]_2_ (E = Al, Ga)^49^ and cyclo-Bi_3_^3−^ in [K(2.2.2-cryptand)]_3_[(η^3^-cyclo-Bi_3_)M(CO)_3_] (M = Cr, Mo, W) (Fig. 1)^65,66^. Notably, the electronic situation with respect to the aromaticity of cyclo*-*Bi_3_ in [(η^3^-cyclo-Bi_3_)M(CO)_3_]^3−^ (M = Cr, Mo, W) has not been clarified, so is open to different interpretations. Therefore, the potential aromaticity of 2/6π-cyclo-Bi_3_^+/3−^, as the heaviest 6*p* analogue of (C_3_H_3_)^+/3−^, has remained an open question.

In this Article, we report the formation of 6π-cyclo-Bi_3_^3−^ in diuranium and dithorium complexes, both exhibiting bridging cyclo-Bi_3_^3−^. The 6π-cyclo-Bi_3_^3−^ ring is perfectly (or highly) symmetric, and as an isolated species exhibits a computed ring current of 16.5 nA T^−1^, which is greater than that of cyclo*-*Bi_4_^4+^ or cyclo-Bi_5_^−^ inorganic congeners, and also greater than 6π-benzene, 2π-(C_3_H_3_)^+^ or 6π-(C_3_H_3_)^3−^ organic reference molecules. Even when proximate to actinide ions, the computed ring currents from the 6π-cyclo-Bi_3_^3−^, which are ±2 Bohr from the cyclo-Bi_3_^3−^ centroid, remain substantial (7.1–9.9 nA T^−1^). Intriguingly, it is found that σ-aromaticity is dominant over π-aromaticity for cyclo-Bi_3_^3−^. Demonstrating 6π-cyclo-Bi_3_^3−^ aromaticity conceptually provides the heaviest all-metal aromatic 6*p* analogue—and contrast—to (C_3_H_3_)^+/3−^, and provides a cyclic complement to an acyclic tribismuth allyl-cation^72^, enabling the development of organic–inorganic aromaticity benchmarking.

## Results and discussion

### Synthesis of co-crystallized diuranium cyclo-Bi_3_^3−^ and Bi^3−^ complexes

**Fig. 2:**
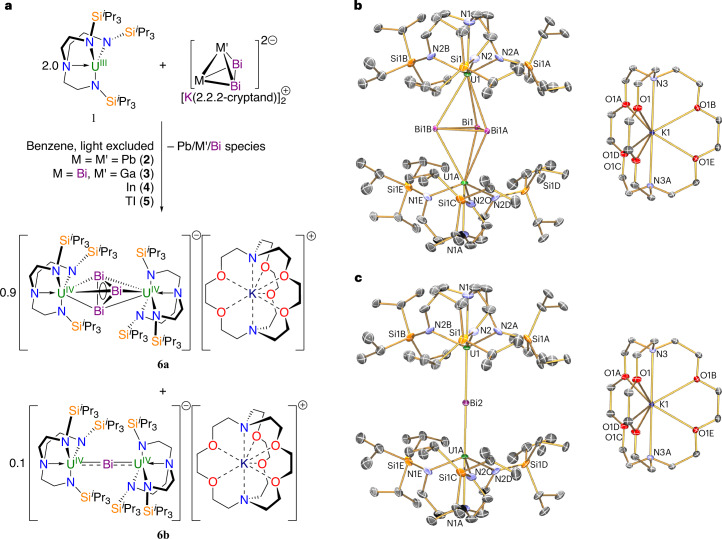
Synthesis and structures of 6a and 6b. **a**, Synthesis of the diuranium cyclo-Bi_3_^3−^ complex **6a**, along with inseparable co-crystallized sub-component **6b**, from the reaction of 2 equiv. of **1** with either **2**, **3**, **4** or **5**. **b**, Molecular structure of **6a** at 150 K with 20% probability ellipsoids and hydrogen atoms omitted for clarity. **c**, Molecular structure of **6b** at 150 K with 20% probability ellipsoids and hydrogen atoms omitted for clarity.

We previously reported that treatment of [U^III^(Tren^TIPS^)] (**1**, Tren^TIPS^ = {N(CH_2_CH_2_NSi^*i*^Pr_3_)_3_}^3−^)^73^ with Me_2_N-PDBN (PDBN = 7*λ*^3^-phosphadibenzonorbornadiene)^74,75^ affords the diphosphorus complex [{U^IV^(Tren^TIPS^)}_2_(μ-η^2^:η^2^-P_2_)]^70^, which when reduced can be converted to diuranium(IV)-P_2_^3−^ or -cyclo*-*P_3_^3−^ derivatives^70,76^. Noting that lanthanide and transition-metal {Bi_*n*_} (*n* = 2, 3) complexes have been prepared from low-valent metal precursors with different bismuth compounds via redox reactions^62,63,66,67,69,71,77–80^, and that clusters with [U@Pb_4_Bi_9_]^3−^, [U@Pb_7_Bi_7_]^3−^, [U@Tl_2_Bi_11_]^3−^ and [U@Bi_12_]^3−^ have been constructed from [K(2.2.2-cryptand)]_2_[MM′Bi_2_] en (M = M′ = Pb (**2**) or M′ = Bi, M = Ga (**3**), In (**4**) and Tl (**5**); en = ethane-1,2-diamine)^62,77^ reacting with uranium–tetramethylcyclopentadienyl complexes^64^, targeting uranium-cyclo-Bi_3_ functionalities, we examined the reactivity of **1** with compounds **2**–**5**. Complex **1** typically undergoes one-electron oxidations^81,82^, so we determined that the optimal ratio for **1** to react with **2**, **3**, **4** or **5** is 2:1. The reactions produced red slurries, from which dark red–black crystalline material was isolated in low but reproducible yields of ~24% (by uranium; Fig. 2a). This product was consistently isolated as the sole product, in contrast to the aforementioned uranium cyclopentadienyl reactions, which gave different products for each of **2**, **3**, **4** and **5**. The dark red–black crystalline material is light-sensitive, and decomposes in tetrahydrofuran (THF) solvent to give an intractable black precipitate and green solutions characteristic of polybismuthides. Although the U–Bi bond in [U^IV^(Tren^DMBS^){Bi(SiMe_3_)_2_}] (Tren^DMBS^ = {N(CH_2_CH_2_NSiMe_2_^*t*^Bu)_3_}^3−^)^83^ could be prepared by salt elimination, reactions of [U^IV^(Tren^TIPS^)(THF)][BPh_4_] with **2**, **3**, **4** or **5** produced **1** rather than effecting salt elimination, underscoring the facile redox chemistry of **2**–**5**. Using the pseudo-element concept^77^, **2**–**5** are best regarded as formally containing (Pb^−^)_2_ or (M′)^2−^ with (Bi^0^)_*n*_ units, where the anionic components are strongly reducing.

### Solid-state structures of co-crystallized diuranium cyclo-Bi_3_^3−^ and Bi^3−^ complexes

The solid-state structure of the dark red–black crystalline material was determined by single-crystal X-ray diffraction to be 89% [K(2.2.2-cryptand)][{U^IV^(Tren^TIPS^)}_2_(μ-η^3^:η^3^-Bi_3_)] (**6a**) (Fig. 2b) with 11% co-crystallized [K(2.2.2-cryptand)][{U^IV^(Tren^TIPS^)}_2_(μ-Bi)] (**6b**) (Fig. 2c). The {U(Tren^TIPS^)}^+^ units in **6a** and **6b** reside on common sites, so only the disordered encapsulated bismuth units vary and the solubilities of **6a** and **6b** are essentially identical, thus preventing separation. The cyclo-Bi_3_^3−^ unit resides around a crystallographic three-fold rotation axis, so is an equilateral triangle with Bi–Bi distances of 2.9534(6) Å, which is between twice the covalent single (3.02 Å) and double bond (2.82 Å) radii of Bi^84^, longer than the Bi=Bi double bonds in [{Ln^III^(C_5_Me_5_)_2_}_2_(μ-η^2^:η^2^-Bi_2_)] (Bi–Bi = av. ~2.85 Å; Ln = Sm, Gd, Tb, Dy, Y)^85,86^, but similar to the Bi–Bi distances of 2.9560(5)–2.9867(3) Å in [K(2.2.2-cryptand)]_3_[(η^3^-cyclo-Bi_3_)M^0^(CO)_3_] (M = Cr, Mo, W)^65,66^. In **6a** the U–Bi distances are 3.4050(8) Å, longer than the single bond covalent radii of U and Bi (3.21 Å)^84^ and the U–Bi distance of 3.3208(4) Å in [U^IV^(Tren^DMBS^){Bi(SiMe_3_)_2_}]^83^, but in the range of U–Bi distances in polybismuthide clusters (3.2104(11)–3.5908(9) Å)^68,69^, reflecting the cyclo-Bi_3_^3−^ inverse sandwich motif. The Bi–Bi and U–Bi bond metrics of **6a** are consistent with the bridging nature of a π-bonded cyclo-Bi_3_^3−^ unit and a delocalized, aromatic cyclo-Bi_3_^3−^ unit. In **6b** (Fig. 2c), the U–Bi distances of 2.9473(9) Å are between the sum of the double and single bond covalent radii of U and Bi (2.75 and 3.21 Å, respectively)^84^, and shorter than the U–Bi distance of 3.3208(4) Å in [U^IV^(Tren^DMBS^){Bi(SiMe_3_)_2_}]^83^, suggesting polar U–Bi multiple bonding analogous to phosphide and stibido congeners^87,88^. The U–N_amide_ and U–N_amine_ distances in **6a**/**6b** are unremarkable^81^.

### Characterization of co-crystallized diuranium cyclo-Bi_3_^3−^ and Bi^3−^ complexes

The 89:11 **6a**:**6b** ratio (hereafter referred to as **6**) is supported by elemental analysis data. Only one set of Tren^TIPS^ resonances are observed in the ^1^H and ^29^Si NMR spectra of **6** (Supplementary Figs. 41–43), reflecting their chemical equivalence, irrespective of the bridging Bi unit. The ^1^H NMR spectrum of **6** spans a narrow range (5 to −3 ppm), and the ^29^Si{^1^H} NMR spectrum exhibits a singlet at −7.73 ppm; both features are consistent with a uranium(IV) formulation^89^. The light sensitivity of **6** precluded the acquisition of definitive Raman data, and the infrared spectrum measurement window cuts off where any Bi–Bi vibrations would be expected to occur (~70–150 cm^−1^).

**Extended Data Fig. 1:**
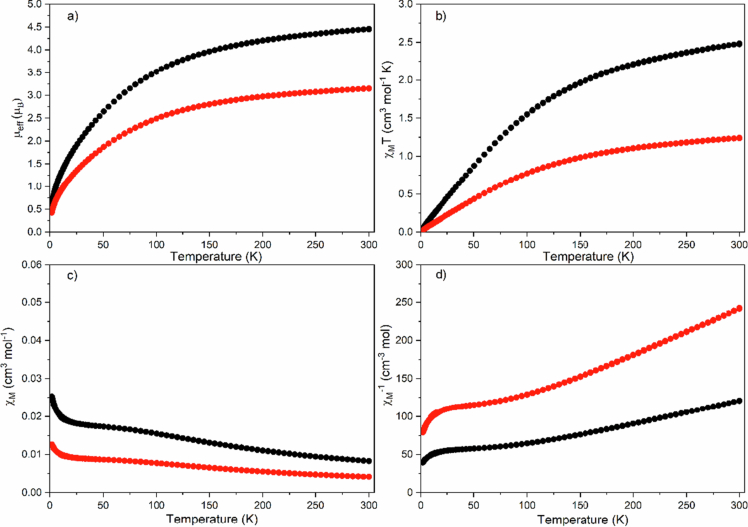
Variable-temperature SQUID magnetometry for 9:1 6a:6b. **a**) μ_eff_ vs T. **b**) χ_M_T vs T. **c**) χ_M_ vs T. **d**) χ_M_^−1^ vs T. Data were recorded over the temperature range 300-1.8 K in an external 0.5 T field. Colour key: black, per molecule; red, per uranium. The lines are a guide to the eye only.

Superconducting quantum interference device (SQUID) magnetometry on powdered **6** (Extended Data Fig. 1 and Supplementary Figs. 126 and 127) revealed an effective magnetic moment of 4.45 μ_B_ (2.48 cm^3^ mol^−1^ K) at 300 K (3.15 μ_B_/1.24 cm^3^ mol^−1^ K per uranium ion), which decreases smoothly over the temperature range, reaching 0.60 μ_B_ (0.05 cm^3^ mol^−1^ K) at 1.8 K (0.43 μ_B_/0.02 cm^3^ mol^−1^ K per uranium ion) and tending to zero. The magnetization versus field data for **6** do not saturate up to 7 T, so the SQUID data are characteristic of ^3^H_4_ uranium(IV) ions with magnetic singlet ground states and temperature-independent paramagnetism, but no uranium–uranium magnetic coupling^90–92^.

### Attempts to access a pure diuranium cyclo-Bi_3_^3−^ complex

**Extended Data Fig. 2:**
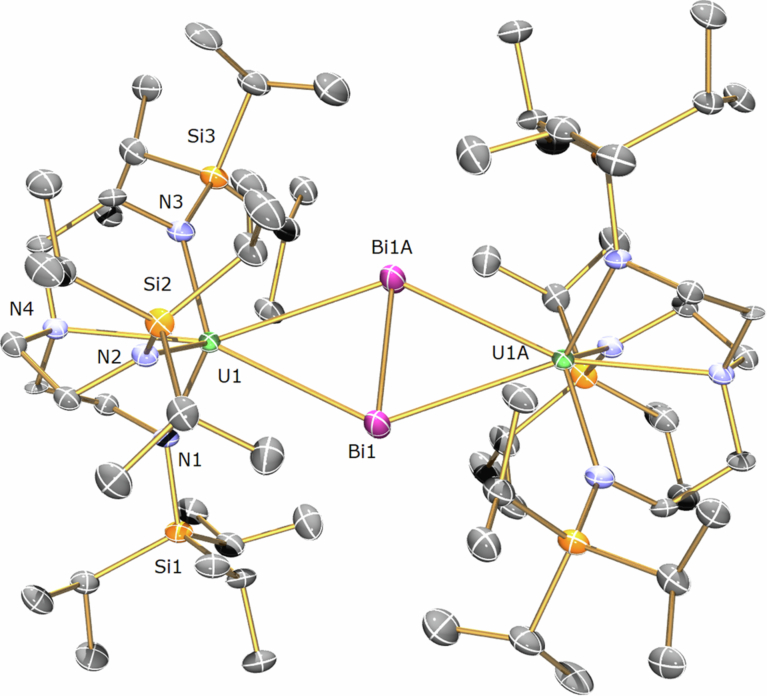
Single crystal X-ray diffraction solid state structure of 8. Data were collected at 100 K with displacement ellipsoids at 30% and selective labelling. Hydrogen atoms and disordered components are omitted for clarity.

**Extended Data Fig. 3:**
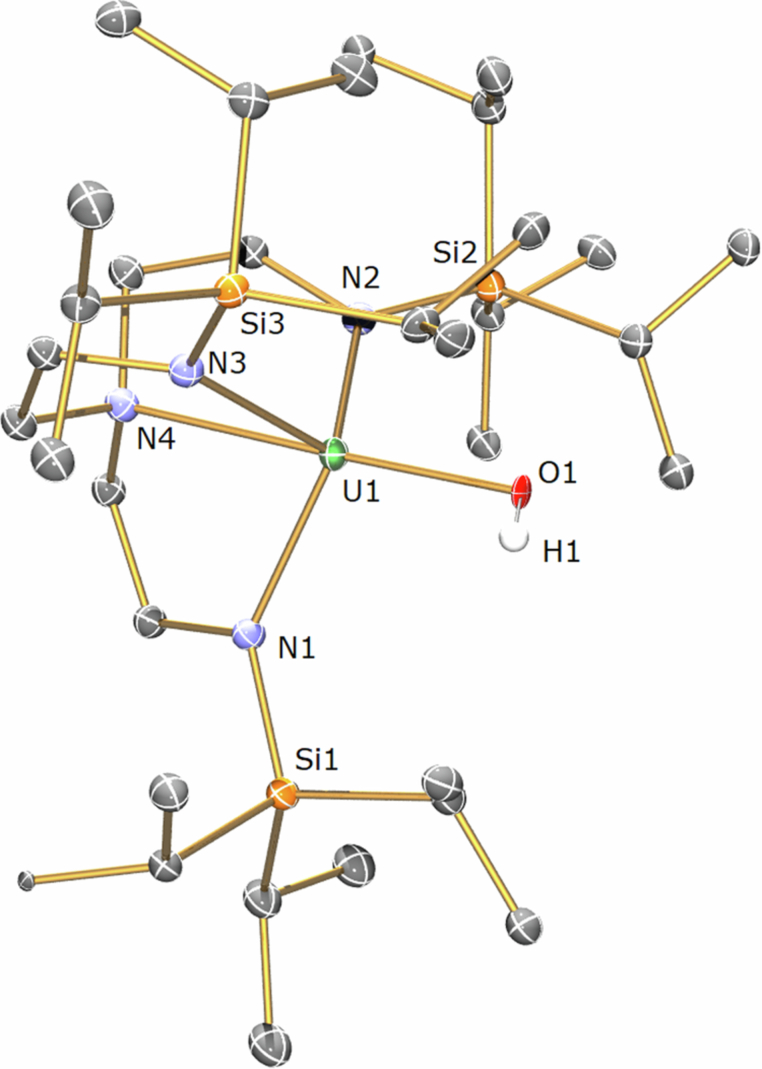
Single crystal X-ray diffraction solid state structure of 9. Data were collected at 100 K with displacement ellipsoids at 30% and selective labelling. Hydrogen atoms except the O-H are omitted for clarity.

**Extended Data Fig. 4:**
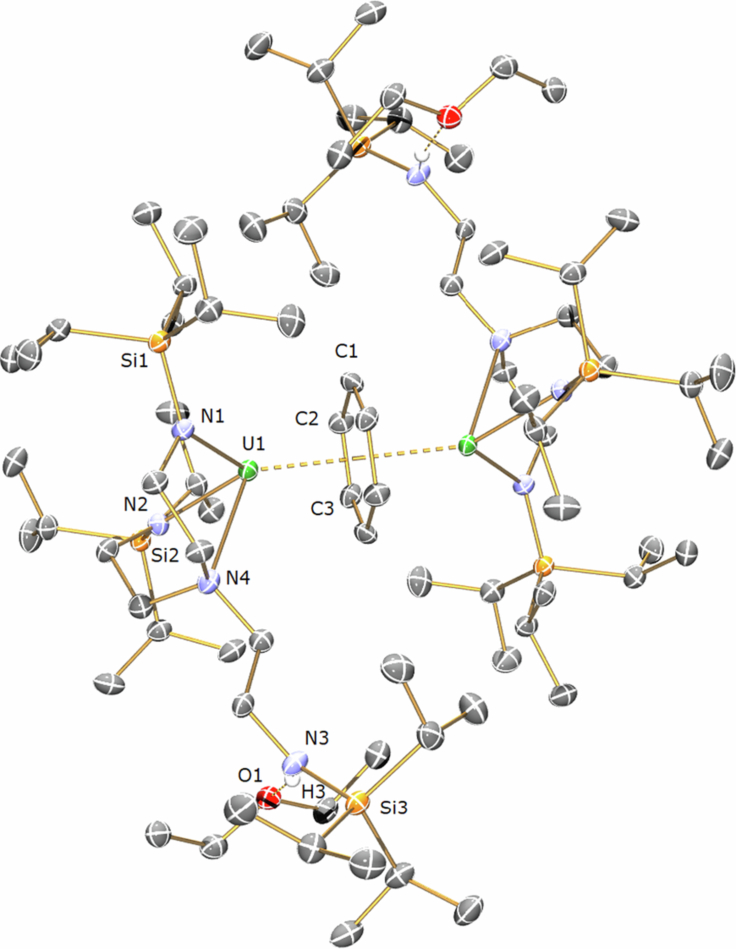
Single crystal X-ray diffraction solid state structure of 10. Data were collected at 100 K with displacement ellipsoids at 30% and selective labelling. Hydrogen atoms except the N-H are omitted for clarity.

Because the 11% co-crystallization of **6b** in **6a** is not ideal, we investigated how to avoid the formation of **6b** or remove it once formed. Changing the reaction solvent to toluene and 1-2-dimethoxyethane (DME) produced modestly different **6a**:**6b** ratios to those in benzene solvent (2:8 and 3:7, respectively), with diethyl ether producing a 1:1 co-crystal, and pentane, THF and pyridine all leading to decomposition (Supplementary Figs. 47–54 and Supplementary Table 1). Varying the reaction times was unsuccessful. Attempts to convert Bi^3−^ to RBi^2−^ (R = H, CPh_3_, SiMe_3_, SnPh_3_), substitute the counter-cation, or change the uranium oxidation state to change the solubilities of **6a** and **6b** were unsuccessful. Utilizing the starting material [K(2.2.2-cryptand)]_2_[Bi_2_] (**7**)^93^ under different reaction conditions variously resulted in the isolation of [{U^IV^(Tren^TIPS^)}_2_(μ-η^2^:η^2^-Bi_2_)] (**8**), [U^IV^(Tren^TIPS^)(OH)] (**9**) and [{U^III^(Tren^TIPS^H)}_2_(μ-η^6^:η^6^-C_6_H_6_)(OEt_2_)_2_] (**10**) (Extended Data Figs. 2–4) in multi-product reactions that frequently contained Tren^TIPS^H_3_ and other unidentified products (Supplementary Figs. 77-80). Utilizing K_5_Bi_4_ (**11**)^94^ in reactions with **1** or [U^IV^(Tren^TIPS^)(THF)][BPh_4_] variously resulted in mixtures containing **1**, Tren^TIPS^H_3_, **9** or [U^IV^(Tren^TIPS^)(μ-Cl)K(2.2.2-cryptand)] (**12**) (Supplementary Fig. 14). Although we could not eliminate co-crystallized **6b** from **6a**, these reactions support the presence of Bi^3−^ as well as cyclo-Bi_3_^3−^ and suggest that the reduction of uranium was complicating matters.

### Density functional theory analysis of 6b

**Fig. 3:**
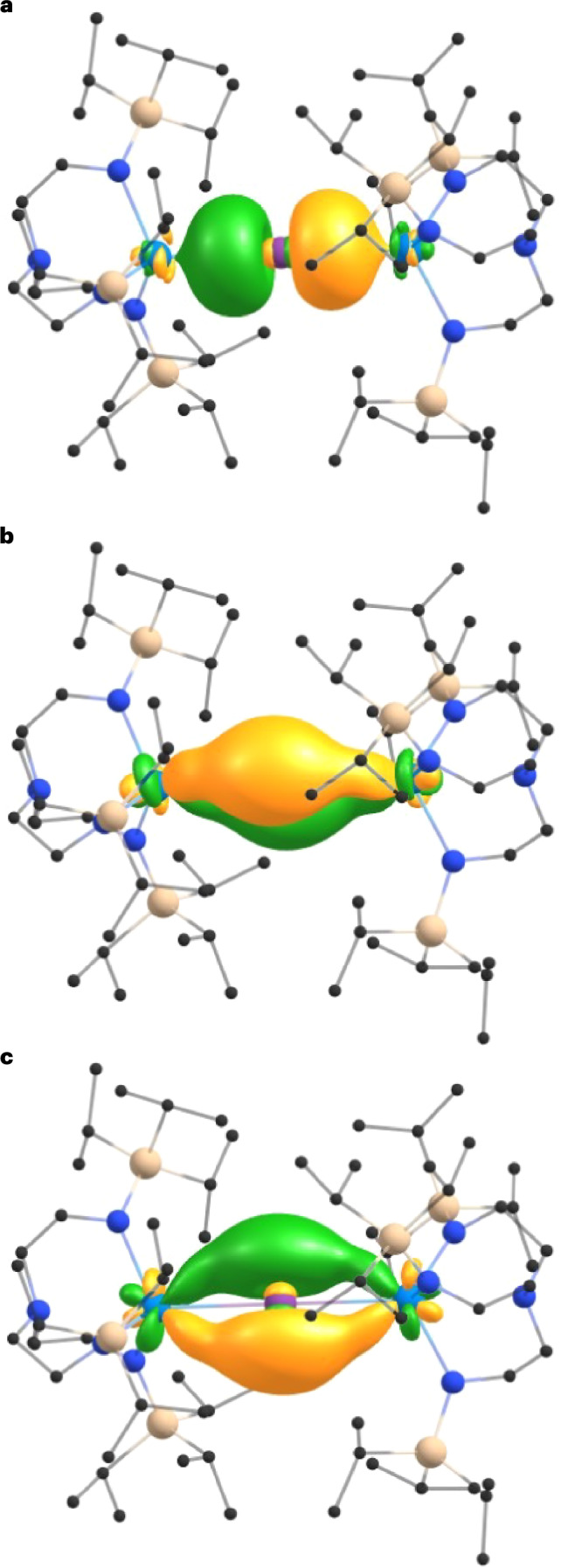
Pipek–Mezey localized orbitals of 6b′. **a**, U–Bi *σ*-bonding interaction. **b**, U–Bi π-bond. **c**, HOMO-6 U–Bi π-bond. Hydrogen atoms are omitted for clarity. Contours are drawn at ±0.04 atomic units (a.u.).

The isolation of a 1:1 co-crystal of **6a**:**6b** suggested that **6b** was worthy of further investigation. Density functional theory (DFT) calculations on the anion of **6b** (**6b′**) revealed a spin quintet formulation (four unpaired α-spin 5*f* electrons, 6.9 kJ mol^−1^ lower in energy than the broken-symmetry state) with key bond distances to within 0.05 Å of the solid-state values. The DFT calculations reveal three three-centre two-electron U–Bi bonding interactions (σ^2^π^2^π^2^) (Fig. 3). From Pipek–Mezey localization followed by Mulliken population analysis of- **6b′**, the U–Bi σ-bond has 62.4% bismuth character (100% 6*p*) and two uranium contributions of 17.3% each (22/11/56/11% 7*s*/7*p*/6*d*/5*f*). The two orthogonal U–Bi π-bonds each have 66.9% bismuth character (100% 6*p*), with two equal uranium contributions totalling 30.2% per π-bond (42/58% 6*d*/5*f*). These polar interactions result in U–Bi Wiberg bond indices (WBIs) of 1.31 (cf. 0.84 for the U–N_amide_ bonds), and hence the U–Bi bonds in **6b** extend the covalent multiple bonding chemistry of uranium from the fifth to sixth period of the periodic table^88,95,96^.

### Synthesis and solid-state structure of a dithorium cyclo-Bi_3_^3−^ complex

**Fig. 4:**
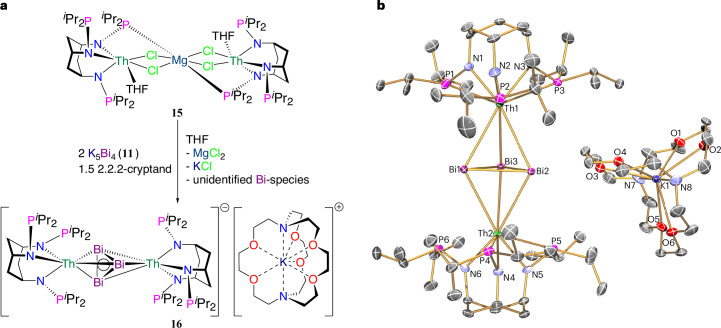
Synthesis and structure of 16. **a**, Synthesis of dithorium cyclo-Bi_3_^3−^ complex **16** from the reaction of **11** with **15**. **b**, Molecular structure of **16** at 150 K with 20% probability ellipsoids and hydrogen atoms, a disordered ^*i*^Pr group, and lattice solvent omitted for clarity.

Given the aforementioned synthetic complications, we refocused our approach on an alternative triamido thorium system. We prepared the proligand C_6_H_9_-1,3,5-{N(H)P^*i*^Pr_2_}_3_ (**13**, Tach^DIPP^H_3_), lithiated it to give (Tach^DIPP^Li_3_) in situ, then installed it onto thorium to give [{(Tach^DIPP^)ThCl}_2_(μ-LiCl)] (**14**) and ultimately [{(Tach^DIPP^)ThCl(THF)}_2_(μ-MgCl_2_)] (**15**) (Supplementary Figs. 15, 16 and 88–97). Treatment of **15** with **11** produced dark green/black [K(2.2.2-cryptand)][{(Tach^DIPP^)Th}_2_(μ-η^3^:η^3^-Bi_3_)] (**16**) in a reproducible crystalline yield of 22% (by thorium) (Fig. 4a). The solid-state structure of **16** (Fig. 4b) reveals Bi–Bi distances that span the range 3.0112(5)–3.0253(5) Å, slightly longer than the Bi–Bi distances in **6a**, but consistent with a cyclo-Bi_3_^3−^ trianion. The six Th–Bi distances (3.3921(4)–3.4757(4) Å) are slightly longer than the sum of the single-bond covalent radii of thorium and bismuth (3.26 Å)^84^ due to the π-bridging mode of the cyclo-Bi_3_^3−^ unit. The characterization data for **16** (Supplementary Figs. 98–100, 117, 124 and 125) support its formulation and its phase purity. Complex **16** provides an independent reference for **6a** as well as being worthy of investigation itself.

### Molecular orbital analysis and experimental benchmarking

For deeper insight into the electronic structures of **6a** and **16**, DFT calculations were performed on the (mono)anion components of **6a** (**6a′**) and **16** (**16′**), as well as the hypothetical thorium analogue of **6a′** (**6a′**(**Th**)) for benchmarking purposes, revealing the ground states to be the anticipated spin–quintet (two α-spin unpaired 5*f* electrons per uranium, consistent with the magnetic data) and spin–singlet formulations, respectively. For **6a′**, the spin–septet state was calculated to be 42 kJ mol^−1^ higher in energy than the spin–quintet. The equilibrium geometry data for these spin formulations agree well with the solid-state crystal structures, with bond distances computed to within 0.03 Å. The structure of **6a′** was then symmetrized to *C*_3_ symmetry to make the following analysis as clear as possible. The U/Th–Bi, U/Th–N and Bi–Bi Wiberg bond orders are 0.42–0.44, 0.82–0.87 and 0.83 (**6a′**) and 0.48–0.53, 0.69 and 0.84–0.86 (**16′**), suggesting slightly more developed Th–Bi bonding interactions compared to the U–Bi ones, but more developed U–N bonds compared to the Th–N linkages. For comparison with **6b′**, the Pipek–Mezey orbitals of **6a′** reveal three identical U–Bi π interactions, each composed of 79.3% bismuth character (100% 6*p*) and two equal uranium contributions totalling 20% (52/48% 6*d*/5*f*).

**Fig. 5:**
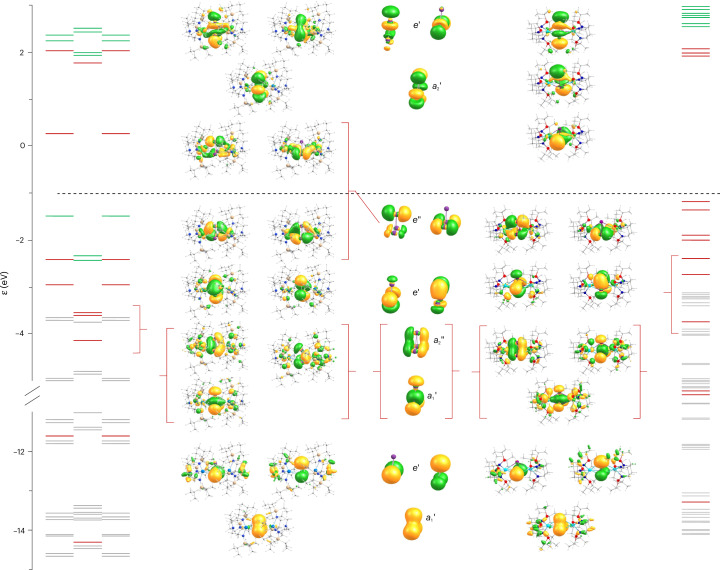
Energies and shapes of occupied canonical molecular orbitals of 6a′, cyclo-Bi_3_^3^^−^ and 16′. Energies and shapes of occupied canonical molecular orbitals of **6a′** (left), cyclo-Bi_3_^3^^−^ (centre) and **16′** (right). For **6a′**, only the majority spin orbitals are shown for simplicity. In the energy level diagram, the molecular orbitals that are depicted are represented by red bars, the four MOs containing the 5*f*-electrons of the two U atoms in **6a′** are shown as green bars, and the other orbitals are shown as grey bars. Contours are drawn at ±0.025 a.u. (±0.05 a.u. for bare cyclo-Bi_3_^3−^).

**Extended Data Fig. 5:**
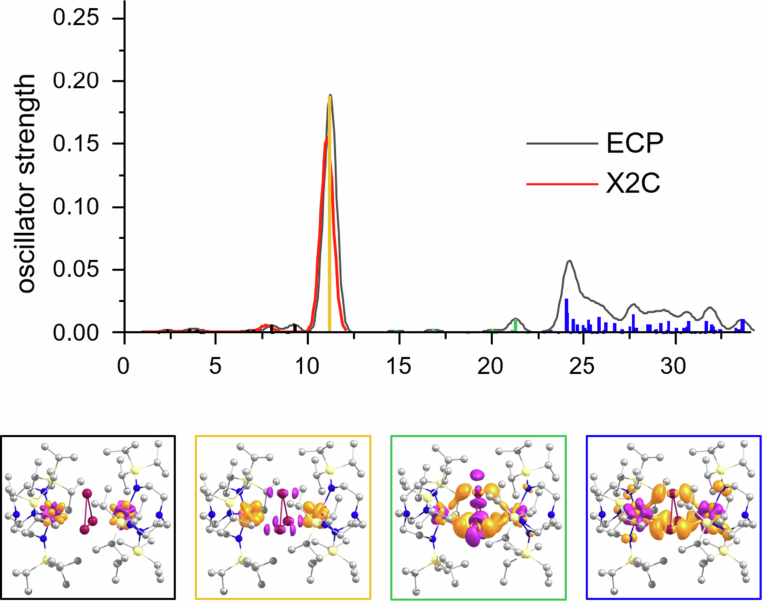
Experimental and computed electronic absorptions for 6a’. With ECPs, 50 excitations are calculated for both irrep a and irrep e, with X2C 10 per irrep. For ECPs, both individual excitations and superimposed Gaussians (FWHM = 800 cm^−1^) are shown, for X2C only the latter. Composite excess density for the ground and excited states are plotted in orange and purple, respectively. Contours are drawn with ±0.001 a.u., and each colour denotes a group of excitations that constitute a composite that is energetically well separated from the other groups. The difference density plots provide an aggregated representation of the electron transfer for each excitation group containing weighted contributions of each excitation within the group by its oscillator strength.

**Extended Data Fig. 6:**
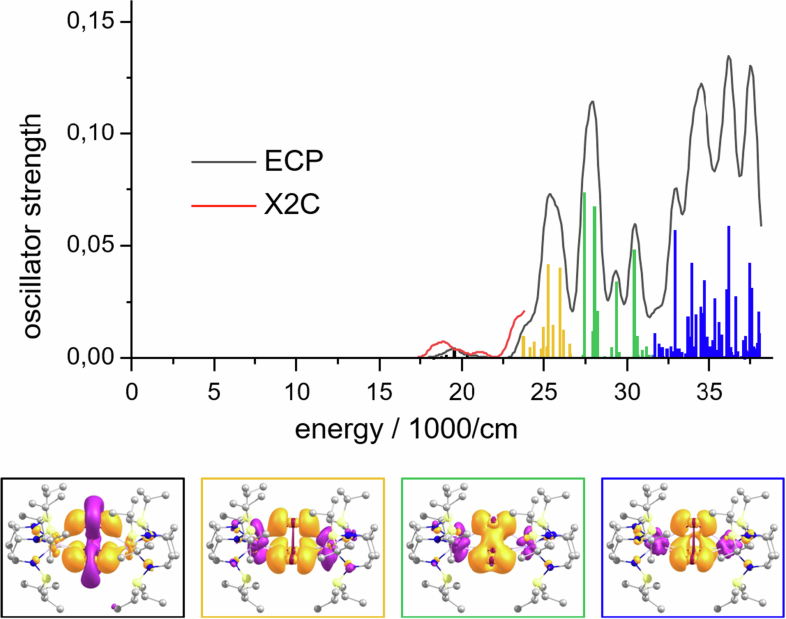
Experimental and computed electronic absorptions for 16’. The computed absorptions (sticks) are the lowest 100 (with ECPs) and lowest 20 (X2C) spin-conserving excitations.

Time-dependent-DFT (TD-DFT) calculations for **6a′** and **16′** (Extended Data Figs. 5 and 6 and Supplementary Tables 12 and 13) reproduce the key features of the solution UV–vis–NIR spectra. Moreover, the solid-state UV–vis–NIR spectrum of **6** has a similar profile overall to the solution spectra, suggesting that the solution spectrum is representative of the solid-state structure. The spectra of **6** and **16** share common features: strong absorptions starting at ~20,000 cm^−1^ from excitations from π(Bi_3_^3−^) to empty 5*f*-orbitals, and weaker absorptions at 12,000–20,000 cm^−1^ from excitations from π(Bi_3_^3−^) to anti-bonding σ*(Bi_3_^3−^) (the lowest unoccupied molecular orbitals (LUMOs) of **16′**; see also Fig. 5). The latter are blueshifted for **16** compared to **6**. For **6a**, additional excitations below ~12,000 cm^−1^ from transitions within the uranium 5*f*-manifold are found^97^, in some cases with admixtures from π(Bi_3_^3−^) that account for the unusually large intensities of these absorptions due to intensity-stealing. The DFT calculations for **6a′** and **16′** reproduce the UV–vis–NIR and magnetic data, so are experimentally validated.

**Extended Data Fig. 7:**
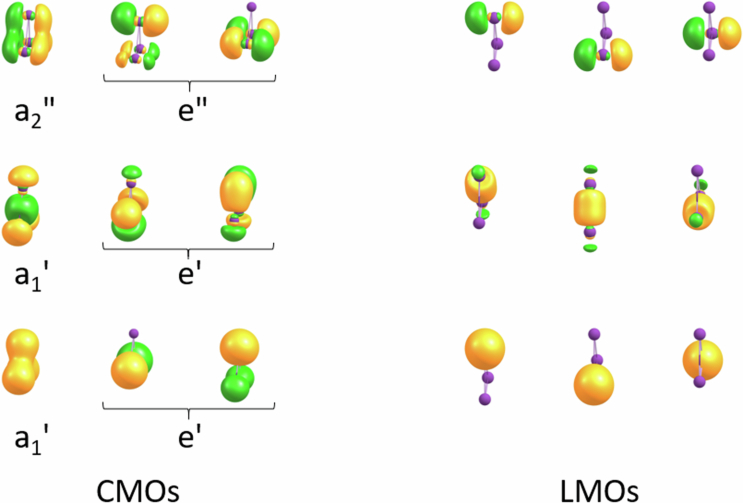
Canonical and localised molecular orbitals of Bi_3_^3–^. Left: canonical molecular orbitals (CMOs). Right: localised molecular orbitals (LMOs) from a Pipek–Mezey localisation procedure. Contours are rendered at 0.05 a.u. with bismuth atoms in purple.

Complexes **6a′** and **16′** are best considered as contiguous cyclo-Bi_3_^3−^ and {M^IV^(Tren^TIPS^)}^+^ or {Th^IV^(Tach^DIPP^)}^+^ (M = U, Th) units, because the characteristic valence MOs of cyclo-Bi_3_^3−^ (*D*_3h_ symmetry) are also found in the cyclo-Bi_3_^3−^ units of the entire systems (Fig. 5). Specifically (Extended Data Fig. 7), the 6*s*-orbitals in the cyclo-Bi_3_^3−^ ring form *a*_1_′ and *e*′ representations, which transform to one *s*-type lone pair per Bi atom by a localization procedure. Three in-plane 6*p*-orbitals form *a*_1_′ and *e*′ representations, yielding three σ-bonds by localization. The three perpendicular 6*p*-orbitals form an a_2_′ representation when combined with the same phase, and an *e*′ representation (the highest occupied molecular orbital (HOMO)) when combined with different phases. These three orbitals can be transformed to one *p*_π_-type lone pair per bismuth atom. These orbitals are found for the whole of **6a′** as well as for **16′**, with a slight delocalization toward the U/Th atoms for *a*_2_′ and *e*′ as well as for *a*_1_′ formed by the in-plane 6*p*-orbitals (Fig. 5). They are somewhat lower in energy than the occupied uranium 5*f*-orbitals. We note that the electronic situation in hypothetical *D*_3h_-symmetric cyclo-Bi_3_^3−^ is very similar to that in (C_3_H_3_)^3−^, the main difference in the latter being the change from *s*-type lone pairs to CH bonds. However, this species is not a minimum structure and it is highly unstable, for example, with respect to decomposition into H^−^ and linear (C_3_H_2_)^2−^ (energy gain of 618 kJ mol^−1^). This is distinct from (C_3_H_3_)^+^, where the triangular *D*_3h_-symmetric isomer is calculated to be the most stable (412 kJ mol^−1^ more stable than linear (HC_3_H_2_)^+^). Also, for the three-membered bismuth rings, the mono-cation, where only the *p*_π_-orbitals’ *a*_2_′ bonding combination is occupied, is much more stable in terms of bond indices, bond distances and vibration frequencies than the trianion, where the *e*′ combination is also occupied, in total equivalent to three *p*_π_-lone pairs. WBIs for Bi_3_^+^/Bi_3_^3−^ thus amount to 1.4/1.0, distances to 289/309 pm, symmetric stretch vibration to 172/127 cm^−1^ and the two other (degenerate) frequencies to 122/91 cm^−1^. Notably, the data for Bi_3_^3−^ are more similar to those of Bi_3_^3+^, where all *p*_π_-orbitals are unoccupied (WBI of 1.0, Bi–Bi distance of 314 pm, frequencies of 142 and 101 cm^−1^). In **6a′** and **16′**, the unoccupied actinide *f*- and *d*-orbitals support delocalization of the electrons in the *p*_π_-orbitals. This reduces Coulomb repulsion in the ring, leading to shorter Bi–Bi distances compared to bare Bi_3_^3−^, but its electronic structure is essentially unchanged.

### Ring current analysis

**Fig. 6:**
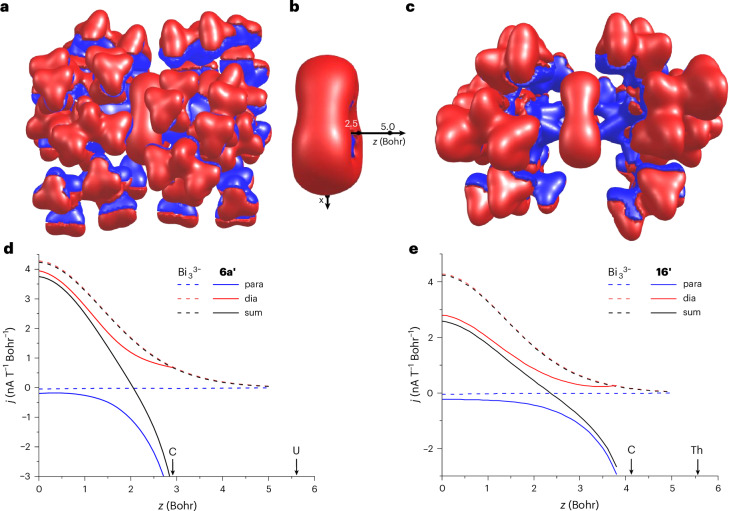
Magnetic field-induced ring current densities and profiles for **6a′**, cyclo-Bi_3_^3^^−^ and **16′** induced by a magnetic field in the *z* direction. **a**, Ring current density for **6a′**. **b**, Ring current density for cyclo-Bi_3_^3−^. **c**, Ring current density for **16′**. Regions with diatropic current density are depicted in red, and those with paratropic current density are in blue. Contours are drawn at 0.02 a.u. **d**, Current profile for **6a′**. **e**, Current profile for **16′**. The current profiles result from integration in the *x* and *y* directions for the cyclo-Bi_3_^3^^−^ unit (dashed lines) and for the whole structures **6a′/16′** (solid lines). Diatropic (red) and paratropic (blue) contributions are plotted separately as well as in total (black). ‘Th’ and ‘U’ mark the position of those ions above the ring plane, ‘C’ is the C atom closest to the ring plane.

The perfectly (**6a**) and nearly (**16**) symmetrical cyclo-Bi_3_^3−^ units in **6a** and **16** prompted us to explore the potential aromatic character of these cyclo-Bi_3_^3−^ units. Contour plots of the magnetically induced current density for cyclo-Bi_3_^3−^ and for **6a′** and **16′** (Fig. 6a and 6c), and integrated values for slices parallel to the ring plane (*x*–*y* plane), which are 0 to 6 Bohr away from the ring (current profiles) (Fig. 6b), enable determination of whether cyclo-Bi_3_^3−^, **6a′** and **16′** exhibit all-metal aromaticity.

**Extended Data Fig. 8:**
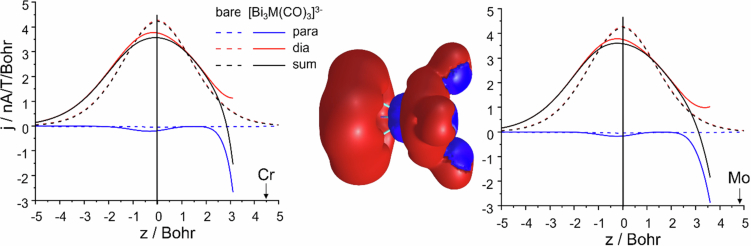
Ring current profiles for [(η^3^-cyclo-Bi_3_)M(CO)_3_]^3–^ (M = Cr, Mo). Left: profile for M = Cr. Middle: ring current density for M = Mo. Right: profile for M = Mo. Current profiles result from the integration in x- and y- direction for Cr and Mo (straight lines) and for comparison for cyclo-Bi_3_^3**−**^ (dotted). Diatropic and paratropic contributions are plotted separately (red/blue) as well as in total (black). ‘Cr’ and ‘Mo’ mark the position of the transition metal atoms above the ring plane. In the current density plot, middle, regions with diatropic current density are marked in red, those with paratropic current density in blue. Contours are drawn at 0.02 a.u.

For cyclo-Bi_3_^3−^, integration in the *z* direction yields a total ring current of 16.2 nA T^−1^. This is more than for, for example, C_6_H_6_ (11.8 nA T^−1^), cyclo*-*Bi_4_^4+^ (9.1 nA T^−1^)^49^ and cyclo-Bi_5_^−^ (14.4 nA T^−1^)^50^, even though the electronic situation in cyclo-Bi_3_^3−^ can be described by localized two-centre σ-bonds and *p*_π_ (and *s*) type lone pairs (see earlier), in contrast to C_6_H_6_, which resists localization to two-centre bonds and lone pairs (due to the six electrons being over six 2*p*-orbitals rather than three 6*p*-orbitals); moreover, it is not much less than in 2π-delocalized cyclo-Bi_3_^+^ (17.5 nA T^−1^), and not dramatically more than in 0π-cyclo-Bi_3_^3**+**^ (10.9 nA T^−1^), pointing to predominantly σ-type aromaticity. For the whole of **6a′**, as well as **16′**, the current density close to the cyclo-Bi_3_^3−^ ring (≤2 Bohr below or above the ring plane) is only slightly smaller than that of the isolated ring (12.9 nA T^−1^), as can be seen from comparison of the current profile dashed and solid black lines in Fig. 6d. Beyond 2 Bohr, strong paratropic atomic currents in **6a′** arise from the uranium(IV) ions, resulting in negative current profile values. For **16′**, calculating the system without the cyclo-Bi_3_^3−^ ring gives nucleus-independent chemical shifts (NICS 0/1/2/3) values at/above the centre of 0.08/0.06/0.09/1.09 ppm, and an integrated current density between −2 and +2 Bohr (that is, the same boundaries as for the whole system **16′**) of 0.03 nA T^−1^. Therefore, we conclude that there is not a strong ‘through space’ influence on the cyclo-Bi_3_^3−^ ring from the thorium atoms and everything beyond. Integration up to the 2-Bohr point results in a total ring current of 9.9 nA T^−1^ for **6a′** and 7.1 nA T^−1^ for **16′** (area below the solid black lines in Fig. 6d,e). These data prompted us to examine [(η^3^-cyclo-Bi_3_)M(CO)_3_]^3−^ (M = Cr, **17′**; Mo, **18′**), yielding integrated total ring current densities of 14.9 and 15.2 nA T^−1^, respectively (upper bound of 2 Bohr above the ring), rising to 16.0/16.6 nA T^−1^ if the upper bound is the zero crossing of the current density at 2.8/3.1 Bohr (Extended Data Fig. 8). These data suggest that neighbouring ligand substituents and the binding mode (terminal versus inverse sandwich) apparently induce modest Bi–Bi distance (*Δ*_BiBi_ = 0.05 Å) and ring current strength variations, but the overall situation is always the same: three σ-type single bonds in the Bi_3_^3−^ fragment with a substantial σ-type ring current (isolated ring: ~10 nA T^−1^) and three π-type lone pairs delocalized towards empty orbitals at M being responsible for the remaining current.

**Extended Data Fig. 9:**
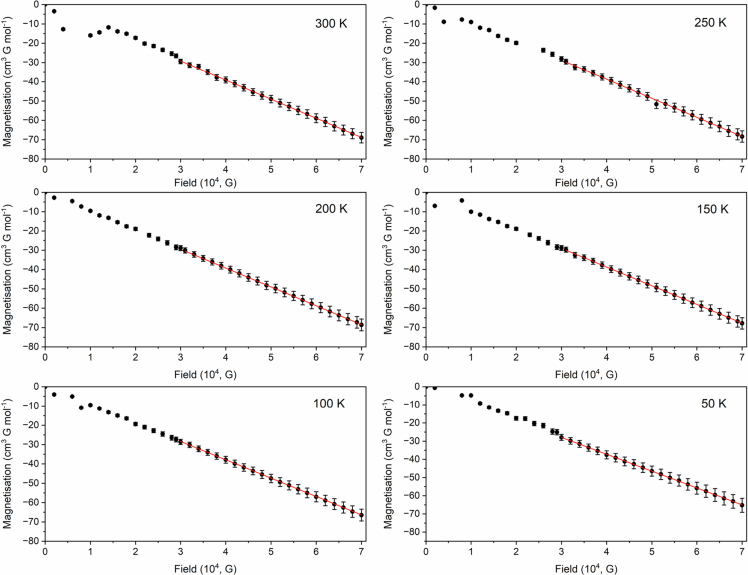
Magnetisation vs Field data for 16 over the 0-7 Tesla range. Top left: 300 K (*R*^2^ = 0.999; average error = 3.93 %). Top right: 250 K (*R*^2^ = 0.999; average error = 4.28 %). Middle left: 200 K (*R*^2^ = 0.999; average error = 4.41 %). Middle right: 150 K (*R*^2^ = 0.999; average error = 4.36 %). Bottom left: 100 K (*R*^2^ = 0.998; average error = 4.60 %). Bottom right: 50 K (*R*^2^ = 0.998; average error = 5.91%). The straight lines are linear regressions of the data points over the range 30,000 Gauss to 70,000 Gauss (10,000 Gauss = 1 Tesla). Errors derive from the standard deviation of the sampling distribution of the points used (sample population divided by the square root of the number of samples) to compute the reported moment represented as a percentage.

Given earlier work^52^ that described exalted diamagnetism *Λ* (ref. ^98^), that is, diamagnetism additional to that anticipated from cumulative Pascal’s constants, and hence aromaticity, of trithorium clusters^52^, we undertook magnetic investigations of **16** (Supplementary Figs. 126 and 127 and Supplementary Tables 6 and 7), noting that any *Λ* in **6a** would be masked by its strong paramagnetism. Complex **16** is confirmed to be diamagnetic, and presents slightly positive and temperature-independent molar magnetic susceptibility (*χ*_M_) and molar magnetic susceptibility temperature product (*χ*_M_*T*) versus *T* plots that indicate weak van Vleck paramagnetism (*χ*_TIP_ = 8.76 × 10^−5^ cm^3^ mol^−1^). Plots of magnetization versus field produce negative, linear field-dependent gradient plots, confirming the dominant diamagnetism of **16**, and a *Λ* value of 63.36 × 10^−6^ cm^3^ mol^−1^ (5.9% increase) was extracted (Extended Data Fig. 9). The *Λ* value for **16** can be compared to *Λ* values of 0 and 13.7 × 10^−6^ cm^3^ mol^−1^ for cyclohexane and benzene, respectively^99^, and is consistent with the corresponding computed ring current values (see earlier). Thus, the magnetic data for **16** are consistent with modest exalted diamagnetism and the presence of all-metal aromaticity.

To extend our comparisons, we calculated other 6π-systems, namely isoelectronic cyclo-Sb_3_^3−^, cyclo-E_3_ (E = Te, Po), closely related (C_3_H_3_)^3−^ and the corresponding 2π-electron systems (cyclo-Bi_3_^+^, cyclo-Sb_3_^+^ and (C_3_H_3_)^+^) (Supplementary Tables 8–11). The resulting total ring currents span a relatively modest range of ~10 to ~18 nA T^−1^. It is noteworthy that the corresponding 6π–2π differences are <2 nA T^−1^, establishing a link from classical (C_3_H_3_)^+^ to (C_3_H_3_)^3−^, and hence cyclo-Bi_3_^3−^. The NICS^2^ 0 and 1 values are all negative (−42 to −5 ppm), and for cyclo-Bi_3_^3−^ the NICS 0 and 1 values are −42 and −33 ppm, respectively, exceeding those of C_6_H_6_ (−7, −9 ppm), (C_3_H_3_)^3−^ (−37, −5 ppm) and (C_3_H_3_)^+^ (−22, −28 ppm). For the 6π-systems, with the exception of benzene, ring centre values of ~−40 ppm are found, which rapidly decrease in absolute values with increasing distance from the ring centre. Nevertheless, the relevance of these metrics should not be overestimated for such small rings. In contrast, for 6π-C_6_H_6_ and the 2π-systems, the ring centre values are smaller (−8 to −22 ppm) than in cyclo-Bi_3_^3−^, but they decrease more slowly with increasing distance from the ring centre, reflecting greater π-contributions to their ring currents. Increasing the number of π-electrons, for example, in more covalently bound, and hence less well-isolated than in **6a**, classically π-bonded systems, will not necessarily lead to a higher π-contribution to the current; if the additional occupied orbitals lead to a situation where localization to lone π-type pairs is possible, the π-contribution becomes smaller. Accounting for spin–orbit coupling leads to changes in the NICS, the magnitude of which depends on the system. Negligible changes are observed for CH systems, with comparably small reduction of NICS by ~10% for the 6π-systems of the fifth and sixth periods. By contrast, for the 2π-systems reductions are larger, for example, −15 ppm to −8 ppm for NICS 0 in Sb_3_^+^, and −14 ppm to +8 ppm for Bi_3_^+^. Of course, given the documented potential drawbacks of NICS with small heavy-element rings^2^, these numbers should not be given undue weight, but they clearly suggest that the aromaticity of these 2π-systems is weakened, if not destroyed, by spin–orbit coupling.

As well as evidencing delocalized electron density in a closed-ring system, the cyclo-Bi_3_^3−^ ring—be it isolated or in **6a** or **16**—exhibits similar Bi–Bi bonds, is isolable, is generally preferentially formed in **6a** and certainly in **16** compared to other oligomeric forms, and exhibits magnetically induced ring currents. To probe any enhanced energetic stability, the potential energy surfaces of cyclo-Bi_3_^3−^, cyclo-Sb_3_^3−^, cyclo-E_3_ (E = Te, Po), (C_3_H_3_)^3−^ and (C_3_H_3_)^+^ were investigated (Supplementary Table 10). In all cases, two minima are found: cyclic equilateral triangles and acyclic bent structures (a third, linear structure is a saddle point). In the bent structure, for the central atom only the s-type lone pair is present, while the other here is involved in bonds to the neighbours. Nevertheless, the remaining lone pair prevents the linear structure from being a minimum. As noted above, the related *D*_3h_-symmetric (C_3_H_3_)^3−^ is unstable, whereas for (C_3_H_3_)^+^ the cyclic *D*_3h_-symmetric isomer is most stable.

## Summary and conclusions

The cyclo-Bi_3_^3−^ rings in **6a** and **16** are highly symmetric, and preferentially formed, implying lowered reactivity and enhanced stability. The cyclo-Bi_3_^3−^ ring exhibits substantial computed ring currents, greater than for cyclo*-*Bi_4_^4+^, cyclo-Bi_5_^−^, 6π-C_6_H_6_, 2π-(C_3_H_3_)^+^ or 6π-(C_3_H_3_)^3−^, and even when proximate to strong paratropic uranium currents the 6π-cyclo-Bi_3_^3−^ ring current ±2 Bohr from the cyclo-Bi_3_^3−^ centroid remains substantial. That the computed ring current for the cyclo*-*Bi_3_^3−^ unit in **16′** is moderately less than that of **6a′** can be related to the longer Bi–Bi distances in the former compared to the latter. Comparison to [(η^3^-cyclo-Bi_3_)M(CO)_3_]^3−^ (M = Cr, Mo) demonstrates that the coordination mode of cyclo-Bi_3_^3−^ and peripheral ligand substituents are also important. The exalted diamagnetism of **16** supports the presence of all-metal aromatic bonding, and the experimental and computational data are internally consistent for **16** and benzene. Calculations reveal similar ring current values for 6π-cyclo-Bi_3_^3−^, 2π-cyclo-Bi_3_^+^ and 0π-cyclo-Bi_3_^3+^, demonstrating a dominance of σ- over π-aromaticity in cyclo-Bi_3_^3−^ that is notable given how favourable it is to describe cyclo-Bi_3_^3−^ with localized bonds. Confirmation of all-metal aromaticity in 6π-cyclo-Bi_3_^3−^ provides confirmation of the heaviest aromatic metal–metal bonded 6*p* analogue to (cyclo-C_3_H_3_)^+/3−^ with contrasting σ- and π-aromaticities, respectively, complementing recent reports of acyclic (RBi_3_R)^+^, cyclo*-*Bi_4_^4+^ and cyclo-Bi_5_^−^, and permits aromaticity in [K(2.2.2-cryptand)]_3_[(η^3^-cyclo-Bi_3_)M(CO)_3_] (M = Cr, Mo) to be recognized. Thus, this work provides benchmark data for organic–inorganic aromaticity comparisons and further elaborates our understanding of aromaticity.

## Methods

### General

Experiments were carried out under a dry, oxygen-free dinitrogen atmosphere using Schlenk-line and glove-box techniques. All solvents and reagents were rigorously dried and deoxygenated before use. Compounds were variously experimentally characterized by single-crystal X-ray diffraction, NMR, FTIR, Raman and UV–vis–NIR spectroscopies, SQUID magnetometry, and elemental analysis. Compounds were theoretically probed using DFT, TD-DFT, NICS and GIMIC calculations. Further details are provided in the Supplementary Materials and Methods.

## Online content

Any methods, additional references, Nature Portfolio reporting summaries, source data, extended data, supplementary information, acknowledgements, peer review information; details of author contributions and competing interests; and statements of data and code availability are available at 10.1038/s41557-026-02123-8.

## Supplementary information

Supplementary Information Experimentals; Supplementary Figs. 1–136, Tables 1–13 and references.

Supplementary Data Optimized Cartesian coordinates for **6a′**, **6a′(Th)**, **6b′** and for **16′**.

## Data Availability

Crystallographic data for the structures reported in this Article have been deposited at the Cambridge Crystallographic Data Centre, under deposition nos. CCDC 2270435 (**6a6b-1**), 2491429 (**6a6b-2**), 2491430 (**6a6b-3**), 2491431 (**6a6b-4**), 2491432 (**8**), 2491433 (**9**), 2491434 (**10**), 2492183 (**12**), 2491451 (**14**), 2491452 (**15**) and 2491453 (**16**). Copies of the data can be obtained free of charge via https://www.ccdc.cam.ac.uk/structures. All the other data supporting the findings of this study are available within the Article or its Supplementary Information (Supplementary Figs. 1–136 and Supplementary Tables 1–13). DFT data are compiled in Supplementary Data File 1. Characterization data have been deposited in the Figshare database (10.48420/31378627)^100^. Source data are provided with this paper.
