# Supplementary material for: All-metal aromaticity of cyclo-Bi33− in diuranium and dithorium inverse-sandwich-type complexes
Source: Nat Chem. 2026 Apr 20;18(7):1308–17. doi: 10.1038/s41557-026-02123-8 (PMC13322972; doi:10.1038/s41557-026-02123-8)
Supplement: Supplementary file 1 — Experimentals; Supplementary Figs. 1–136, Tables 1–13 and references. [file 41557_2026_2123_MOESM1_ESM.pdf]

nature chemistry

Supplementary information https://doi.org/10.1038/s41557-026-02123-8

All-metal aromaticity of cyclo-Bi_3^{3-} in diuranium and dithorium inverse-sandwich-type complexes

In the format provided by the authors and unedited

Table of Contents

Supplementary Materials and Methods……………………………………………………….1

Supplementary Figures…………………………………………………………………...40

Supplementary Tables………………………………………………………………..118

Supplementary References…………………………………………………………...128

Supplementary Materials and Methods

General Experimental Details

All manipulations were carried out under an inert atmosphere of dry dinitrogen using Schlenk techniques, or an MBraun UniLab or Vigor glovebox operating under an atmosphere of dry dinitrogen. Methyl-*tert*-butyl ether, DCM, Et_2O, THF, toluene, hexane, and pentane solvents were dried by passage through activated alumina towers and degassed before use. Benzene and DME were distilled from potassium. Pyridine was distilled from CaH_2. Ethane-1,2-diamine was refluxed over CaH_2 for 24 hour and then distilled onto 4 Å molecular sieves. All solvents were stored over potassium mirrors except for ethers which were stored over activated 4 Å sieves. Deuterated solvents were distilled from potassium, degassed by three freeze-pump-thaw cycles, and stored under dinitrogen prior to use. $[U^{III}(Tren^{TIPS})]$ (**1**),[1] $[U^{IV}(Tren^{TIPS})Cl]$,[1] $[U^{IV}(Tren^{TIPS})(THF)][BPh_4]$,[2] $AgBPh_4$,[3] CPh_3BPh_4,[4] $[HNEt_3][BPh_4]$,[5] $TlBPh_4$,[6] KC_8,[7] [K(2.2.2-cryptand)]$_2$[Pb_2Bi_2] (**2**),[8] [K(2.2.2-cryptand)]$_2$[MBi_3] (M = Ga (**3**), In (**4**), Tl (**5**)),[9,10] [K(2.2.2-cryptand)]$_2$[Bi_2] (**7**),[11] *cis,cis*-1,3,5-triaminocyclohexane,[12] K_5Bi_4 (**11**),[13] *cis,cis*-1,3,5-*tris*-[{(benzyloxy)carbonyl}aminocyclohexane, *cis,cis*-1,3,5-triamino-cyclohexane•3HBr, KCH_2Ph,[14] $Tren^{TIPS}H_3$,[1] $Tren^{TIPS}Li_3$,[1] $U^{IV}Cl_4$,[15] and $[U\{N(CH_2CH_2NSiPr^i_3)_2(CH_2CH_2SiPr^i_2CHMeCH_2)\}]$[16] were prepared using modified literature methods described below. K_2TlBi_3, "KPbBi", $K_5Ga_2Bi_4$, and "$K_5In_2Bi_4$" were prepared as previously reported.[8,9,17] 2.2.2-cryptand and triphenyltin chloride was purchased from Sigma Aldrich and dried under dynamic vacuum (1×10^{-3} mbar) at 50 ºC for 24 hours prior to use. The following metals were purchased from the suppliers listed in parentheses along with respective percentage of purities: Bi

(ChemPur, 99.5%), Ga (Roth, 99.99%), In (Roth, >99%), Tl (ChemPur, 99.9%), Pb (Roth, 99.95 %). Elemental potassium was freed from oxides and washed with hexane to remove mineral oil prior to use. Depleted UO_3 was supplied by the National Nuclear Laboratory. All other chemicals were purchased and used as received.

Single crystals were examined on either a Rigaku XtalLAB Synergy-S/R diffractometers equipped with Hypix 6000HE photon counting pixel array detectors with mirror-monochromated CuKα radiation (λ = 1.54184 Å) or MoKα radiation (λ = 0.71073). Intensities were integrated from a sphere of data recorded on narrow (Cu: 0.5 or 1.0°; Mo: 0.2 or 0.5°) frames by ω rotation. Cell parameters were refined from the observed positions of all strong reflections in each data set. Gaussian grid face-indexed absorption corrections with a beam profile correction were applied. The structures were solved using SHELXT[18] and all non-hydrogen atoms were refined by full-matrix least-squares on all unique F^2 values with anisotropic displacement parameters (with exceptions noted in the respective cif files). Hydrogen atoms were refined with constrained geometries and riding thermal parameters; $U_{iso(H)}$ was set at 1.2 (1.5 for methyl groups) times U_{eq} of the parent atom. The Bi_3 unit in **6a**/**6b** was modelled anharmonically ("anis -a=3") as this approach has been proven to be effective in the modelling of bridging tripnictide units.[19] The largest features in final difference syntheses were close to heavy atoms and are of no chemical significance. CrysAlisPro was used for control and integration,[20] and SHELXL and Olex2 were employed for structure refinement.[21,22] ORTEP-3 and POV-Ray were employed for molecular graphics.[23,24]

Powder X-ray diffraction (PXRD) measurements were conducted using a STOE StadiMP diffractometer system equipped with a Mythen 1 K silicon strip detector and a Cu Kα radiation source (λ = 1.54056 Å). Samples were filled into a glass capillary (0.5 mm diameter), which was then sealed to be airtight with soft wax. The tube was then mounted onto the goniometer head using wax (horizontal set-up) and rotated throughout the measurements.

^{1}H, ^{7}Li, $^{11}B\{^{1}H\}$, $^{13}C\{^{1}H\}$, $^{29}Si\{^{1}H\}$, and $^{31}P\{^{1}H\}$ NMR spectra were recorded on either a Bruker 400 MHz spectrometer operating at 400, 155.5, 128.4, 100.6, 79.5, and 161.95 MHz, respectively; or, for ^{1}H, $^{11}B\{^{1}H\}$, and $^{29}Si\{^{1}H\}$, recorded on a JEOL JNM-ECZ 400 MHz spectrometer operating at 399.78, 128.27, and 79.42 MHz, respectively. Chemical shifts are quoted in ppm, relative to tetramethylsilane (^{1}H, ^{13}C, and ^{29}Si), 1 M aqueous LiCl (^{7}Li), $BF_3.OEt_2$ (^{11}B), or 85% H_3PO_4 (^{31}P).

ATR-IR spectra were recorded on a Bruker Alpha spectrometer with a Platinum-ATR module in the glovebox.

Raman spectra were recorded on a Horiba XploRA Plus Raman microscope using a 638 nm laser with a power of 1.5 mW. The power was adjusted using a power filter for each complex to inhibit sample decomposition.

Solid-state UV/Vis/NIR spectra were run on a powdered sample using an Edinburgh Instruments FP920 Phosphorescence Lifetime Spectrometer equipped with a 450 W steady state Xenon lamp (with single 300 mm focal length excitation and emission monochromators in Czerny Turner configuration), a red sensitive photomultiplier in Peltier housing (Hamamatsu R928P), and fitted with an integrating sphere. The instrument was set to measure the spectral reflectance of the sample and a $BaSO_4$ blank; the software was then used to calculate the absorbance spectrum from this data.

Solution UV/Vis/NIR spectra were recorded on a Perkin Elmer LAMBDA 750 or 1050 spectrometer. Data were collected in a 1 mm path-length cuvette loaded in an MBraun glovebox and were run versus the appropriate solvent.

Variable-temperature magnetic moment data were recorded with various applied direct current (DC) external fields on a Quantum Design MPMS3 superconducting quantum interference device

magnetometer using doubly recrystallised powdered samples. Measurements were performed in dc scan mode using 40 mm scan length and 6 s scan time. Samples were carefully checked for purity and data reproducibility between several independently prepared batches for each compound examined where great care was taken to ensure accurate weighing of materials. Samples were crushed (mortar and pestle) under an argon atmosphere and immobilised in an eicosane matrix in a borosilicate glass NMR tube to prevent sample reorientation during measurements. The tube was flame-sealed under dynamic vacuum (1×10^{-3} mbar) to a length of approximately 3 cm and mounted in the centre of a drinking straw, with the straw fixed to the end of an MPMS3 sample rod. Care was taken to ensure complete thermalisation of samples before each data point was measured by employing delays at each temperature point as well as a slow cooling rate (2.5 K/min from 300 to 100 K; 1 K/min from 100 to 55 K; 0.5 K/min from 55 to 1.8 K). For isothermal magnetisation measurements, long delays were employed at each temperature point to account for slow thermal equilibration of the sample. Diamagnetic corrections were applied using tabulated Pascal constants or the magnetic susceptibility was corrected for the intrinsic diamagnetism of the sample estimated as the molecular weight (g mol^{-1}) multiplied by -0.4 to -0.6×10^{-6} cm^3 mol^{-1}. Measurements were corrected for the blank sample holders (flame sealed Wilmad NMR tube and straw) and eicosane matrix. Variable temperature magnetic susceptibility and isothermal magnetisation measurements were conducted on calibrated blanks (sealed NMR tubes containing varying eicosane masses) to determine eicosane contributions, which is defined per mg of eicosane used, in addition to the contribution from the NMR tube/straw, which is kept approximately constant from sample to sample.

CHN microanalyses of complexes **6a/6b**, **9**, and **12** were carried out by Martin Jennings and Anne Davies at the University of Manchester. CHN microanalyses of complexes **13**, **14**, **15**, and **16** were carried out by Hai-Jun Li at Zhengzhou University.

Crystallographic Alerts

Validation Reply form for **6a6b-2**

_vrf_PLAT342_6a6b-2

PROBLEM: Low Bond Precision on C-C Bonds .... 0.02086 Ang.

RESPONSE: Low precisions is due to disorder.

_vrf_PLAT971_6a6b-2

PROBLEM: Check Calcd Resid. Dens. 1.55Ang From Bi2 4.93 eA-3

RESPONSE: Residual density is close to a heavy atom, and is present despite applying the correct absorption correction. It is not chemically significant

_vrf_PLAT972_6a6b-2

PROBLEM: Check Calcd Resid. Dens. 2.26Ang From C4 -4.85 eA-3

RESPONSE: Residual density is close to a heavy atom, and is present despite applying the correct absorption correction. It is not chemically significant

Validation Reply form for **6a6b-3**

_vrf_PLAT972_6a6b-3

PROBLEM: Check Calcd Resid. Dens. 1.88Ang From N4 -5.62 eA-3

RESPONSE: Residual density is likely part of a minor disordered component in the cryptand unit. Attempts to model this were unsuccessful. It is not chemically significant.

_vrf_PLAT975_6a6b-3

PROBLEM: Check Calcd Resid. Dens. 1.06Ang From N4 . 2.12 eA-3

RESPONSE: Residual density is likely part of a minor disordered component in the cryptand unit. Attempts to model this were unsuccessful. It is not chemically significant.

Validation Reply form for **6a6b-4**

_vrf_PLAT342_6a6b-4

PROBLEM: Low Bond Precision on C-C Bonds .... 0.03 Ang.

RESPONSE: Low precisions is due to disorder.

_vrf_PLAT971_6a6b-4

PROBLEM: Check Calcd Resid. Dens. 1.72Ang From Bi1 7.10 eA-3

RESPONSE: Residual density is close to a heavy atom, and is present despite applying the correct absorption correction. It is not chemically significant

_vrf_PLAT972_6a6b-4

PROBLEM: Check Calcd Resid. Dens. 2.29Ang From C4 -7.72 eA-3

RESPONSE: Residual density is close to a heavy atom, and is present despite applying the correct absorption correction. It is not chemically significant

_vrf_PLAT976_6a6b-4

PROBLEM: Check Calcd Resid. Dens. 0.82Ang From N4 . -2.14 eA-3

RESPONSE: Residual density is likely part of a minor disordered component in the cryptand unit. Attempts to model this were unsuccessful. It is not chemically significant.

Validation Reply form for **8**

_vrf_PLAT342_8

PROBLEM: Low Bond Precision on C-C Bonds .... 0.04762 Ang.

RESPONSE: Low precision is due to twinned nature of the

crystal.

Validation Reply form for **9**

_vrf_PLAT420_9

PROBLEM: D-H Bond Without Acceptor O1 --H1A . Please Check

RESPONSE: The Oxygen is sat in the apical pocket of the Tren-U unit and there is no formal acceptor.

Validation Reply form for **10**

_vrf_PLAT971_10

PROBLEM: Check Calcd Residual Density 0.83A From U1 4.60 eA-3

RESPONSE: Residual density is close to a heavy atom, and is present despite applying the correct absorption correction. It is not chemically significant

_vrf_PLAT972_10

PROBLEM: Check Calcd Residual Density 0.83A From U1 -5.34 eA-3

RESPONSE: Residual density is close to a heavy atom, and is present despite applying the correct absorption correction. It is not chemically significant

Validation Reply form for **12**

_vrf_PLAT910_12

PROBLEM: Missing # of FCF Reflection(s) Below Theta(Min). 46 Note

RESPONSE: Missing reflections are likely due to obfuscation by the beamstop

_vrf_PLAT971_12

PROBLEM: Check Calcd Resid. Dens. 0.94Ang From U3 8.41 eA-3

RESPONSE: Residual density is close to a heavy atom and present despite applying absorption corrections. It is not chemically significant.

_vrf_PLAT972_12

PROBLEM: Check Calcd Resid. Dens. 0.82Ang From U4 -4.74 eA-3

RESPONSE: Residual density is close to a heavy atom and present despite applying absorption corrections. It is not chemically significant.

_vrf_PLAT973_12

PROBLEM: Check Calcd Positive Resid. Density on U4 4.11 eA-3

RESPONSE: Residual density is close to a heavy atom and present despite applying absorption corrections. It is not chemically significant.

Validation Reply form for **14**

_vrf_PLAT342_14

PROBLEM: Low Bond Precision on C-C Bonds .... 0.02972 Ang.

RESPONSE: Low precision is due to low certainty on atom positions due to a pseudo C centred supercell. A Twin-law to account for this could not be found so is not accounted for in the final refinements.

Validation Reply form for **15**

None

Validation Reply form for **16**

_vrf_PLAT971_16

PROBLEM: Check Calcd Resid. Dens. 0.70Ang From Th2 2.52 eA-3

RESPONSE: Residual density is close to a heavy atom and present despite applying absorption corrections. It is not chemically significant.

_vrf_PLAT972_16

PROBLEM: Check Calcd Resid. Dens. 0.85Ang From Th1 -3.23 eA-3

RESPONSE: Residual density is close to a heavy atom and present despite applying absorption corrections. It is not chemically significant.

Modified Synthesis of cis,cis-1,3,5-tris-[{(benzyloxy)carbonyl}aminocyclohexane

A 500 mL round-bottomed Schlenk flask was charged with *cis*,*cis*-1,3,5-cyclohexane tricarboxylic acid (10.00 g, 46.26 mmol). To this, a solution of diphenyl phophorylazide (30 mL, 139.20 mmol) and triethylamine (20 mL, 144.28 mmol) in benzene (250 mL) was added with stirring at room temperature. The mixture was stirred for 30 minutes before being heated to reflux resulting in the

gradual effervescence of the reaction mixture. Once gas evolution had ceased (approximately 30 minutes), benzyl alcohol (16 mL, 153.89 mmol) was added, and the reaction solution was refluxed for 18 hours. The resultant white suspension was allowed to cool to room temperature before being filtered and the residue washed with benzene (3 × 25 mL). Removal of volatiles *in vacuo* afforded *cis*,*cis*-1,3,5-*tris*-[{(benzyloxy)carbonyl}aminocyclohexane as a free-flowing white powder. Yield: 14.77 g, 60%. ^{1}H NMR (D_6-DMSO, 298 K) δ 7.35 (s, 15H, *Ph*-$COOCH_2$Ph), 5.01 (s, 6H, C*H*$_2$-$COOCH_2$Ph), 3.41-3.38 (m, 3H, C*H*-Cy), 1.90 (d, J = 11.0 Hz, 3H, C*H*$_2$-Cy), 1.12-1.03 (m, 3H, C*H*$_2$-Cy).

Modified Synthesis of cis,cis-1,3,5-triamino-cyclohexane•3HBr

Solid *cis*,*cis*-1,3,5-*tris*-[{(benzyloxy)carbonyl}aminocyclohexane (40.00 g, 75.17 mmol) was added in a portion-wise manner to a stirring solution of HBr/glacial acetic acid mixture (33%, 250 mL, 345.83 g). The mixture was stirred for 4 hours before EtOH (250 mL) was added and the mixture further stirred at room temperature for 30 minutes. The resultant white suspension was filtered and the residue washed with Et_2O (3 × 25 mL). Removal of volatiles *in vacuo* afforded *cis*,*cis*-1,3,5-triamino-cyclohexane•3HBr as a white powder. Yield: 26.00 g, 93%. ^{1}H NMR (D_6-DMSO, 298 K): δ 8.23 (d, J = 2.9 Hz, 9H, NH_3Br-Cy), 3.32 (s, 3H, CH-Cy), 2.29 (d, J = 11.4 Hz, 3H, C*H*$_2$-Cy), 1.54-1.38 (m, 3H, C*H*$_2$-Cy).

Modified Synthesis of cis,cis-1,3,5-triamino-cyclohexane

Under a dinitrogen or argon atmosphere, solid NaOMe (17.30 g, 320.23 mmol) was added in a portion-wise manner to a stirring solution of *cis*,*cis*-1,3,5-triamino-cyclohexane•3HBr (4.00 g, 10.75 mmol) in THF (500 mL). The resultant slurry was refluxed for 18 hours with stirring to obtain a suspension which was allowed to cool to room temperature before being filtered to obtain a pale-yellow filtrate. Removal of volatiles *in vacuo* afforded *cis*,*cis*-1,3,5-triamino-cyclohexane as an off-

white crystalline solid. Yield: 1.18 g, 85%. ^{1}H NMR ($CDCl_3$, 298 K) δ 2.72 (s, 3H, CH-Cy), 1.96 (d, *J* = 11.3 Hz, 3H, CH_2-Cy), 0.84 (d, *J* = 11.8 Hz, 3H, CH_2-Cy).

Modified Synthesis of AgBPh$_4$

A 250 mL round-bottomed flask was charged with silver nitrate (1.189 g, 7.00 mmol) and sodium tetraphenylborate (3.40 g, 7.00 mmol). In the absence of light, acetone (150 mL) was added and the reaction stirred for 3 hours. The resultant white suspension was then filtered and the residue washed with H_2O (100 mL), EtOH (2 × 50 mL) and Et_2O (2 × 50 mL). Removal of volatiles *in vacuo* and further drying under dynamic vacuum (1 × 10^{-3} mbar) for 24 hours afforded $AgBPh_4$ as a free-flowing white powder, which was used without further purification and stored in the absence of light. Yield: 2.72 g, 91%. ^{1}H (D_6-DMSO, 298 K): δ 7.18 (br s, 8H, *meta*-Ar*H*), 6.93 (s, 8H, *ortho*-Ar*H)*, 6.79 (m, 4H, *para*-Ar*H*) ppm. ^{13}C{^{1}H} (D_6-DMSO, 298 K): δ 135.54 (Ar*C*), 125.35 (Ar*C*), 121.56 (Ar*C*) ppm. ^{11}B{^{1}H} (D_6-DMSO, 298 K): δ –7.66 ppm. ATR-IR ν/cm^{-1}: 3046 (m), 2987 (w), 1705 (w), 1573 (m), 1558 (w), 1474 (s), 1422 (s), 1258 (m), 1174 (w), 1151 (w), 1138 (w), 1123 (w), 1064 (m), 1028 (m), 995 (m), 977 (m), 920 (m), 868 (s), 849 (m), 737 (s), 729 (s), 716 (s), 610 (s), 534 (w), 488 (m), 470 (m), 420 (w) cm^{-1}.

Modified Synthesis of [CPh$_3$][BPh$_4$]

Under a dinitrogen or argon atmosphere, solid silver triflate (0.72 g, 2.79 mmol) was added in a portion-wise manner to a stirring solution of trityl chloride (0.78 g, 2.78 mmol) in DCM (10 mL), resulting in the immediate precipitation of AgCl and a colour change to yellow. The suspension was stirred for 30 minutes at room temperature, before being filtered and soluble residues extracted from the precipitate with DCM (2 × 5 mL). The resultant solution was concentrated to approximately 5 mL, before hexane (15 mL) was added with stirring resulting in the precipitation of the product and the solution collected by filtration. This process is repeated until the filtrate is very pale yellow in colour, with no visible brown impurities (usually 3-4 times). Removal of volatiles from the solid *in*

vacuo and further drying under dynamic vacuum (1 × 10^{-3} mbar) afforded $[CPh_3][OS(O)_2CF_3]$ as a deep yellow microcrystalline solid, which was used immediately without further purification. A Schlenk flask then was charged with $[CPh_3][OS(O)_2CF_3]$ (0.98 g, 2.50 mmol) and $NaBPh_4$ (0.86 g, 2.50 mmol) and the solid mixture cooled to 0 °C. In the strict absence of light, acetonitrile (10 mL) was added resulting in the formation of a deep red solution which was stirred for one hour. Stirring was then stopped and the solution allowed to stand for 30-60 minutes resulting in the formation of red needle crystals which were isolated by filtration. Drying of the crystals under dynamic vacuum (1 × 10^{-3} mbar) for 2 hours afforded $[CPh_3][BPh_4]$ as an air-sensitive red crystalline powder which was stored at –30 °C in the strict absence of light. **Note** – this reaction does not work when DCM is used as the reaction solvent. Yield: 1.29 g, 92%. ^{1}H (D_3-MeCN, 298 K): δ 7.76 (s), 7.63 (s), 7.44 (s), 7.36 (s), 7.26 (br, s), 7.12 (s), 6.84 (s), 4.24 (d) ppm. $^{13}C\{^{1}H\}$ (D_3-MeCN, 298 K): δ 136.71, 135.71, 133.97, 129.30, 128.79, 127.96, 126.60, 126.20 ppm. $^{11}B\{^{1}H\}$ (D_3-MeCN, 298 K): δ –1.29 (br), –7.58 ppm. In solution, $[CPh_3][BPh_4]$ exists in equilibrium with $(Ph_3C)(H)C_6H_4(BPh_3)$ resulting in many overlapping peaks in the aryl regions of the ^{1}H (8–6 ppm) and $^{13}C\{^{1}H\}$ (140–125 ppm) spectra inhibiting accurate assignment and integration. In addition, this equilibrium results in two resonances in the $^{11}B\{^{1}H\}$ spectrum.[4] ATR-IR ν/cm^{-1}: 3053 (w), 1619 (w), 1578 (s), 1480 (s), 1447 (s), 1426 (w), 1354 (s), 1292 (m), 1271 (m), 1241 (m), 1230 (w), 1183 (s), 1031 (s), 994 (m), 980 (m), 950 (w), 914 (m), 843 (s), 805 (m), 768 (m), 733 (s), 700 (s), 646 (s), 623 (m), 609 (s), 583 (m), 522 (s), 488 (w), 468 (s), 426 (w) cm^{-1}.

Modified Synthesis of $[HNEt_3][BPh_4]$

To a vigorously stirring solution of sodium tetraphenylborate (5.13 g, 15.00 mmol) in H_2O (50 mL) was added dropwise a solution of triethylamine hydrochloride (2.47 g, 18.00 mmol) in H_2O (15 mL), and the reaction stirred for 16 hours. The resultant white suspension was filtered and the residue washed with H_2O (3 × 10 mL). Removal of volatiles *in vacuo* and further drying at 60 °C afforded $[HNEt_3][BPh_4]$ as a free-flowing white powder, which was used without further purification. Yield:

6.89 g, 90%. ^{1}H (D_6-DMSO, 298 K): δ 8.80 (s, 1H, *H*NEt_3), 7.13 (br s, 8H, *meta*-Ar*H*), 6.88 (t, $^{3}J_{HH}$ = 7.3 Hz, 8H, *ortho*-Ar*H)*, 6.75 (m, 4H, *para*-Ar*H*), 3.05 (m, 6H, HN(C$H_2$$CH_3$)$_3$), 1.12 (t, $^{3}J_{HH}$ = 7.3 Hz, 9H, HN(CH_2CH_3)$_3$ ppm. ^{13}C{^{1}H} (D_6-DMSO, 298 K): δ 136.05 (Ar*C*), 125.89 (Ar*C*), 122.06 (Ar*C*), 46.25 (HN(*C*$H_2$$CH_3$)$_3$), 9.18 (HN($CH_2$*C*$H_3$)$_3$) ppm. ^{11}B{^{1}H} ($D_6$-DMSO, 298 K): δ −7.81 ppm. ATR-IR ν/cm^{-1}: 3128 (m), 3051 (m), 3000 (w), 2986 (w), 1580 (w), 1477 (m), 1426 (m), 1385 (m), 1292 (w), 1268 (w), 1179 (w), 1148 (m), 1064 (m), 1031 (m), 1013 (m), 863 (w), 838 (m), 807 (w), 772 (m), 739 (s), 702 (s), 604 (s), 476 (m), 462 (m), 415 (w) cm^{-1}.

Modified Synthesis of TlBPh$_4$

To a vigorously stirring solution of sodium tetraphenylborate (1.71 g, 5.00 mmol) in H_2O (50 mL) was added a solution of thallium nitrate (1.47 g, 5.50 mmol) in H_2O (50 mL), and the reaction stirred for 16 hours. The resultant white suspension was filtered and the residue washed with hot H_2O (3 × 10 mL) and hexane (2 × 20 mL). Removal of volatiles *in vacuo* and further drying under dynamic vacuum (1 × 10^{-3} mbar) for 24 hours afforded $TlBPh_4$ as a free-flowing off-white powder, which was used without further purification. Yield: 2.36 g, 90%. ^{1}H (D_6-DMSO, 298 K): δ 7.18 (br s, 8H, *meta*-Ar*H*), 6.91 (s, 8H, *ortho*-Ar*H)*, 6.77 (m, 4H, *para*-Ar*H*) ppm. ^{13}C{^{1}H} (D_6-DMSO, 298 K): δ 136.07 (Ar*C*), 125.87 (Ar*C*), 122.07 (Ar*C*) ppm. ^{11}B{^{1}H} (D_6-DMSO, 298 K): δ –7.67 ppm. ATR-IR ν/cm^{-1}: 3056 (w), 3034 (w), 3003 (w), 2983 (w), 2967 (w), 1577 (m), 1479 (s), 1426 (s), 1308 (w), 1270 (m), 1182 (s), 1155 (s), 1130 (w), 1067 (w), 1028 (w), 988 (m), 918 (m), 865 (w), 852 (s), 738 (s), 712 (s), 625 (m), 615 (w), 599 (s), 484 (s), 458 (s), 435 (w), 425 (w), 407 (w) cm^{-1}.

Modified procedure for the preparation of KC$_8$

A 250 mL round-bottomed Schlenk flask was charged with reagent grade (> 99.9%) graphite (3.55 g, 818.4 mmol) and dried under dynamic vacuum (1 × 10^{-3} mbar) at 100 °C for four hours. In an argon-filled glovebox, freshly cut potassium metal (1.45 g, 102.3 mmol) is added. The mixture is then heated under an argon atmosphere with a blowtorch whilst agitating causing the potassium metal to

melt and intercalation to occur. Continue heating until the mixture has completely changed colour from black to bronze, which will be for approximately 3 hours. Once the reaction is complete, allow to cool to room temperature. Yield: 5.0 g, 99%.

Modified synthesis of KCH_2Ph

To a stirring suspension of $KOBu^t$ (5.60 g, 50.00 mmol) in toluene (100 mL) was added Bu^nLi (20 mL, 2.5 M in hexane, 50.00 mmol) dropwise at 0 °C. The mixture was warmed to room temperature and stirred for an additional 30 minutes. The orange/red suspension was filtered, and the resultant red solid washed with toluene (2 × 50 mL) and hexane (20 mL). Removal of the volatiles *in vacuo* afforded KCH_2Ph as a free-flowing orange powder, which was used without further purification. Yield: 6.40 g, 98%.

Modified preparation of $Tren^{TIPS}H_3$

$N(CH_2CH_2NH_2)_3$ (10 mL, 66.60 mmol) was dissolved in THF (100 mL). Bu^nLi (2.5 M, 80 mL, 200.00 mmol) was added dropwise at –78 °C, warmed to room temperature and the mixture stirred for 6 hours. The solution was then cooled to –78 °C, and $ClSiPr^i_3$ (42.80 mL, 200.00 mmol) was added in a portion-wise manner and the solution stirred at room temperature for 16 hours. Removal of volatiles *in vacuo* resulted in a pale-yellow sticky solid. The product was extracted with hexane (2 × 50 mL), and the solution was filtered through a Celite® padded coarse-porosity frit, affording a colourless filtrate. Removal of volatiles *in vacuo* resulted a colourless oil which was used without further purification. Yield = 38.86 g (90%). ^1H NMR (D_6-benzene, 298 K): δ 2.88 (6H, q, $^3J_{HH}$ = 6.6 Hz, CH_2), 2.43 (6H, t, $^3J_{HH}$ = 6.3 Hz, CH_2), 1.15–0.95 (63H, m, Pr^i-CH_3 and Pr^i-CH), 0.76 (3H, t, $^3J_{HH}$ = 7.3 Hz, -H) ppm. $^{29}Si\{^1H\}$ NMR (D_6-benzene, 298 K): δ 4.89 ppm.

Modified Synthesis of Tren^{TIPS}Li$_3$

$N(CH_2CH_2NH_2)_3$ (10 mL, 66.60 mmol) was dissolved in THF (100 mL). Bu^nLi (2.5 M, 80 mL, 200.00 mmol) was added dropwise at –78 °C, warmed to room temperature and the mixture stirred for 6 hours. The solution was then cooled to –78 °C, and $ClSiPr^i_3$ (42.80 mL, 200.00 mmol) was added in a portion-wise manner and the solution stirred at room temperature for 16 hours. Removal of volatiles *in vacuo* resulted in a pale-yellow sticky solid. The product was extracted with hexane (2 × 50 mL), and the solution was filtered from the LiCl precipitate. Bu^nLi (2.5 M, 80 mL, 200.00 mmol) was added dropwise at –78 °C, warmed to room temperature and the solution was stirred for 6 hours at room temperature. Removal of volatiles *in vacuo* resulted in an off-white solid which was washed with cold hexane (2 × 10 mL) to yield $Tren^{TIPS}Li_3$ as a white powder. Colourless crystals of $Tren^{TIPS}Li_3$ were grown from a concentrated solution in hexane stored at –30 °C. Yield: 34.25 g, 81%. ^1H NMR (D_6-benzene, 298 K): δ 3.22 (t, 6H, NCH_2CH_2), 2.38 (t, 6H, NCH_2CH_2), 1.29 (m, 9H, $CH(CH_3)_2$), 1.28 (m, 54H, $CH(CH_3)_2$) ppm. ATR-IR ν/cm^{-1}: 2936 (s), 2883 (m), 2854 (s), 2770 (w), 2661 (w), 1461 (s), 1362 (w), 1342 (w), 1270 (w), 1237 (w), 1138 (w), 1056 (s), 1027 (s), 1004 (w), 992 (w), 933 (s), 877 (s), 776 (s), 657 (s), 637 (s), 567 (m), 513 (w), 493 (s), 462 (w), 448 (w), 419 (m) cm^{-1}.

Modified procedure for the preparation of U^{IV}Cl$_4$

A 1000 mL round-bottomed flask was charged with UO_3 (23.54 g, 82.22 mmol) and hexachloropropene (250 mL). The flask was equipped with two condensers stacked on top of one another, and the flask placed under an inert gas supply. The mixture was heated carefully to reflux, which was accompanied by a violent exotherm and the liberation of a dark brown gas. The flask was lifted away from the heating mantle to allow the exotherm to subside before heating was resumed. **Note**: this moderation of the exotherm step may be needed to be conducted multiple times. The reaction mixture was then left to gently reflux for 16 hours. During which time, UCl_4 precipitates from solution as a green solid. The mixture was cooled to room temperature, and the reaction mixture

carefully filtered away from the green solid before washing with DCM (3 × 150 mL). Removal of volatiles *in vacuo* afforded UCl_4 as a free-flowing green powder, which was used without further purification. Yield: 28.01 g, 90%.

Modified synthesis of [U^{IV}(Tren^{TIPS})Cl]

A solution of Tren^{TIPS}Li$_3$ (12.66 g, 20.00 mmol) in THF (50 mL) was added dropwise to a stirring solution of UCl_4 (7.60 g, 20.00 mmol) in THF (80 mL) at –78 °C. The mixture was allowed to warm to room temperature before stirring for 16 hours. Removal of volatiles *in vacuo* resulted in a brown solid. The product was extracted in hot toluene (100 mL) and the solution was filtered from the LiCl precipitate. Removal of volatiles *in vacuo* resulted in a pale-brown solid. The product was washed with hexane (2 × 10 mL) to yield [U^{IV}(Tren^{TIPS})Cl] as a brown solid. Green crystals of [U^{IV}(Tren^{TIPS})Cl] were grown from a concentrated solution in toluene at room temperature. Yield: 10.75 g, 61%. ^{1}H NMR (D$_6$-benzene, 298 K): δ 9.20 (s, 54H, CH(C*H*$_3$)$_2$), 8.42 (s, 9H, C*H*(CH$_3$)$_2$), 5.72 (s, 6H, NCH$_2$C*H*$_2$), –36.20 (s, 6H, NC*H*$_2$CH$_2$) ppm. ATR-IR ν/cm^{-1}: 2938 (m), 2922 (m), 2860 (s), 1463 (s), 1383 (w), 1360 (w), 1339 (w), 1272 (w), 1132 (w), 1039 (m), 1009 (m), 988 (w), 918 (s), 879 (s), 817 (w), 723 (s), 672 (s), 626 (s), 548 (m), 515 (m), 448 (m).

Modified Synthesis of [U{N(CH$_2$CH$_2$NSiPr$^i_3$)$_2$(CH$_2$CH$_2$SiPr$^i_2$CHMeCH$_2$)}].

A Schlenk flask was charged with [U^{IV}(Tren^{TIPS})Cl] (9.00 g, 10.00 mmol) and KCH$_2$Ph (1.30 g, 10.00 mmol). At –78 °C, toluene (100 mL) was added with stirring before the mixture was warmed to room temperature and stirred for a further 16 hours to afford an orange-brown mixture. Removal of volatiles *in vacuo* resulted in a sticky red solid. The product was extracted in hexane (3 x 50 mL), filtered and volatiles removed *in vacuo* before being washed with cold pentane (2 × 10 mL) to yield **2U** as a red solid, which was further dried *in vacuo*. Orange crystals of **2U** were grown through the storage of a concentrated pentane solution at 4 °C for 72 hours. Yield: 0.55 g, 65%. ^{1}H NMR (C_6D_6, 298 K): δ 46.75 (1H, s, C*H*$_2$), 32.65 (1H, s, C*H*$_2$), 28.89 (1H, s, C*H*$_2$), 23.20 (1H, s, C*H*$_2$), 15.93 (1H, s,

UCH_2CH), 15.76 (3H, s, UCH_2CHMe), 11.73 (1H, s, CH_2), 11.30 (1H, m, $CHMe_2$), 9.54 (1H, m, $CHMe_2$), 8.38 (3H, d, $^3J_{HH}$ = 7.3 Hz, Si*Me*), 6.82 (1H, s, CH_2), 6.02 (3H, d, $^3J_{HH}$ = 7.3 Hz, Si*Me*), 5.65 (3H, d, $^3J_{HH}$ = 6.4 Hz, Si*Me*), 4.01 (9H, s, 3×Si*Me*), 3.72 (3H, d, $^3J_{HH}$ = 6.4 Hz, Si*Me*), 2.77 (1H, s, CH_2), 1.52 (3H, s, 3×$CHMe_2$), – 0.53 (1H, s, CH_2), –1.33 (2H, s, UCH_2), –2.61 (9H, 3×Si*Me*), –2.89 (9H, s, 3×Si*Me*), –4.70 (9H, s, 3×Si*Me*), –20.84 (3H, s, 3×$CHMe_2$), –23.09 (1H, s, CH_2), –24.14 (1H, s, CH_2), –31.64 (1H, s, CH_2), –35.20 (1H, s, CH_2) ppm. ATR-IR ν/cm^{-1}: 2938 (s), 2885 (m), 2858 (s), 2778 (m), 2721 (w), 2665 (w), 1236 (s), 1379 (w), 1362 (w), 1346 (w), 1261 (m), 1136 (w), 1089 (m), 1068 (s), 1009 (m), 906 (s), 879 (s), 762 (s), 737 (s), 669 (s), 628 (s), 587 (s), 550 (m), 513(m), 474 (w), 406 (m) cm^{-1}.

Modified Synthesis of [U^{IV}(Tren^{TIPS})(THF)][BPh$_4$]

To a THF (50 mL) solution of $[U\{N(CH_2CH_2NSiPr^i_3)_2(CH_2CH_2SiPr^i_2CHMeCH_2)\}]$ was added $[HNEt_3][BPh_4]$ (3.33 g, 7.9 mmol) in a portion-wise manner at −78 °C. The resultant orange slurry was warmed to room temperature and stirred for a further 16 hours to afford a yellow-green solution. Removal of the volatiles *in vacuo* afforded a yellow-green oil to which hexane (50 ml) was added. The mixture was heated briefly to 60 °C and allowed to cool slowly to room temperature whilst stirring vigorously. Trituration was complete inside 2 hours affording a pale green solid which was isolated by filtration, washed with hexane (3 × 5 mL) and dried *in vacuo*. Green crystals of $[U^{IV}(Tren^{TIPS})(THF)][BPh_4]$ were grown through the storage of a concentrated toluene solution at −30 °C. Yield: 9.02 g, 92%. ^{1}H NMR (D_6-benzene, 298 K): δ 28.32 (s, 6H, CH_2), 7.03 (s, 4H, Ar-H), 5.80 (s, 9H, $CH(CH_3)_2$), 4.08 (s, 4H, THF), 3.06 (s, 54H, $CH(CH_3)_2$), 2.46 (s, 4H, THF), 2.12 (s, 8H, Ar-*H*), 0.82 (s, 8H, Ar-*H*), −49.51 (s, 6H, CH_2) ppm. ATR-IR ν/cm^{-1}: 3058 (w), 3035 (w), 2938 (m), 2862 (m), 2731 (m), 1580 (w), 1463 (m), 1424 (w), 1387 (w), 1366 (w), 1288 (w), 1257 (m), 1128 (m), 1064 (s), 1031 (m), 1007 (m), 924 (m), 891 (s), 842 (w), 762 (s), 733 (s), 704 (s), 672 (s), 639 (s), 612 (s), 585 (s), 511 (w), 470 (m), 419 (m) cm^{-1}.

Modified Synthesis of [U^{III}(Tren^{TIPS})] (1)

A suspension of [U^{IV}(Tren^{TIPS})Cl] (4.42 g, 5.00 mmol) in hexane (20 mL) was transferred into a Schlenk flask containing a freshly prepared potassium mirror (20-fold excess) and a glass-coated stirrer bar. The mixture was stirred vigorously for seven days, after which the resultant dark purple solution was filtered into another Schlenk flask containing a freshly prepared potassium mirror (20-fold excess) and a glass-coated stirrer bar. The mixture was stirred vigorously for a further five days. Filtration of the deep purple solution and removal of volatiles *in vacuo* yielded **1** as a dark purple powder. Dark purple crystals of **1** were grown through the storage of a concentrated hexane solution at −30 °C. Yield: 1.33 g, 78%. ^{1}H NMR (D_6-benzene, 298 K): δ 17.67 (s, 6H, CH_2), 7.22 (s, 9H, CH(CH_3)$_2$), 4.03 (s, 54H, CH(CH_3)$_2$), –40.07 (s, 6H, CH_2) ppm. ATR-IR ν/cm^{-1}: 2936 (s), 2885 (m), 2858 (s), 2751 (m), 2661 (w), 1459 (s), 1385 (w), 1362 (w), 1344 (w), 1257 (m), 1134 (w), 1093 (s), 1070 (s), 1035 (w), 1000 (m), 926 (s), 912 (s), 877 (s), 772 (s), 750 (s), 735 (s), 663 (s), 628 (s), 589 (m), 567 (m), 544 (m), 509 (m), 497 (m), 417 (m) cm^{-1}.

Modified Synthesis of [K(2.2.2-cryptand)]$_2$[Pb_2Bi_2] (2)

A Schlenk flask was charged with solid "KPbBi" (1.00 g, 2.2 mmol) and 2.2.2-cryptand (0.87 g, 2.31 mmol, 1.05 eq.). In the strict absence of light and at room temperature, ethane-1,2-diamine (100 mL) was added and the resultant dark-brown suspension stirred for 4 hours. Filtration of the suspension and removal of volatiles *in vacuo* yielded **2** as a dark brown crystalline powder. Analysis by PXRD confirmed the identity of **2** as [K(2.2.2-cryptand)]$_2$[Pb_2Bi_2]. Yield: 1.04 g, 62%.

Modified Synthesis of [K(2.2.2-cryptand)]$_2$[$GaBi_3$] (3)

A Schlenk flask was charged with solid $K_5Ga_2Bi_4$ (0.5 g, 0.43 mmol) and 2.2.2-cryptand (0.402 g, 1.07 mmol, 2.5 eq.). In the strict absence of light and at room temperature, ethane-1,2-diamine (12.5 mL) was added and the resultant turquoise suspension stirred for 2 hours. After which, the suspension was filtered and the filtrate layered with toluene (37.5 mL) and hexane (25 mL). After storage for

7 days at room temperature, the deeply coloured solution was filtered away and the remaining residue washed with toluene (2 × 5 mL). Removal of volatiles *in vacuo* afforded a crystalline material which was analysed using PXRD. The PXRD pattern always shows a mixture of $[K(crypt\text{-}222)]_2Bi_2$ and $[K(crypt\text{-}222)]_2Bi_4$, however, this material has repeatedly resulted in gallium containing species upon reaction.[25,26] The assignment of **3** as $[K(2.2.2\text{-cryptand})]_2[GaBi_3]$ has been made based on the identification of known indium, **4**, and thalium, **5**, analogues. Yield: 0.20 g, 34%.

Modified Synthesis of $[K(2.2.2\text{-cryptand})]_2[InBi_3]$ (4)

A Schlenk flask was charged with solid "$K_5In_2Bi_4$" (1.20 g, 0.952 mmol) and 2.2.2-cryptand (1.81 g, 4.81 mmol, 5.05 eq.). In the strict absence of light and at room temperature, ethane-1,2-diamine (120 mL) was added, and the resultant suspension stirred for 18 hours before being sonicated for a further 4 hours. All remaining solid residues were allowed to settle for several hours, before the supernatant solution was filtered. Concentration of the filtrate *in vacuo* and storage at 5 °C for 5 days afforded crystals of **4**, which were isolated by filtration. Removal of volatiles *in vacuo* and analysis by PXRD confirmed the identity of **4** as $[K(2.2.2\text{-cryptand})]_2[InBi_3]$. Yield: 1.305 g, 93%.

Modified Synthesis of $[K(2.2.2\text{-cryptand})]_2[TlBi_3]$ (5)

A Schlenk flask was charged with solid K_2TlBi_3 (1.00 g, 1.1 mmol) and 2.2.2-cryptand (0.85 mg, 2.25 mmol, 2.05 eq.). In the strict absence of light and at room temperature, ethane-1,2-diamine (100 mL) was added, and the resultant olive-green suspension stirred for 3 days. After which, in the strict absence of light, the suspension was filtered. Concentration of the filtrate *in vacuo* to approximately 10 mL and storage at 5°C for two days afforded dark brown crystals of **5**, which were isolated by filtration. Removal of volatiles *in vacuo* and analysis by PXRD confirmed the identity of **5** as $[K(2.2.2\text{-cryptand})]_2[TlBi_3]$. Yield: 1.39 g, 80%. There is no reference data for this compound however, the PXRD spectrum is consistent with multiple different extractions and has led to the same reactivity.

Modified Synthesis of [K(2.2.2-cryptand)]$_2$[Bi$_2$] (7)

A Schlenk flask was charged with solid **11** (0.52 g, 0.50 mmol) and 2.2.2-cryptand (0.75 g, 2.00 mmol, 4.0 eq.). In the strict absence of light and at room temperature, ethane-1,2-diamine (12.5 mL) was added, and the resultant turquoise suspension stirred for 15 minutes. After which, the suspension was filtered and the filtrate layered with methyl-*tert*-butylether (20 mL). After storage for 7 days at room temperature, the deeply coloured solution was filtered away and the remaining residue washed with toluene (2 × 15 mL). Removal of volatiles *in vacuo* afforded a red crystalline material and analysis by PXRD confirmed the identity of **7** as [K(2.2.2-cryptand)]$_2$[Bi$_2$]. Yield: 0.90 g, 72%.

Modified Synthesis of K_5Bi_4 (11).

A niobium tube was charged with bismuth metal (4.05 g, 19.4 mmol) and potassium (0.95 g, 24.3 mmol, 1.25 eq.) The niobium tube was then sealed by arc welding before being placed into a quartz tube and sealed under vacuum. The sealed tube was then placed into an oven and tempered at 700 °C for 7 days with a heating rate of 50 K/h and a cooling rate of 25 K/h. A brittle dark grey powder of **11** was obtained and analysis by PXRD confirmed the identity of **11** as K_5Bi_4, with a residual impurity of K_3Bi_2. Yield: 4.50 g, 90%.

Synthesis and isolation of [K(2.2.2-cryptand)][{U^{IV}(Tren^{TIPS})}$_2$(μ-η^3:η^3-Bi$_3$)] (6a) with co-crystallised sub-component [K(2.2.2-cryptand)][{U^{IV}(Tren^{TIPS})}$_2$(μ-Bi)] (6b)

Representative procedure: Benzene (40 mL) was added to the solid mixture of **1** (0.34 g, 0.4 mmol) and **4** (0.32 g, 0.2 mmol) in a 20 mL glass scintillation vial at room temperature. The mixture was stirred for two days with the exclusion of light, during which time it slowly turned into a dark red suspension with an oily precipitate. Celite® was added into the reaction, and then the mixture was heated up to 80 °C and filtered through a glass microfibre disc packed in a glass pipette. A small crop of dark red-black crystals of **6a**/**6b** formed after standing the resulting dark red filtrate at room temperature for two days. The crystalline solid was isolated by decanting the mother liquor, washing

with pentane (2 × 1 mL), and then drying *in vacuo*. The mother liquor and washings were combined, and volatiles were removed *in vacuo* to afford a red oily residue, which was extracted with Et_2O (3 mL) and then filtered through a glass microfibre disc packed in a glass pipette to obtain a dark red solution. Storage of the red filtrate at −30 °C for two days yielded a second crop of dark red-black **6a**/**6b** that was isolated by decanting the mother liquor, washing with cold Et_2O (2 × 1 mL), and then drying *in vacuo*. Combined yield: 0.13 g, 24% (by U content). Due to very similar solubilities it is not possible to separate **6a**/**6b**, but the presence of small amount **6b** does not affect the further characterisation of **6a**. Substituting **2**, **3** or **5** for **4** also gave the same products with similar yields. Anal. Calcd for $C_{84}H_{186}N_{10}O_6KSi_6Bi_{2.8}U_2$•$(C_6H_6)_2$ (**6a**:**6b** = 9:1): C, 40.24; H, 7.25; N, 4.89%. Found: C, 40.28; H, 7.76; N, 4.65%. ^{1}H NMR (D_8-THF, 298 K): δ 5.25 (12H, s, br, CH_2), 5.09 (18H, s, br, Pr^i-CH), 3.89 (108H, s, br, Pr^i-CH_3), 3.54 (12H, s, br, CH_2-cryptand), 3.48 (12H, s, br, CH_2-cryptand), 2.51 (12H, s, br, CH_2-cryptand), –1.99 (12H, s, br, CH_2) ppm. ^{29}Si{^{1}H} NMR (D_8-THF, 298 K): δ –7.73 ppm. ATR-IR v/cm^{-1}: 2881 (br, w), 2854 (br, m), 1445 (m), 1355 (m), 1259 (w), 1104 (s), 1011 (m), 933 (s), 880 (s), 780 (s), 730 (vs), 676 (s), 617 (m), 570 (w), 514 (w), 438 (w).

The use of differing Bi reagents towards the synthesis and isolation of [K(2.2.2-cryptand)][{U^{IV}(Tren^{TIPS})}$_2$(μ-η^3:η^3-Bi$_3$)] (6a) without co-crystallised sub-component [K(2.2.2-cryptand)][{U^{IV}(Tren^{TIPS})}$_2$(μ-Bi)] (6b): synthesis of [{U^{IV}(Tren^{TIPS})}$_2$(μ-η^2:η^2-Bi$_2$)] (8) using [K(2.2.2-cryptand)]$_2$[Bi$_2$] (7) and isolation of [U^{IV}(Tren^{TIPS})(OH)] (9)

0.05 mmol per U scale: Benzene (10 mL) was added to the solid mixture of [U^{IV}(Tren^{TIPS})(THF)][BPh_4] (0.062 g, 0.05 mmol) and **7** (0.0325 g, 0.025 mmol) in a 20 mL glass scintillation vial at room temperature. The mixture was stirred for two days with the exclusion of light, during which time it slowly turned into a dark brown suspension with a black insoluble solid precipitate. Celite® was added into the reaction, and then the mixture was heated up to 80 °C and filtered through a glass microfibre disc packed in a glass pipette to obtain a dark brown solution. Volatiles were removed *in vacuo* to afford a dark brown residue, which was extracted with Et_2O (3

mL) and then filtered. Slow evaporation of the dark brown filtrate at −30 °C for two days yielded a mixture of dark orange crystals of **9** and a small number of dark brown crystals of **8** as confirmed by SC-XRD. Single crystals used for SC-XRD were immediately measured via Raman spectroscopy. NMR spectroscopic analysis of the crude reaction mixtures showed the presence of $Tren^{TIPS}H_3$, **1**, **8**, and **9**, as well as many unidentifiable paramagnetically shifted resonances. Due to the very small amount of this product (<1%), its instability, and capricious formation, **8** was not characterised beyond SC-XRD, crude NMR spectroscopy, and Raman spectroscopy. ^{1}H NMR (D_6-benzene, 298 K): δ 119.54 (s), 107.76 (12H, s, **8**, CH_2), 58.37 (s), 48.85 (s), 46.16 (s), 43.78 (s), 39.14 (s), 29.31 (6H, s, **9**, CH_2), 27.22 (s), 21.88 (s), 17.52 (6H, s, **1**, CH_2), 14.98 (s), 14.78 (s), 12.64 (12H, s, **8**, CH_2), 11.94 (6H, s, **9**, CH_2), 10.85 (d), 10.31 (s), 9.07 (s), 7.09 (9H, s, **1**, Pr^i-C*H*), 6.90 (d), 6.75 (d), 6.54 (s), 4.43 (s), 3.88 (54H, s, **1**, Pr^i-CH_3), 2.90 (6H, q, $Tren^{TIPS}H_3$, CH_2), 2.44 (6H, t, $Tren^{TIPS}H_3$, CH_2), 1.15–0.95 (63H, m, $Tren^{TIPS}H_3$, ^{i}Pr-CH_3 and Pr^i-C*H*), 0.76 (3H, t, $Tren^{TIPS}H_3$, -*H*), −3.78 (d), −4.07 (9H, s, **9**, Pr^i-C*H*), −5.19 (54H, s, **9**, Pr^i-CH_3), −5.84 (s), −8.47 (s), −8.82 (s), −12.20 (s), 19.49 (s), −20.46 (18H, s, **8**, Pr^i-C*H*), −24.05 (s), −25.31 (108H, s, br, **8**, Pr^i-CH_3), −40.25 (6H, s, **1**, CH_2), −49.12 (s) ppm. ^{1}H NMR integrations are internally consistent to the specific named molecule, but not relative to the whole mixture where they may represent a small percentage of the total constituents. ^{29}Si{^{1}H} NMR (D_6-benzene, 298 K): δ −21.85 (**8**), −96.93 (**9**) ppm. Raman of a single crystal of **8** ν/cm^{-1} (638 nm, 10% filter): 84 (s), 107 (s, Bi=Bi), 155 (w, br), 291 (w, br), 434 (w), 570 (m, br), 891 (s), 980 (w), 1080 (w), 1164 (w), 1247 (m), 1301 (w), 1394 (w), 1457 (w), 1476 (m), 2718 (w), 2765 (w), 2872 (s), 2898 (w), 2943 (s, br).

Greater than 0.05 mmol per U scale: Benzene (10 mL) was added to the solid mixture of [U^{IV}($Tren^{TIPS}$)(THF)][BPh_4] (0.124 g, 0.10 mmol) and **7** (0.065 g, 0.05 mmol) in a 20 mL glass scintillation vial at room temperature. The mixture was stirred for two days with the exclusion of light, resulting in a purple suspension. Celite® was added into the reaction, and then the mixture was heated up to 80 °C and filtered through a glass microfibre disc packed in a glass pipette to obtain a

dark purple solution. Volatiles were removed *in vacuo* to afford a dark purple solid, which was extracted with Et_2O (3 mL) and then filtered through a glass microfibre disc packed in a glass pipette. Volatiles were removed *in vacuo* to afford a dark purple solid. Subsequent NMR spectroscopic analysis of this solid showed **1** to be the major product, along with minor impurities of $Tren^{TIPS}H_3$ and **9**. ^{1}H NMR (D_6-benzene, 298 K): 29.22 (6H, s, **9**, CH_2), 17.55 (6H, s, **1**, CH_2), 11.96 (6H, s, **9**, CH_2), 7.09 (9H, s, **1**, Pr^i-C*H*), 3.92 (54H, s, **1**, Pr^i-CH_3), 2.90 (6H, q, $Tren^{TIPS}H_3$, CH_2), 2.46 (6H, t, $Tren^{TIPS}H_3$, CH_2), 1.16–0.95 (63H, m, $Tren^{TIPS}H_3$, Pr^i-CH_3 and Pr^i-C*H*), 0.77 (3H, t, $Tren^{TIPS}H_3$, -*H*), −4.04 (9H, s, **9**, Pr^i-C*H*), −5.15 (54H, s, **9**, Pr^i-CH_3), −40.23 (6H, s, **1**, CH_2) ppm. ^{1}H NMR integrations are internally consistent to the specific named molecule, but not relative to the whole mixture where they may represent a small percentage of the total constituents.

Attempted reduction of 8

Benzene (5 mL) was added to the solid mixture of $[U^{IV}(Tren^{TIPS})(THF)][BPh_4]$ (0.062 g, 0.05 mmol) and **7** (0.0325 g, 0.025 mmol) in a 20 mL glass scintillation vial at room temperature. The mixture was stirred for two days with the exclusion of light, during which time it slowly turned into a dark brown suspension with a black insoluble solid precipitate. Celite® was added into the reaction, and then the mixture was heated up to 80 °C and filtered through a glass microfibre disc packed in a glass pipette to obtain a dark brown solution. With the exclusion of light, 2.2.2-cryptand (0.0188 g, 0.05 mmol) was added with stirring followed by KC_8 (0.0067 g, 0.05 mmol) approximately 5 minutes after. The mixture was stirred for two days resulting in the formation of a pale-yellow solution with significant amounts of black insoluble solid. The mixture was filtered through a glass microfibre disc packed in a glass pipette, and volatiles were removed *in vacuo* to afford a light brown oily solid. Subsequent NMR spectroscopic analysis of this solid showed the presence of $Tren^{TIPS}H_3$ and 2.2.2-cryptand, as well as many unidentifiable resonances. ^{1}H NMR (D_6-benzene, 298 K): 7.89 (s), 7.40 (d), 6.45 (t), 4.20 (s), 3.97 (s, br), 3.65 (12H, s, CH_2-cryptand), 3.50 (12H, s, CH_2-cryptand), 3.43 (s), 3.34 (s), 3.27 (s), 2.89 (6H, q, $Tren^{TIPS}H_3$, CH_2), 2.79 (s), 2.72 (s), 2.59 (s), 2.53 (12H, s, CH_2-

cryptand), 2.45 (6H, s, $Tren^{TIPS}H_3$, CH_2), 2.26 (s), 1.42 (s), 1.33 (s), 1.15–0.95 (63H, m, $Tren^{TIPS}H_3$, Pr^i-CH_3 and Pr^i-CH), 0.79 (3H, s, $Tren^{TIPS}H_3$, -H) ppm. ^{1}H NMR integrations are internally consistent to the specific named molecule, but not relative to the whole mixture where they may represent a small percentage of the total constituents.^{29}Si{^{1}H} NMR (D_6-benzene, 298 K): δ 4.92 ppm.

Attempts to synthesise and isolate [K(2.2.2-cryptand)][{U^{IV}($Tren^{TIPS}$)}$_2$(μ-η^3:η^3-Bi_3)] (6a) without co-crystallised sub-component [K(2.2.2-cryptand)][{U^{IV}($Tren^{TIPS}$)}$_2$(μ-Bi)] (6b)

Using toluene as reaction solvent: Toluene (10 mL) was added to the solid mixture of **1** (0.17 g, 0.2 mmol) and **4** (0.16 g, 0.1 mmol) in a 20 mL glass scintillation vial at room temperature. The mixture was stirred for two days with the exclusion of light, during which time it slowly turned into a dark red suspension with an oily precipitate. Celite® was added into the reaction, and then the mixture was heated up to 80 °C and filtered through a glass microfibre disc packed in a glass pipette to obtain a dark red solution. Volatiles were removed *in vacuo* to afford a red oily residue, which was extracted with Et_2O (3 mL) and then filtered. Storage of the red filtrate at −30 °C for two days yielded dark red-black crystals of **6a**/**6b**. Anal. Calcd for $C_{84}H_{186}N_{10}O_6KSi_6Bi_{2.56}U_2$ (**6a**:**6b** = 4:1): C, 38.06; H, 7.07; N, 5.28%. Found: C, 38.26; H, 6.73; N, 4.95%.

Using pentane as reaction solvent: Pentane (10 mL) was added to the solid mixture of **1** (0.17 g, 0.2 mmol) and **4** (0.16 g, 0.2 mmol) in a 20 mL glass scintillation vial at room temperature. The mixture was stirred for two days with the exclusion of light, resulting in a purple suspension. Celite® was added into the reaction, and then the mixture was heated up to 80 °C and filtered through a glass microfibre disc packed in a glass pipette to obtain a dark purple solution. Volatiles were removed *in vacuo* to afford a dark purple solid, which was extracted with Et_2O (3 mL) and then filtered. Storage of the purple filtrate at −30 °C for one day yielded dark purple crystals of **1** suggestive of no reaction occurring likely due to poor solubility of Bi reagents in the reaction solvent. ^{1}H NMR (D_6-benzene, 298 K): 17.55 (6H, s, CH_2), 7.09 (9H, s, Pr^i-CH), 3.92 (54H, s, Pr^i-CH_3), −40.23 (6H, s, CH_2) ppm.

Using THF as reaction solvent: THF (10 mL) was added to the solid mixture of **1** (0.17 g, 0.2 mmol) and **4** (0.16 g, 0.1 mmol) in a 20 mL glass scintillation vial at room temperature. The mixture was stirred for two days with the exclusion of light. Volatiles were removed *in vacuo* to afford a dark black/brown oily insoluble residue indicative of decomposition. NMR spectroscopic analysis of the reaction mixtures showed the presence of $Tren^{TIPS}H_3$ and 2.2.2-cryptand. NMR samples contained significant amounts of black insoluble solid in both D_6-benzene and D_8-THF. ^{1}H NMR (D_6-benzene, 298 K): δ 3.65 (12H, s, *C*H_2-cryptand), 3.50 (12H, t, *C*H_2-cryptand), 2.89 (6H, q, CH_2), 2.53 (12H, t, CH_2-cryptand), 2.44 (6H, t, CH_2), 1.15–0.95 (63H, m, Pr^i-CH_3 and Pr^i-C*H*), 0.77 (3H, t, -*H*) ppm. ^{1}H NMR (D_8-THF, 298 K): δ 3.53 (s), 2.89 (s), 2.58 (s), 2.51 (s), 1.07 (s) ppm. ^{1}H NMR integrations are internally consistent to the specific named molecule, but not relative to the whole mixture where they may represent a small percentage of the total constituents. ^{29}Si{^{1}H} NMR (D_8-THF, 298 K): δ 4.58 ppm.

Using pyridine as reaction solvent: Pyridine (10 mL) was added to the solid mixture of **1** (0.17 g, 0.2 mmol) and **4** (0.16 g, 0.1 mmol) in a 20 mL glass scintillation vial at room temperature. The mixture was stirred for two days with the exclusion of light. Volatiles were removed *in vacuo* to afford a dark black/brown oily insoluble residue indicative of decomposition. NMR spectroscopic analysis of the reaction mixtures showed the presence of $Tren^{TIPS}H_3$ and 2.2.2-cryptand. NMR samples contained significant amounts of black insoluble solid in D_6-benzene. ^{1}H NMR (D_6-benzene, 298 K): δ 3.64 (12H, s, CH_2-cryptand), 3.49 (12H, t, CH_2-cryptand), 3.34 (s), 2.88 (6H, q, CH_2), 2.52 (12H, t, CH_2-cryptand), 2.44 (6H, t, CH_2), 1.38 (s), 1.36 (s), 1.15–0.95 (63H, m, Pr^i-CH_3 and Pr^i-C*H*), 0.76 (3H, t, -*H*) ppm. ^{1}H NMR integrations are internally consistent to the specific named molecule, but not relative to the whole mixture where they may represent a small percentage of the total constituents.^{29}Si{^{1}H} NMR (D_6-benzene, 298 K): δ 4.40, 4.33, 2.06 ppm. The peaks at 1.38 and 1.36 ppm likely represent a pyridine reduction product, but this is unconfirmed.

Using Et_2O as reaction solvent: Et_2O (10 mL) was added to the solid mixture of **1** (0.17 g, 0.2 mmol) and **4** (0.16 g, 0.1 mmol) in a 20 mL glass scintillation vial at room temperature. The mixture was stirred for two days with the exclusion of light, during which time it slowly turned into a dark red suspension with an oily precipitate. Celite® was added into the reaction, and then the mixture was heated up to 80 °C and filtered through a glass microfibre disc packed in a glass pipette to obtain a dark red solution. Storage of the red filtrate at −30 °C for two days yielded dark red-black crystals of **6a**/**6b**. Anal. Calcd for $C_{84}H_{186}N_{10}O_6KSi_6Bi_{2.04}U_2$ (**6a**:**6b** = 1:1): C, 39.68; H, 7.37; N, 5.51%. Found: C, 40.22; H, 7.52; N, 5.66%.

Using DME as reaction solvent: DME (10 mL) was added to the solid mixture of **1** (0.17 g, 0.2 mmol) and **4** (0.16 g, 0.1 mmol) in a 20 mL glass scintillation vial at room temperature. The mixture was stirred for two days with the exclusion of light, during which time it slowly turned into a dark red suspension with an oily precipitate. Celite® was added into the reaction, and then the mixture was heated up to 80 °C and filtered through a glass microfibre disc packed in a glass pipette to obtain a dark red solution. Volatiles were removed *in vacuo* to afford a red oily residue, which was extracted with Et_2O (3 mL) and then filtered. Storage of the red filtrate at −30 °C for two days yielded dark red-black crystals of **6a**/**6b**. Anal. Calcd for $C_{84}H_{186}N_{10}O_6KSi_6Bi_{2.48}U_2$ (**6a**:**6b** = 3:1): C, 38.29; H, 7.11; N, 5.32%. Found: C, 38.77; H, 7.87; N, 4.87%.

Longer reaction time: Benzene (10 mL) was added to the solid mixture of **1** (0.17 g, 0.2 mmol) and **4** (0.16 g, 0.1 mmol) in a 20 mL glass scintillation vial at room temperature. The mixture was stirred for five days with the exclusion of light, during which time it slowly turned into a dark red suspension with an oily precipitate. Celite® was added into the reaction, and then the mixture was heated up to 80 °C and filtered through a glass microfibre disc packed in a glass pipette to obtain a dark red solution. Volatiles were removed *in vacuo* to afford a red oily residue, which was extracted with Et_2O

(3 mL) and then filtered. Storage of the red filtrate at −30 °C for five days yielded dark red-black crystals of **6a**/**6b** (**6a**:**6b** = 9:1).

Extraction into DME: Benzene (10 mL) was added to the solid mixture of **1** (0.17 g, 0.2 mmol) and **4** (0.16 g, 0.1 mmol) in a 20 mL glass scintillation vial at room temperature. The mixture was stirred for five days with the exclusion of light, during which time it slowly turned into a dark red suspension with an oily precipitate. Celite® was added into the reaction, and then the mixture was heated up to 80 °C and filtered through a glass microfibre disc packed in a glass pipette to obtain a dark red solution. Volatiles were removed *in vacuo* to afford a red oily residue, which was extracted with DME (3 mL) and then filtered. Storage of the red filtrate at −30 °C for two days yielded dark red-black crystals of **6a**/**6b** (**6a**:**6b** = 9:1).

Extraction into DCM: Benzene (10 mL) was added to the solid mixture of **1** (0.17 g, 0.2 mmol) and **4** (0.16 g, 0.1 mmol) in a 20 mL glass scintillation vial at room temperature. The mixture was stirred for five days with the exclusion of light, during which time it slowly turned into a dark red suspension with an oily precipitate. Celite® was added into the reaction, and then the mixture was heated up to 80 °C and filtered through a glass microfibre disc packed in a glass pipette to obtain a dark red solution. Volatiles were removed *in vacuo* to afford a red oily residue, which was extracted with DCM (3 mL) resulting in the immediate precipitation of black insoluble solid. The mixture was filtered resulting in a clear orange solution. Storage of the orange filtrate at −30 °C for one day yielded orange crystals of [U^{IV}($Tren^{TIPS}$)Cl].[1]

Reactivity of [K(2.2.2-cryptand)][{U^{IV}($Tren^{TIPS}$)}$_2$(μ-η^3:η^3-Bi_3)] (6a) in an attempt to remove co-crystallised sub-component [K(2.2.2-cryptand)][{U^{IV}($Tren^{TIPS}$)}$_2$(μ-Bi)] (6b)

*Reaction of **6** with 10% $AgBPh_4$:* Benzene (5 mL) was added to the solid mixture of **6a/b** (0.15 g, 0.055 mmol) and 0.1 equivalents of $AgBPh_4$ (0.0023 g, 0.0055 mmol) in a 20 mL glass scintillation

vial at room temperature. There was an immediate precipitation of black insoluble solid. The mixture was allowed to stir overnight before being filtered through Celite® and a glass microfibre disc packed in a glass pipette to obtain a dark yellow solution. Volatiles were removed *in vacuo* to afford a brown solid, which was extracted with Et_2O (1 mL) and then filtered. Storage of the brown filtrate at −30 °C for one day resulted in the deposition of black insoluble solid, amongst which a small number of colourless crystals of $KBPh_4$ were present, as confirmed by SC-XRD. *Note* – when conducting this reaction at −30 °C, the outcome is unchanged. NMR spectroscopic analysis of the reaction mixtures showed the presence of $Tren^{TIPS}H_3$ and 2.2.2-cryptand, as well as many unidentifiable paramagnetically shifted resonances. NMR samples contained significant amounts of black insoluble solid in D_6-benzene. ^1H NMR (D_6-benzene, 298 K): δ 76.05 (s), 42.53 (s), 37.51 (s), 30.20 (d), 26.76 (s), 18.90 (s), 14.64 (s), 11.14 (q), 10.29 (s), 8.13 (t), 7.25 (br), 7.13–6.96 (m), 3.65 (12H, s, CH_2-cryptand), 3.50 (12H, t, CH_2-cryptand), 2.88 (6H, q, CH_2), 2.53 (12H, t, CH_2-cryptand), 2.44 (6H, t, CH_2), 1.15–0.95 (63H, m, $Tren^{TIPS}H_3$, Pr^i-CH_3 and Pr^i-CH), 0.76 (3H, t, -*H*), −2.53 (d), −8.29 (s), −8.47 (s), −45.06 (s), −79.65 (s) ppm. ^1H NMR integrations are internally consistent to the specific named molecule, but not relative to the whole mixture where they may represent a small percentage of the total constituents. $^{29}Si\{^1H\}$ NMR (D_6-benzene, 298 K): δ 7.68, 4.41 ppm.

*Reaction of **6** with 10% CPh_3BPh_4 at room temperature:* Benzene (5 mL) was added to the solid mixture of **6a/b** (0.15 g, 0.055 mmol) and 0.1 equivalents of CPh_3BPh_4 (0.0031 g, 0.0055 mmol) in a 20 mL glass scintillation vial at room temperature. There was an immediate colour change to very deep red, followed by a colour change to dark brown with the gradual precipitation of black insoluble solid after approximately 20 seconds. The mixture was allowed to stir overnight before being filtered through Celite® and a glass microfibre disc packed in a glass pipette to obtain a dark orange solution. Volatiles were removed *in vacuo* to afford an orange solid, which was extracted with Et_2O (1 mL) and then filtered. Storage of the dark orange filtrate at −30 °C for three days yielded a small number of orange block crystals of **9**. Due to the very small crystalline amounts of this by-product (<1%), **9**

was not characterised beyond SC-XRD, elemental analysis, NMR and IR spectroscopy. Anal. Calcd for $C_{33}H_{76}N_4OSi_3U$: C, 45.70; H, 8.83; N, 6.46 %. Found: C, 45.48; H, 8.86; N, 6.85 %. ^{1}H NMR (D_6-benzene, 298 K): δ 29.20 (6H, s, C*H*$_2$), 11.94 (6H, s, C*H*$_2$), −4.04 (9H, s, Pr^{i}-C*H*), −5.16 (54H, s, Pr^{i}-C*H*$_3$) ppm. ^{29}Si{^{1}H} NMR (D_6-benzene, 298 K): δ −96.93 ppm. ATR-IR v/cm^{-1}: 2940 (s), 2885 (w), 2862 (s), 1460 (s), 1381 (m), 1354 (s), 1275 (s), 1132 (s, br), 1056 (s), 1026 (w), 1010 (w), 988 (m), 923 (s), 895 (w), 880 (s), 838 (w), 821 (w), 732 (s), 670 (s), 658 (w), 637 (s), 583 (w), 567 (m), 548 (s), 514 (s), 444 (s, br). NMR spectroscopic analysis of the crude reaction mixtures showed the presence of $HCPh_3$, Tren$^{TIPS}H_3$ and 2.2.2-cryptand, as well as many unidentifiable paramagnetically shifted resonances. NMR samples contained significant amounts of black insoluble solid in both D_6-benzene and D_8-THF. ^{1}H NMR (D_6-benzene, 298 K): δ 7.14–7.01 (m, 15H, HC-*Ph*$_3$), 5.41 (s, 1H, *H*CPh$_3$), 3.65 (12H, s, C*H*$_2$-cryptand), 3.50 (12H, t, C*H*$_2$-cryptand), 2.90 (6H, q, C*H*$_2$), 2.53 (12H, t, C*H*$_2$-cryptand), 2.45 (6H, t, C*H*$_2$), 2.12 (s), 1.16–0.95 (63H, m, Tren$^{TIPS}H_3$, Pr^{i}-C*H*$_3$ and Pr^{i}-C*H*), 0.77 (3H, t, -*H*), 0.16 (s) ppm. ^{1}H NMR (D_8-THF, 298 K): δ 48.85 (s), 46.77 (s), 45.93 (s), 45.15 (s), 34.62 (s), 30.87 (6H, s, **9**, C*H*$_2$), 26.93 (s), 25.54 (s), 18.96 (s), 15.41 (s), 14.16 (s), 13.09 (s), 12.55 (s), 12.40 (s), 11.99 (6H, s, **9**, C*H*$_2$), 11.69 (s), 10.46 (s), 10.19 (s), 9.31 (s), 7.80 (s), 7.60 (s), 7.32–7.10 (m, 15H, HC-*Ph*$_3$), 6.89 (s), 6.80 (s), 5.58 (s, 1H, *H*CPh$_3$), 4.30 (s), 4.04 (s), 3.66 (12H, s, C*H*$_2$-cryptand), 3.54 (12H, s, C*H*$_2$-cryptand), 2.88 (6H, s, Tren$^{TIPS}H_3$, C*H*$_2$), 2.64 (s), 2.58 (12H, t, C*H*$_2$-cryptand), 2.49 (6H, s, Tren$^{TIPS}H_3$, C*H*$_2$), 1.05 (s, Tren$^{TIPS}H_3$), −4.15 (9H, s, **9**, Pr^{i}-C*H*), −4.79 (s), 5.48 (54H, s, **9**, Pr^{i}-C*H*$_3$), −5.81 (s), −6.59 (s), −9.01 (s), −9.12 (s), −9.34 (s), −12.44 (s), −12.74 (s), −13.77 (s) ppm. ^{1}H NMR integrations are internally consistent to the specific named molecule, but not relative to the whole mixture where they may represent a small percentage of the total constituents.

At −30 °C: A solution of **6a/b** (0.15 g, 0.055 mmol) in Et_2O (2 mL) was stored at −30 °C for three hours. To this was added 0.1 equivalents of CPh_3BPh_4 (0.0031 g, 0.0055 mmol). There was an immediate colour change to very deep red. Storage of the reaction mixture at −30 °C for three days

resulted in a colour change to dark orange/brown and the deposition of a small number of dark orange block crystals of **9**.

*Reaction of **6** with 10% [HNEt$_3$][BPh$_4$]:* Benzene (5 mL) was added to the solid mixture of **6a/b** (0.15 g, 0.055 mmol) and 0.1 equivalents of [HNEt$_3$][BPh$_4$] (0.0023 g, 0.0055 mmol) in a 20 mL glass scintillation vial at room temperature. There was an immediate precipitation of black insoluble solid. The mixture was allowed to stir overnight before being filtered through Celite® and a glass microfibre disc packed in a glass pipette to obtain a dark red/black solution. Volatiles were removed *in vacuo* to afford a dark red/black solid, which was extracted with Et$_2$O (1 mL) and then filtered. Storage of the red/black filtrate at −30 °C for one day resulted in the deposition of lots of black insoluble solid, amongst which a small number of dark red-black block crystals of **9**, [{U^{III}(Tren^{TIPS}H)}$_2$(μ-η^6:η^6-C$_6$H$_6$)(OEt$_2$)$_2$] (**10**), and colourless crystals of KBPh$_4$, as confirmed by SC-XRD. **Note** - When conducting this reaction at −30 °C, the outcome is unchanged. Due to the very small amount of this by-product (<1%), **10** was not characterised beyond SC-XRD and IR spectroscopy. NMR spectroscopic analysis of the reaction mixtures showed the presence of Tren^{TIPS}H$_3$, **1**, **9**, and 2.2.2-cryptand, as well as many unidentifiable paramagnetically shifted resonances. It was not possible to accurately assign resonances belonging to **10**. NMR samples contained significant amounts of black insoluble solid in both D$_6$-benzene and D$_8$-THF. ^{1}H NMR (D$_6$-benzene, 298 K): δ 52.48 (s), 29.21 (6H, s, **9**, C*H*$_2$), 27.19 (s), 26.77 (s), 20.76 (s), 20.51 (s), 18.91 (s), 17.55 (**1**, 6H, s, C*H*$_2$), 16.88 (s), 16.39 (s), 15.67 (s), 11.95 (6H, s, **9**, C*H*$_2$), 10.30 (s), 9.09 (s), 7.36 (s), 7.09 (9H, s, **1**, Pr^i-C*H*), 6.96 (s), 3.92 (54H, s, **1**, Pr^i-C*H*$_3$), 3.66 (12H, s, C*H*$_2$-cryptand), 3.51 (12H, t, C*H*$_2$-cryptand), 2.89 (6H, q, Tren^{TIPS}H$_3$, C*H*$_2$), 2.53 (12H, t, C*H*$_2$-cryptand), 2.45 (6H, t, Tren^{TIPS}H$_3$, C*H*$_2$), 1.15–0.95 (63H, m, Tren^{TIPS}H$_3$, Pr^i-C*H*$_3$ and Pr^i-C*H*), 0.77 (3H, t, Tren^{TIPS}H$_3$, -*H*), −4.05 (9H, s, **9**, Pr^i-C*H*), −5.16 (54H, s, **9**, Pr^i-C*H*$_3$), −5.80 (s), −8.46 (s), −8.70 (s), 8.87 (s), −10.32 (s), −10.66 (s), −10.98 (s), −11.41 (s), −11.62 (s), −13.40 (s), −40.25 (6H, s, **1**, C*H*$_2$), −174.93 (s) ppm. ^{1}H NMR (D$_8$-THF, 298 K): δ 7.36 (s), 7.26 (s), 6.89 (s), 6.73 (s), 3.59 (s), 3.45 (s), 2.44 (s), 1.06 (s) ppm. ^{1}H NMR integrations are

internally consistent to the specific named molecule, but not relative to the whole mixture where they may represent a small percentage of the total constituents. $^{29}Si\{^1H\}$ NMR (D_6-benzene, 298 K): δ 7.68, 4.41 ppm. $^{11}B\{^1H\}$ NMR (D_8-THF, 298 K): δ −7.21 ppm. ATR-IR ν/cm^{-1}: 2938 (s), 2860 (s), 2717 (w), 1633 (m), 1461 (s), 1362 (m), 1328 (w), 1293 (m), 1271 (w), 1213 (w), 1123 (s), 1068 (s), 1013 (w), 981 (s), 920 (s), 881 (s), 850 (m), 833 (w), 817 (w), 774 (m), 735 (s), 668 (s), 630 (s), 581 (w), 567 (w), 546 (w), 514(w), 475 (w), 461 (w), 411 (w).

*Reaction of **6** with 10% $TlBPh_4$:* Benzene (5 mL) was added to the solid mixture of **6a/b** (0.15 g, 0.055 mmol) and 0.1 equivalents of $TlBPh_4$ (0.0029 g, 0.0055 mmol) in a 20 mL glass scintillation vial at room temperature. There was an immediate precipitation of black insoluble solid. The mixture was allowed to stir overnight before being filtered through Celite® and a glass microfibre disc packed in a glass pipette to obtain a dark red solution. Volatiles were removed *in vacuo* to afford a dark black iridescent solid, which was extracted with Et_2O (1 mL) and then filtered. Storage of the black filtrate at −30 °C for one day yielded a mixture of dark red-black crystals of **9**, **10**, and colourless crystals of $KBPh_4$ as confirmed by SC-XRD. **Note** -When conducting this reaction at −30 °C, the outcome is unchanged. NMR spectroscopic analysis of the reaction mixtures showed the presence of $Tren^{TIPS}H_3$, **1**, **9**, and 2.2.2-cryptand, as well as many unidentifiable paramagnetically shifted resonances. It was not possible to accurately assign resonances belonging to **10**. NMR samples contained significant amounts of black insoluble solid in both D_6-benzene and D_8-THF. ^1H NMR (D_6-benzene, 298 K): δ 52.46 (s), 46.94 (s), 29.21 (6H, s, **9**, C*H*$_2$), 27.22 (s), 26.75 (s), 20.88 (s), 20.53 (s), 17.54 (6H, s, **1**, C*H*$_2$), 16.46 (s), 11.94 (6H, s, **9**, C*H*$_2$), 10.29 (s), 9.08 (s), 7.37 (s), 7.08 (9H, s, **1**, Pr^i-C*H*), 3.91 (54H, s, **1**, Pr^i-C*H*$_3$), 3.66 (12H, s, C*H*$_2$-cryptand), 3.51 (12H, t, C*H*$_2$-cryptand), 2.89 (6H, q, $Tren^{TIPS}H_3$, C*H*$_2$), 2.53 (12H, t, C*H*$_2$-cryptand), 2.45 (6H, t, $Tren^{TIPS}H_3$, C*H*$_2$), 1.15–0.95 (63H, m, $Tren^{TIPS}H_3$, Pr^i-C*H*$_3$ and Pr^i-C*H*), 0.76 (3H, t, $Tren^{TIPS}H_3$, -*H*), −2.55 (s), −4.05 (9H, s, **9**, Pr^i-C*H*), −5.17 (54H, s, **9**, Pr^i-C*H*$_3$), −5.45 (s), −5.87 (s), −8.46 (s), −8.70 (s), −10.32 (s), −10.97 (s), −13.51 (s), −40.23 (6H, s, **1**, C*H*$_2$), −79.62 (s) ppm. ^1H NMR (D_8-THF, 298 K): δ 7.56 (s), 7.33 (s), 7.07 (s), 6.92 (s), 3.68 (s),

3.35 (s), 2.94 (s), 2.62 (s), 2.33 (s), 1.14 (s) ppm. ^{1}H NMR integrations are internally consistent to the specific named molecule, but not relative to the whole mixture where they may represent a small percentage of the total constituents. ^{29}Si{^{1}H} NMR (D_6-benzene, 298 K): δ 4.43 ppm.

*Reaction of **6** with 10% trimethylsilyl iodide:* To a benzene (10 mL) solution of **6a/b** (0.27 g, 0.1 mmol) in a 20 mL glass scintillation vial was added dropwise 0.1 equivalents of $(CH_3)_3SiI$ (0.002 g, 0.01 mmol) in benzene (2 mL) at room temperature. There was an immediate precipitation black insoluble solid, leaving a colourless solution. Removal of solvent *in vacuo* resulted in a small amount of colourless solid amongst lots of black insoluble solid. NMR spectroscopic analysis of the reaction mixture showed the presence of $Tren^{TIPS}H_3$. NMR samples contained significant amounts of black insoluble solid in D_6-benzene. ^{1}H NMR (D_6-benzene, 298 K): δ 3.41 (br, s), 2.89 (6H, q, C*H*$_2$), 2.43 (6H, t, C*H*$_2$), 1.14–0.95 (63H, m, $Tren^{TIPS}H_3$, Pr^i-C*H*$_3$ and Pr^i-C*H*), 0.76 (3H, t, -*H*) ppm. ^{1}H NMR integrations are internally consistent to the specific named molecule, but not relative to the whole mixture where they may represent a small percentage of the total constituents.

*Reaction of **6** with 10% Ph_3SnCl:* Benzene (10 mL) was added to the solid mixture of **6a/b** (0.15 g, 0.055 mmol) and 0.1 equivalents of Ph_3SnCl (0.002 g, 0.0055 mmol) in a 20 mL glass scintillation vial at room temperature. There was a gradual precipitation of black solid. The mixture was allowed to stir overnight before being filtered through Celite® and a glass microfibre disc packed in a glass pipette to obtain a dark orange solution. Volatiles were removed *in vacuo* to afford an orange solid, which was extracted with Et_2O (1 mL) and then filtered. Storage of the dark orange filtrate at −30 °C for one day yielded a mixture of dark orange crystals of [U^{IV}($Tren^{TIPS}$)Cl] and colourless crystals of Sn_2Ph_6 as confirmed by SC-XRD and ^{1}H NMR spectroscopy.[27] NMR spectroscopic analysis of the reaction mixtures showed the presence of $Tren^{TIPS}H_3$ and 2.2.2-cryptand. NMR samples contained significant amounts of black insoluble solid in D_6-benzene. ^{1}H NMR (D_6-benzene, 298 K): δ 9.09 (54H, s, [U^{IV}($Tren^{TIPS}$)Cl], Pr^i-C*H*$_3$), 8.41 (9H, s, [U^{IV}($Tren^{TIPS}$)Cl], Pr^i-C*H*), 7.75-7.60 (18H, m,

Sn_2Ph_6 -C*H*), 7.11–7.08 (18H, m, Sn_2Ph_6 -C*H*), 5.59 (6H, s, [U^{IV}($Tren^{TIPS}$)Cl], CH_2), 3.66 (12H, s, CH_2-cryptand), 3.51 (12H, t, CH_2-cryptand), 2.92 (6H, q, $Tren^{TIPS}H_3$, CH_2), 2.53 (12H, t, CH_2-cryptand), 2.46 (6H, t, $Tren^{TIPS}H_3$, CH_2), 1.17–0.95 (63H, m, $Tren^{TIPS}H_3$, Pr^i-CH_3 and Pr^i-C*H*), 0.78 (3H, t, $Tren^{TIPS}H_3$, -*H*), −36.43 (6H, s, [U^{IV}($Tren^{TIPS}$)Cl], CH_2) ppm. ^{1}H NMR integrations are internally consistent to the specific named molecule, but not relative to the whole mixture where they may represent a small percentage of the total constituents.

Reaction of 1 with K_5Bi_4 (11)

Benzene (10 mL) was added to the solid mixture of **1** (0.138 g, 0.16 mmol), **11** (0.084 g, 0.08 mmol), and 2.2.2-cryptand (0.031g, 0.16 mmol) in a 20 mL glass scintillation vial at room temperature. The mixture was stirred for two days with the exclusion of light, resulting in colourless solution with significant amounts of black insoluble solid present indicative of decomposition. The mixture was filtered through a glass microfibre disc packed in a glass pipette, and volatiles were removed *in vacuo* to afford a pale-yellow oil. NMR spectroscopic analysis of the reaction mixture showed the presence of $Tren^{TIPS}H_3$ and 2.2.2-cryptand. ^{1}H NMR (D_6-benzene, 298 K): 3.66 (12H, s, CH_2-cryptand), 3.50 (12H, s, CH_2-cryptand), 2.91 (6H, q, $Tren^{TIPS}H_3$, CH_2), 2.52 (12H, s, CH_2-cryptand), 2.43 (6H, s, $Tren^{TIPS}H_3$, CH_2), 1.14–0.95 (63H, m, $Tren^{TIPS}H_3$, Pr^i-CH_3 and Pr^i-C*H*), 0.77 (3H, s, $Tren^{TIPS}H_3$, -*H*) ppm. ^{29}Si{^{1}H} NMR (D_6-benzene, 298 K): δ 4.91 ppm. ^{1}H NMR integrations are internally consistent to the specific named molecule, but not relative to the whole mixture where they may represent a small percentage of the total constituents.

Reaction of 11 with [U^{IV}($Tren^{TIPS}$)(THF)][BPh_4]

Benzene (10 mL) was added to the solid mixture of [U^{IV}($Tren^{TIPS}$)(THF)][BPh_4] (0.31 g, 0.25 mmol), **11** (0.129 g, 0.125 mmol), and 2.2.2-cryptand (0.047g, 0.25 mmol) in a 20 mL glass scintillation vial at room temperature. The mixture was stirred for two days with the exclusion of light, resulting in a purple suspension. Celite® was added into the reaction, and then the mixture was heated up to 80 °C

and filtered through a glass microfibre disc packed in a glass pipette to obtain a dark purple solution. Volatiles were removed *in vacuo* to afford a dark purple solid, which was extracted with Et_2O (3 mL) and then filtered through a glass microfibre disc packed in a glass pipette. Volatiles were removed *in vacuo* to afford a dark purple solid. Subsequent NMR spectroscopic analysis of this solid showed **1** to be the major product, along with minor impurities of H_3Tren^{TIPS} and **9**, and some other unidentifiable paramagnetically shifted resonances. ^1H NMR (D_6-benzene, 298 K): 29.76 (6H, s, **9**, CH_2), 17.88 (6H, s, **1**, CH_2), 12.07 (6H, s, **9**, CH_2), 9.27, 7.08 (9H, s, **1**, Pr^i-C*H*), 4.00 (54H, s, **1**, Pr^i-CH_3), 3.30, 2.90 (6H, q, $Tren^{TIPS}H_3$, CH_2), 2.45 (6H, t, $Tren^{TIPS}H_3$, CH_2), 1.15–0.95 (63H, m, $Tren^{TIPS}H_3$, Pr^i-CH_3 and Pr^i-C*H*), 0.77 (3H, t, $Tren^{TIPS}H_3$, -*H*), –4.10 (9H, s, **9**, Pr^i-C*H*), –4.83, –5.28 (54H, s, **9**, Pr^i-CH_3), –5.95, –8.68, –12.50, –41.07 (6H, s, **1**, CH_2), –50.03 ppm. ^1H NMR integrations are internally consistent to the specific named molecule, but not relative to the whole mixture where they may represent a small percentage of the total constituents.

Synthesis of [U^{IV}($Tren^{TIPS}$)(μ-Cl)K(2.2.2-cryptand)] (12)

Benzene (10 mL) was added to the solid mixture of [U^{IV}($Tren^{TIPS}$)Cl] (0.11 g, 0.125 mmol), **11** (0.26 g, 0.25 mmol), and 2.2.2-cryptand (0.047g, 0.125 mmol) in a 20 mL glass scintillation vial at room temperature. The mixture was stirred for two days with the exclusion of light, resulting in a black suspension. Celite® was added into the reaction, and then the mixture was heated up to 80 °C and filtered through a glass microfibre disc packed in a glass pipette to obtain a blue-grey solution. Volatiles were removed *in vacuo* to afford a gray blue solid, which was extracted with hexane (3 mL) along with vigorous stirring and then filtered through a glass microfibre disc packed in a glass pipette. Storage of the blue-grey filtrate at –30 °C for one day yielded dark blue crystals of **12**. Yield: 0.040 g, 24.6 %. Anal. Calcd for $C_{51}H_{111}N_6Si_3O_6KClU$: C, 47.07; H, 8.60; N, 6.46 %. Found: C, 46.87; H, 8.44; N, 6.38%. ^1H NMR (D_6-benzene, 298 K): 5.55 (6H, s, CH_2), 3.56 (9H, s, Pr^i-C*H*), 2.61 (54H, s, Pr^i-CH_3), 2.37 (12H, s, CH_2-cryptand), 2.32 (12H, t, CH_2-cryptand), 1.40 (12H, s, CH_2-cryptand), –5.42 (6H, s, CH_2) ppm. Despite repeated attempts, no signal could be observed in the $^{29}Si\{^1H\}$ NMR

spectrum of **12**. ATR-IR v/cm^{-1}: 2937 (w), 2881 (w), 2857 (s), 2809 (m), 1478 (w), 1459 (m), 1443 (w), 1377 (w), 1354 (m), 1336 (w), 1295 (m), 1272 (w), 1260 (m), 1239 (m), 1175 (w), 1134 (m), 1106 (s), 1073 (s), 1056 (w), 1028 (m), 1009 (m), 982 (m), 952 (m), 933 (s), 879 (s), 832 (w), 802 (w), 744 (s), 668 (s), 654 (w), 623 (s), 561 (m), 547 (w), 522 (w), 508 (s), 439 (m).

Synthesis of [Tach^{DIPP}H$_3$] (13)

To a pre-cooled (0 °C) solution of *cis,cis*-1,3,5-triaminocyclohexane (1.29 g, 10.00 mmol) and Et_3N (6.95 mL, 50 mmol) in DCM (100 mL) was added $Pr^i{}_2PCl$ (5.34 g, 35.00 mmol) dropwise, resulting in the immediate precipitation of a white solid. The resultant suspension was allowed to warm slowly to room temperature, before being stirred for 12 hours. Volatiles were removed *in vacuo* to afford a white solid, which was extracted with Et_2O (60 mL) and then filtered. Removal of volatiles *in vacuo* yielded **13** as an analytically pure white powder. Yield: 3.30 g, 69%. Anal. Calcd for $C_{24}H_{54}N_3P_3$: C, 60.35; H, 11.40; N, 8.80%. Found: C, 60.56; H, 11.68; N, 8.71%. ^{1}H NMR (D$_6$-benzene, 298 K): δ 2.72 (m, 3H, C*H*-Cy), 2.29 (d, 3H, C*H$_2$*-Cy), 1.41 (m, 6H, C*H*-Pr$^i{}_2$P), 1.11-0.94 (m, 36H, C*H$_3$*-Pr$^i{}_2$P), 0.85 (q, 3H, N*H*), 0.74 (m, 3H, C*H$_2$*-Cy) ppm. ^{13}C{^{1}H} NMR (D$_6$-benzene, 298 K) δ 54.00 (d, *C*H-Cy), 47.45 (t, *C*H$_2$-Cy), 26.75 (d, *C*H-Pr$^i{}_2$P), 19.54 (d, *C*H$_3$-Pr$^i{}_2$P), 17.45 (d, *C*H$_3$-Pr$^i{}_2$P) ppm. ^{31}P{^{1}H} NMR (D$_6$-benzene, 298 K) δ 55.99 (s) ppm. ATR-IR v/cm^{-1}: 3311 (m, NH-stretch), 2944 (w), 2928 (w), 2863 (m), 1461 (s), 1406 (s), 1379 (m), 1359 (m), 1348 (w), 1266 (s), 1235 (m), 1153 (w), 1128 (vs), 1086 (s), 1070 (s), 1014 (s), 984 (w), 924 (m), 876 (vs), 821 (s), 657 (vs), 610 (s), 572 (w), 479 (w), 447 (m), 415 (m).

Synthesis of [{(Tach^{DIPP})ThCl}$_2$(μ-LiCl)] (14)

To a pre-cooled (−78 °C) solution of **13** (2.39 g, 5.00 mmol) in THF (30 mL) was added Bu^{n}Li (2.50 M, 6.60 mL, 16.50 mmol) dropwise. After addition, the solution was allowed to warm slowly to room temperature, before being stirred for 3 hours. The resultant pale-yellow solution was then added to a pre-cooled (−78 °C) solution of $ThCl_4(THF)_{3.5}$ (3.13 g, 5.00 mmol) in THF (20 mL), before being

warmed slowly to room temperature and stirred for 12 hours. Volatiles were removed *in vacuo* to afford a white solid, which was extracted with toluene (80 mL) and then filtered. Volatiles were removed *in vacuo* to afford an off-white powder, which was washed with HMDSO (2 × 10 mL). The resultant solid was dried *in vacuo* to afford a white powder. Colourless single crystals suitable for SC-XRD were grown from a saturated solution of **14** in Et_2O stored at −30 °C. Yield: 2.48 g, 65%. $C_{48}H_{102}Cl_3LiN_6P_6Th_2$: C, 37.77; H, 6.73; N, 5.51%. Found: C, 37.68; H, 6.79; N, 5.62%. ^{1}H NMR (D_8-THF, 298 K) δ 4.42–4.01 (m, 6H, C*H*-Cy), 2.20–1.98 (m, 12H, C*H*-Pr^i_2P), 1.83–1.61 (m, 12H, CH_2-Cy; this resonance could not be definitively assigned or integrated due to overlapping with peaks from the D_8-THF solvent used for the NMR experiments), 1.20–1.08 (m, 72H, CH_3-Pr^i_2P) ppm. ^{13}C{^{1}H} NMR (D_8-THF, 298 K) δ 52.13 (d, *C*H-Cy), 42.66 (s, *C*H_2-Cy), 26.41 (d, *C*H-Pr^i_2P), 21.27 (d, *C*H_3-Pr^i_2P), 20.27 (d, *C*H_3-Pr^i_2P) ppm. ^{31}P{^{1}H} NMR (D_8-THF, 298 K) δ 23.28–17.14 (m) ppm. ^{7}Li{^{1}H} NMR (D_8-THF, 298 K) δ 0.99 (s) ppm. ATR-IR v/cm^{-1}: 2945 (w), 2920 (w), 2888 (w), 2863 (m), 1601 (w), 1459 (m), 1417 (w), 1379 (w), 1361 (w), 1276 (w), 1241 (m), 1138 (s), 1091 (s), 1032 (w), 984 (w), 957 (m), 920 (w), 844 (vs), 778 (m), 764 (s), 712 (m), 653 (s), 607 (s), 586 (s), 536 (s), 464 (w).

Synthesis of [{(Tach^{DIPP})ThCl(THF)}$_2$(μ-MgCl$_2$)] (15)

To a pre-cooled (−78 °C) solution of **14** (0.76 g, 0.50 mmol) in THF (20 mL) was added C_3H_5MgCl (2.00 M, 0.55 mL, 1.10 mmol) dropwise. After addition, the solution was allowed to warm slowly to room temperature, before being stirred for 12 hours. Volatiles were removed *in vacuo* to afford a white solid, which was extracted with toluene (20 mL) and filtered. Volatiles were removed *in vacuo* to afford an off-white powder, which was washed with pentane (2 × 5 mL). The resultant solid was dried *in vacuo* to afford a white powder. Colourless single crystals suitable for SC-XRD were grown from a saturated solution of **15** in Et_2O stored at −30 °C. Yield: 0.61 g, 70%. Anal. Calcd for $C_{56}H_{118}Cl_4MgN_6O_2P_6Th_2$•($Et_2O$): C, 40.09; H, 7.18; N, 4.67%. Found: C, 40.25; H, 7.31; N, 4.82%. ^{1}H NMR (D_8-THF, 298 K) δ 4.18 (m, 6H, C*H*-Cy), 3.58 (m, 8H, CH_2-THF; this resonance could not

be definitively assigned or integrated due to overlapping with peaks from the D_8-THF solvent used for the NMR experiments), 2.09 (m, 12H, C*H*-Pr^i_2P), 1.78−1.72 (m, 12H, C*H₂*-Cy; this resonance could not be definitively assigned or integrated due to overlapping with peaks from the D_8-THF solvent used for the NMR experiments), 1.69 (s, 8H, C*H₂*-THF), 1.12 (m, 72H, C*H₃*-Pr^i_2P) ppm. $^{31}P\{^1H\}$ NMR (D_8-THF, 298 K) δ 57.34 (s), 21.80 (br) ppm. The poor solubility of **15** in THF once isolated in pure form precluded the acquisition of reliable $^{13}C\{^1H\}$ NMR spectroscopic data. ATR-IR ν/cm^{-1}: 2946 (w), 2905 (w), 2891 (w), 2862 (m), 1457 (m), 1417 (w), 1379 (w), 1360 (w), 1330 (w), 1275 (w), 1231 (w), 1140 (s), 1093 (m), 1065 (m), 1021 (m), 977 (m), 919 (m), 882 (w), 867 (w), 842 (vs), 777 (m), 763 (s), 714 (s), 650 (s), 604 (s), 578 (s), 537 (s), 474 (s), 453 (s), 432 (s), 416 (w).

Synthesis of [K(2.2.2-cryptand)][{(Tach^{DIPP})Th}$_2$(μ-η^3:η^3-Bi$_3$)] (16)

THF (10 mL) was added to the solid mixture of **14** (0.12 g, 0.07 mmol) and **11** (0.14 g, 0.14 mmol) in a 20 mL glass scintillation vial at room temperature. The mixture was stirred for four days with the exclusion of light, during which time it slowly turned into a dark green suspension. 2.2.2-cryptand (0.0414 g, 0.11 mmol) was then added into the reaction, and the mixture stirred for a further 12 hours at room temperature. The resultant mixture was filtered through a glass microfibre disc packed in a glass pipette to obtain a dark green solution, which was concentrated *in vacuo* to ~3 mL. Addition of three drops of benzene, a layer of Et_2O (5 mL), and storage of this solution at –30 °C for 24 hours yielded **16** as dark green/black crystals that were isolated by decanting the mother liquor, washing with cold Et_2O (2 × 1 mL), and then drying *in vacuo.* Yield: 0.0378 g, 22% (by Th content). Anal. Calcd for $C_{66}H_{138}Bi_3KN_8O_6P_6Th_2$•$(Et_2O)(C_6H_6)_{0.5}$: C, 34.13; H, 5.92; N, 4.36%. Found: C, 34.06; H, 5.98; N, 4.41%. ^1H NMR (D_8-THF, 298 K) δ 4.12 (d, 6H, C*H*-Cy), 3.64 (s, 12H, C*H₂*-cryptand), 3.58 (t, 12H, C*H₂*-cryptand), 2.58 (t, 12H, C*H₂*-cryptand), 2.09−1.96 (m, 12H, C*H*-Pr^i_2P), 1.57 (m, 12H, C*H₂*-Cy), 1.14 (m, 72H, C*H₃*-Cy) ppm. $^{13}C\{^1H\}$ NMR (D_8-THF, 298 K) δ 71.38 (s, *C*H₂-cryptand), 68.39 (s, *C*H₂-cryptand), 54.68 (s, *C*H₂-cryptand), 53.41 (m, *C*H-Cy), 42.93 (s, *C*H₂-Cy), 26.62 (d,

*C*H-Pr^i_2P), 21.65 (m, *C*H_3-Pr^i_2P) ppm. $^{31}P\{^1H\}$ NMR (D_8-THF, 298 K) δ 40.92 (s) ppm. ATR-IR ν/cm^{-1}: 2939 (w), 2858 (m), 1476 (w), 1457 (w), 1444 (w), 1417 (w), 1377 (w), 1354 (s), 1325 (w), 1295 (w), 1275 (w), 1259 (w), 1225 (w), 1143 (w), 1130 (w), 1102 (m), 1078 (m), 1028 (w), 1010 (w), 948 (s), 931 (w), 878 (m), 844 (s), 765 (vs), 696 (s), 642 (s), 604 (s), 579 (w), 532 (vs), 465 (m).

General Computational Details

Density functional calculations were carried out with the program suite TURBOMOLE.[28] All structure optimisations of the anion components of **6a** (**6a'**), the Th analogue of **6a** (**6a'(Th)**), **6b'**, and **16** (**16'**) were done using the PBE functional[29] and def2-TZVP bases[30] for Bi and N, def2-SV(P) bases for C, H and Si, along with effective core potentials dhf-ECP-60[31] for Bi. For U and Th, mwb-ECP-60[32] were employed, together with the segmented contracted basis by Cao, Dolg and Stoll,[33] but with a modified g set consisting of three primitives with exponents of 4.5/1.4/0.37 for U and 3.94/1.23/0.32 for Th. The D3-BJ dispersion correction[34] was employed throughout, and for the title compounds C_3 symmetry was assumed. The structures optimised in this way are minimum structures, lowest frequencies amount to 13 (**6a'**), 11 (**6a'(Th)**), 16 (**6b'**) and 7 (**16'**) cm^{-1}. For the calculations of the isolated Bi_3^{3-} ring and related ring systems (see below), the conductor-like screening model (COSMO)[35] was used with default parameters to model the anionic environment and thus ensure negative orbital energies. The numerical grids used were of fine size (grid size 5, including weight derivatives for all gradient and force constant calculations),[36] and the multipole-accelerated RI approximation was used.[36-38] The Cartesian coordinates are deposited in Supplementary Data File 1, all subsequent calculations were done for these structure parameters.

For the ring currents, the magnetic response was calculated with the module for chemical shielding tensors[39] in TURBOMOLE, currents were obtained from this using the gauge-independent magnetically induced current (GIMIC) tool.[40]

Electronic excitations were calculated with the PBE0 functional.[41] Plots of the difference of densities of the ground state and the corresponding excited states were generated as described previously.[42] The MOs and their energies shown in the main text were obtained at this level. For bare Bi_3^{3-} a Pipek-Mezey localisation procedure[43] was done. Resulting localised MOs are shown together with canonical MOs in Supplementary Figures 130 and 131.

Supplementary Tables 8 and 9 document a thorough methodological study on bare Bi_3^{3-}, quantifying the influence of the functional (PBE vs. PBE0), of relativity described by ECPs vs. X2C without and with spin-orbit coupling (SOC, via two-component X2C) on bare Bi_3^{3-}. In Supplementary Table 8, structure parameters for the triangular and a bent structure are listed. The impact of the functional choice is quite substantial, distances with PBE0 are shorter than with PBE by ~5pm, the effect of SOC (2c vs. 1c) is of similar size, but opposed to that of the change from PBE to PBE0); the differences between X2C and ECPs are very small compared to that. Further data for Bi_3^{3-} at the same levels are listed in Supplementary Table 9, namely the energy of the bent relative to the triangular structure, the lowest excitation energies, for the one-component calculations separately listed for singlet and triplet, induced magnetic currents, and nucleus-independent chemical shifts 0, 1, 2, 3 Bohr above the ring centre.

Supplementary Table 10 lists energies of the bent structure relative to the triangular structure in kJ mol^{-1} for compounds related to Bi_3^{3-} , namely Sbi_3^{3-}, Po_3, and Te_3 at levels PBE0/ECP, PBE/1c-X2C, PBE/2c-X2C, PBE0/2c-X2C.

Supplementary Table 11 lists ring currents and nucleus-independent chemical shifts for the triangular isomers with very similar numbers for X2C and ECPs at one-component level and only small changes when considering SOC (~10% for Bi). The impact of the functional was exemplarily checked for Bi_3^{3-}: PBE0/X2C NICS(0/1/2/3) for the PBE/X2C structure amount to –43/–34/–19/–9 ppm, and thus are very similar to the PBE/X2C NICS, –42/–33/–18/–9 ppm. All systems exhibit ring currents

between 10 and 18 nA/T (comparable to benzene, 12 nA/T), which are not substantially changed by the presence of actinides.

In Supplementary Table 12 we list the lowest 50 (spin-conserving) excitations for both irrep a and (two-fold degenerate) irrep e of **6a'** and the hypothetical Th analogue **6a'(Th)**, obtained at TDDFT(PBE0) level with ECPs assuming C_3 symmetry, and the lowest 100 excitations for related Th compound **16'** (in C_1 symmetry. In Supplementary Table 13 we list the lowest 10 excitations for each irrep obtained with X2C for **6a'** and **6a'(Th)**, and the lowest 20 excitations for **16'**. The spectra are visualised in Supplementary Figures 132-134 together with composite excess density for the ground and excited states for groups of excitations that are energetically well separated from the others. These plots provide an aggregated representation of the electron transfer for each excitation group containing weighted contributions of each excitation within the group by its oscillator strength.

Optimised Cartesian coordinates for **6a', 6a'(Th)** and **16'**, the tri-anionic three-membered systems, their mono-cationic counterparts, and frequences for **6a', 6b'**, and **16'** are given in Supplementary Data File 1. We note that the optimised global minimum structure of **6b'** in the gas-phase departs from the eclipsed form found in the solid-state, moving towards a staggered form; nevertheless, the optimised gas-phase structure provides a representative description of the electronic structure of **6b'** in the solid-state.

Supplementary Figures

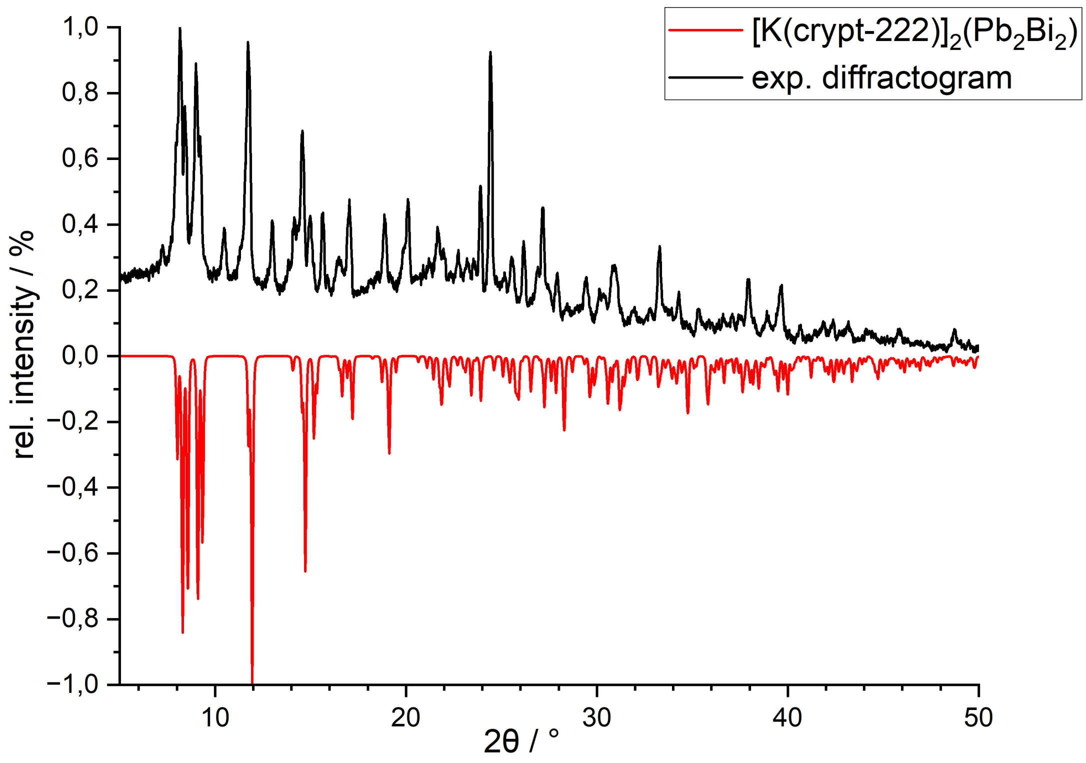

Supplementary Figure 1. Selected-range PXRD pattern of **2**.

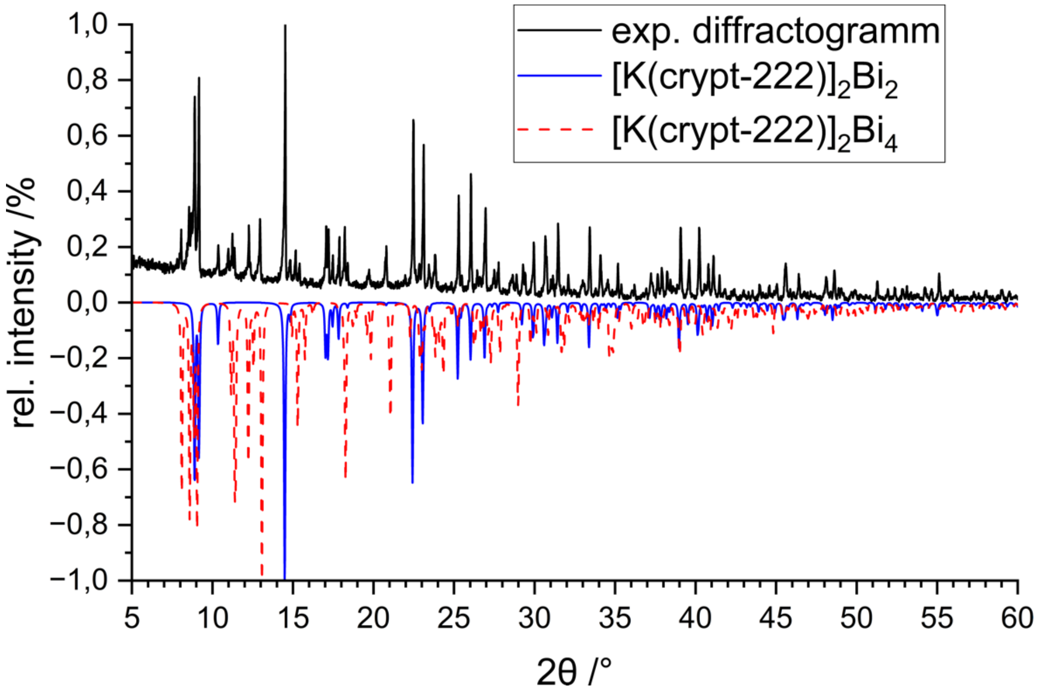

Supplementary Figure 2. Selected-range PXRD pattern of **3**.

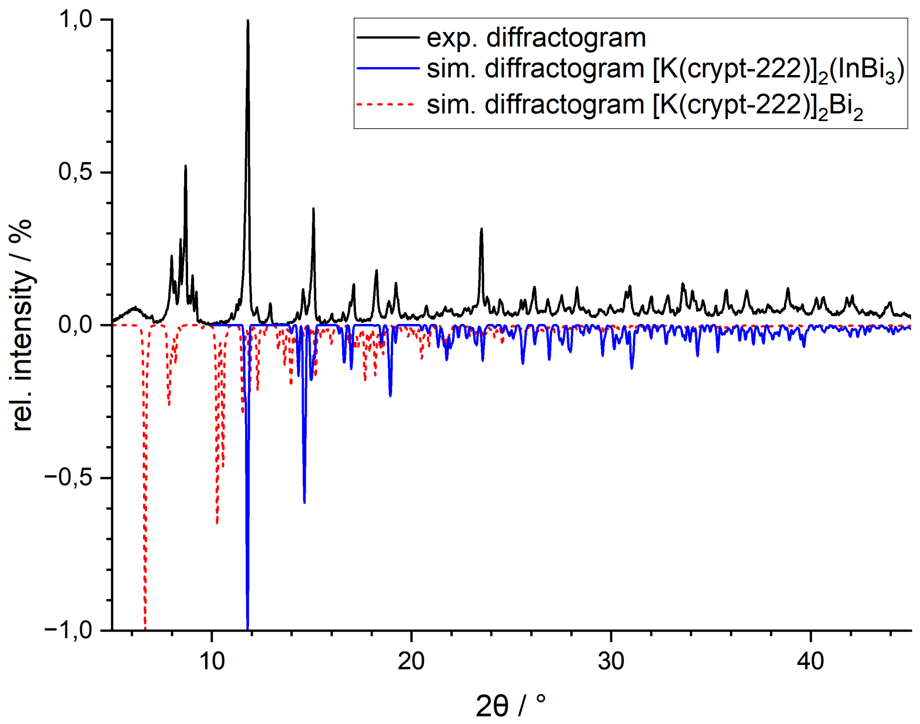

Supplementary Figure 3. Selected-range PXRD pattern of **4**.

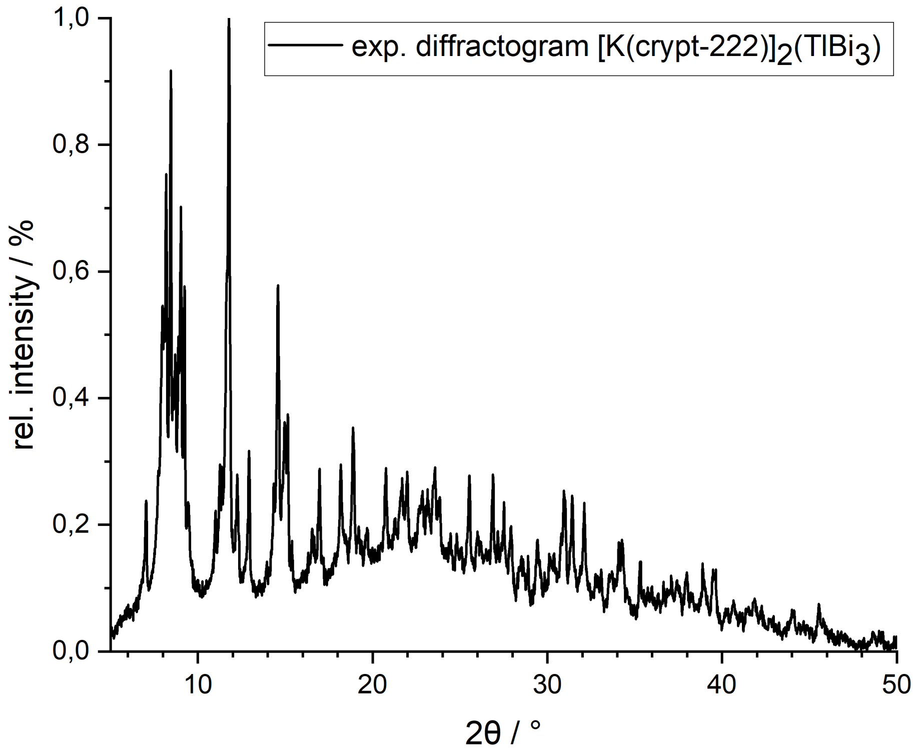

Supplementary Figure 4. Selected-range PXRD pattern of **5**. The amorphous broad signal arises from the capillary used for the measurement. There is no reference data for this compound as the reported structure of the $TlBi_3$ anion is a THF solvate, however, the PXRD spectrum shown is consistent with multiple different measurements and subsequent reactivity.

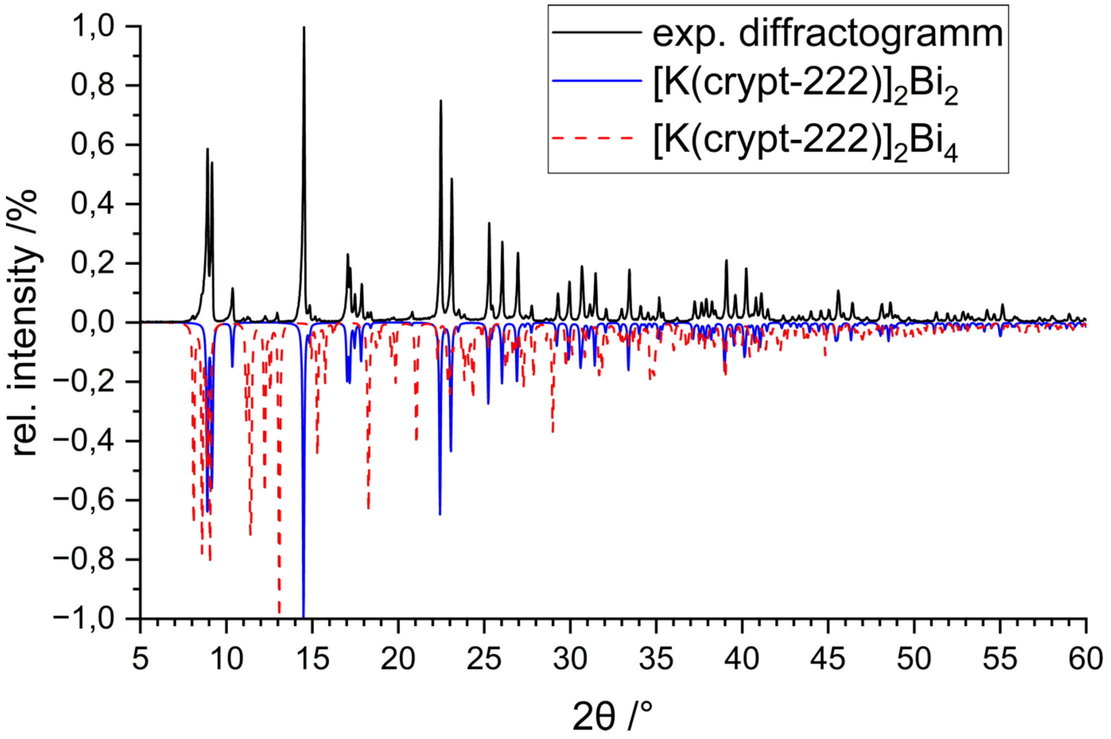

Supplementary Figure 5. Selected-range PXRD pattern of **7**.

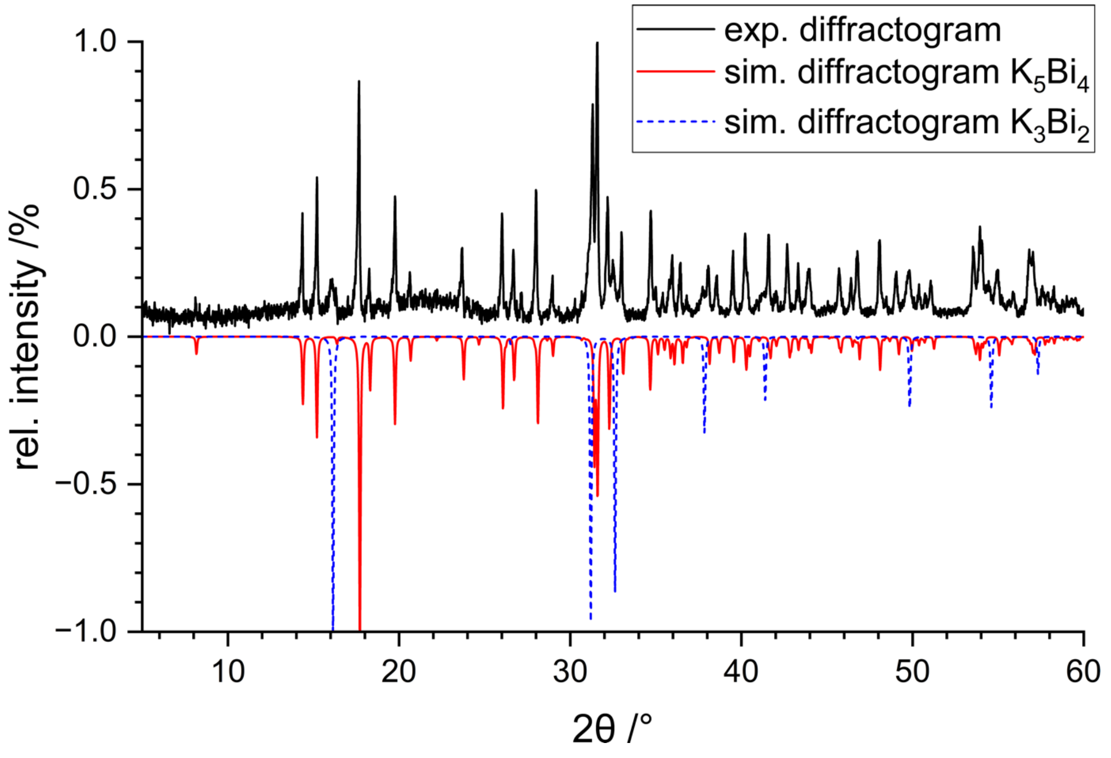

Supplementary Figure 6. Selected-range PXRD pattern of **11**.

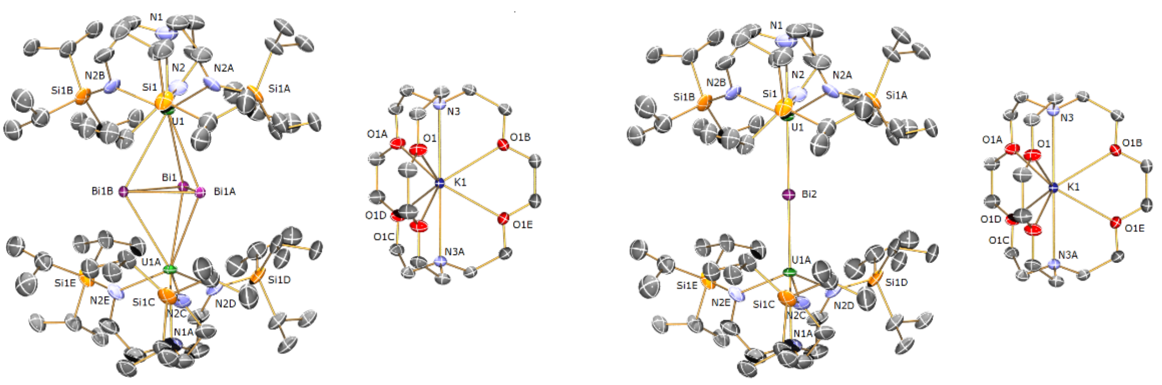

Supplementary Figure 7. Single crystal X-ray diffraction solid state structure of **6a6b-1** at 150 K with displacement ellipsoids at 30% and selective labelling. Hydrogen atoms and disordered components are omitted for clarity. CCDC deposition number 2270435.

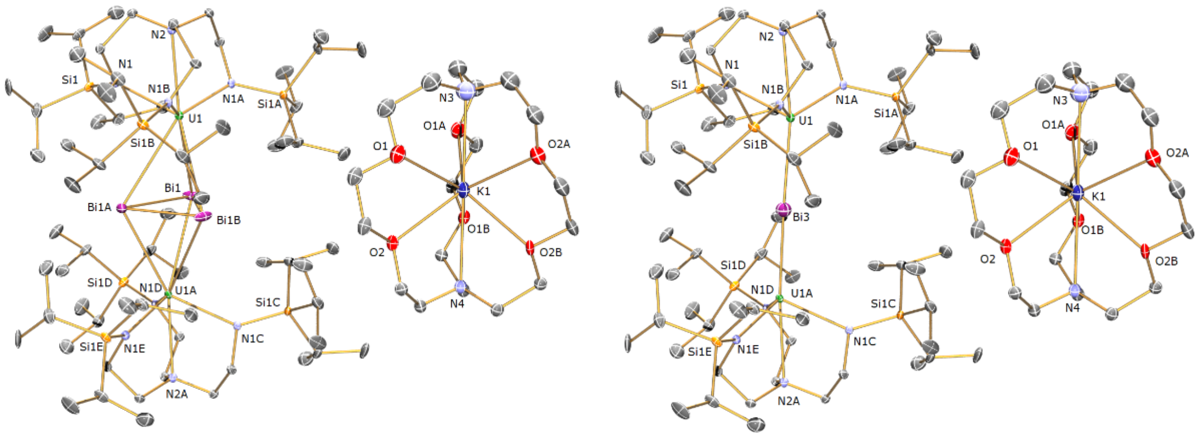

Supplementary Figure 8. Single crystal X-ray diffraction solid state structure of **6a6b-2** at 100 K with displacement ellipsoids at 30% and selective labelling. Hydrogen atoms and disordered components are omitted for clarity. CCDC deposition number 2491429.

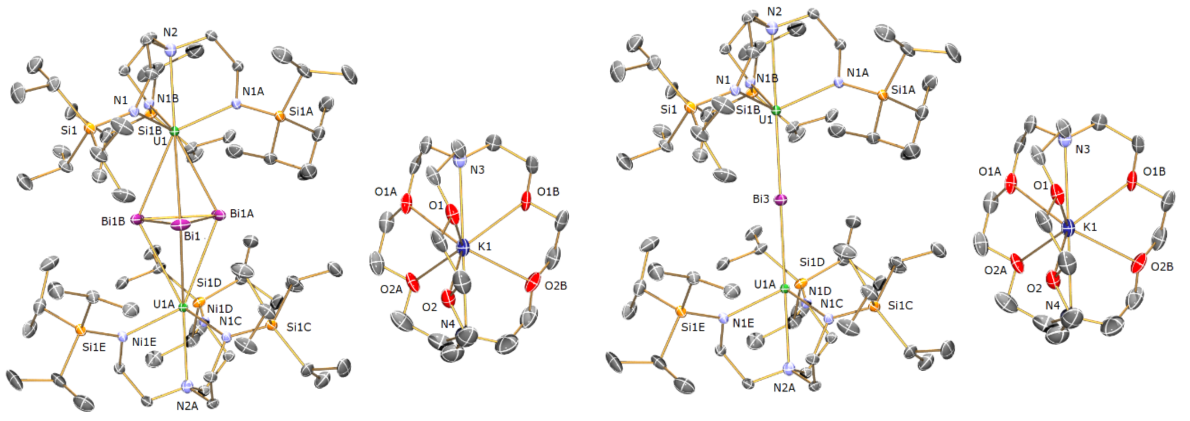

Supplementary Figure 9. Single crystal X-ray diffraction solid state structure of **6a6b-3** at 150 K with displacement ellipsoids at 30% and selective labelling. Hydrogen atoms and disordered components are omitted for clarity. CCDC deposition number 2491430.

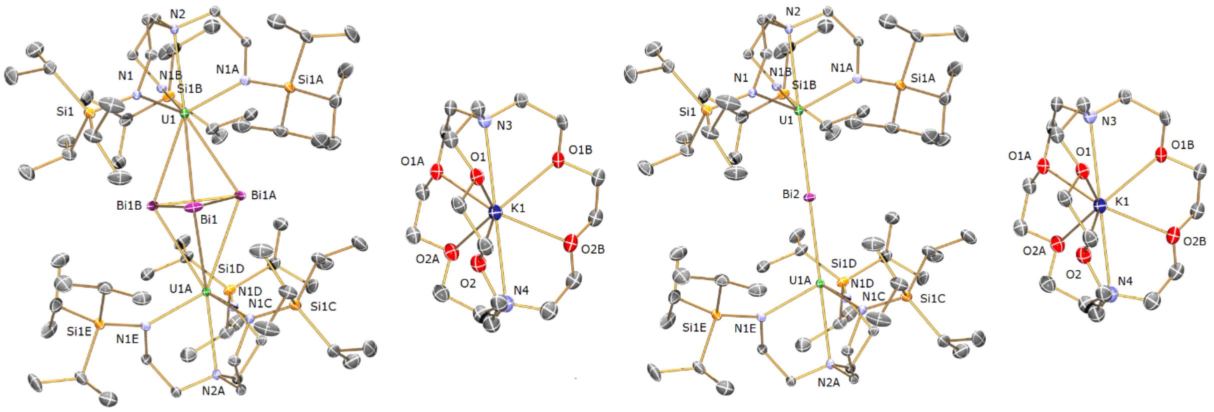

Supplementary Figure 10. Single crystal X-ray diffraction solid state structure of **6a6b-4** at 150 K with displacement ellipsoids at 30% and selective labelling. Hydrogen atoms and disordered components are omitted for clarity. CCDC deposition number 2491431.

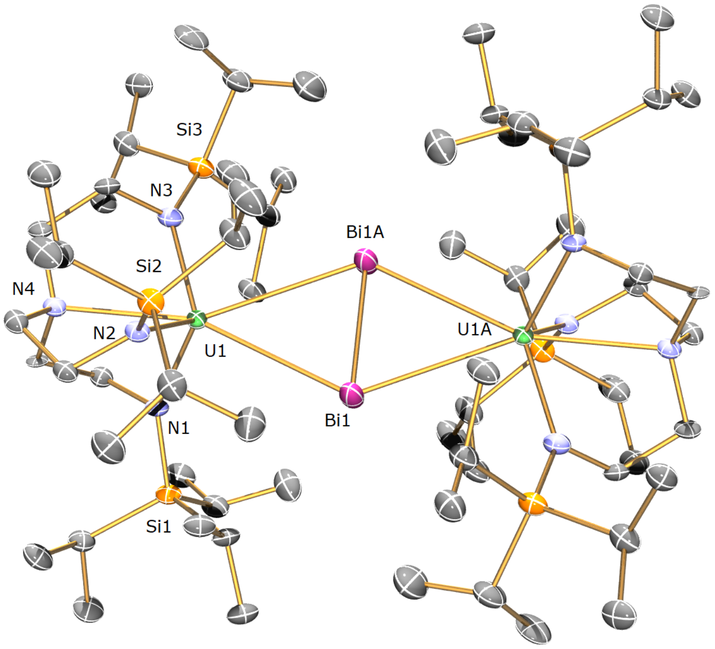

Supplementary Figure 11. Single crystal X-ray diffraction solid state structure of **8** at 100 K with displacement ellipsoids at 30% and selective labelling. Hydrogen atoms and disordered components are omitted for clarity. CCDC deposition number 2491432.

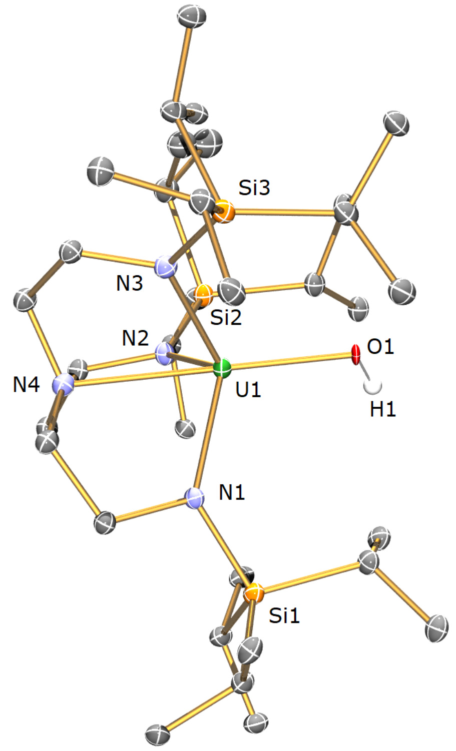

Supplementary Figure 12. Single crystal X-ray diffraction solid state structure of **9** at 100 K with displacement ellipsoids at 40% and selective labelling. Hydrogen atoms except the O-H are omitted for clarity. CCDC deposition number 2491433.

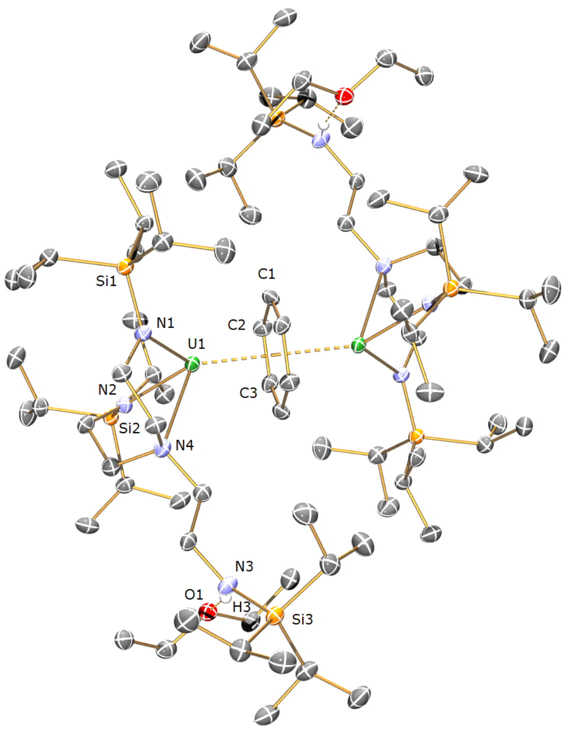

Supplementary Figure 13. Single crystal X-ray diffraction solid state structure of **10** at 100 K with displacement ellipsoids at 30% and selective labelling. Hydrogen atoms except the N-H are omitted for clarity. CCDC deposition number 2491434.

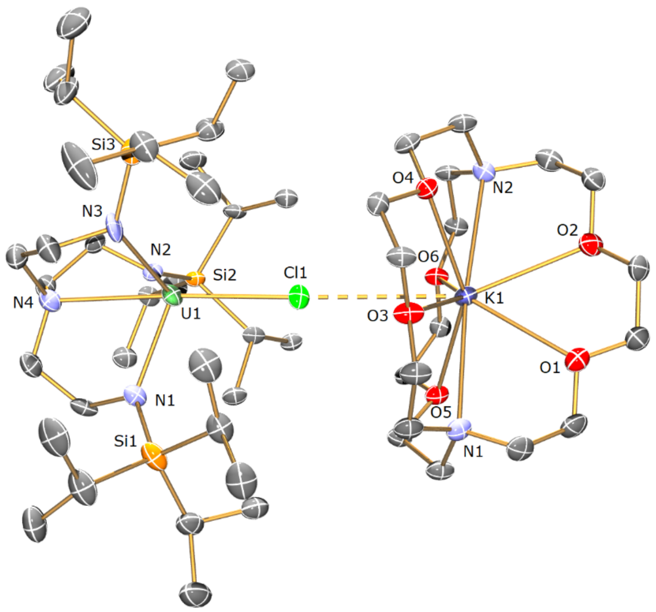

Supplementary Figure 14. Single crystal X-ray diffraction solid state structure of **12** at 100 K with displacement ellipsoids at 30% and selective labelling. Hydrogen atoms and disordered components are omitted for clarity. There are five molecules of **12** in the asymmetric unit, they are very similar so only one is shown for clarity. CCDC deposition number 2492183.

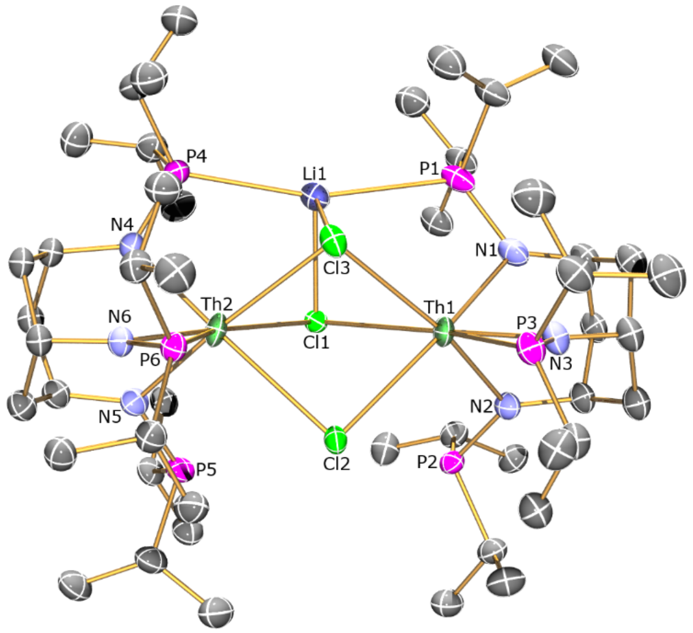

Supplementary Figure 15. Single crystal X-ray diffraction solid state structure of **14** at 150 K with displacement ellipsoids at 20% and selective labelling. Hydrogen atoms and disordered components are omitted for clarity. CCDC deposition number 2491451.

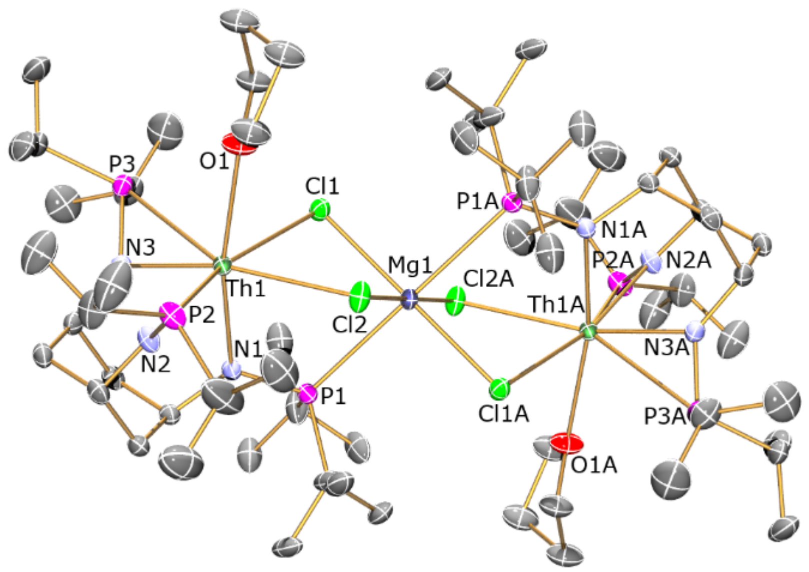

Supplementary Figure 16. Single crystal X-ray diffraction solid state structure of **15** at 150 K with displacement ellipsoids at 40% and selective labelling. Hydrogen atoms, lattice solvents, and disordered components are omitted for clarity. CCDC deposition number 2491452.

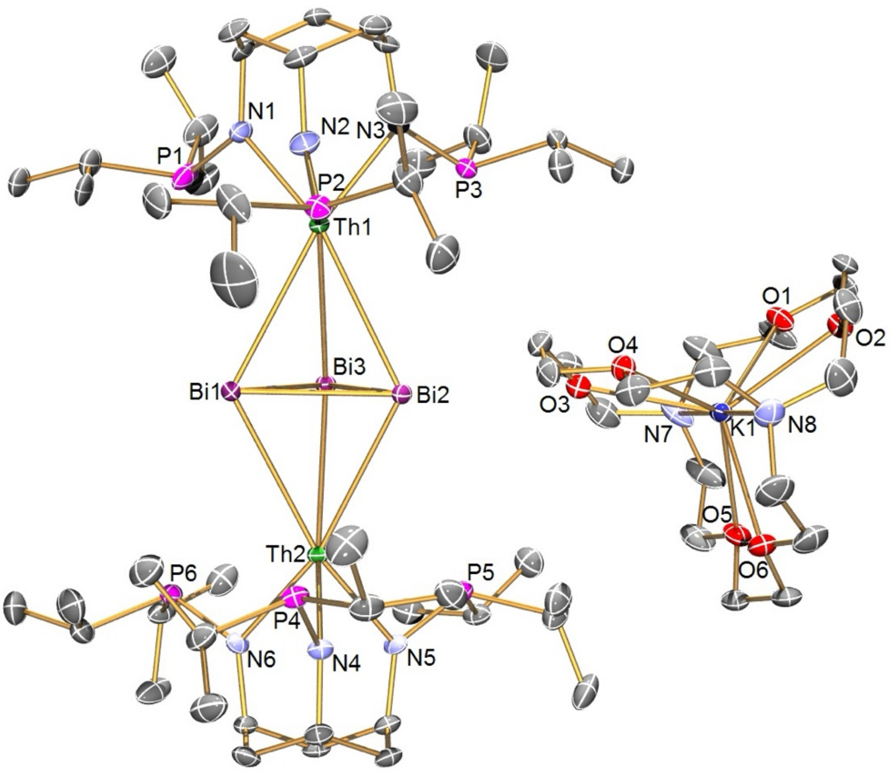

Supplementary Figure 17. Single crystal X-ray diffraction solid state structure of **16** at 150 K with displacement ellipsoids at 40% and selective labelling. Hydrogen atoms, lattice solvents, and disordered components are omitted for clarity. CCDC deposition number 2491453.

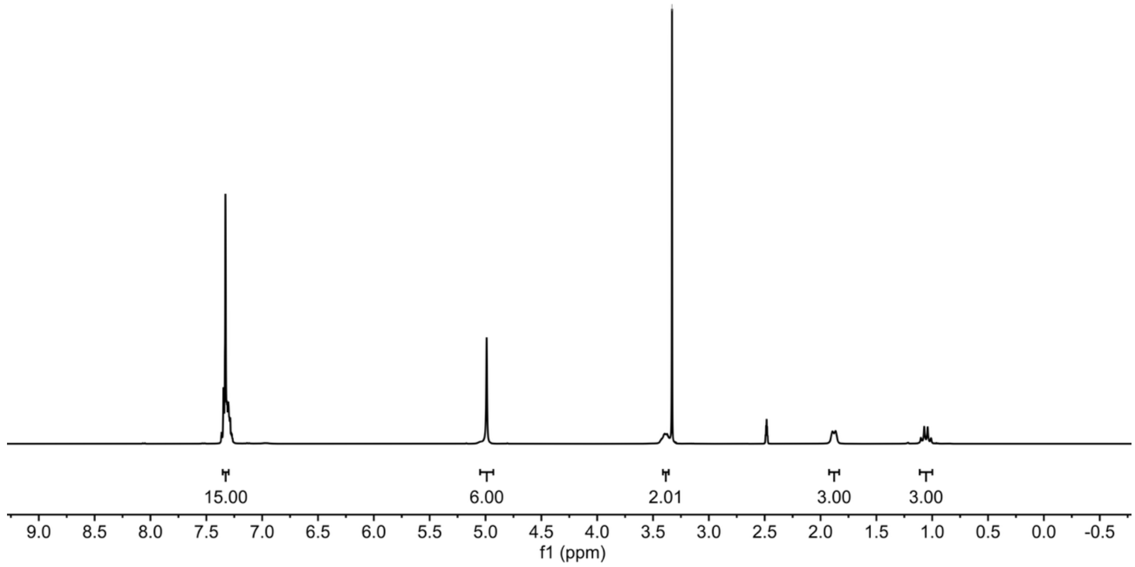

Supplementary Figure 18. ^{1}H NMR (D_6-DMSO, 298 K) spectrum of *cis*,*cis*-1,3,5-*tris*-[{(benzyloxy)carbonyl}aminocyclohexane.

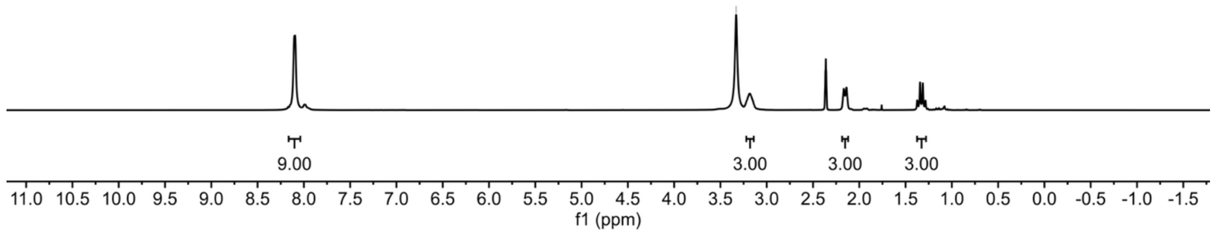

Supplementary Figure 19. ^{1}H NMR (D_6-DMSO, 298 K) spectrum of *cis*,*cis*-1,3,5-triamino-cyclohexane•3HBr.

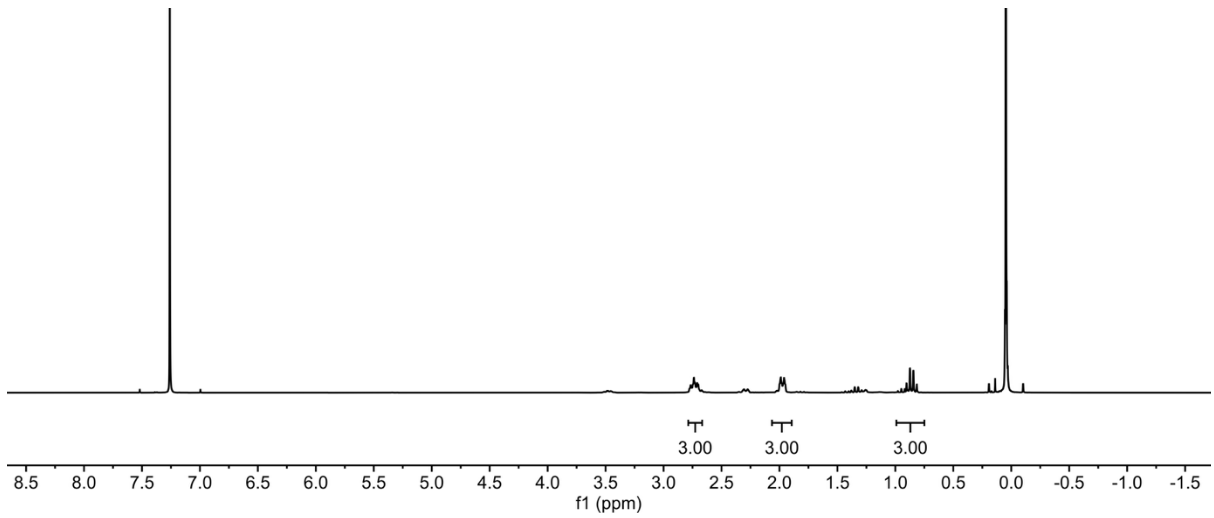

Supplementary Figure 20. ^{1}H NMR ($CDCl_3$, 298 K) spectrum of *cis*,*cis*-1,3,5-triamino-cyclohexane. The peak at ~0.07 ppm is residual silicone grease.

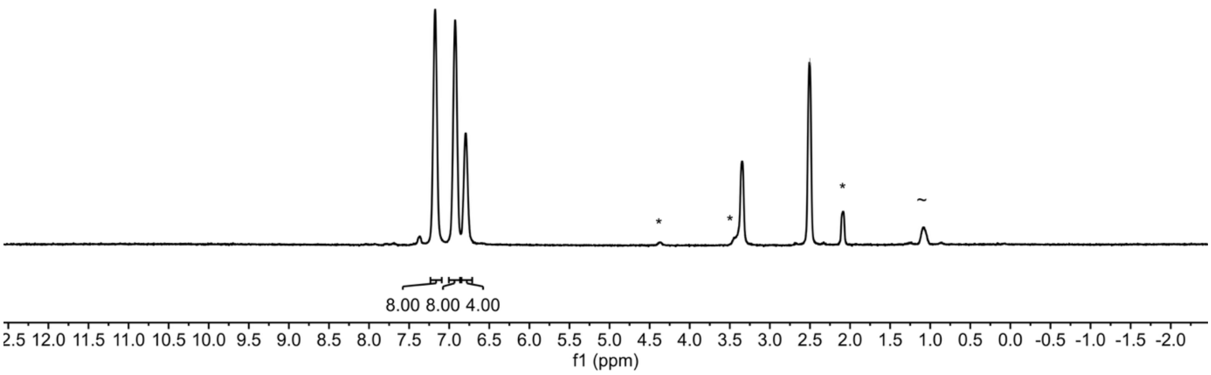

Supplementary Figure 21. ^{1}H NMR (D_6-DMSO, 298 K) spectrum of $AgBPh_4$. The asterisk (*) denotes residual EtOH; ~ = residual acetone.

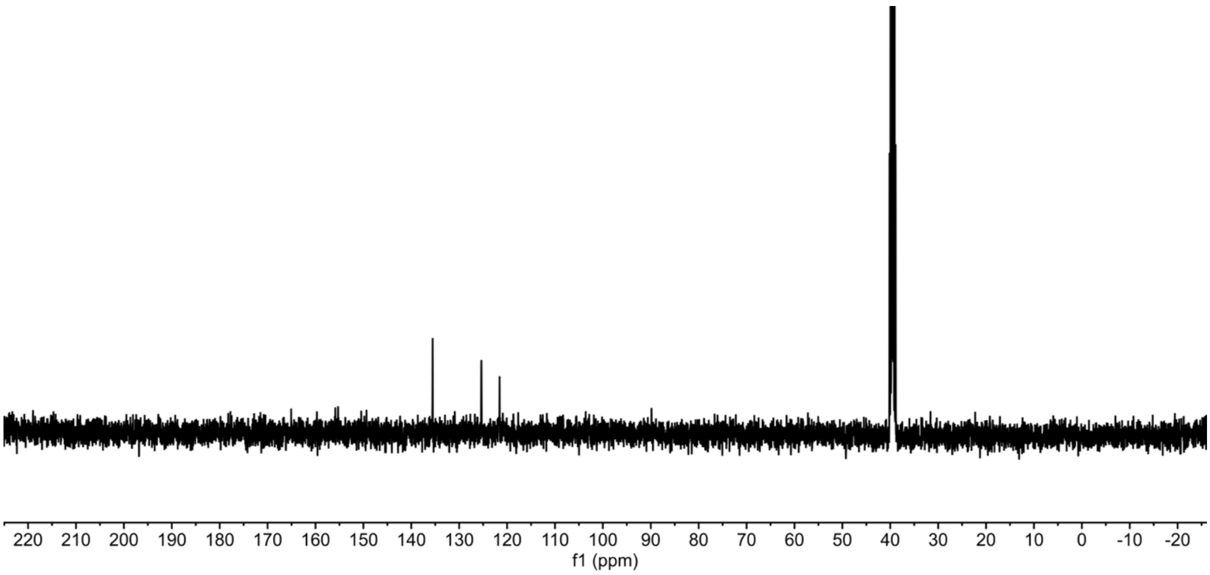

Supplementary Figure 22. $^{13}C\{^{1}H\}$ NMR (D_6-DMSO, 298 K) spectrum of $AgBPh_4$.

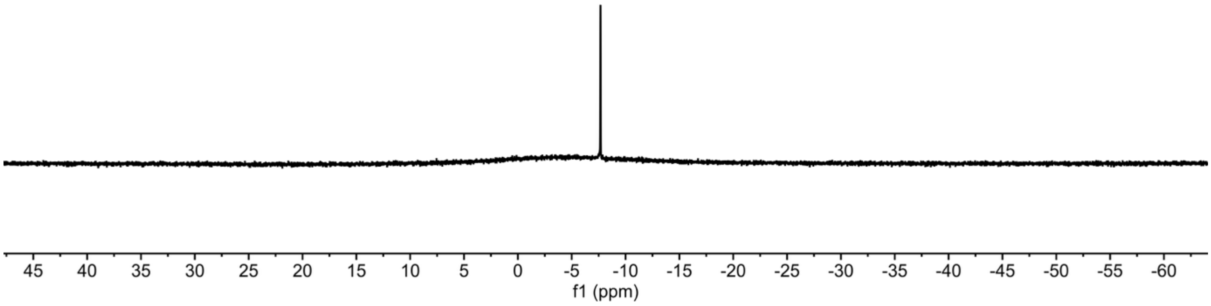

Supplementary Figure 23. ^{11}B NMR (D_6-DMSO, 298 K) spectrum of $AgBPh_4$.

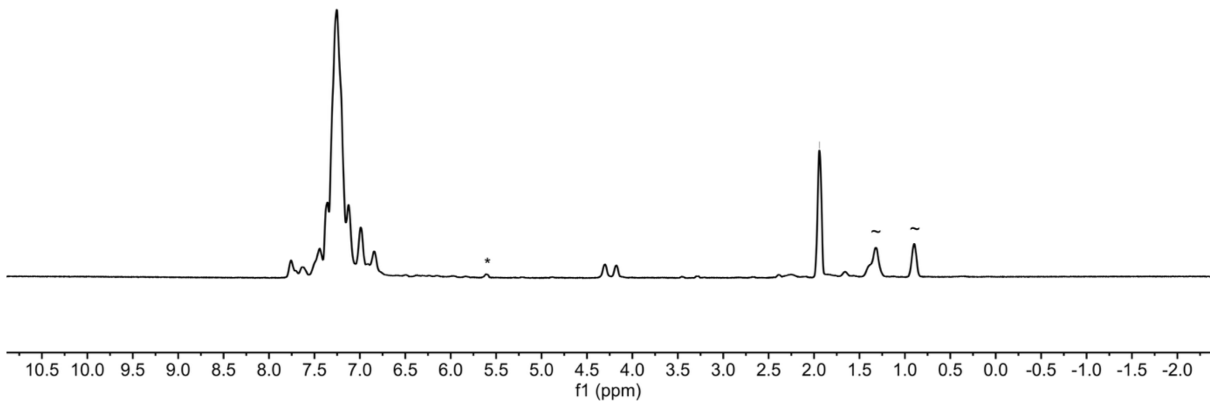

Supplementary Figure 24. ^{1}H NMR (D_3-MeCN, 298 K) spectrum of $[CPh_3][BPh_4]$. The tilde (~) denotes residual hexane; * = $HCPh_3$, a common decomposition product when using trityl cation-based reagents.

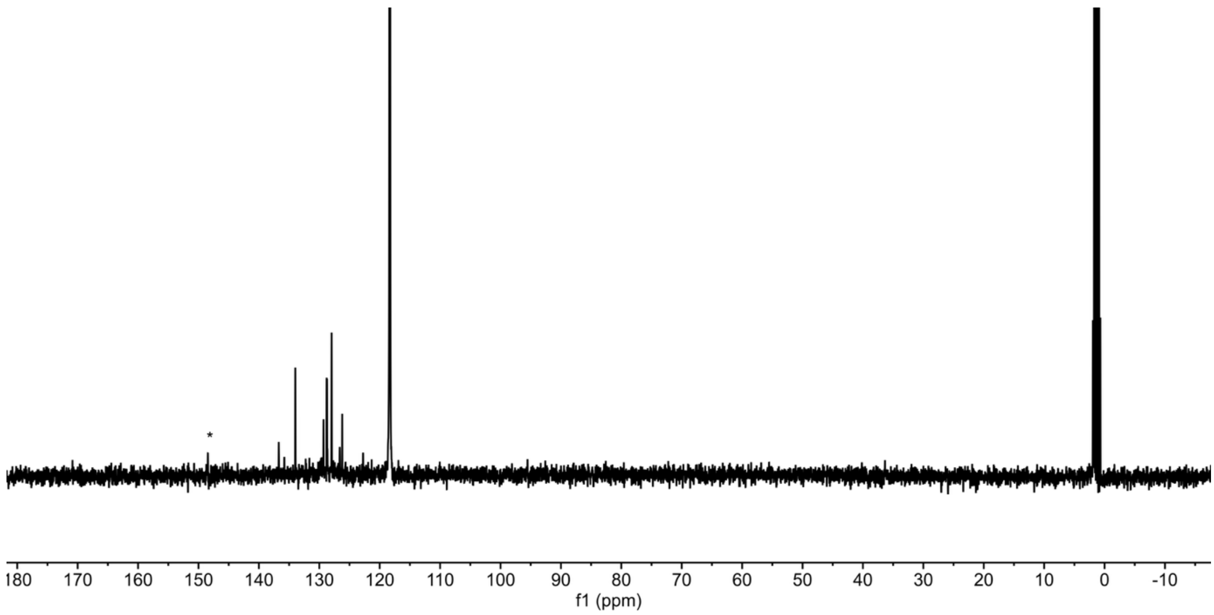

Supplementary Figure 25. $^{13}C\{^{1}H\}$ NMR (D_3-MeCN, 298 K) spectrum of $[CPh_3][BPh_4]$. The asterisk (*) denotes $HCPh_3$, a common decomposition product when using trityl cation-based reagents.

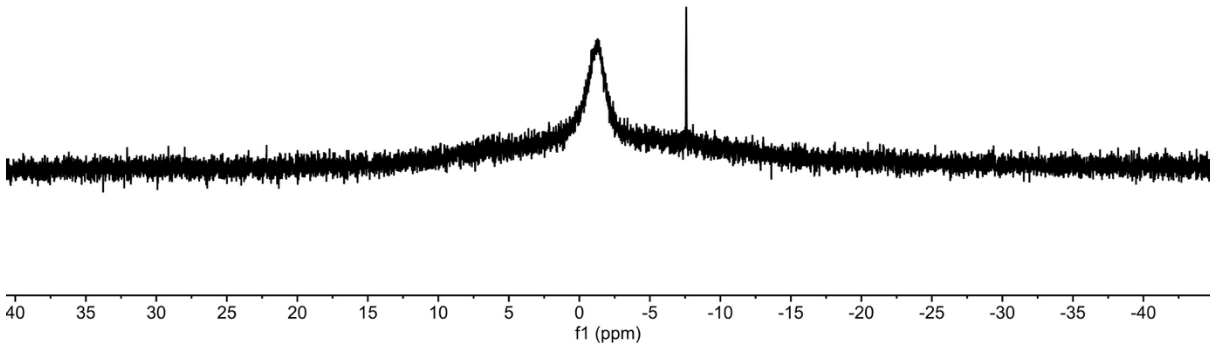

Supplementary Figure 26. ^{11}B NMR (D_3-MeCN, 298 K) spectrum of $[CPh_3][BPh_4]$.

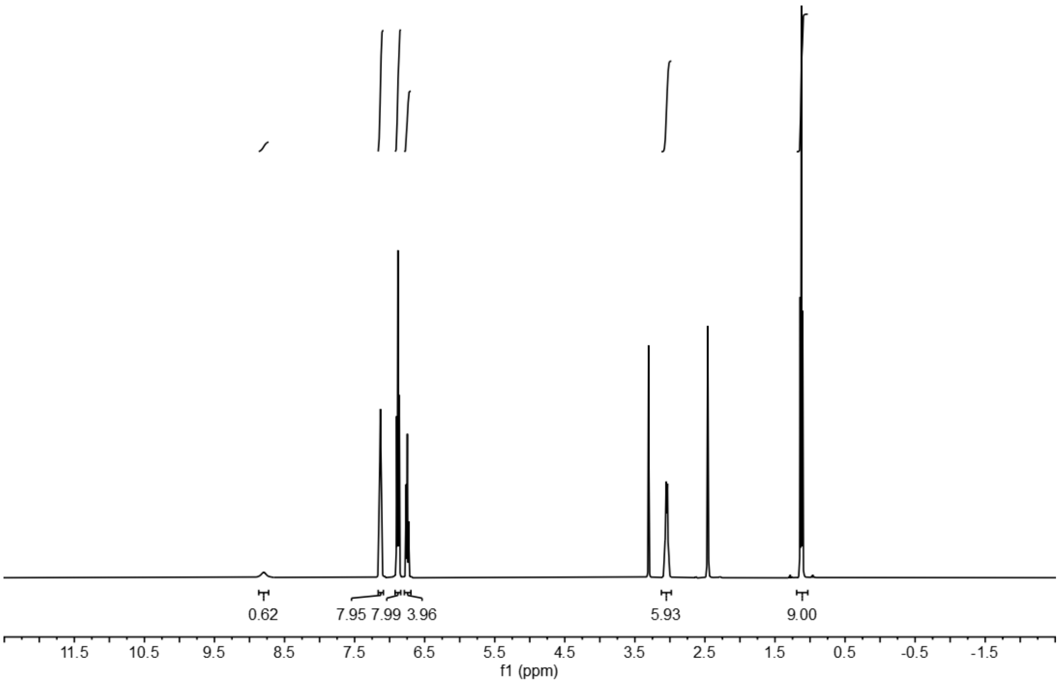

Supplementary Figure 27. ^{1}H NMR (D_6-DMSO, 298 K) spectrum of $[HNEt_3][BPh_4]$.

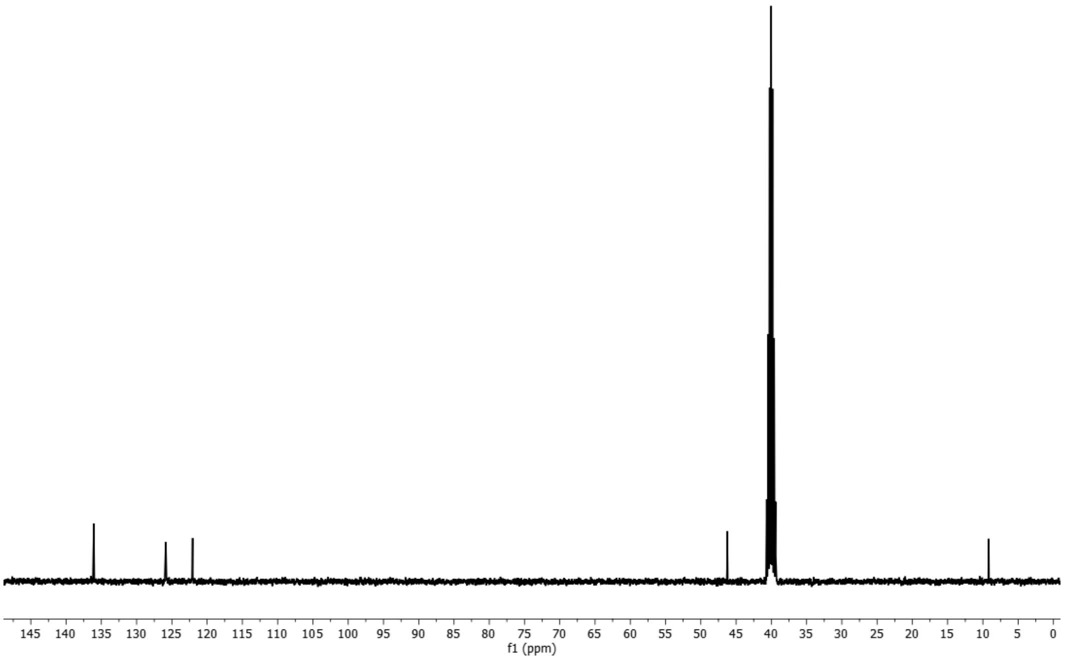

Supplementary Figure 28. $^{13}C\{^{1}H\}$ NMR (D_6-DMSO, 298 K) spectrum of $[HNEt_3][BPh_4]$.

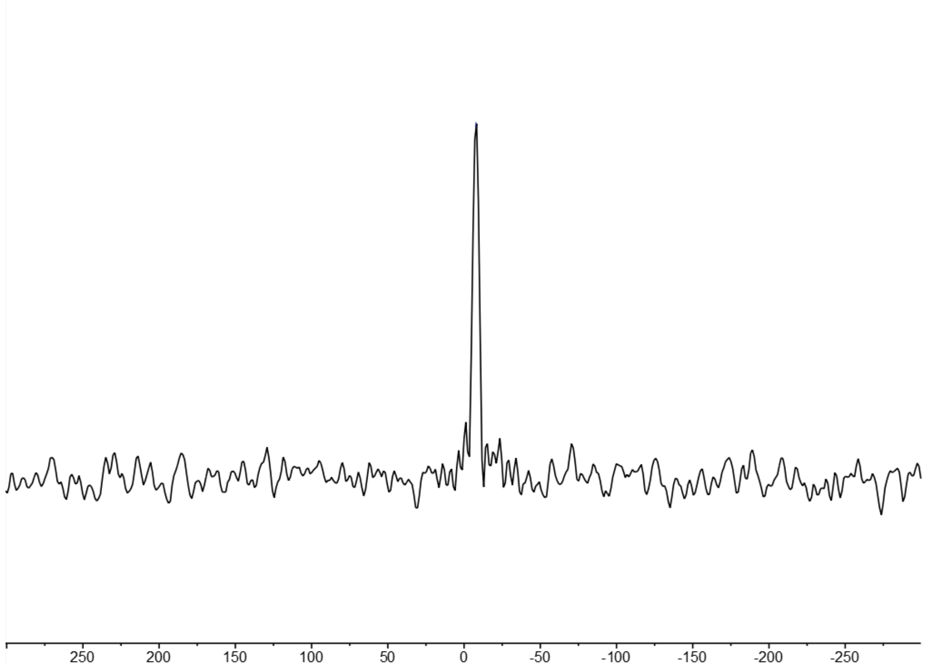

Supplementary Figure 29. ^{11}B NMR (D_6-DMSO, 298 K) spectrum of $[HNEt_3][BPh_4]$.

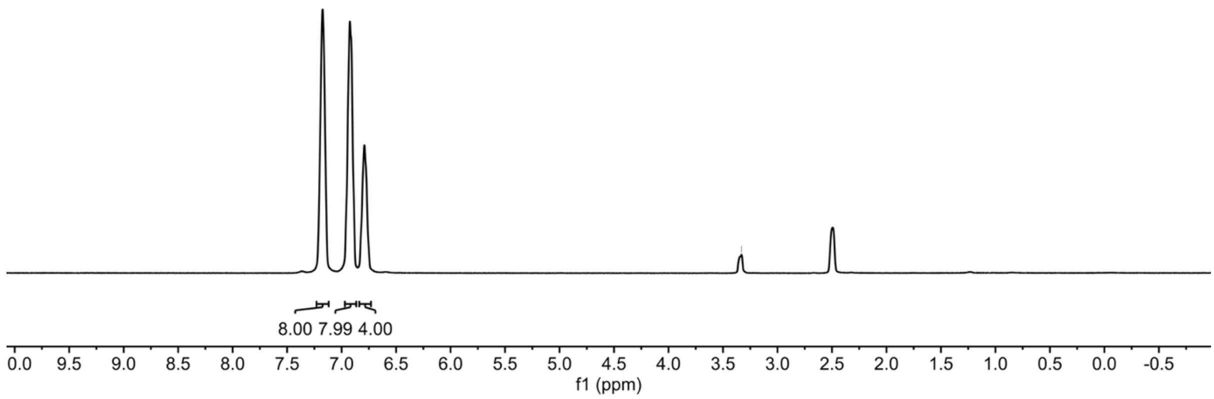

Supplementary Figure 30. ^{1}H NMR (D_6-DMSO, 298 K) spectrum of $TlBPh_4$.

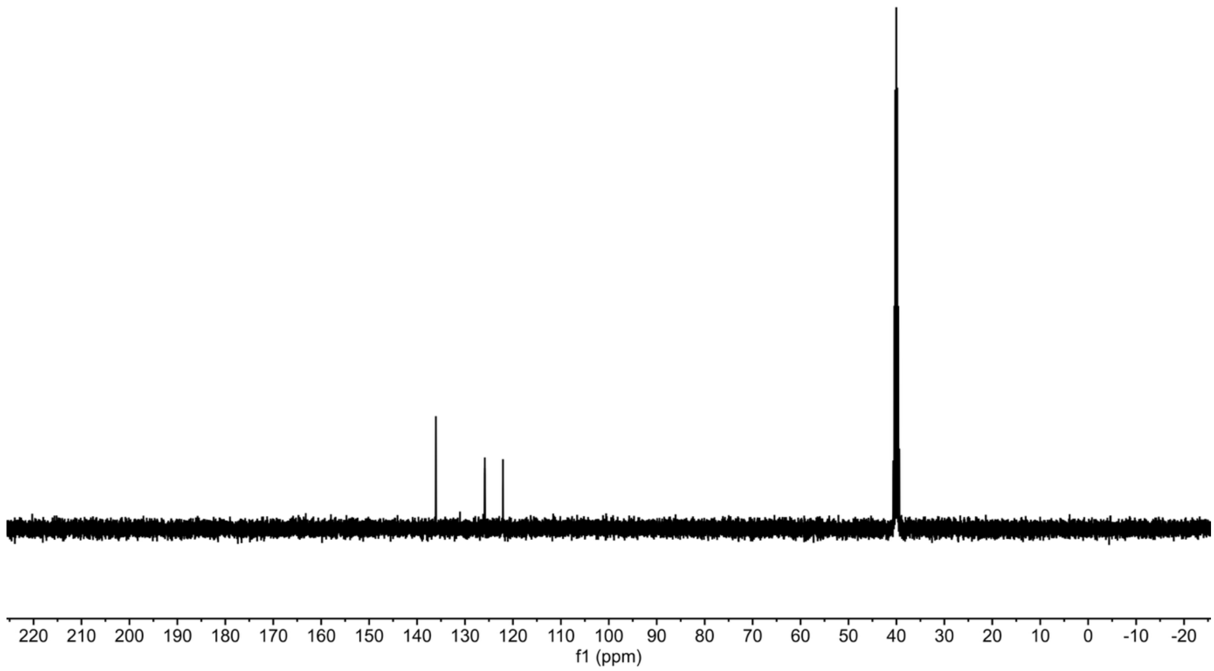

Supplementary Figure 31. $^{13}C\{^{1}H\}$ NMR (D_6-DMSO, 298 K) spectrum of $TlBPh_4$.

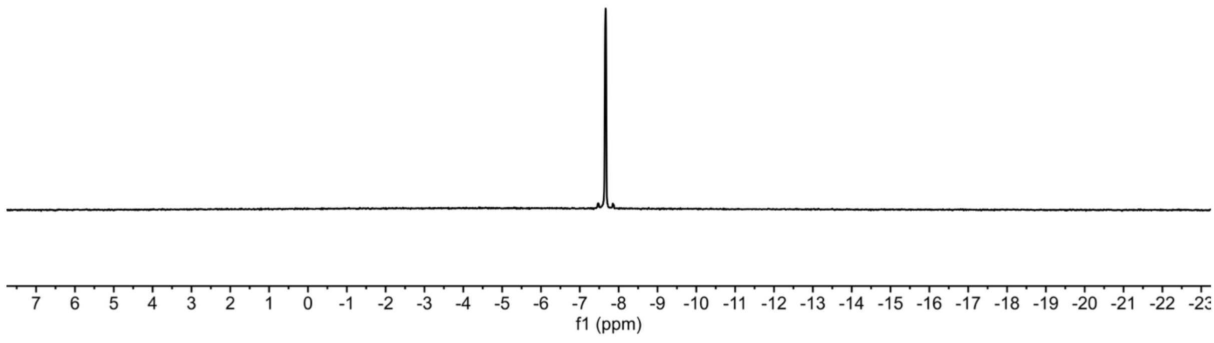

Supplementary Figure 32. ^{11}B NMR (D_6-DMSO, 298 K) spectrum of $TlBPh_4$.

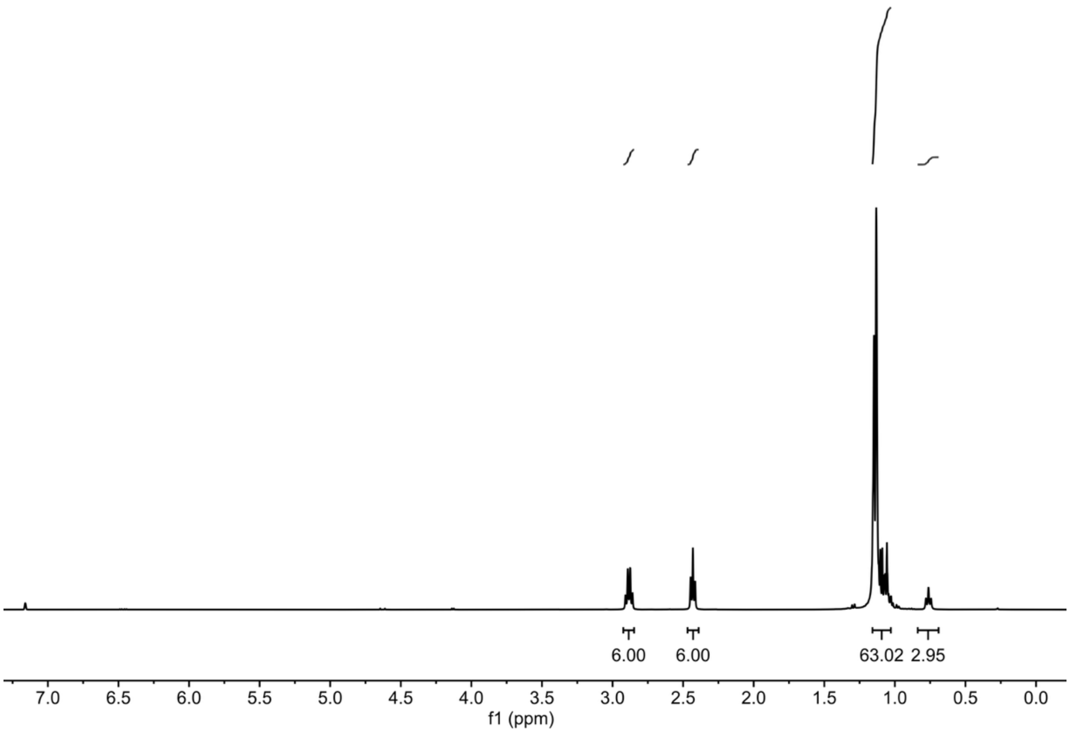

Supplementary Figure 33. ^{1}H NMR (D_6-benzene, 298 K) spectrum of $Tren^{TIPS}H_3$.

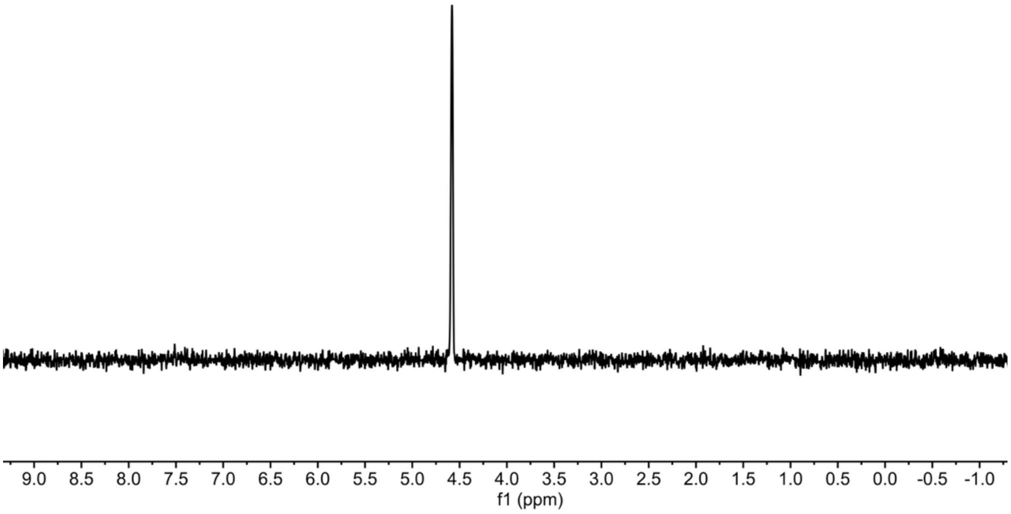

Supplementary Figure 34. ^{29}Si NMR (D_6-benzene, 298 K) spectrum of $Tren^{TIPS}H_3$.

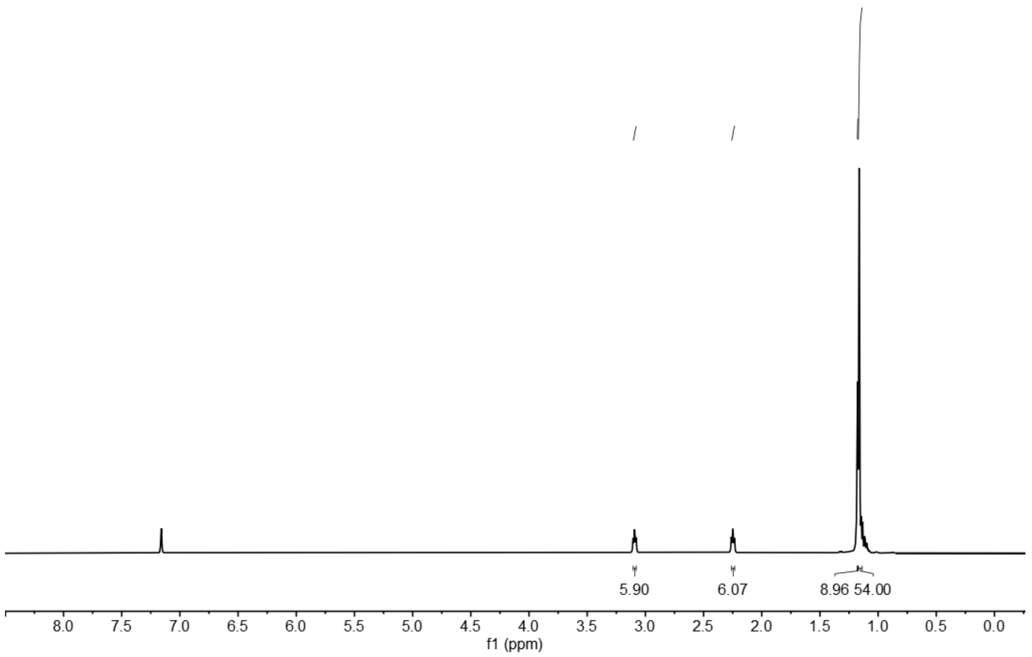

Supplementary Figure 35. ^{1}H NMR (D_6-benzene, 298 K) spectrum of $Tren^{TIPS}Li_3$.

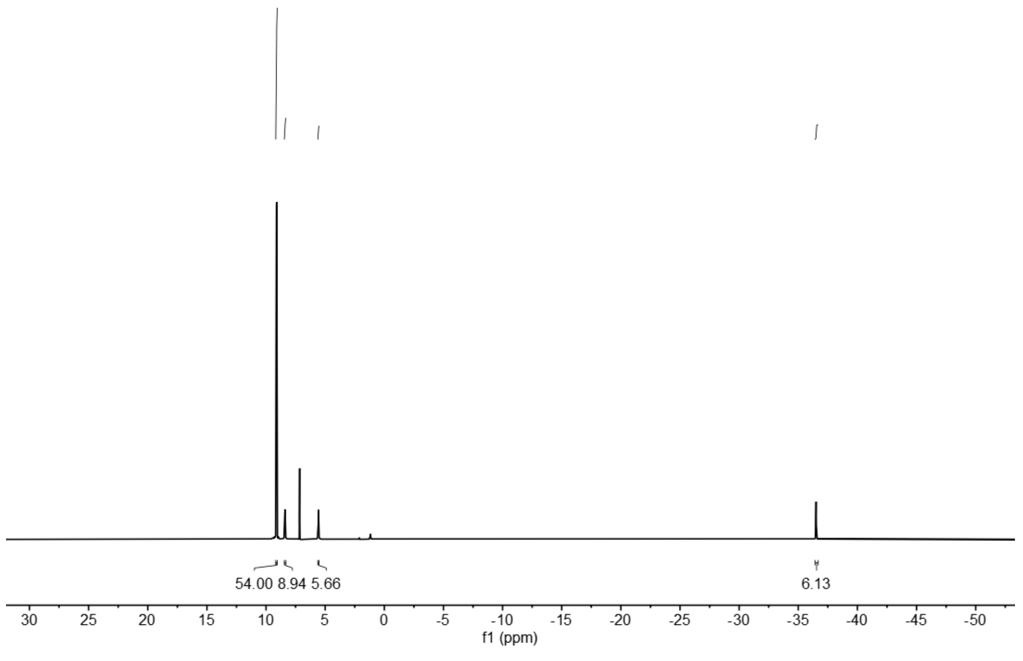

Supplementary Figure 36. ^{1}H NMR (D_6-benzene, 298 K) spectrum of $[U^{IV}(Tren^{TIPS})Cl]$.

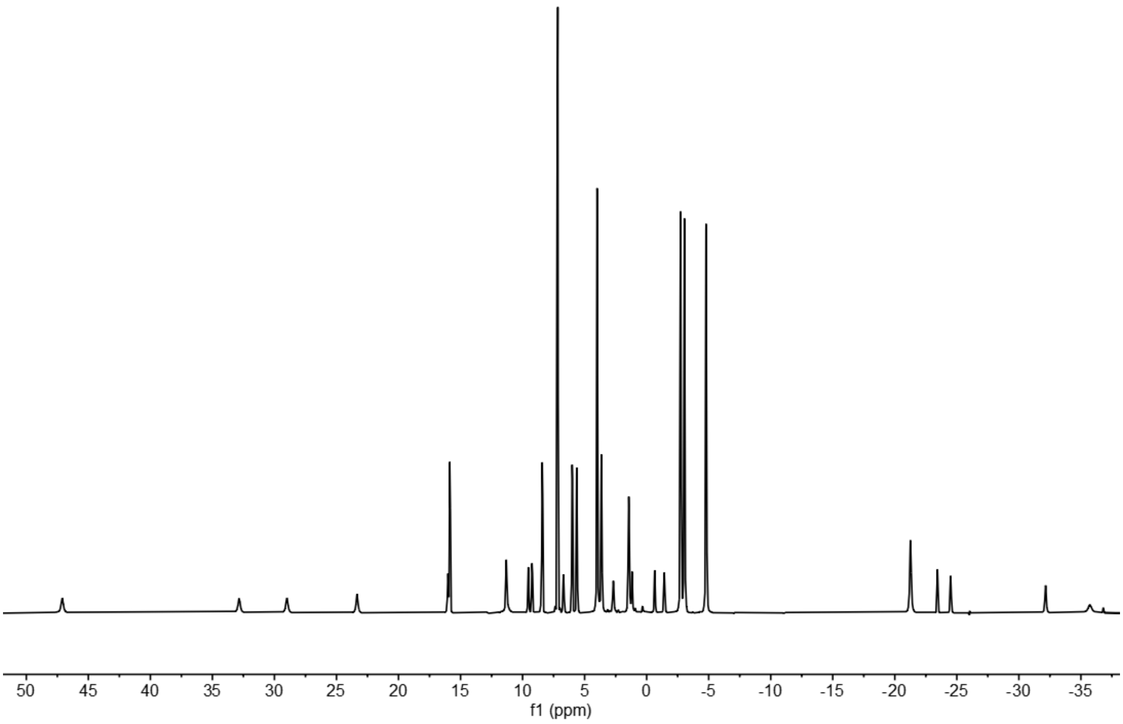

Supplementary Figure 37. ^{1}H NMR (D_6-benzene, 298 K) spectrum of [U{N($CH_2CH_2NSiPr^i{}_3$)$_2$($CH_2CH_2SiPr^i{}_2CHMeCH_2$)}].

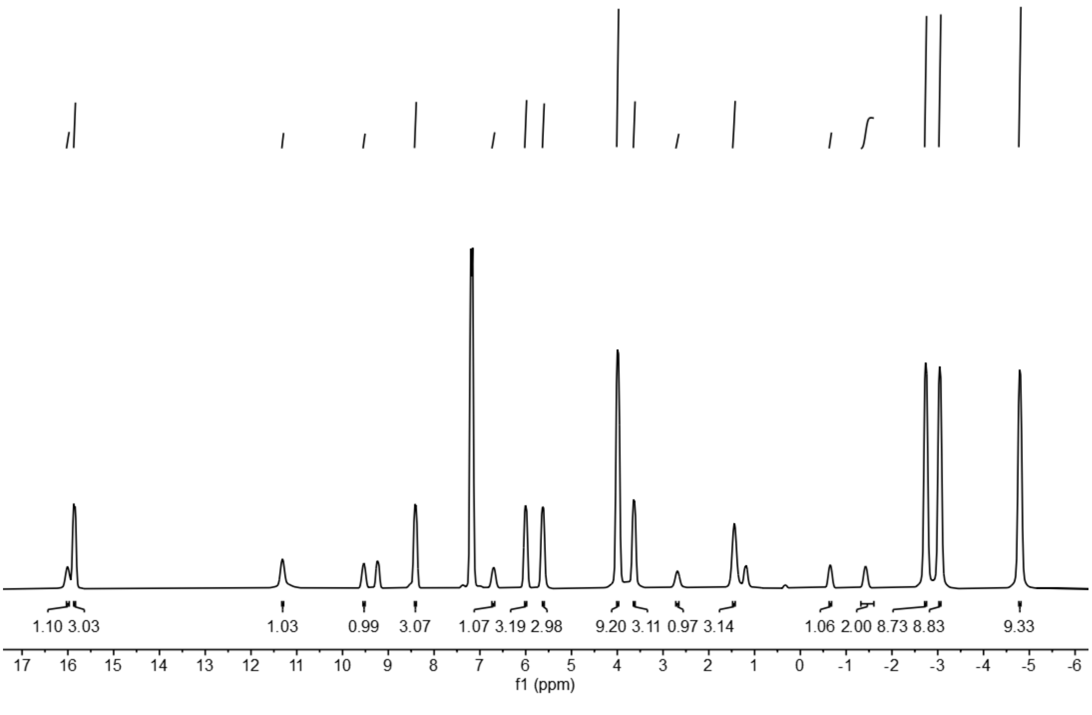

Supplementary Figure 38. Zoom-in of the ^{1}H NMR (C_6D_6, 298 K) spectrum of [U{N($CH_2CH_2NSiPr^i{}_3$)$_2$($CH_2CH_2SiPr^i{}_2CHMeCH_2$)}].

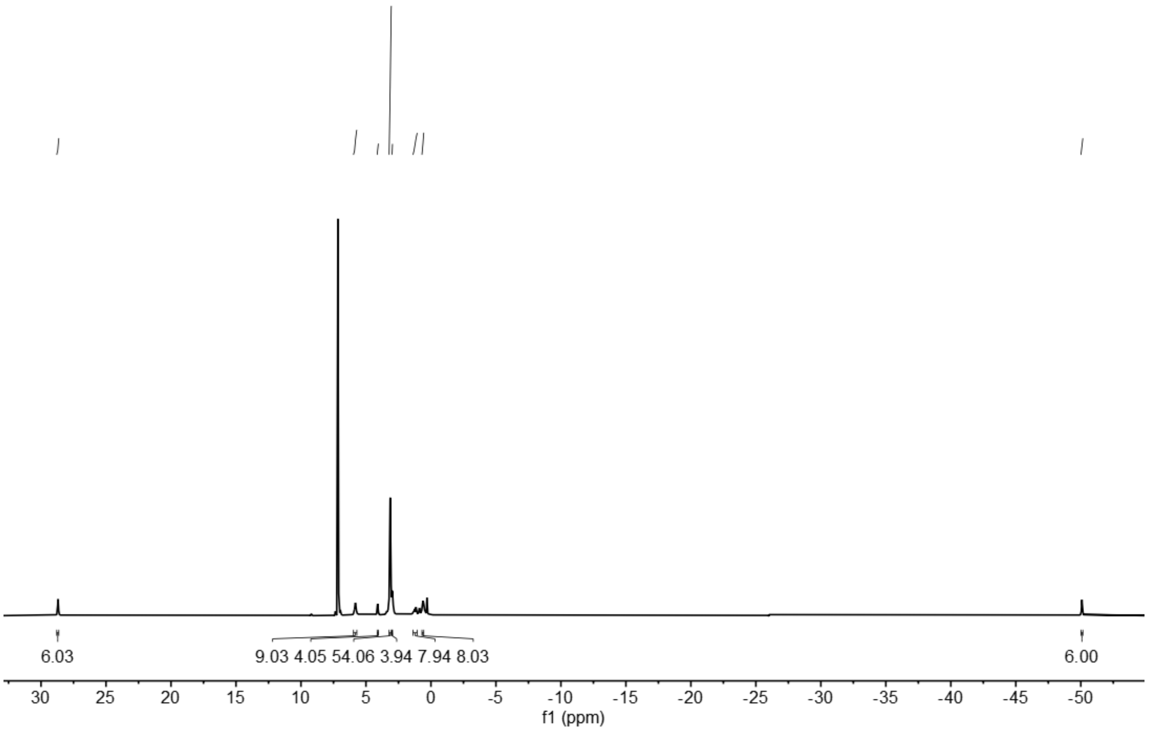

Supplementary Figure 39. ^{1}H NMR (D_6-benzene, 298 K) spectrum of $[U^{IV}(Tren^{TIPS})(THF)][BPh_4]$.

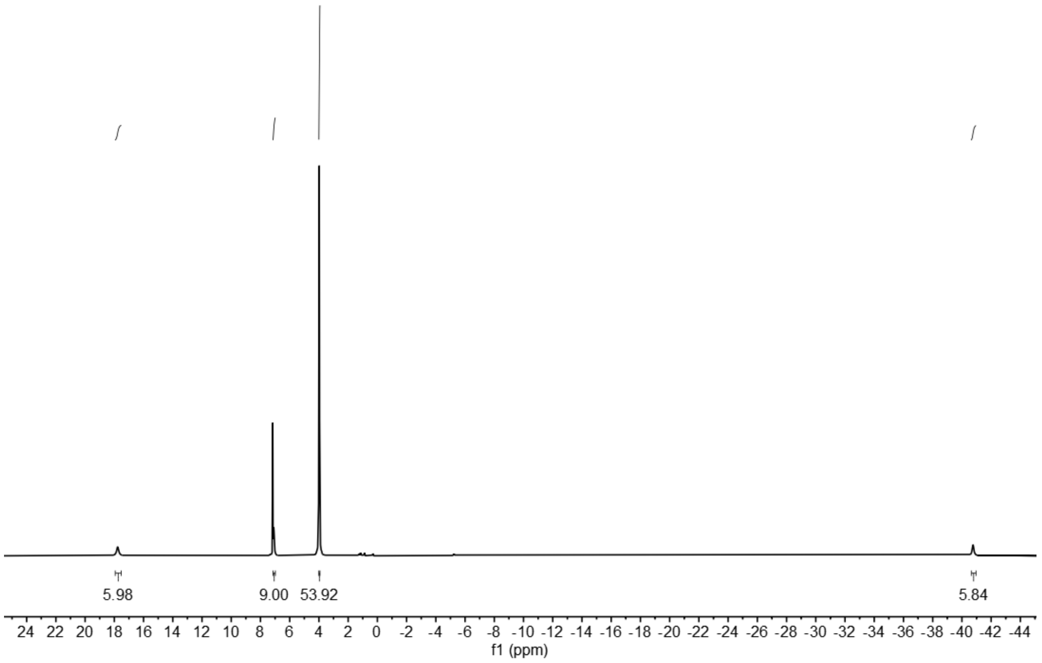

Supplementary Figure 40. ^{1}H NMR (D_6-benzene, 298 K) spectrum of **1**.

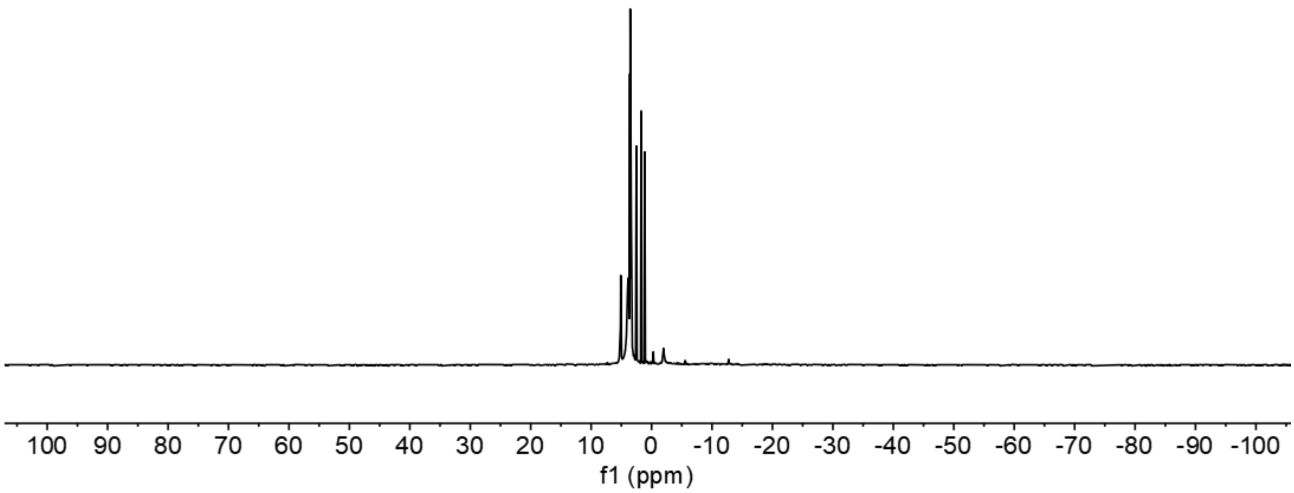

Supplementary Figure 41. ^{1}H NMR (D_8-THF, 298 K) spectrum of 9:1 **6a**:**6b**.

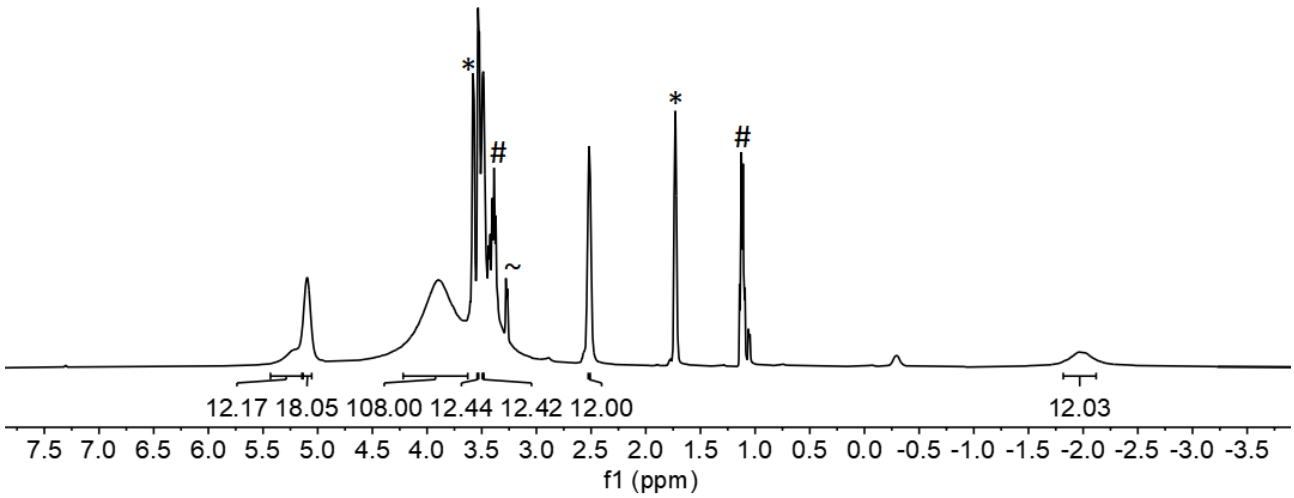

Supplementary Figure 42. Zoom-in of the ^{1}H NMR (D_8-THF, 298 K) spectrum of 9:1 **6a**:**6b**. The asterisks (*) at ~ 3.58 and 1.72 ppm denote THF solvent. # = residual Et_2O; ~ = residual DME from its use as a crystallisation solvent.

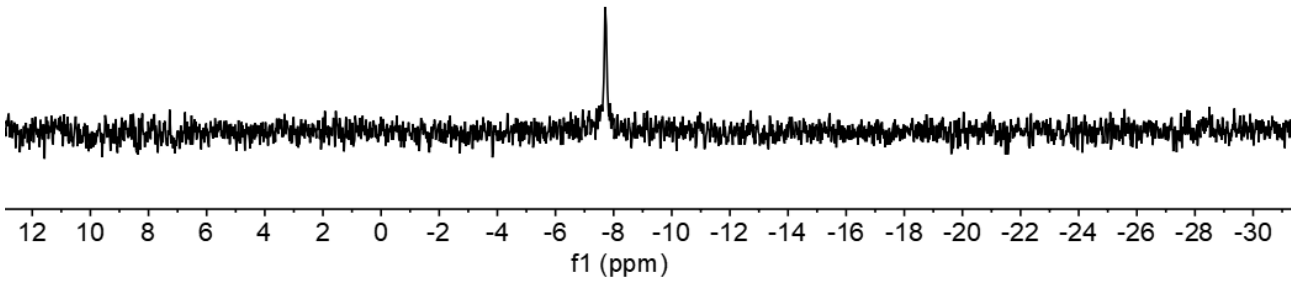

Supplementary Figure 43. $^{29}Si\{^{1}H\}$ NMR (D_8-THF, 298 K) spectrum of 9:1 **6a**:**6b**.

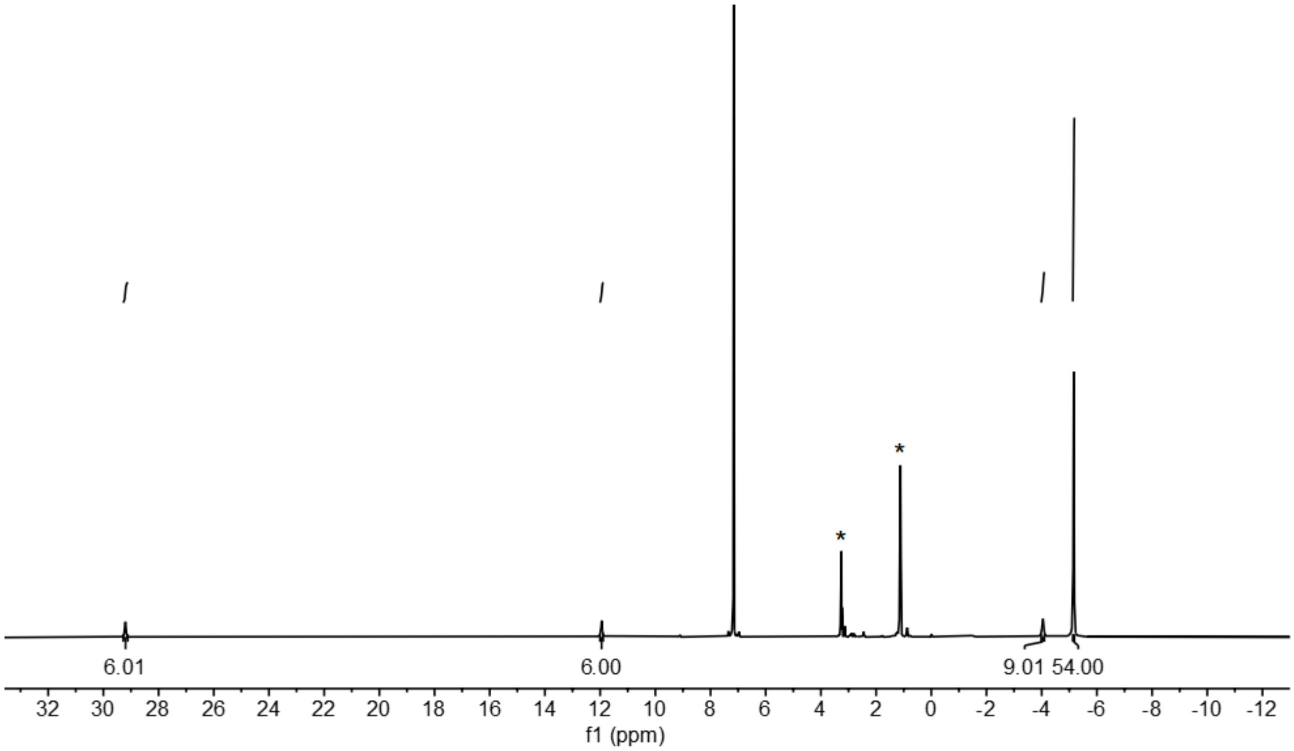

Supplementary Figure 44. ^{1}H NMR (D_8-THF, 298 K) spectrum of **9**. The asterisk (*) at ~3.30 and 1.10 ppm denotes trace Et_2O.

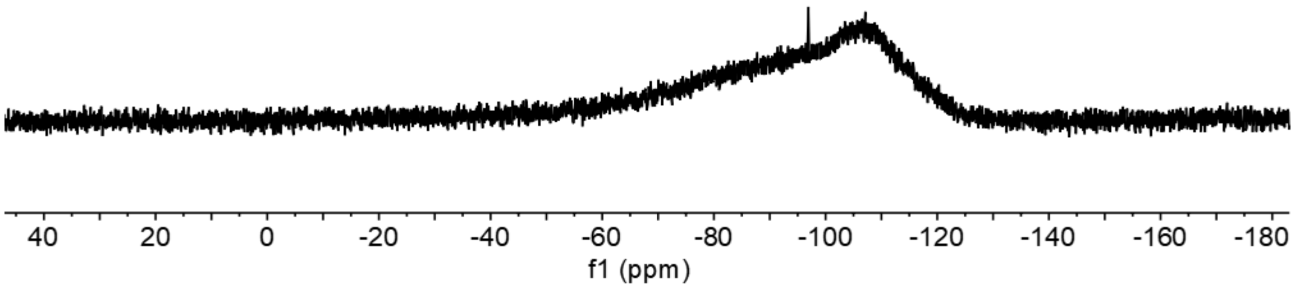

Supplementary Figure 45. $^{29}Si\{^1H\}$ NMR (D_8-THF, 298 K) spectrum of **9**.

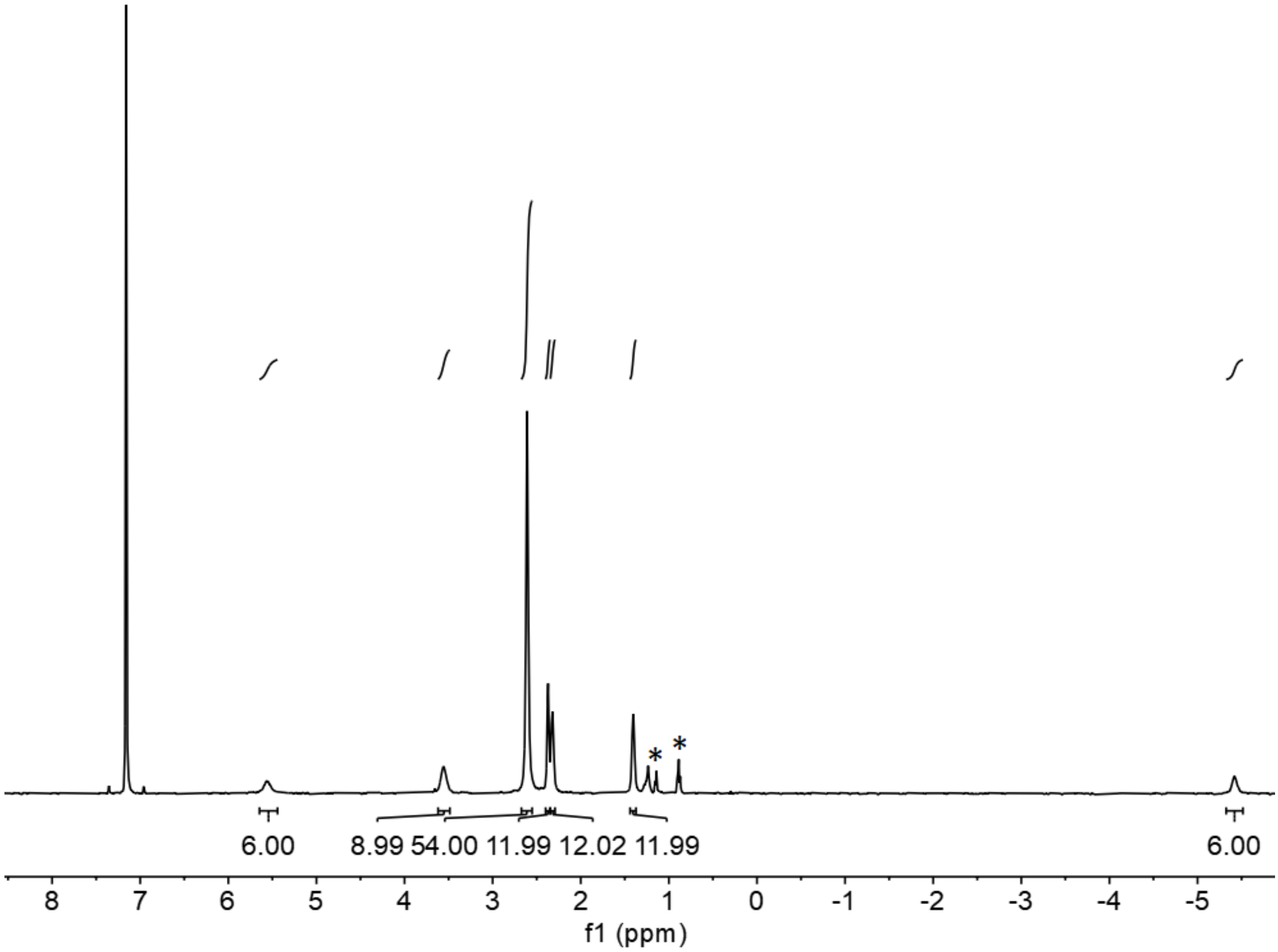

Supplementary Figure 46. ^1H NMR (D_6-benzene, 298 K) spectrum of **12**. The asterisk (*) at ~1.24 and 0.89 ppm denotes trace hexane.

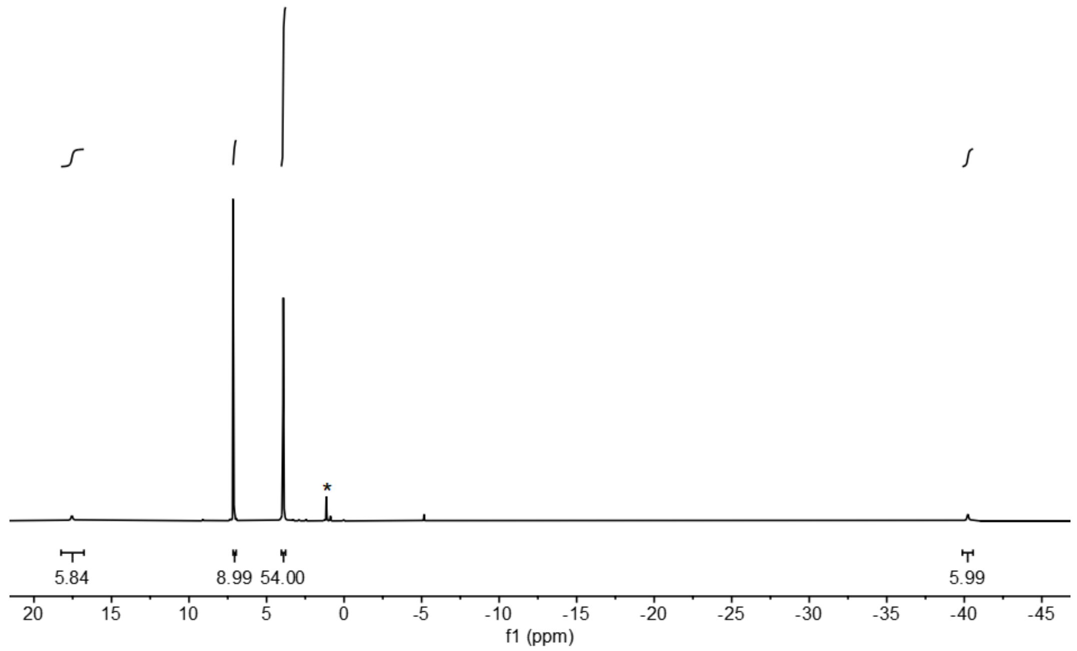

Supplementary Figure 47. ^{1}H NMR (D_6-benzene, 298 K) spectrum of the reaction mixture when using pentane as the reaction solvent to attempt the synthesis of **6a**. Integrations represent the four ^{1}H environments of Tren^{TIPS}U: δ 17.55 (6H, s, C*H*$_2$), 7.09 (9H, s, Pr^{i}-C*H*), 3.92 (54H, s, Pr^{i}-C*H*$_3$), −40.23 (6H, s, C*H*$_2$) ppm. The asterisk (*) at ~1.10 ppm denotes Tren^{TIPS}H$_3$.

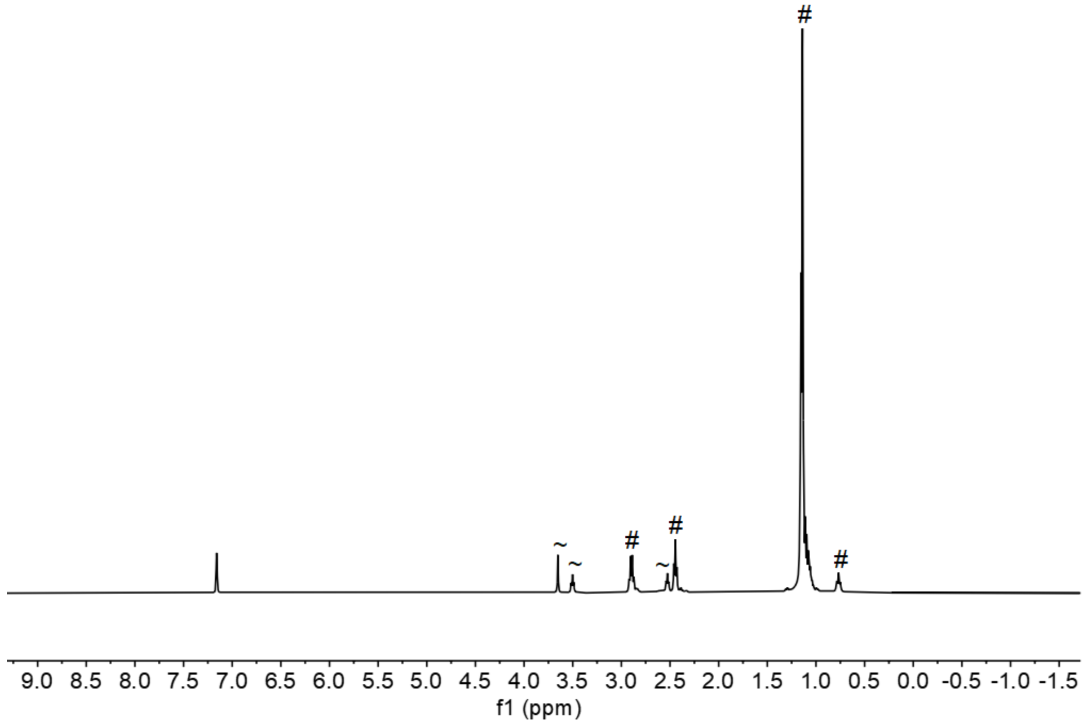

Supplementary Figure 48. ^{1}H NMR (D_6-benzene, 298 K) spectrum of the reaction mixture when using THF as the reaction solvent to attempt the synthesis of **6a**. The tilde (~) at ~3.60, 3.50, and 2.53 ppm denotes 2.2.2-cryptand; # = Tren^{TIPS}H$_3$.

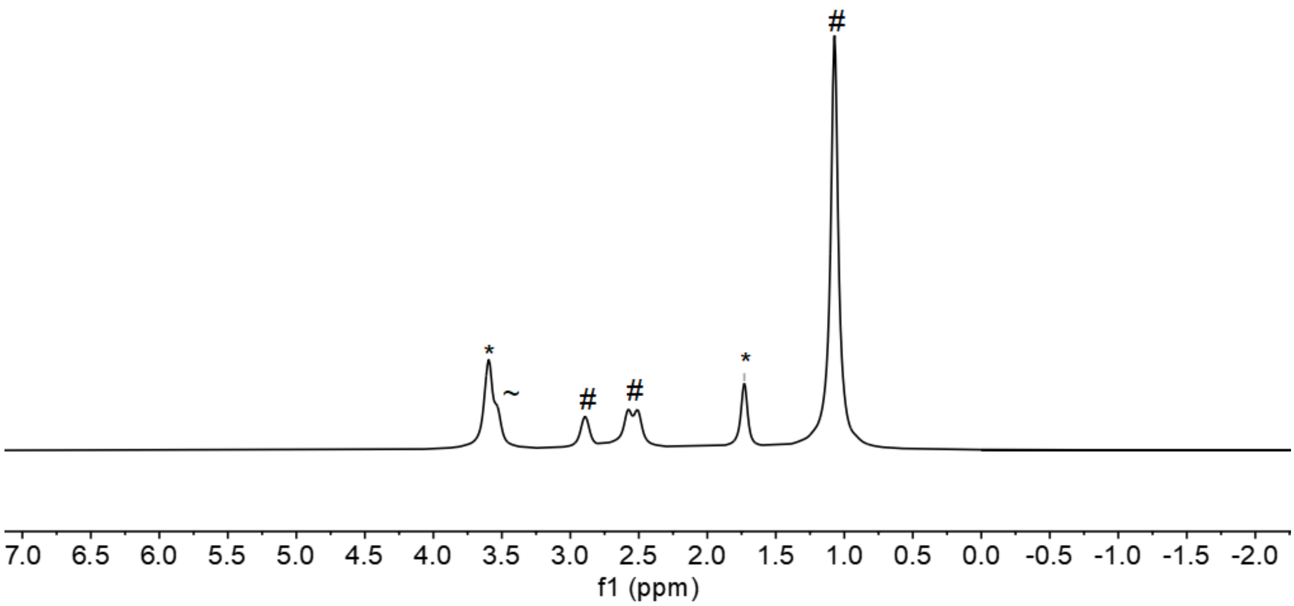

Supplementary Figure 49. ^{1}H NMR (D_8-THF, 298 K) spectrum of the reaction mixture when using THF as the reaction solvent to attempt the synthesis of **6a**. The tilde (~) denotes 2.2.2-cryptand; # = Tren^{TIPS}H_3; * = THF solvent.

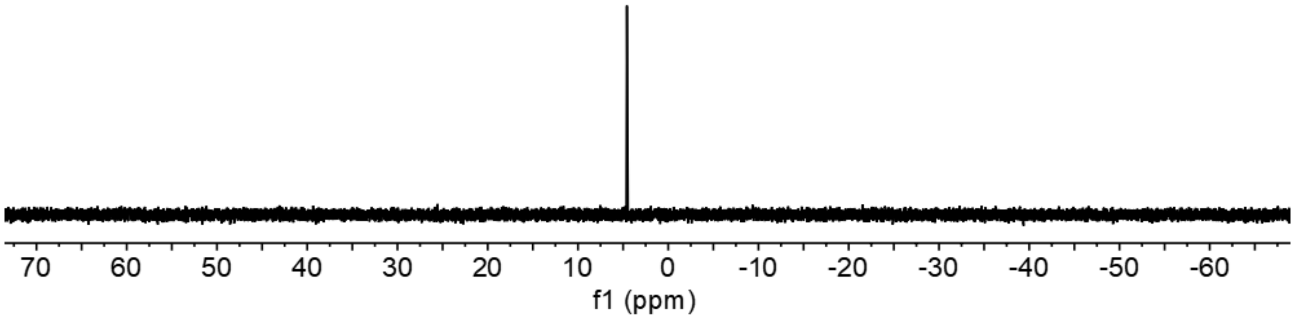

Supplementary Figure 50. ^{29}Si{^{1}H} NMR (D_8-THF, 298 K) spectrum of the reaction mixture when using THF as the reaction solvent to attempt the synthesis of **6a** showing the presence of Tren^{TIPS}H_3 at ~4.60 ppm.

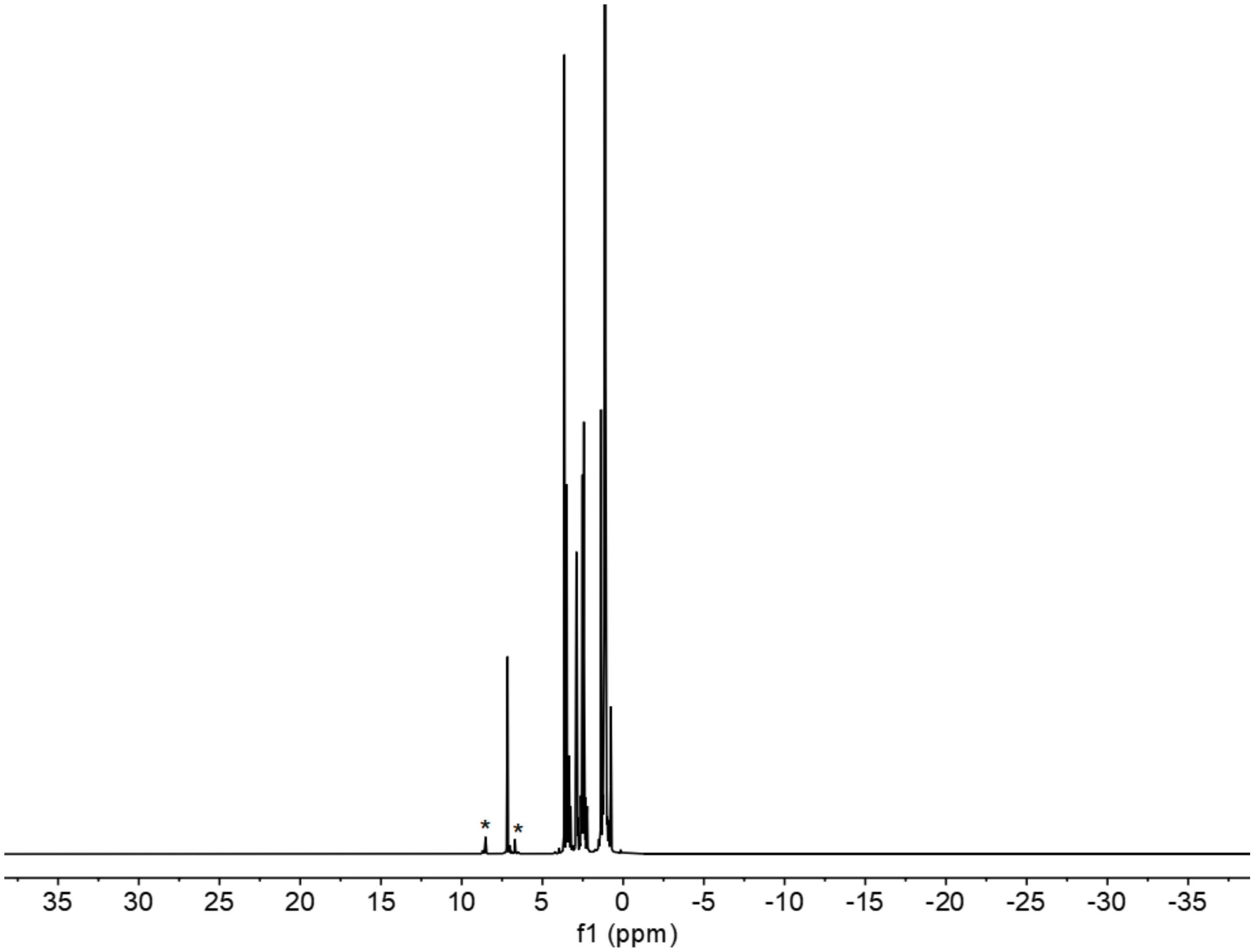

Supplementary Figure 51. ^{1}H NMR (D_6-benzene, 298 K) spectrum of the reaction mixture when using pyridine as the reaction solvent to attempt the synthesis of **6a**. The asterisks (*) at ~8.52, 7.01, and 6.69 ppm denotes trace pyridine.

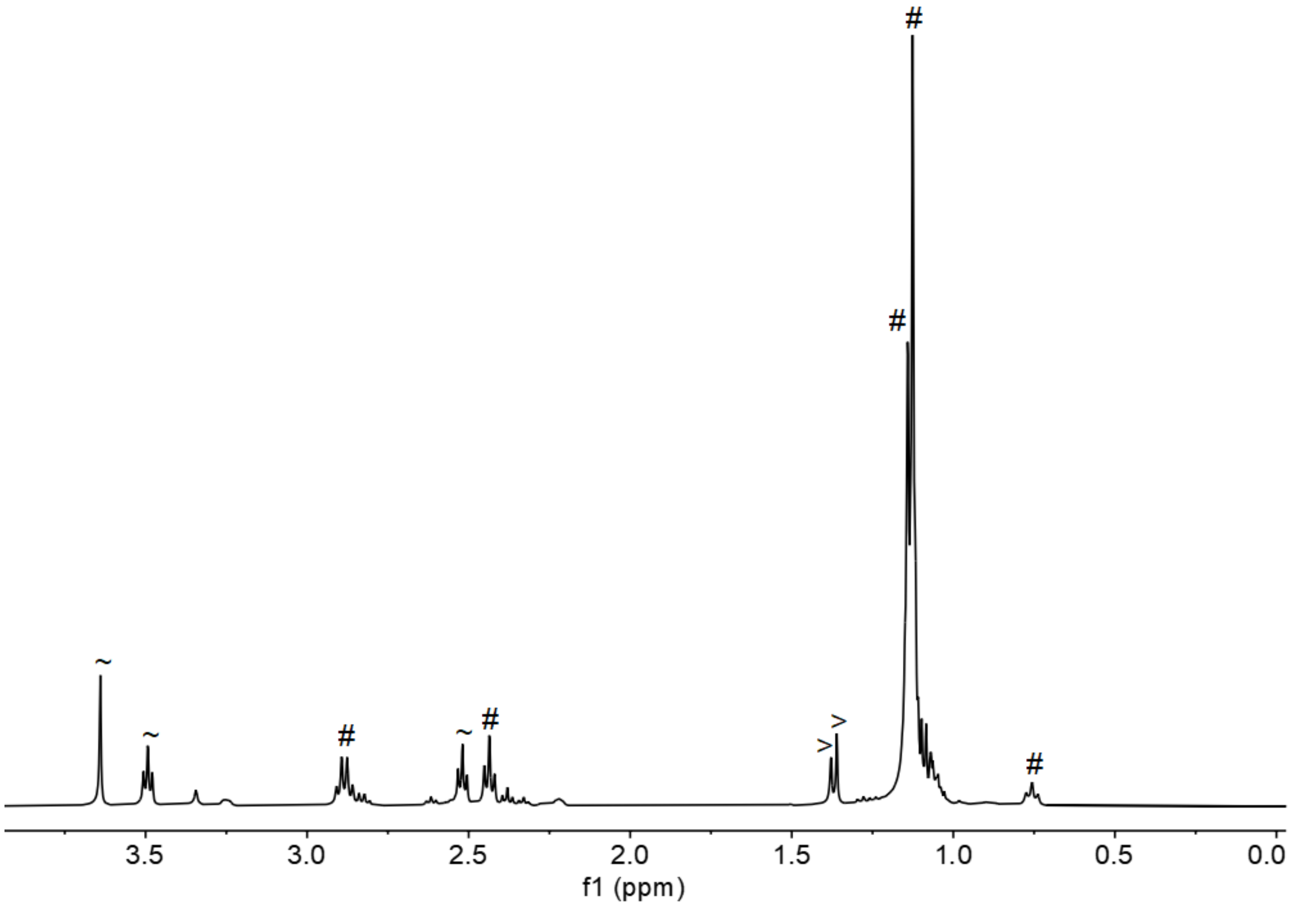

Supplementary Figure 52. Zoom-in of ^{1}H NMR (D_6-benzene, 298 K) spectrum of the reaction mixture when using pyridine as the reaction solvent to attempt the synthesis of **6a**. The tilde (~) at at ~3.60, 3.50, and 2.53 ppm denotes 2.2.2-cryptand; # = Tren^{TIPS}H_3; > = unknown pyridine reduction product.

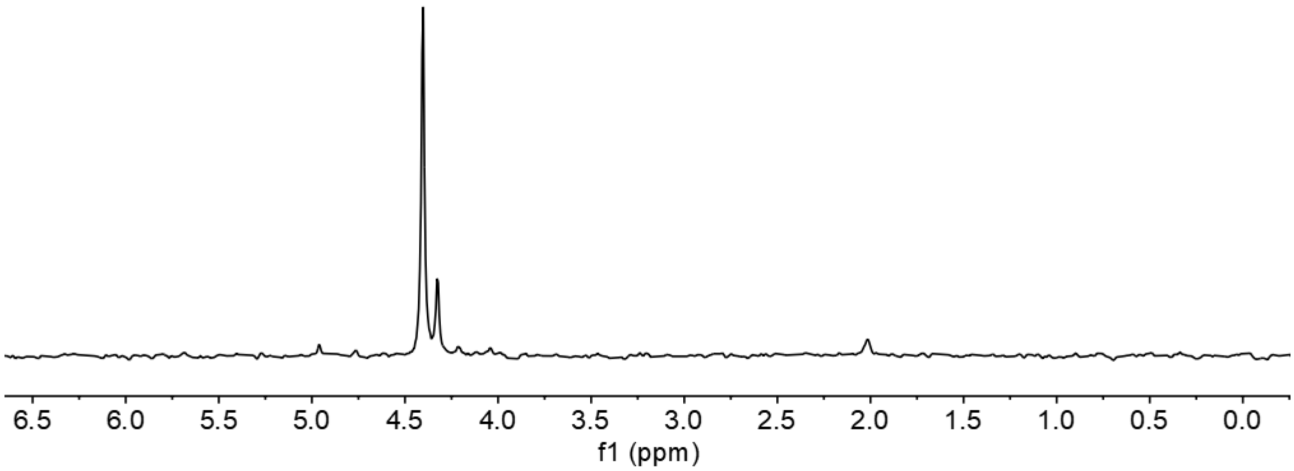

Supplementary Figure 53. ^{29}Si{^{1}H} NMR (D_6-benzene, 298 K) spectrum of the reaction mixture when using pyridine as the reaction solvent to attempt the synthesis of **6a** showing the presence of Tren^{TIPS}H_3 at ~4.40 ppm.

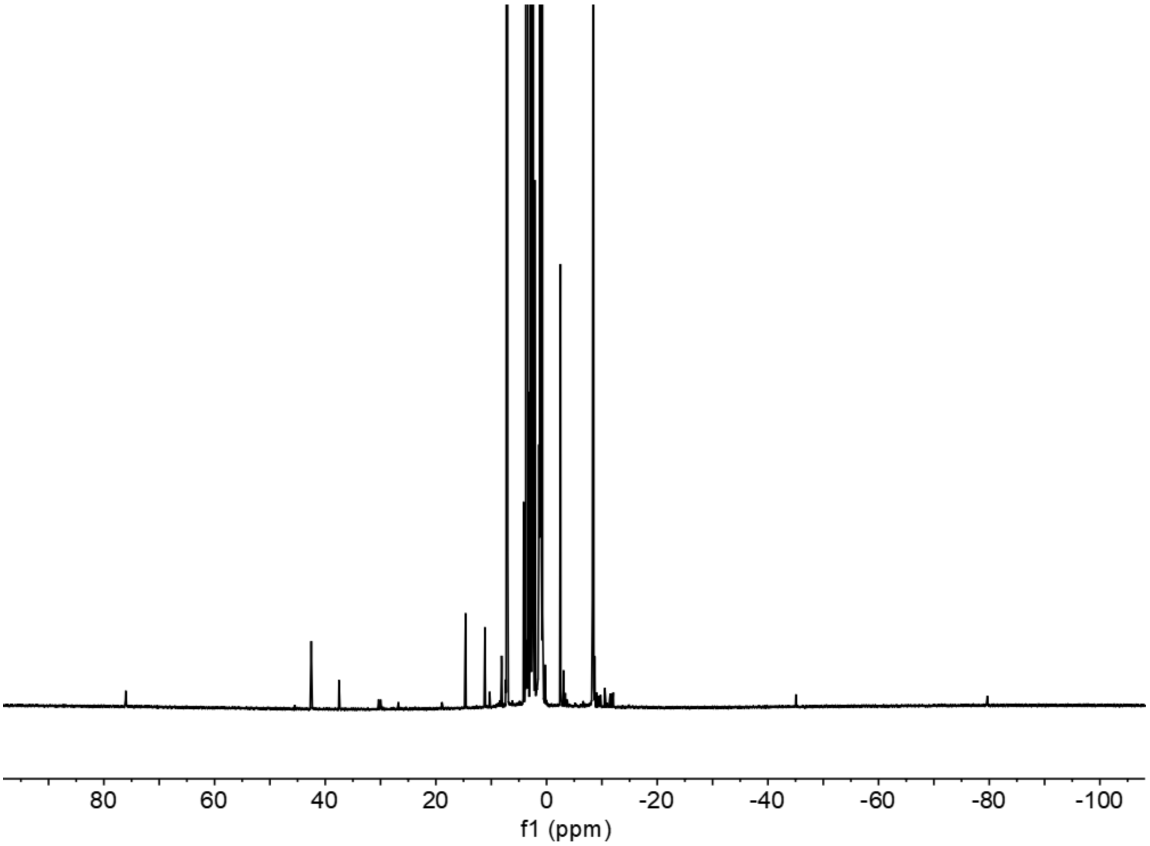

Supplementary Figure 54. ^{1}H NMR (D_6-benzene, 298 K) spectrum of the crude reaction mixture resulting from the reaction of **6a/6b** with 10% $AgBPh_4$.

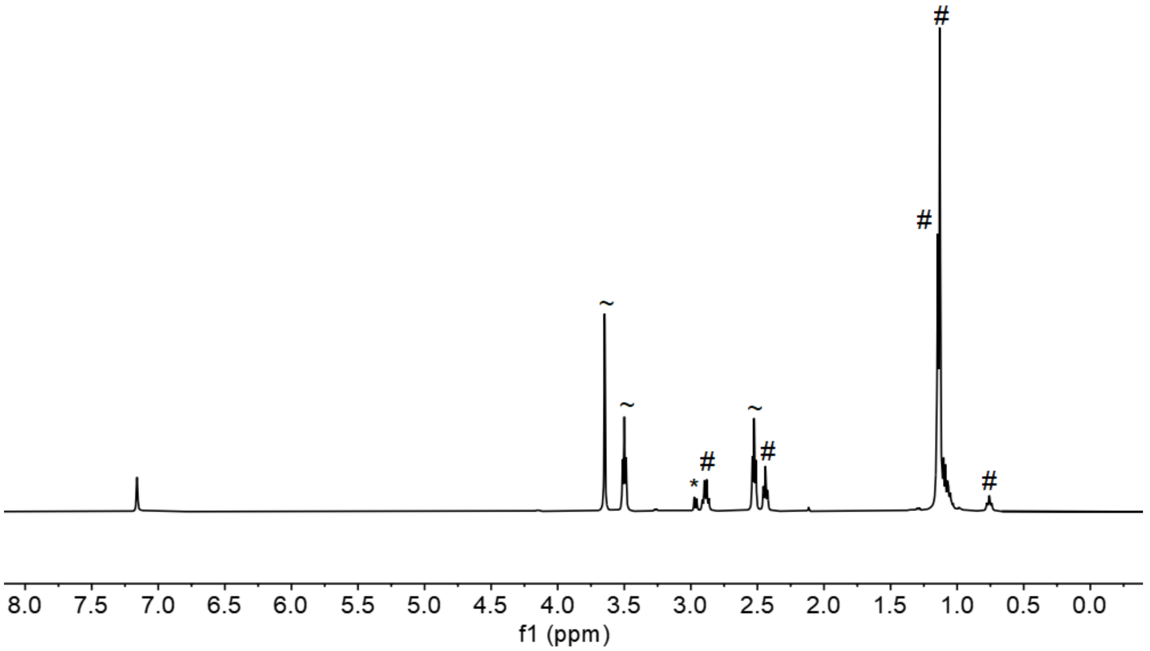

Supplementary Figure 55. Zoom-in of the ^{1}H NMR (D_6-benzene, 298 K) spectrum of the crude reaction mixture resulting from the reaction of **6a/6b** with 10% $AgBPh_4$. The tilde (~) at ~3.60, 3.50, and 2.53 ppm denotes 2.2.2-cryptand; # = $Tren^{TIPS}H_3$; * = trace Et_2O.

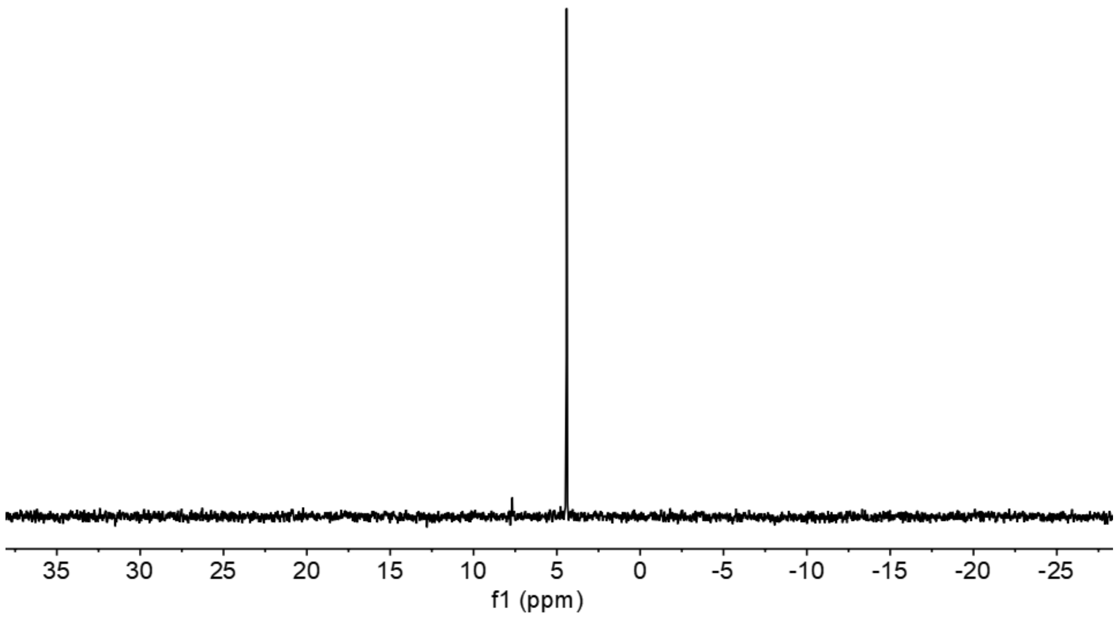

Supplementary Figure 56. $^{29}Si\{^1H\}$ NMR (D_6-benzene, 298 K) spectrum of the crude reaction mixture resulting from the reaction of **6a/6b** with 10% $AgBPh_4$ showing the presence of $Tren^{TIPS}H_3$ at ~4.40 ppm.

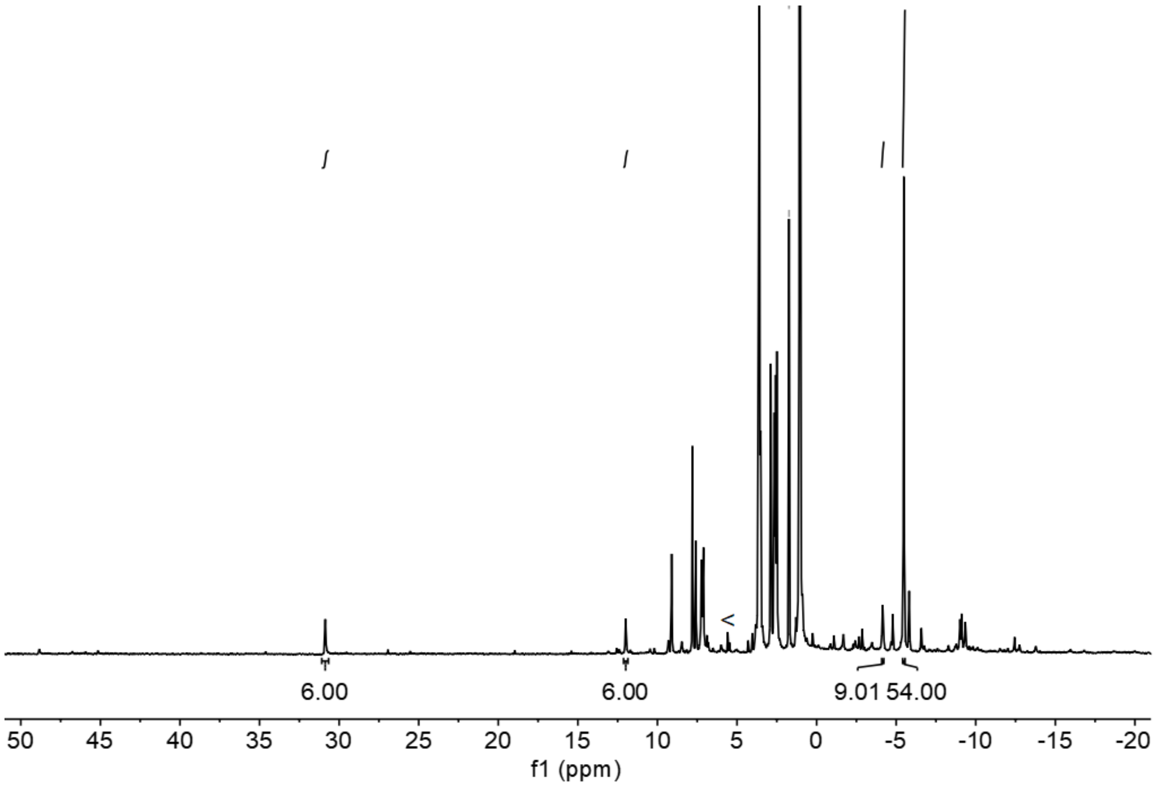

Supplementary Figure 57. ^1H NMR (D_8-THF, 298 K) spectrum of the crude reaction mixture resulting from the reaction of **6a/6b** with 10% $[CPh_3][BPh_4]$. Integrations represent the four ^1H environments of **9**: δ 29.20 (6H, s, CH_2), 11.94 (6H, s, CH_2), –4.04 (9H, s, Pr^i-CH), –5.16 (54H, s, Pr^i-CH_3) ppm. < = $HCPh_3$.

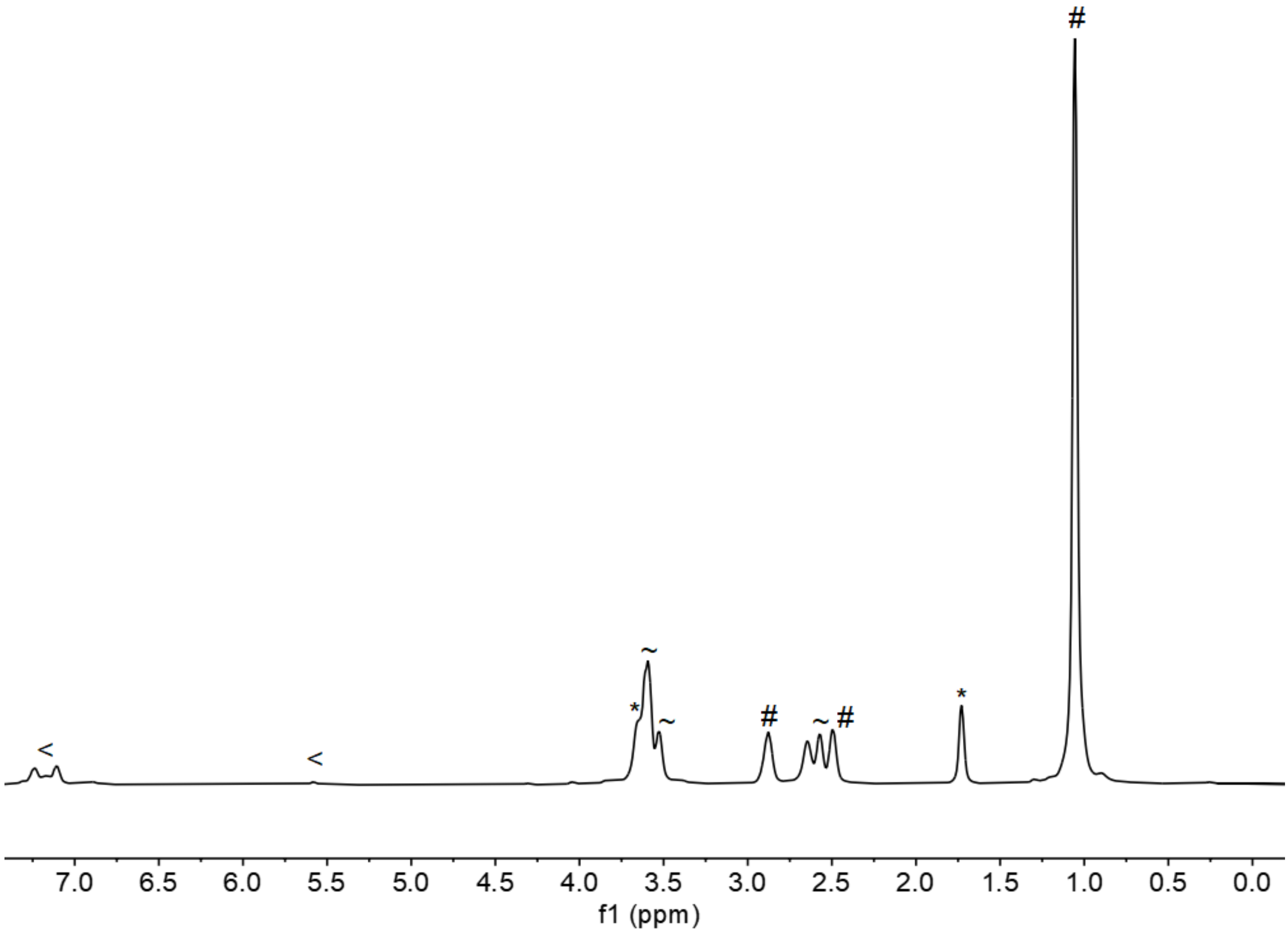

Supplementary Figure 58. Zoom-in of the ^{1}H NMR (D_8-THF, 298 K) spectrum of the crude reaction mixture resulting from the reaction of **6a/6b** with 10% [CPh_3][BPh_4]. The tilde (~) at ~3.60, 3.50, and 2.53 ppm denotes 2.2.2 cryptand; # = $Tren^{TIPS}H_3$; < = $HCPh_3$.

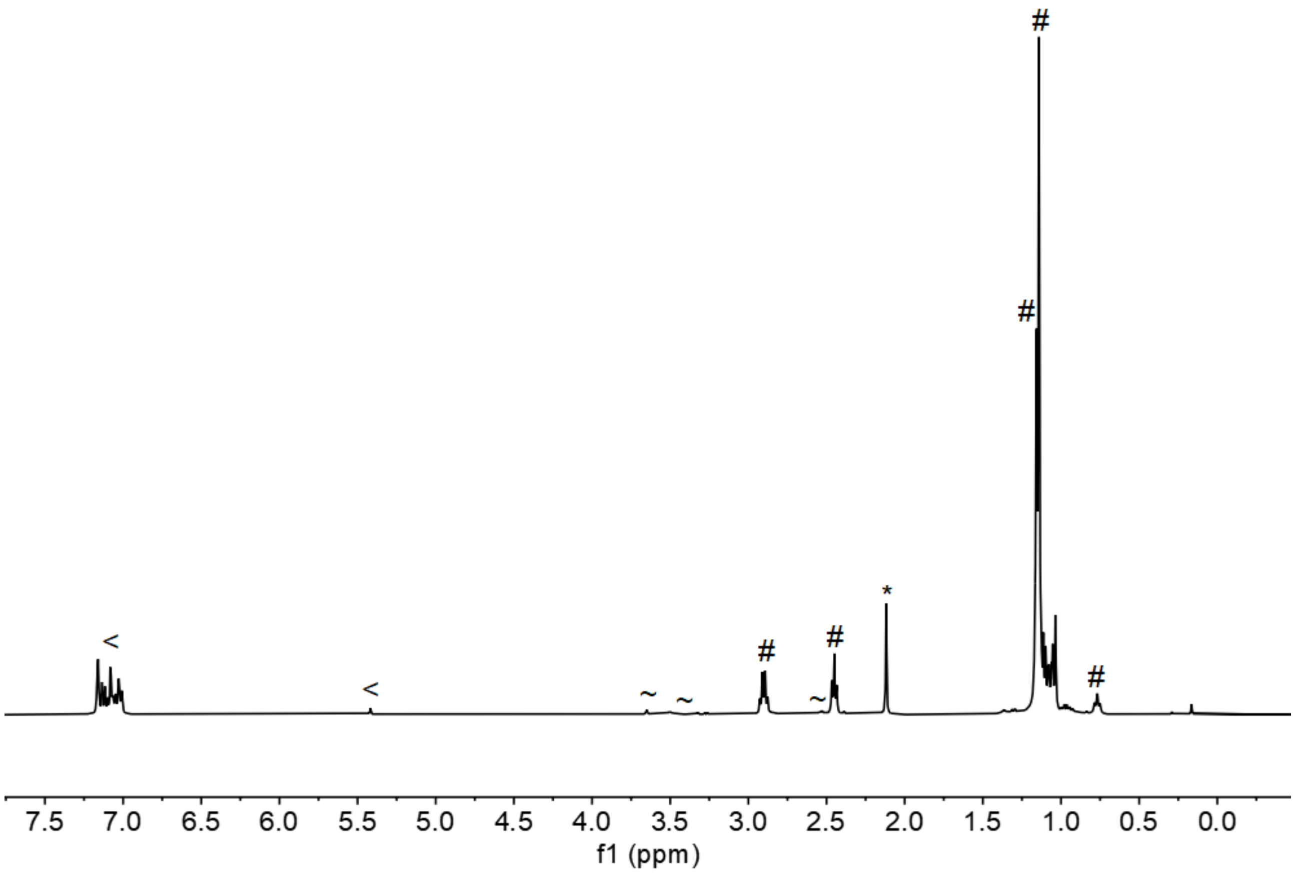

Supplementary Figure 59. ^{1}H NMR (D_6-benzene, 298 K) spectrum of the crude reaction mixture resulting from the reaction of **6a/6b** with 10% $[CPh_3][BPh_4]$. The tilde (~) at ~3.60, 3.50, and 2.50 ppm denotes 2.2.2 cryptand; # = $Tren^{TIPS}H_3$; < = $HCPh_3$.

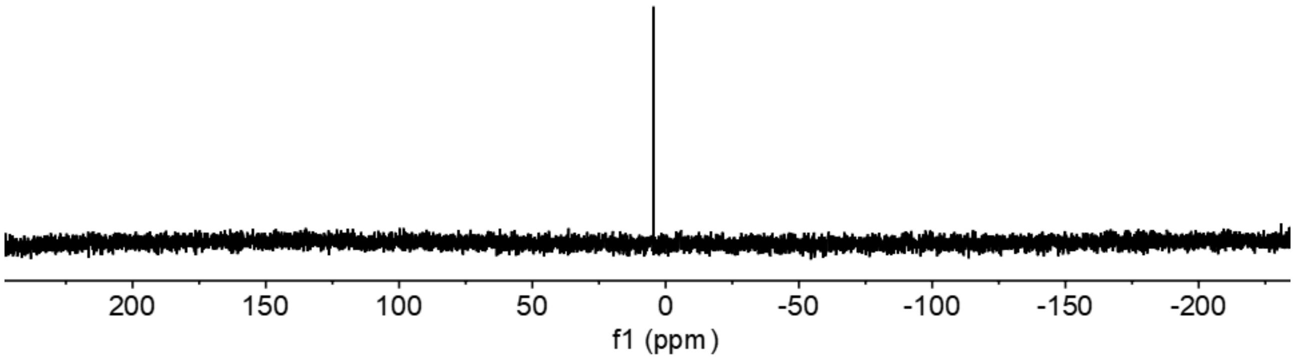

Supplementary Figure 60. $^{29}Si\{^{1}H\}$ NMR (D_6-benzene, 298 K) spectrum of the crude reaction mixture resulting from the reaction of **6a/6b** with 10% $[CPh_3][BPh_4]$ showing the presence of $Tren^{TIPS}H_3$ at ~4.40 ppm.

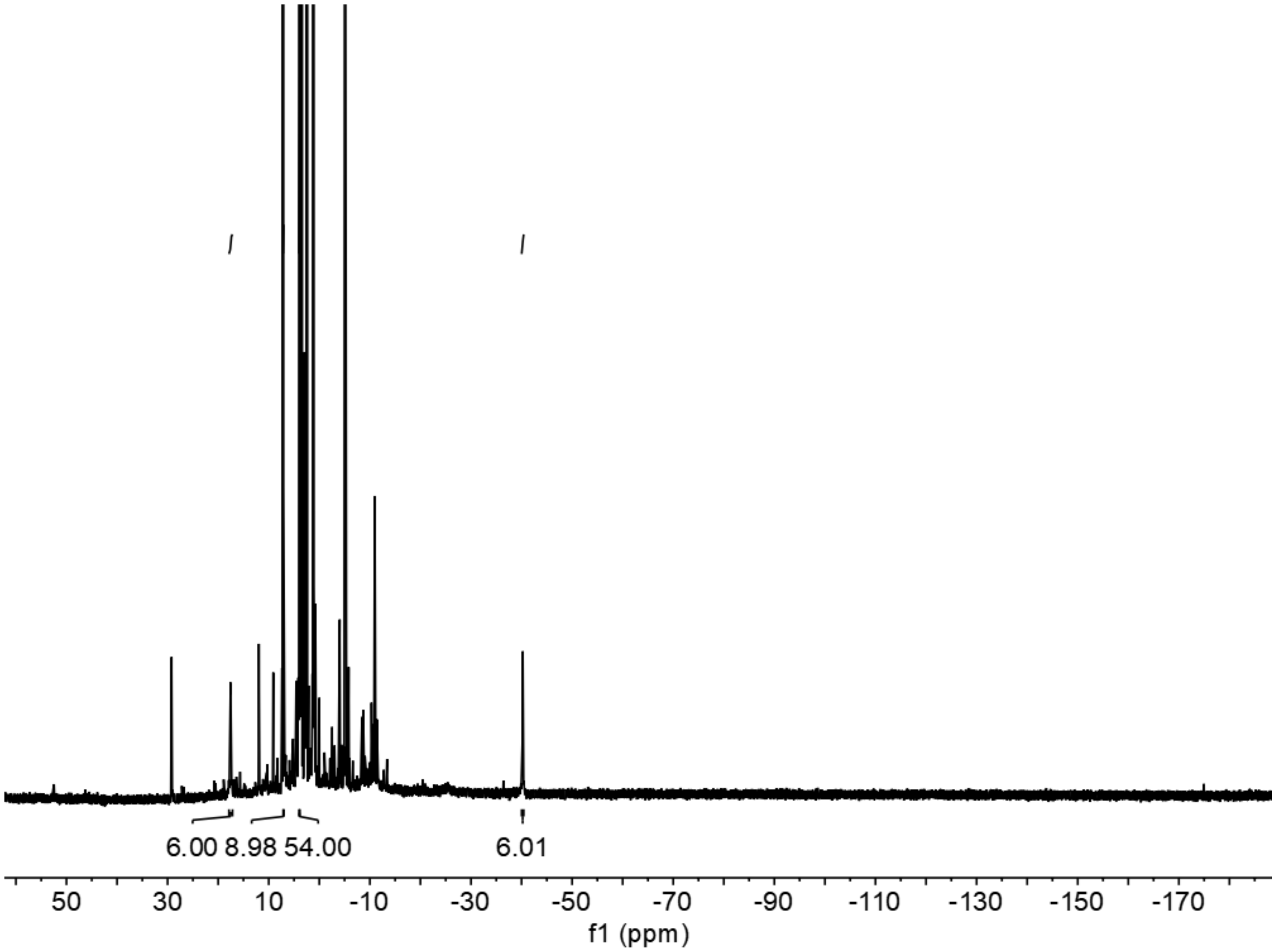

Supplementary Figure 61. ^{1}H NMR (D_6-benzene, 298 K) spectrum of the crude reaction mixture resulting from the reaction of **6a/6b** with 10% [$HNEt_3$][BPh_4]. Integrations represent the four ^{1}H environments of Tren^{TIPS}U: δ 17.55 (6H, s, CH_2), 7.09 (9H, s, Pr^i-C*H*), 3.92 (54H, s, Pr^i-CH_3), –40.25 (6H, s, CH_2) ppm.

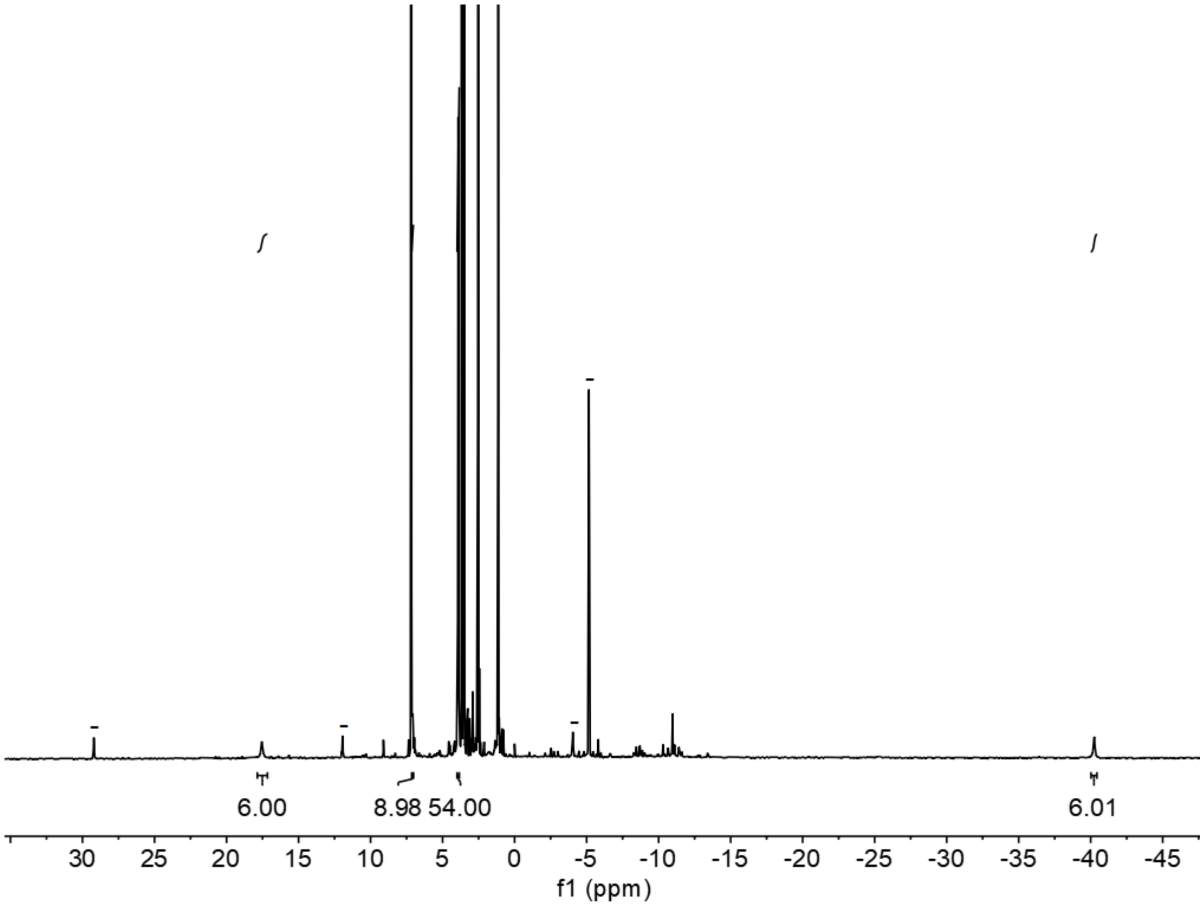

Supplementary Figure 62. Zoom-in of the ^{1}H NMR (D_6-benzene, 298 K) spectrum of the crude reaction mixture resulting from the reaction of **6a/6b** with 10% $[HNEt_3][BPh_4]$. Integrations represent the four ^{1}H environments of $Tren^{TIPS}U$: δ 17.55 (6H, s, CH_2), 7.09 (9H, s, Pr^i-C*H*), 3.92 (54H, s, Pr^i-CH_3), –40.25 (6H, s, CH_2) ppm. The hyphens (-) denote **9**.

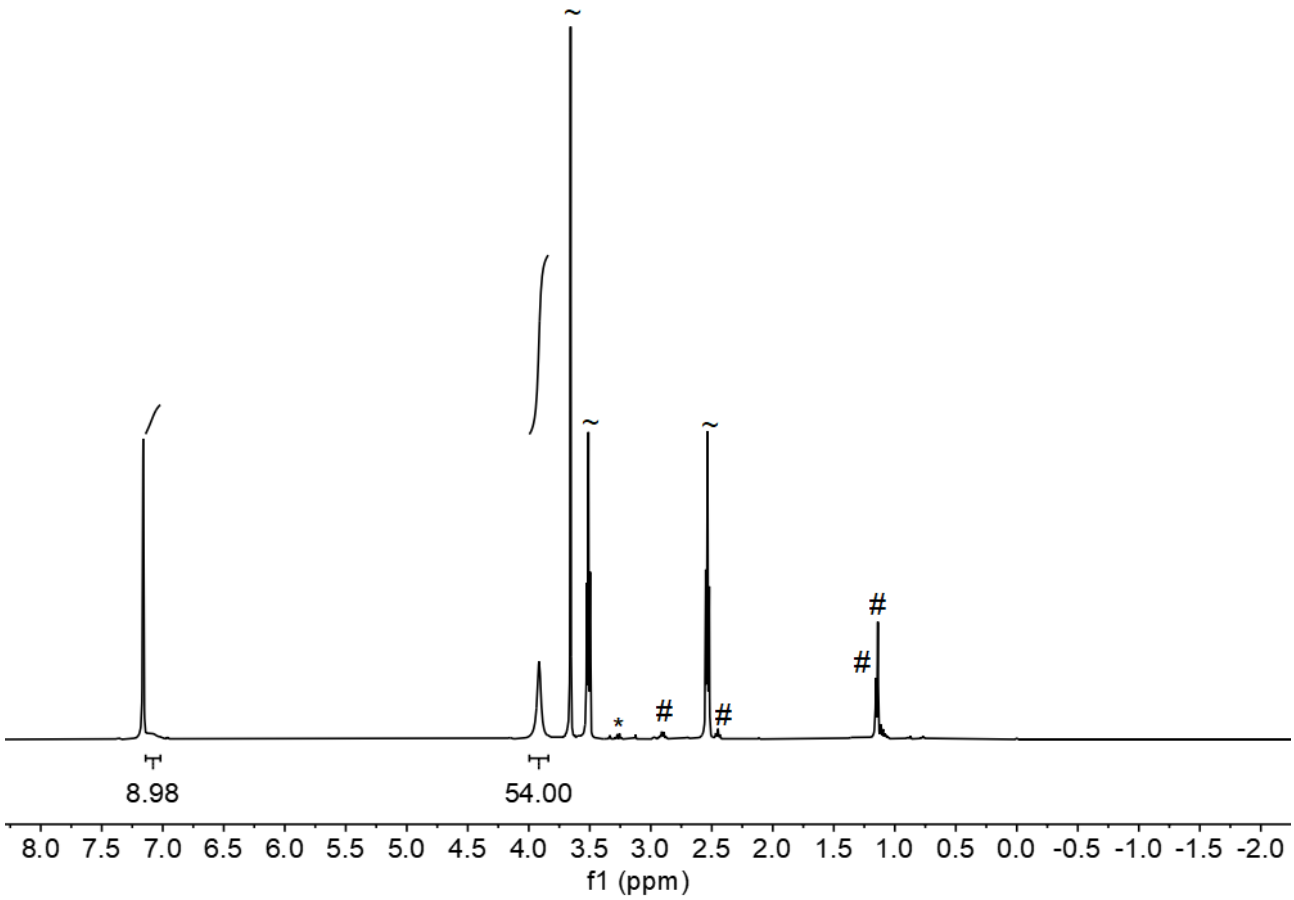

Supplementary Figure 63. Zoom-in of the ^{1}H NMR (D_6-benzene, 298 K) spectrum of the crude reaction mixture resulting from the reaction of **6a/6b** with 10% [$HNEt_3$][BPh_4]. Integrations represent two ^{1}H environments of Tren^{TIPS}U: δ 7.09 (9H, s, Pr^{i}-C*H*), 3.92 (54H, s, Pr^{i}-CH_3 ppm. The tilde (~) at ~3.60, 3.50, and 2.53 ppm denotes 2.2.2 cryptand; # = Tren^{TIPS}H_3; * = trace Et_2O.

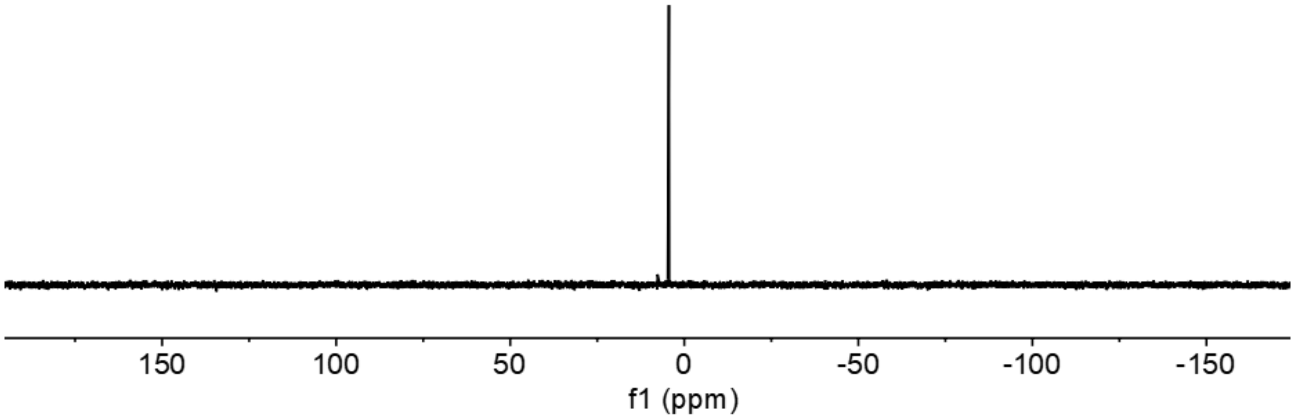

Supplementary Figure 64. ^{29}Si{^{1}H} NMR (D_6-benzene, 298 K) spectrum of the crude reaction mixture resulting from the reaction of **6a/6b** with 10% [$HNEt_3$][BPh_4] showing the presence of Tren^{TIPS}H_3 at ~4.40 ppm.

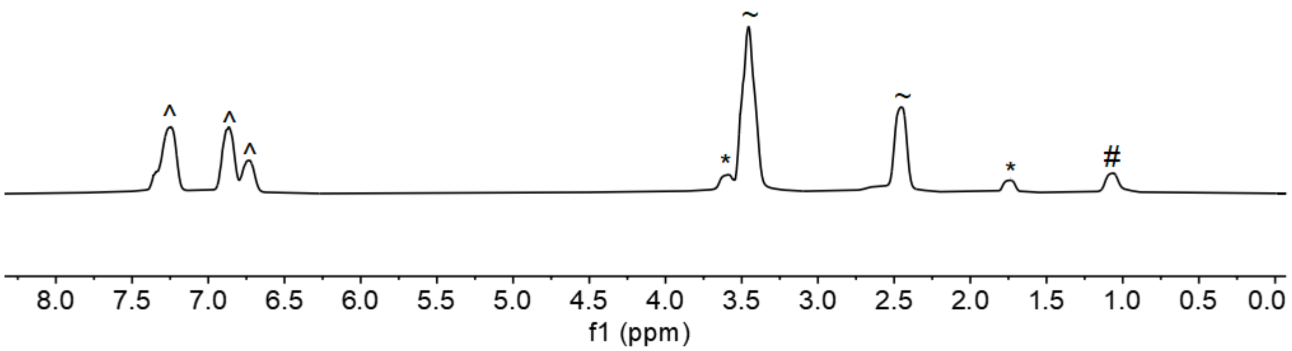

Supplementary Figure 65. ^{1}H NMR (D_8-THF, 298 K) spectrum of the crude reaction mixture resulting from the reaction of **6a/6b** with 10% [$HNEt_3$][BPh_4]. The tilde (~) at ~3.50, and 2.50 ppm denotes 2.2.2 cryptand; # = $Tren^{TIPS}H_3$; * = THF solvent; ^ = $KBPh_4$.

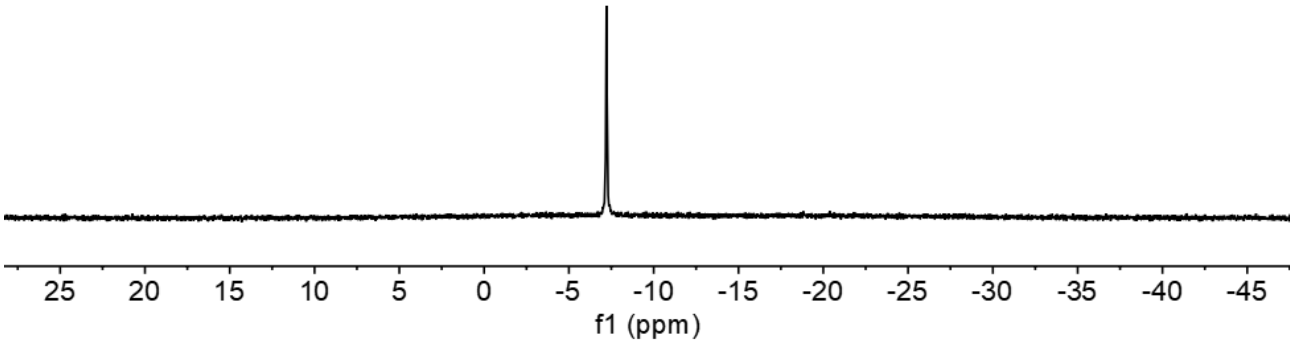

Supplementary Figure 66. ^{11}B NMR (D_8-THF, 298 K) spectrum of the crude reaction mixture resulting from the reaction of **6a/6b** with 10% [$HNEt_3$][BPh_4] showing the presence of $KBPh_4$ at ~ −7 ppm.

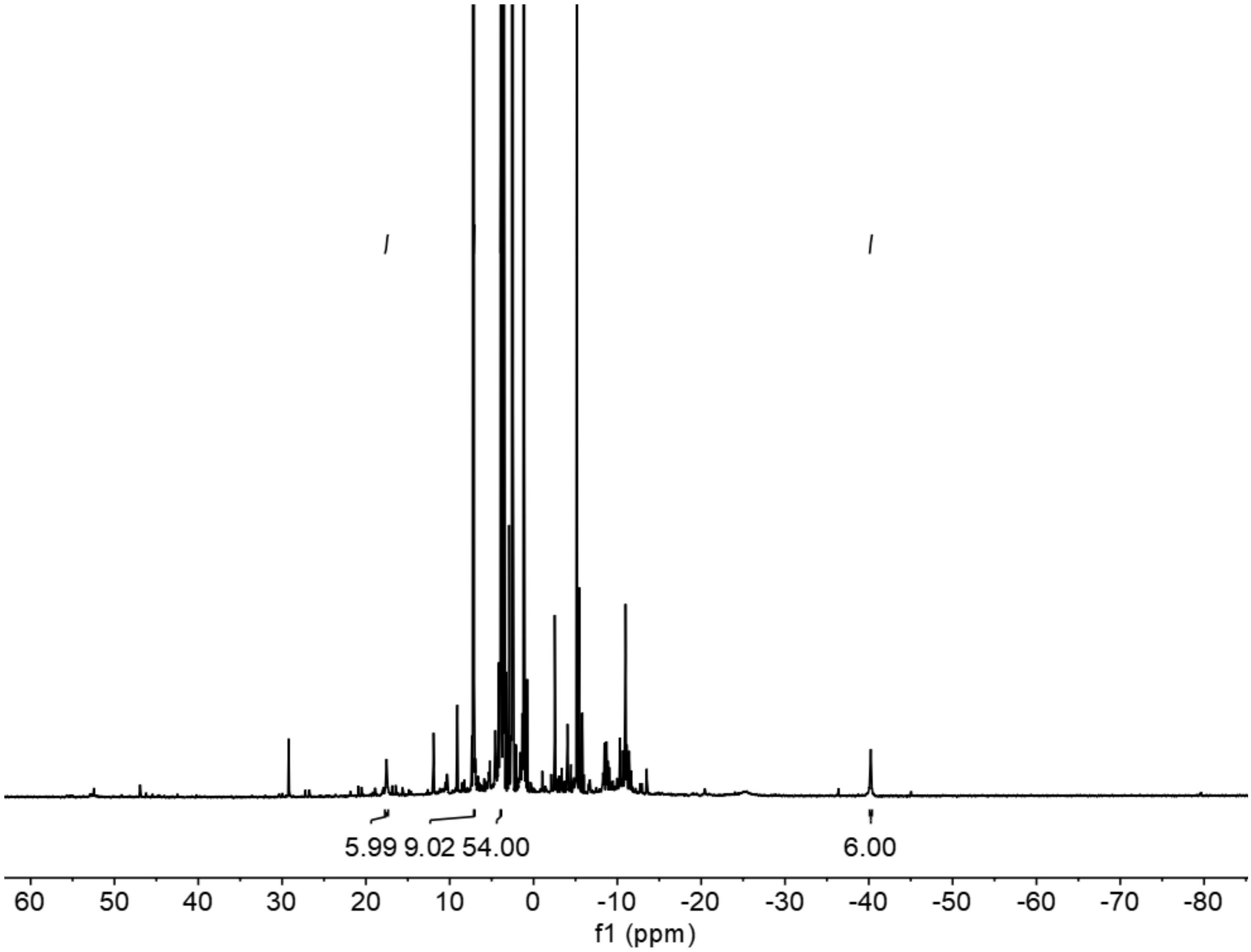

Supplementary Figure 67. ^{1}H NMR (D_6-benzene, 298 K) spectrum of the crude reaction mixture resulting from the reaction of **6a/6b** with 10% $TlBPh_4$. Integrations represent the four ^{1}H environments of Tren^{TIPS}U: δ 17.54 (6H, s, CH_2), 7.08 (9H, s, Pr^i-C*H*), 3.91 (54H, s, Pr^i-CH_3), –40.23 (6H, s, CH_2) ppm.

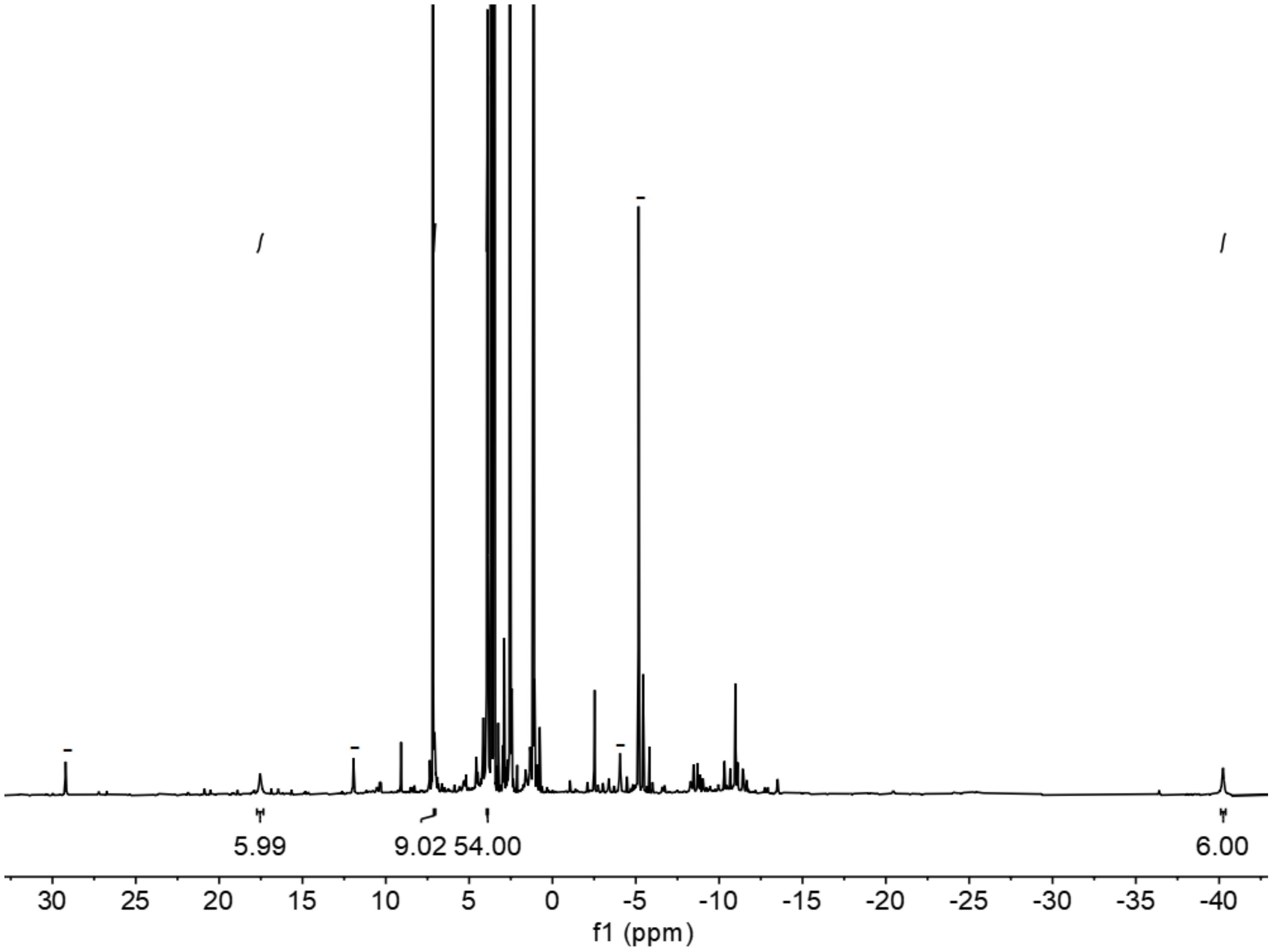

Supplementary Figure 68. Zoom-in of the ^{1}H NMR (D_6-benzene, 298 K) spectrum of the crude reaction mixture resulting from the reaction of **6a/6b** with 10% $TlBPh_4$. Integrations represent the four ^{1}H environments of Tren^{TIPS}U: δ 17.54 (6H, s, CH_2), 7.08 (9H, s, Pr^i-CH), 3.91 (54H, s, Pr^i-CH_3), –40.23 (6H, s, CH_2) ppm. The hyphens (-) denote **9**.

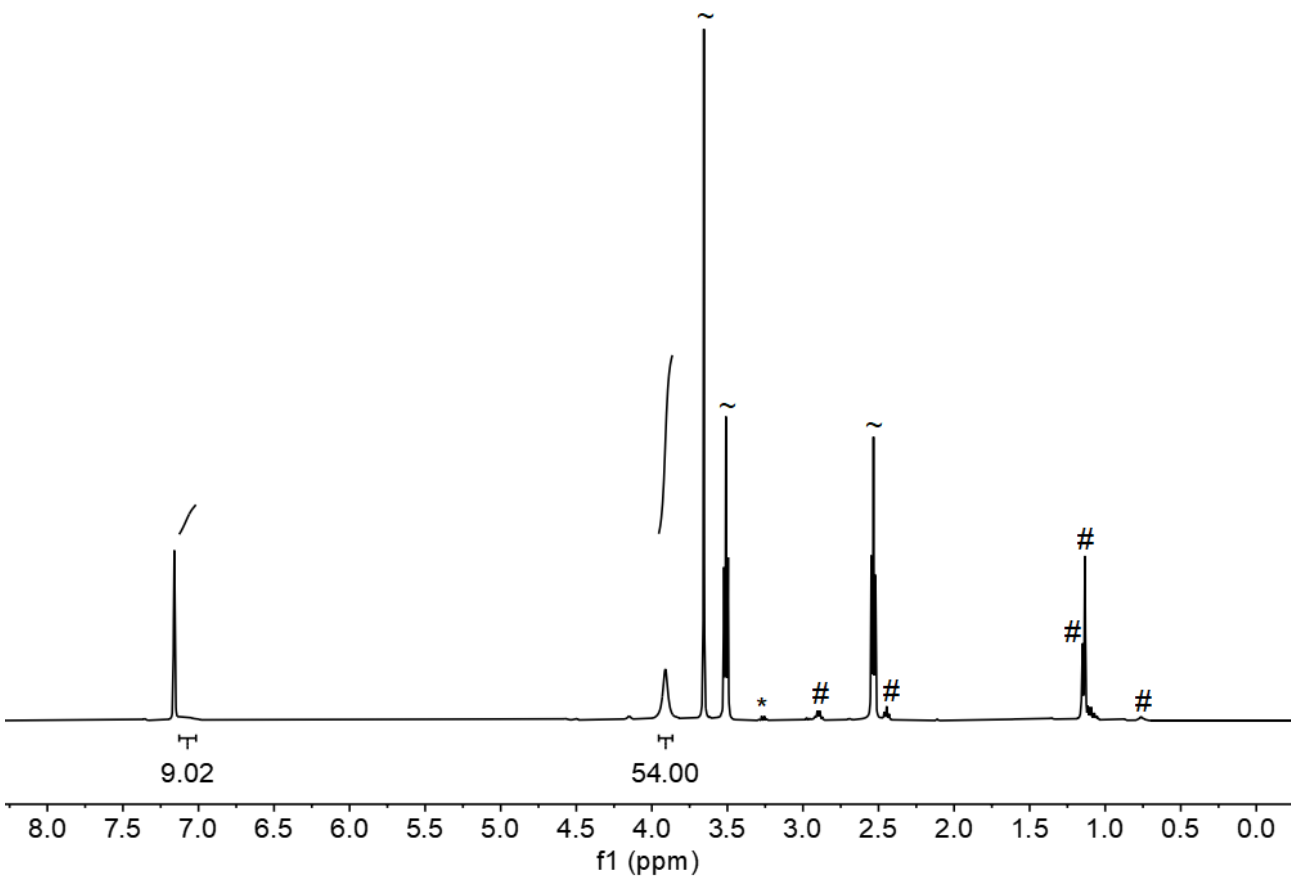

Supplementary Figure 69. Zoom-in of the ^{1}H NMR (D_6-benzene, 298 K) spectrum of the crude reaction mixture resulting from the reaction of **6a/6b** with 10% $TlBPh_4$. Integrations represent two ^{1}H environments of Tren^{TIPS}U: δ 7.09 (9H, s, Pr^{i}-C*H*), 3.92 (54H, s, Pr^{i}-C*H*$_3$ ppm. The tilde (~) at ~3.60, 3.50, and 2.53 ppm denotes 2.2.2 cryptand; # = Tren$^{TIPS}H_3$; * = trace Et_2O.

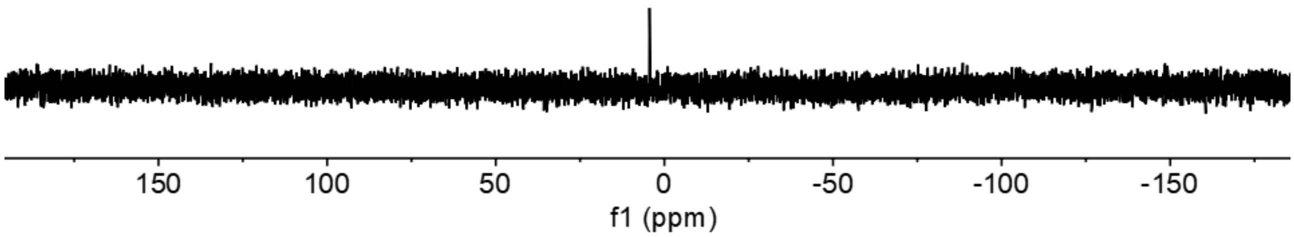

Supplementary Figure 70. $^{29}Si\{^{1}H\}$ NMR (D_6-benzene, 298 K) spectrum of the crude reaction mixture resulting from the reaction of **6a/6b** with 10% $TlBPh_4$ showing the presence of Tren$^{TIPS}H_3$ at ~4.40 ppm.

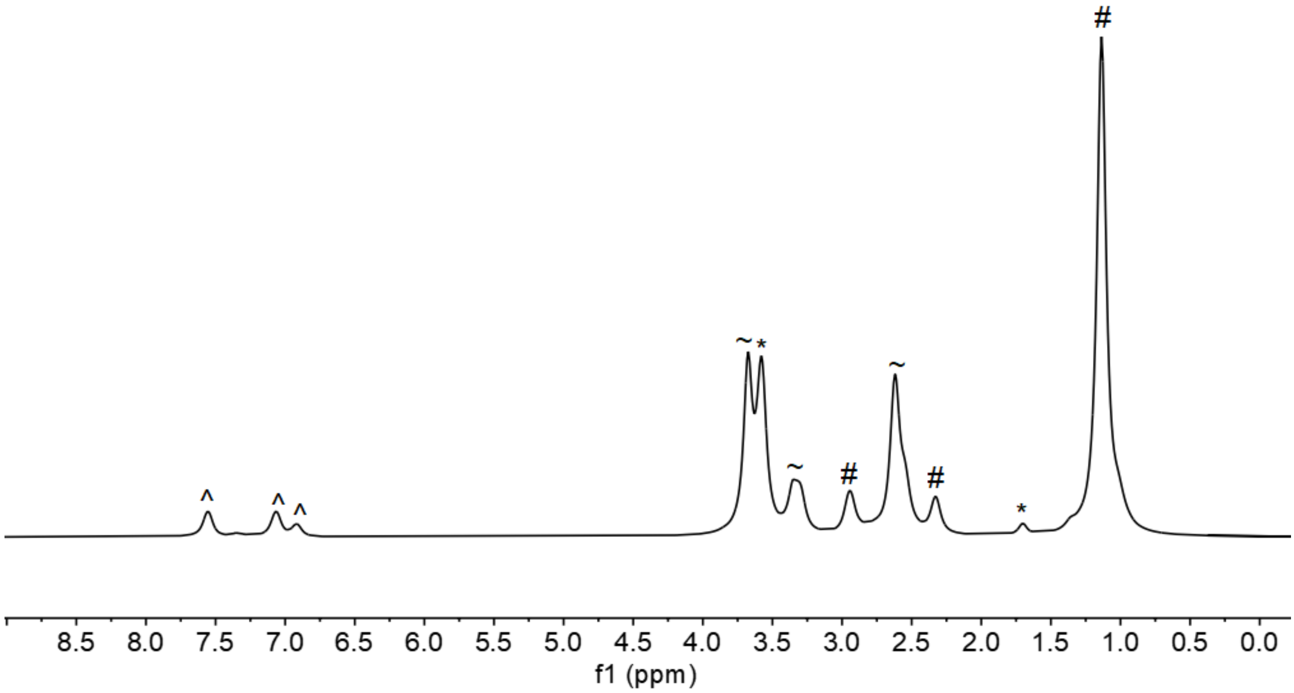

Supplementary Figure 71. ^{1}H NMR (D_8-THF, 298 K) spectrum of the crude reaction mixture resulting from the reaction of **6a/6b** with 10% $TlBPh_4$. The tilde (~) at ~3.60, 3.50, and 2.50 ppm denotes 2.2.2 cryptand; # = Tren$^{TIPS}H_3$; * = THF solvent; ^ = $KBPh_4$.

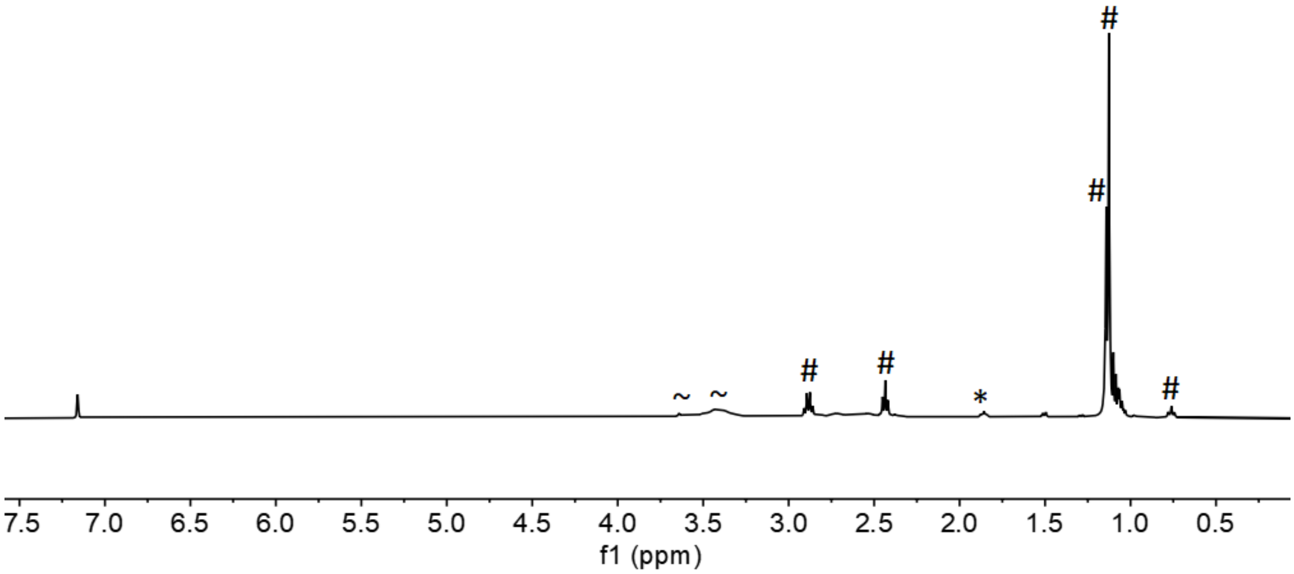

Supplementary Figure 72. ^{1}H NMR (D_6-benzene, 298 K) spectrum of the crude reaction mixture resulting from the reaction of **6a/6b** with 10% trimethylsilyl iodide. The tilde (~) at ~3.60 and 3.50 ppm denotes 2.2.2 cryptand; # = Tren$^{TIPS}H_3$; * = trace Et_2O.

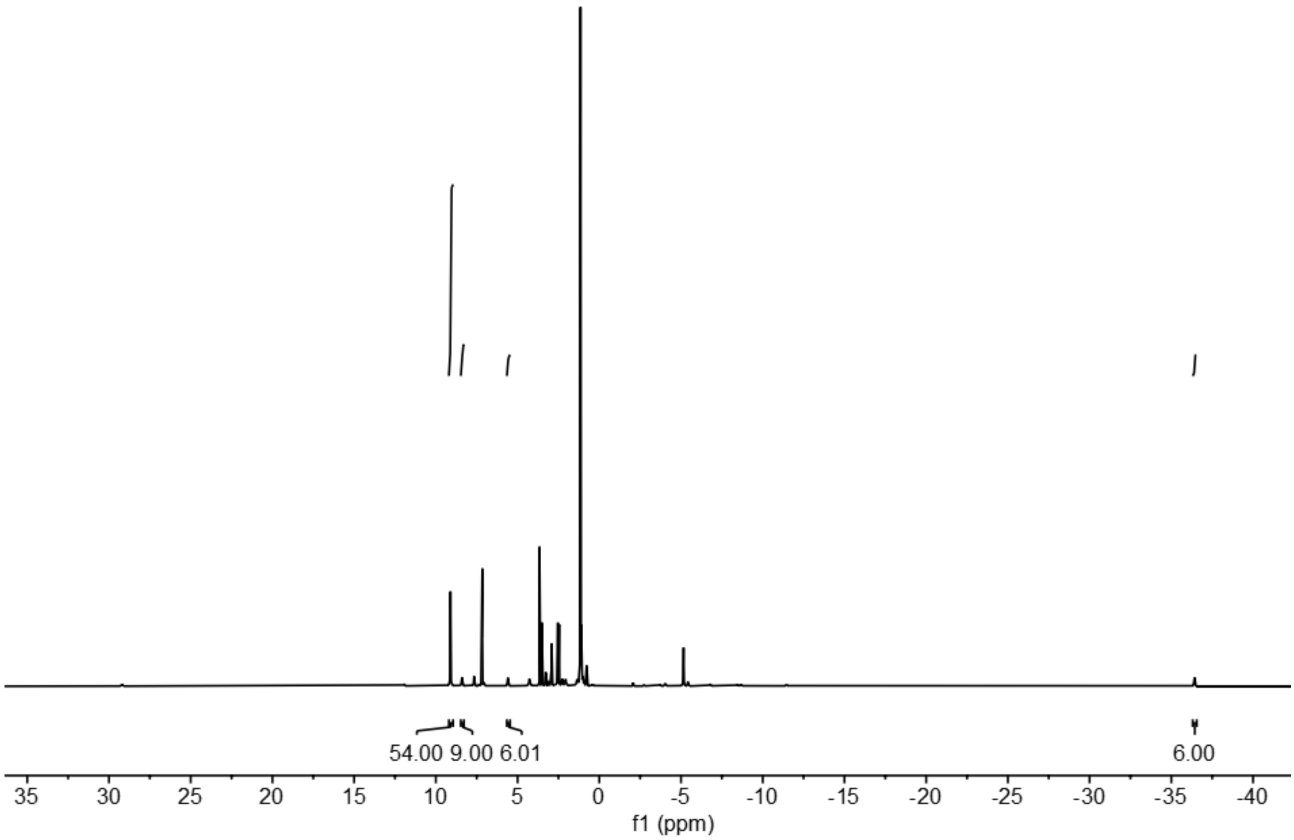

Supplementary Figure 73. ^{1}H NMR (D_6-benzene, 298 K) spectrum of the crude reaction mixture resulting from the reaction of **6a/6b** with 10% Ph_3SnCl. Integrations represent the four ^{1}H environments of $[U^{IV}(Tren^{TIPS})Cl]$: δ 9.09 (54H, s, Pr^{i}-CH_3), 8.41 (9H, s, Pr^{i}-CH), 5.59 (6H, s, CH_2), −36.43 (6H, s, CH_2) ppm.

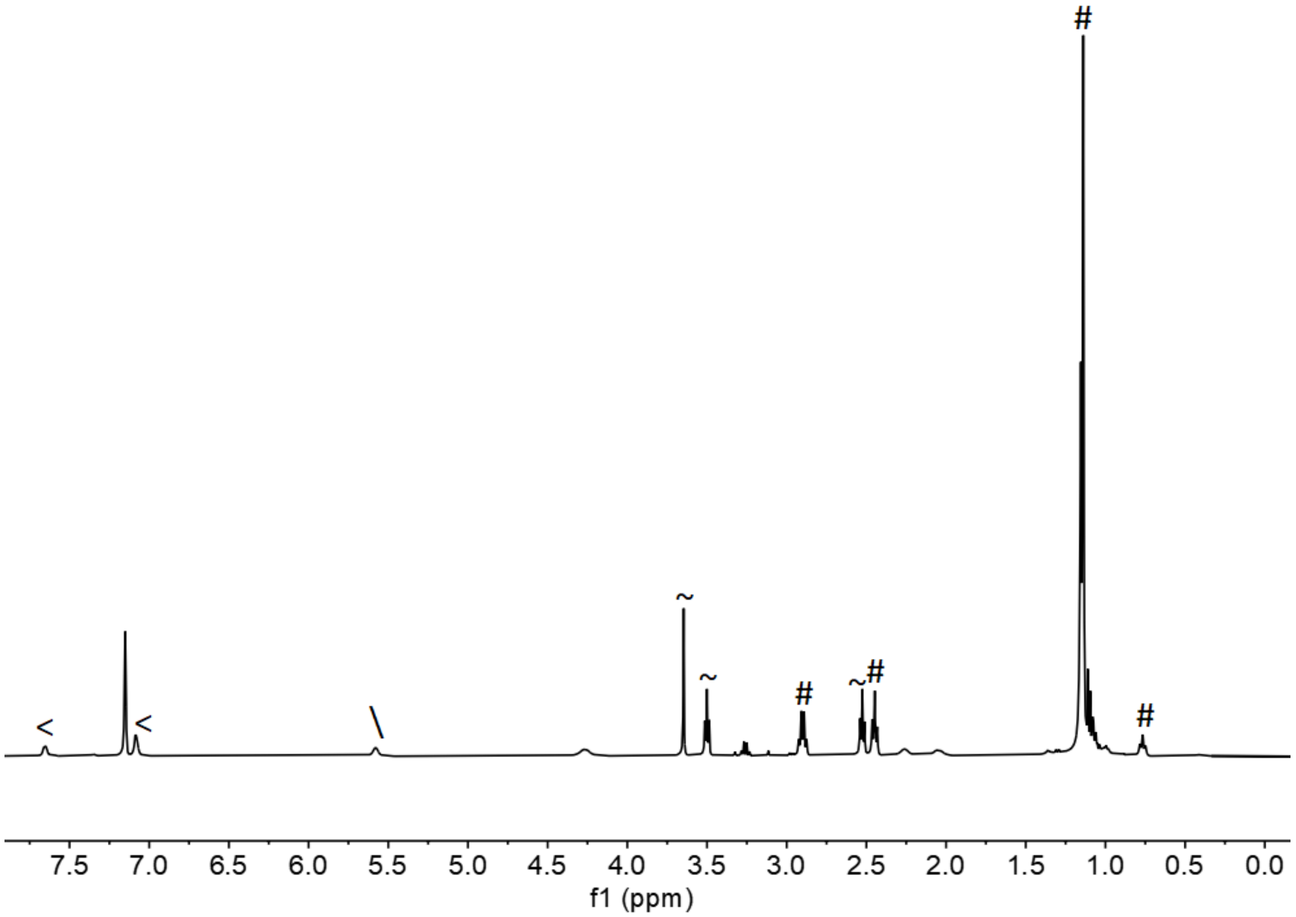

Supplementary Figure 74. Zoom-in of ^{1}H NMR (D_6-benzene, 298 K) spectrum of the crude reaction mixture resulting from the reaction of **6a/6b** with 10% Ph_3SnCl. The tilde (~) at ~3.60, 3.50, and 2.53 ppm denotes 2.2.2 cryptand; # = $Tren^{TIPS}H_3$; < = Sn_2Ph_6; \ = $[U^{IV}(Tren^{TIPS})Cl]$.

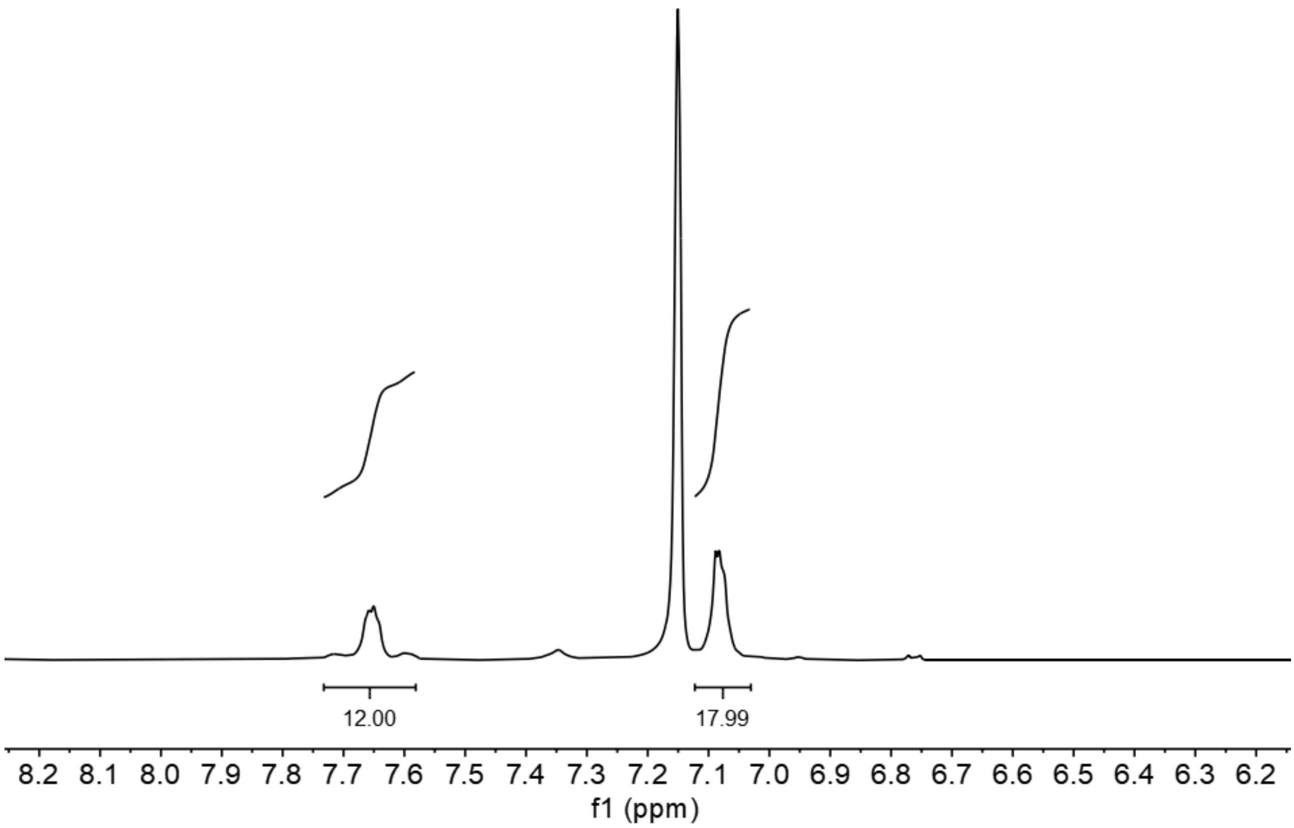

Supplementary Figure 75. Zoom-in of ^{1}H NMR (D_6-benzene, 298 K) spectrum of the crude reaction mixture resulting from the reaction of **6a/6b** with 10% Ph_3SnCl highlighting the resonances at ~7.75-7.60 and ~7.11–7.08 ppm attributed to Sn_2Ph_6.

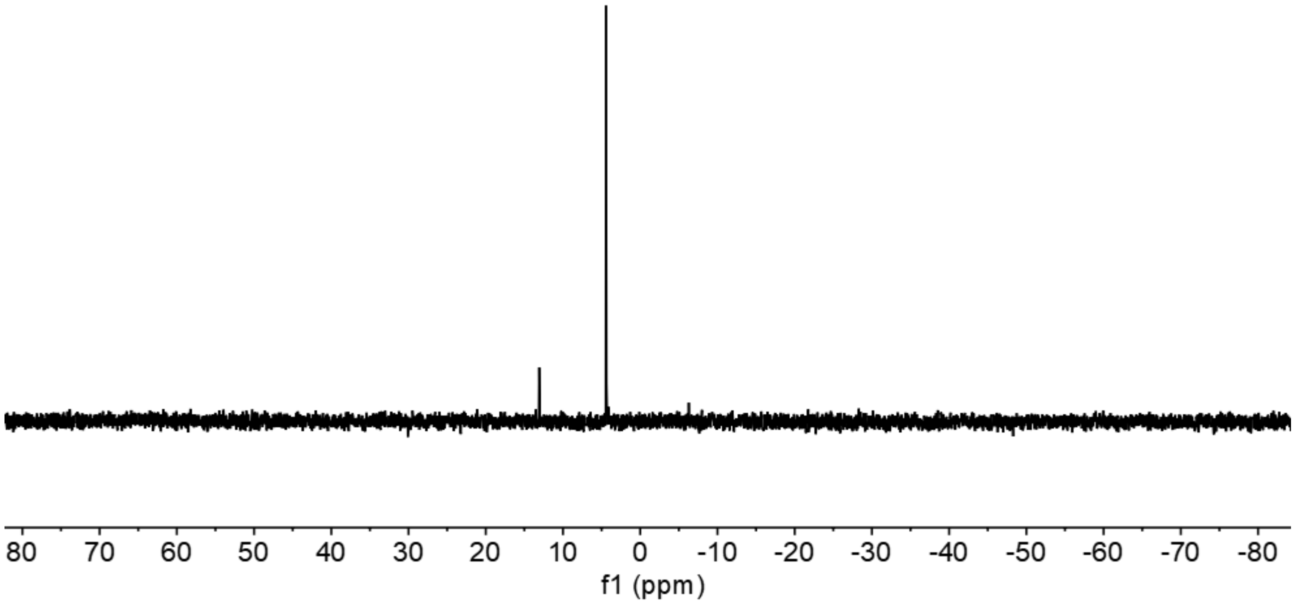

Supplementary Figure 76. $^{29}Si\{^{1}H\}$ NMR (D_6-benzene, 298 K) spectrum of the crude reaction mixture resulting from the reaction of **6a/6b** with 10% Ph_3SnCl showing the presence of $[U^{IV}(Tren^{TIPS})Cl]$ at ~13.10 ppm and $Tren^{TIPS}H_3$ at ~4.40 ppm.

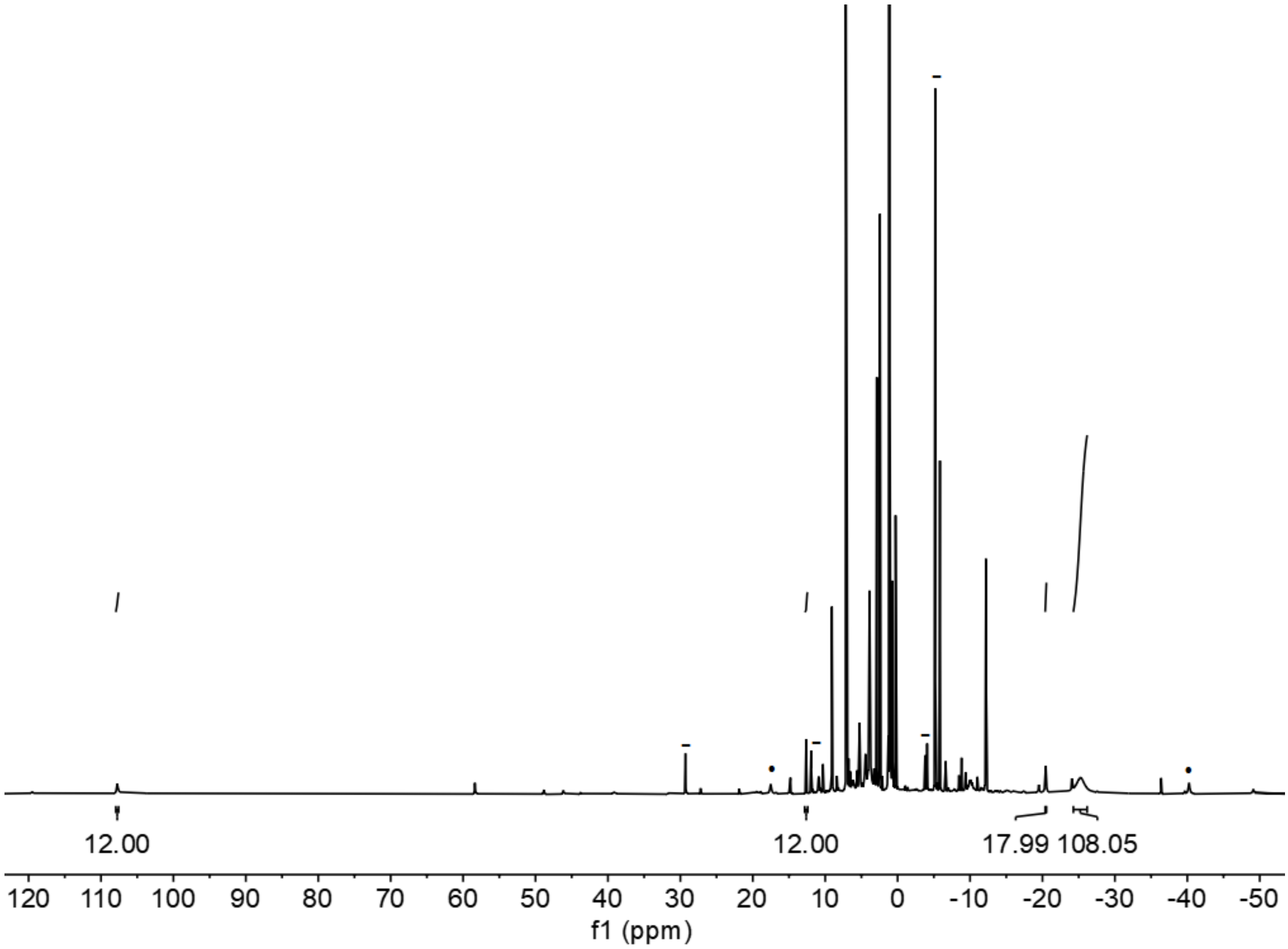

Supplementary Figure 77. ^{1}H NMR (D_6-benzene, 298 K) spectrum of the crude reaction mixture resulting from the synthesis of **8** as a minority product. Integrations represent the four ^{1}H environments of [{U^{IV}($Tren^{TIPS}$)}$_2$(μ-^{2}:η^2-Bi_2)]: δ 107.76 (12H, s, C*H*$_2$), 12.64 (12H, s, C*H*$_2$), −20.46 (18H, s, Pr^i-C*H*), −25.31 (108H, s, br, Pr^i-C*H*$_3$) ppm. The dots (•) denote **1**; - = **9**.

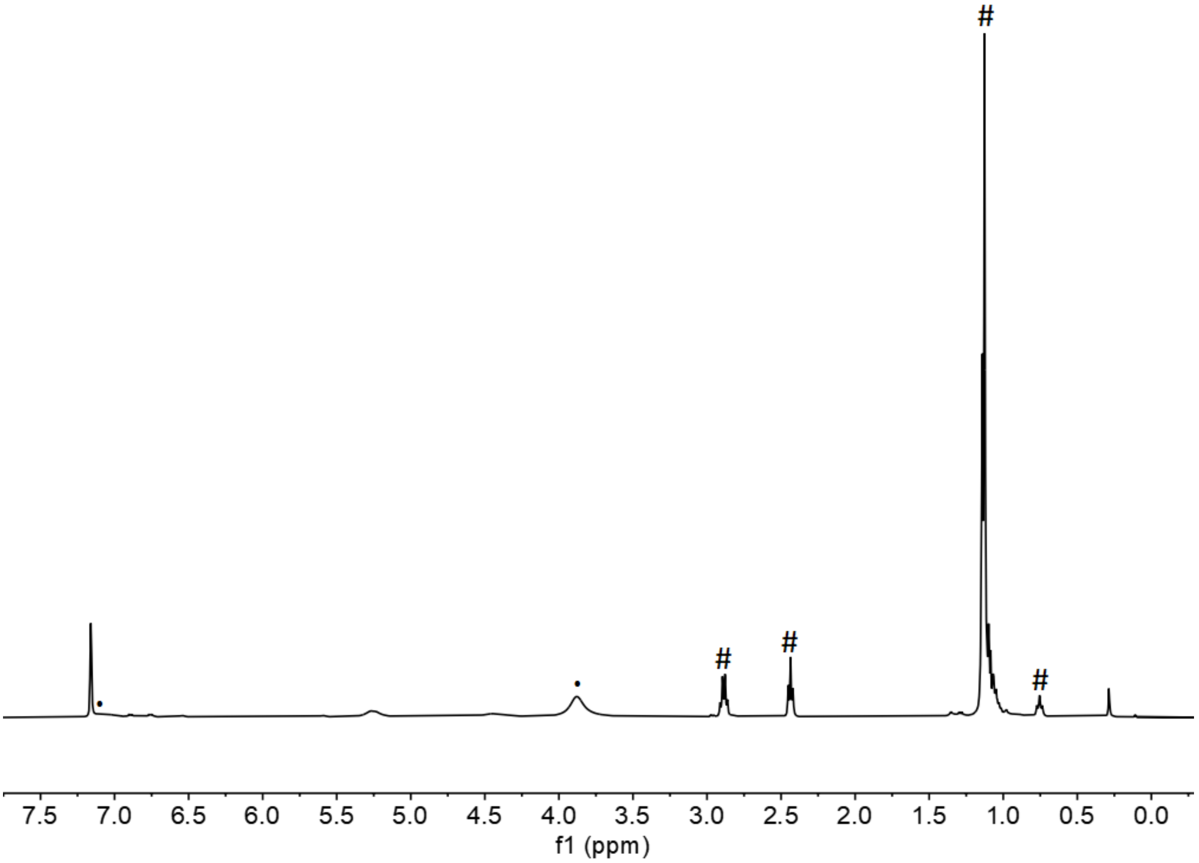

Supplementary Figure 78. Zoom-in of the ^{1}H NMR (D_6-benzene, 298 K) spectrum of the crude reaction mixture resulting from the synthesis of **8** as a minority product. The hash (#) at ~2.90, 2.46, 1.15, and 0.77 ppm denotes $Tren^{TIPS}H_3$; • = **1**.

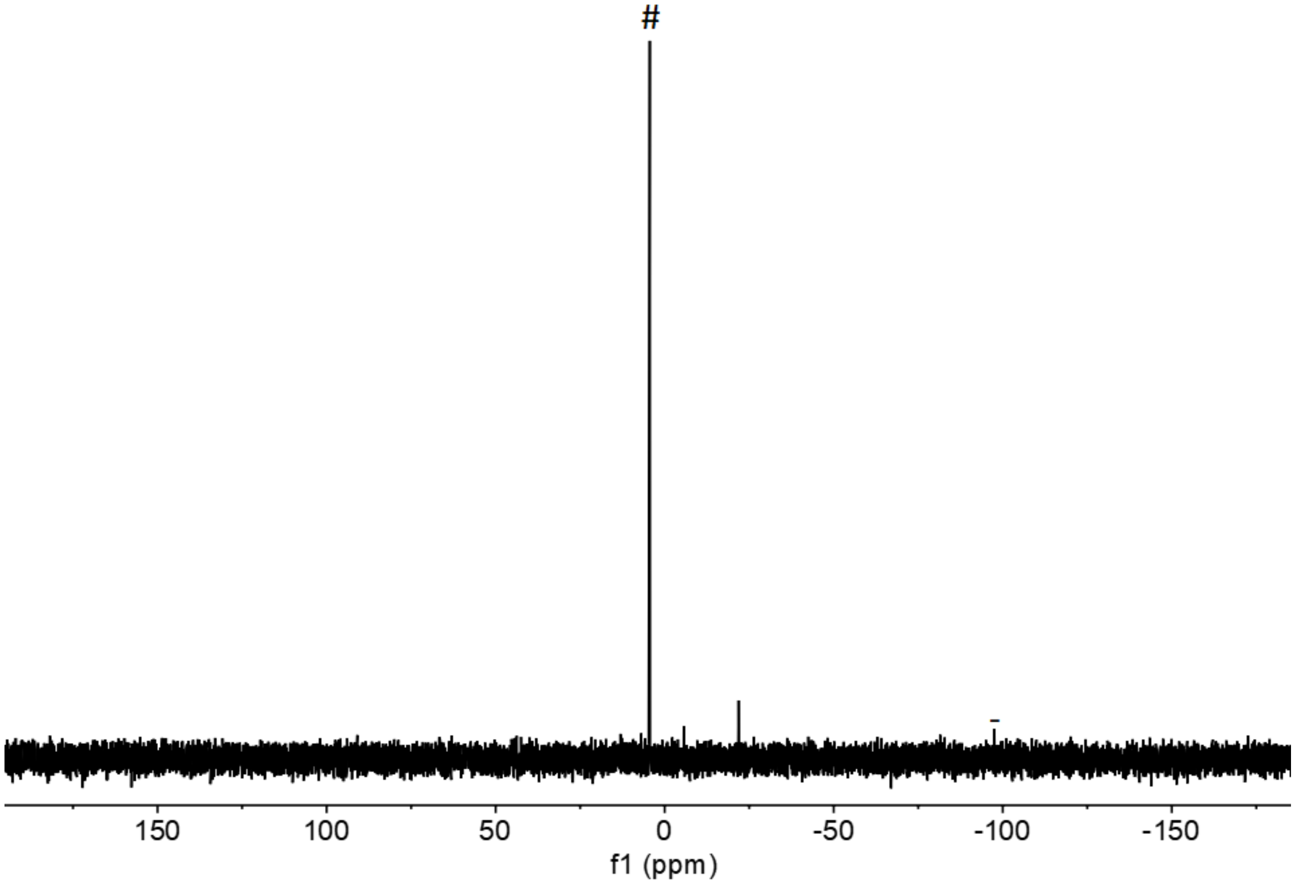

Supplementary Figure 79. $^{29}Si\{^{1}H\}$ NMR (D_6-benzene, 298 K) spectrum of the crude reaction mixture resulting from the synthesis of **8** as a minority product. # = Tren$^{TIPS}H_3$; - = **9**.

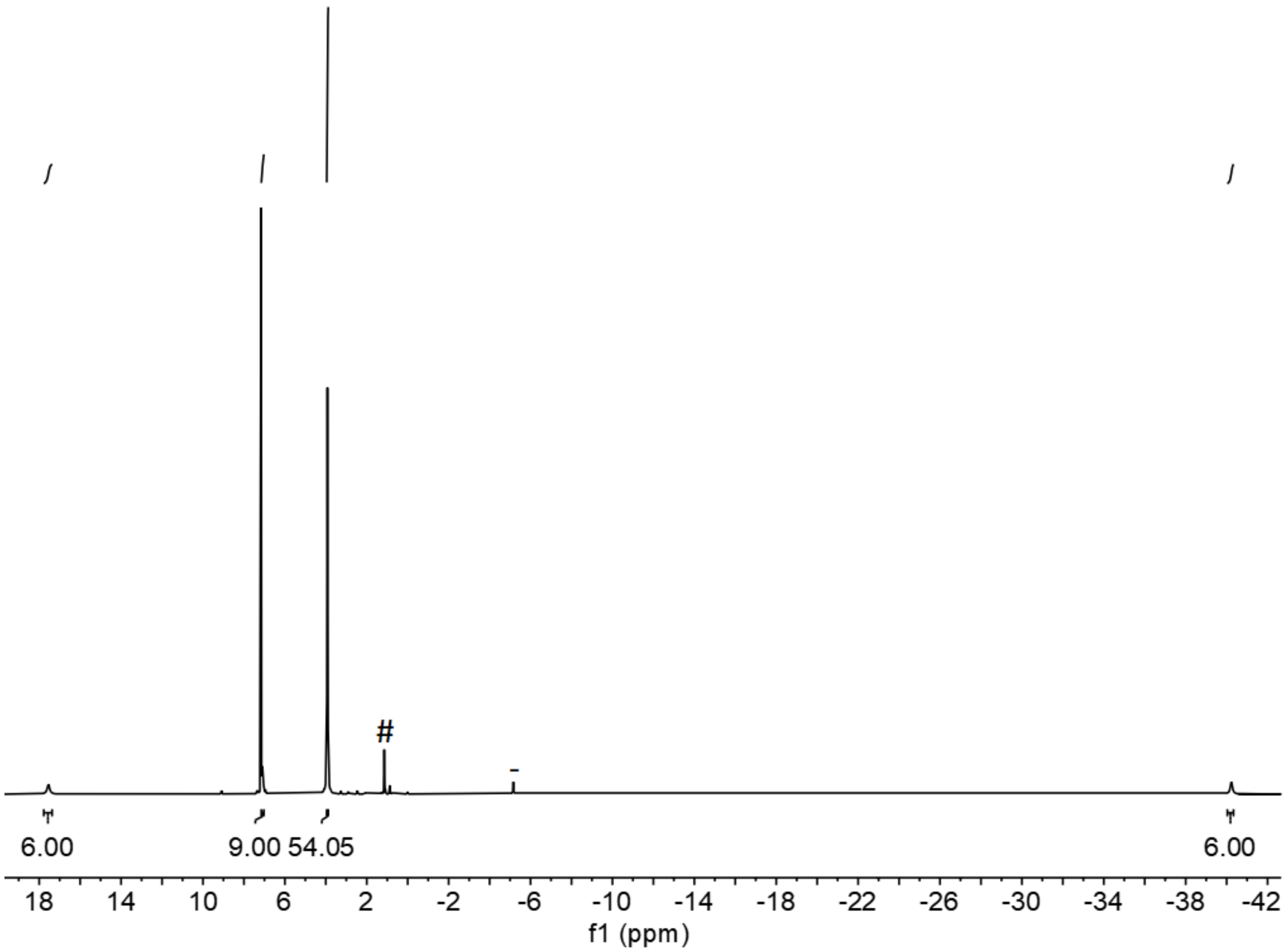

Supplementary Figure 80. ^{1}H NMR (D_6-benzene, 298 K) spectrum of the crude reaction mixture resulting from the reaction of **7** with [U^{IV}($Tren^{TIPS}$)(THF)][BPh_4] where the scale is greater than 0.05 mmol per U. Integrations represent the four ^{1}H environments of $Tren^{TIPS}$U: δ 17.54 (6H, s, CH_2), 7.08 (9H, s, Pr^i-CH), 3.91 (54H, s, Pr^i-CH_3), –40.23 (6H, s, CH_2) ppm. The hash (#) denotes $Tren^{TIPS}H_3$; - = **9**.

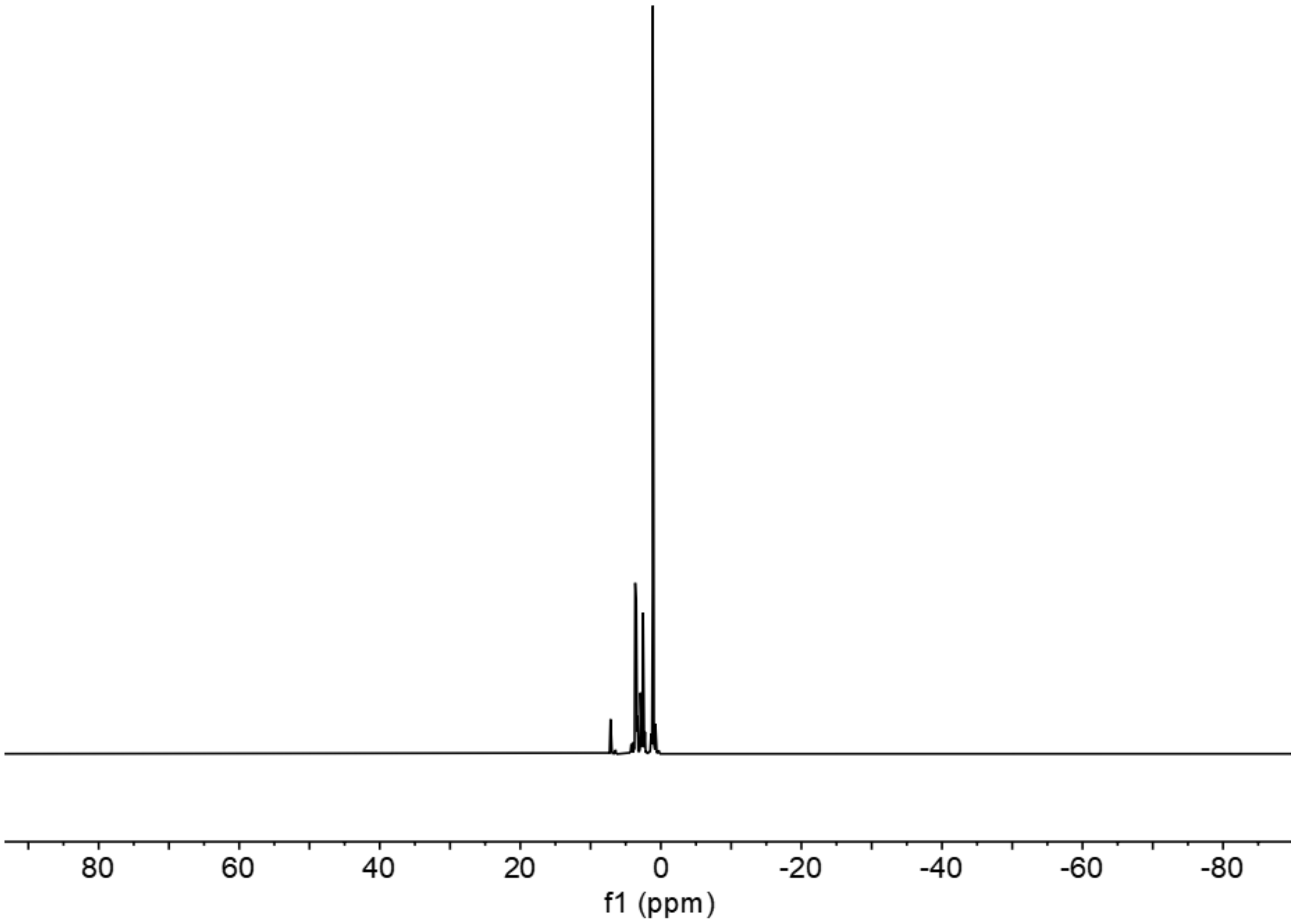

Supplementary Figure 81. ^{1}H NMR (D_6-benzene, 298 K) spectrum of the crude reaction mixture resulting from the reduction of the crude mixture of **8** with KC_8. The tilde (~) at ~3.60, 3.50, and 2.53 ppm denotes 2.2.2 cryptand; # = $Tren^{TIPS}H_3$.

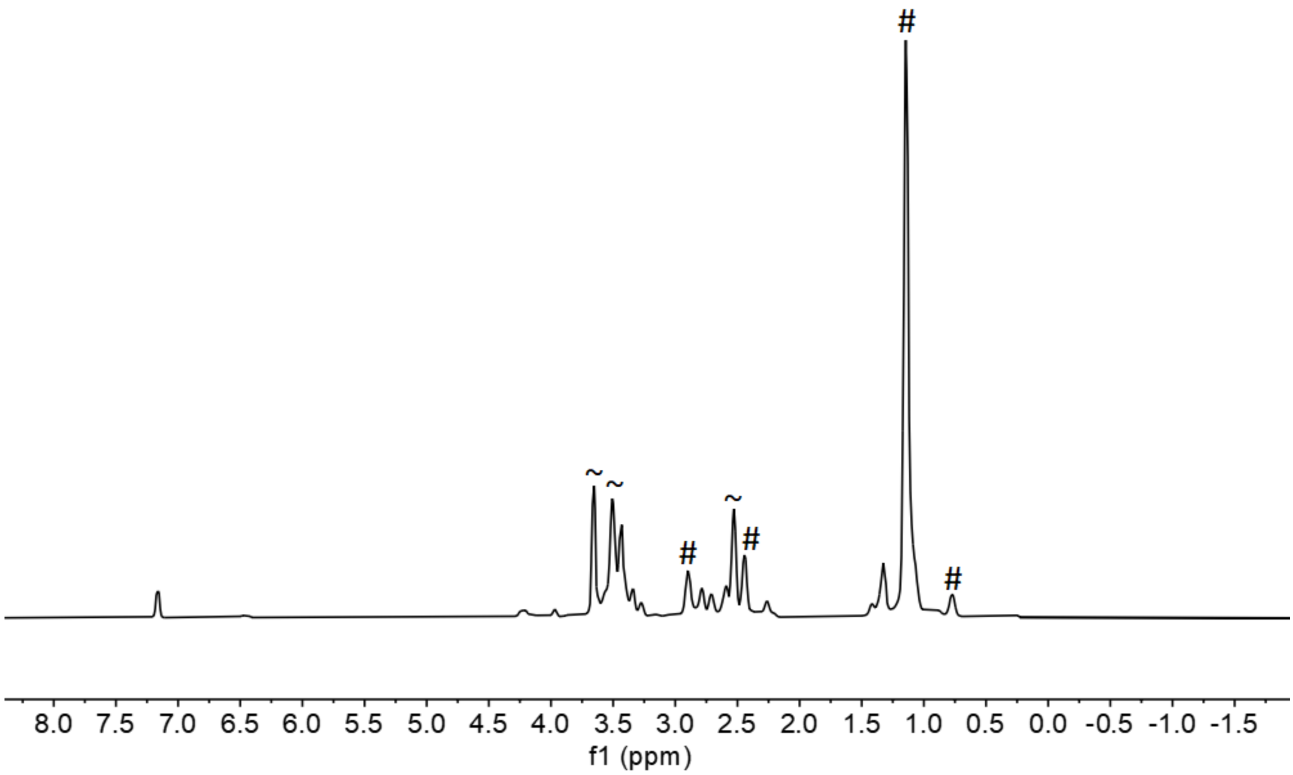

Supplementary Figure 82. Zoom-in of the ^{1}H NMR (D_6-benzene, 298 K) spectrum of the crude reaction mixture resulting from the reduction of the crude mixture of **8** with KC_8. The tilde (~) at ~3.60, 3.50, and 2.53 ppm denotes 2.2.2 cryptand; # = Tren^{TIPS}H_3.

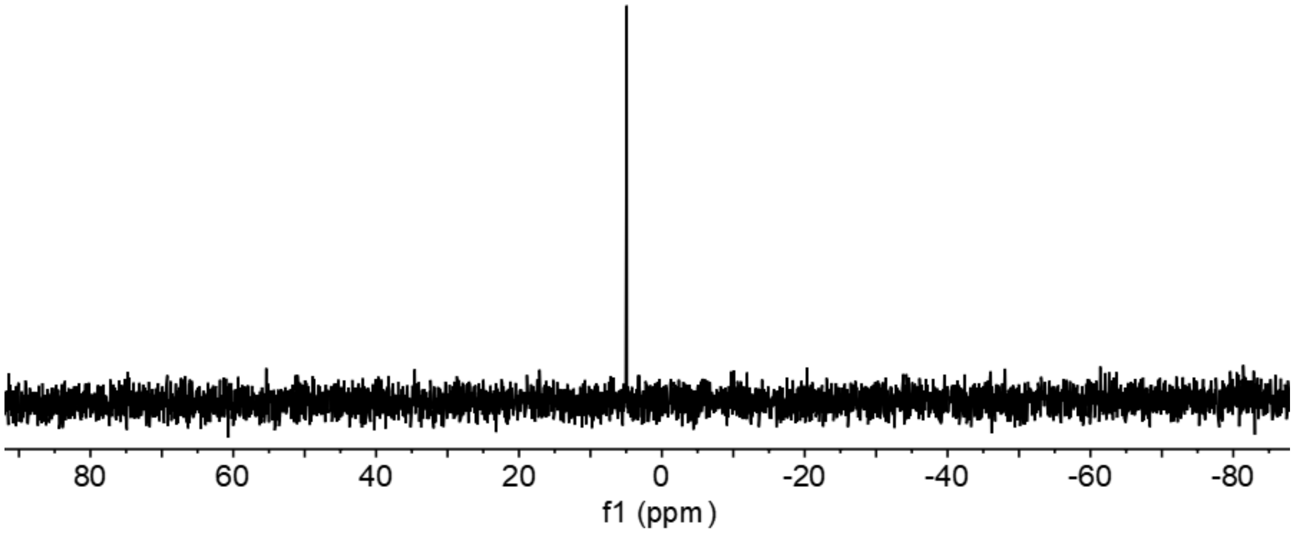

Supplementary Figure 83. ^{29}Si{^{1}H} NMR (D_6-benzene, 298 K) spectrum of the crude reaction mixture resulting from the reduction of the crude mixture of **8** with KC_8 showing the presence of Tren^{TIPS}H_3 at ~4.40 ppm.

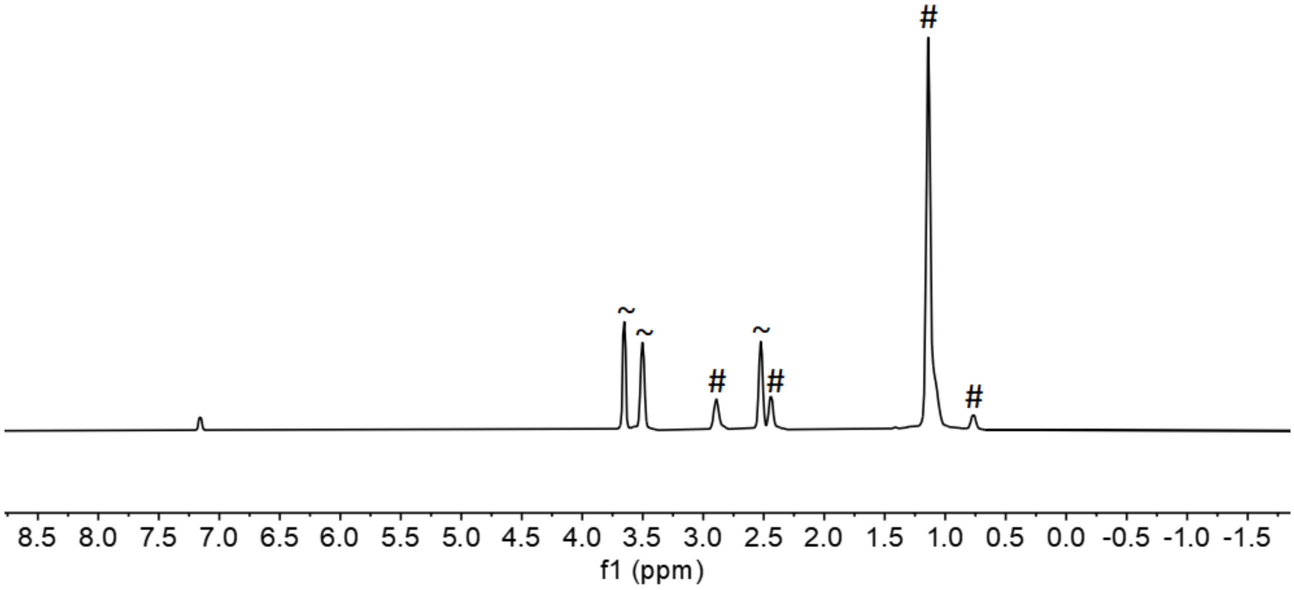

Supplementary Figure 84. Zoom-in of the ^{1}H NMR (D_6-benzene, 298 K) spectrum of the crude reaction mixture resulting from the reaction of **1** with **11**. The tilde (~) at ~3.60, 3.50, and 2.53 ppm denotes 2.2.2 cryptand; # = $Tren^{TIPS}H_3$.

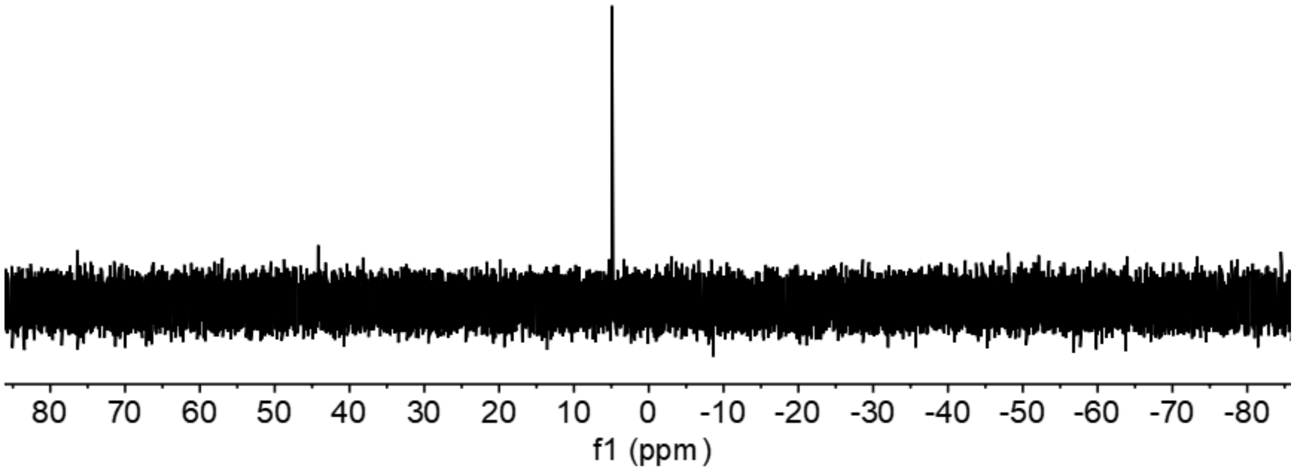

Supplementary Figure 85. $^{29}Si\{^{1}H\}$ NMR (D_6-benzene, 298 K) spectrum of the crude reaction mixture resulting from the reaction of **1** with **11** showing the presence of $Tren^{TIPS}H_3$ at ~4.40 ppm.

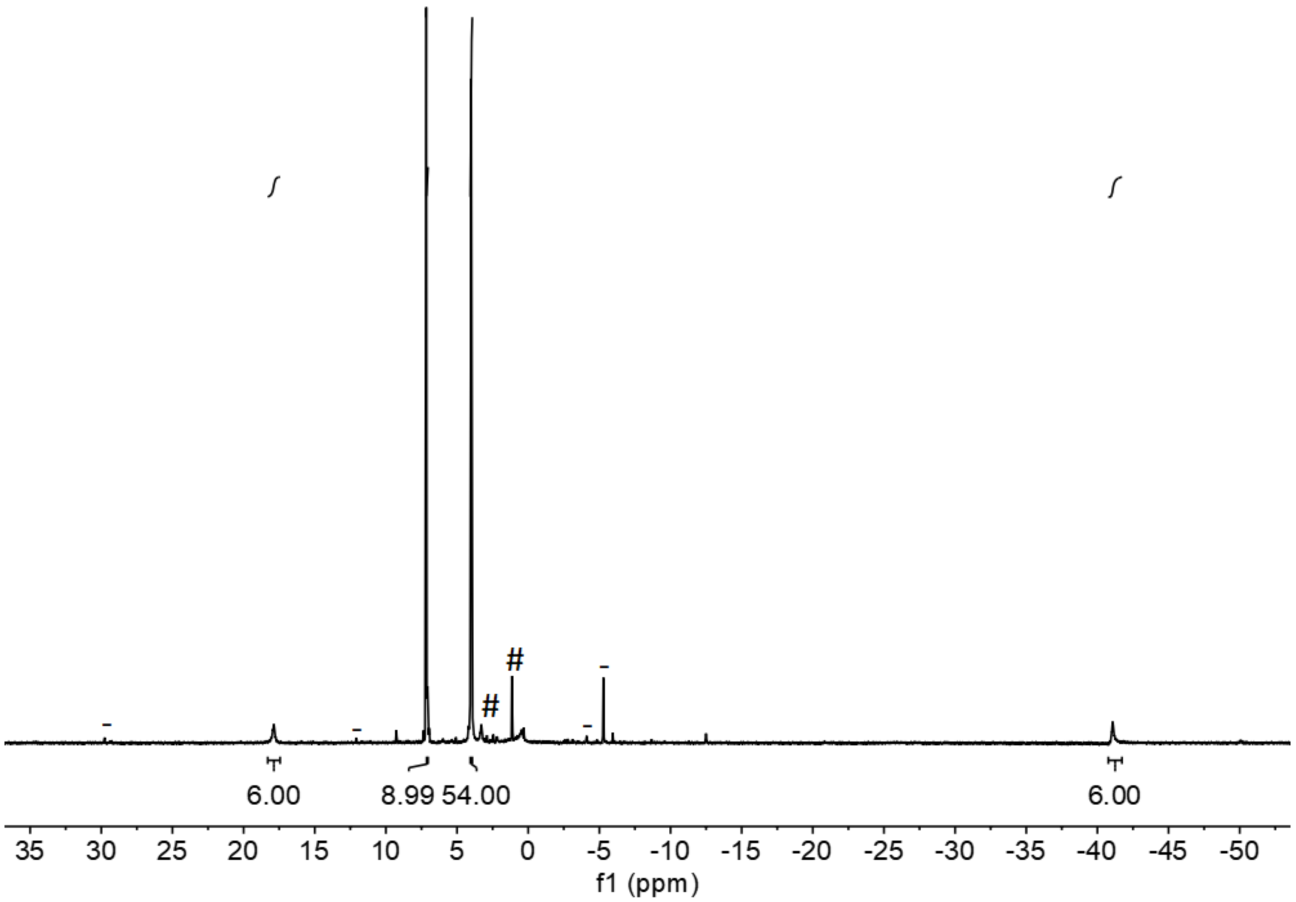

Supplementary Figure 86. ^{1}H NMR (D_6-benzene, 298 K) spectrum of the reaction mixture resulting from the reaction of [U^{IV}(Tren^{TIPS})(THF)][BPh_4] with **11**. Integrations represent the four ^{1}H environments of Tren^{TIPS}U: δ 17.88 (6H, s, CH_2), 7.08 (9H, s, Pr^{i}-CH), 4.00 (54H, s, Pr^{i}-CH_3), –41.07 (6H, s, CH_2) ppm. The hash (#) denotes Tren$^{TIPS}H_3$; - = **9**.

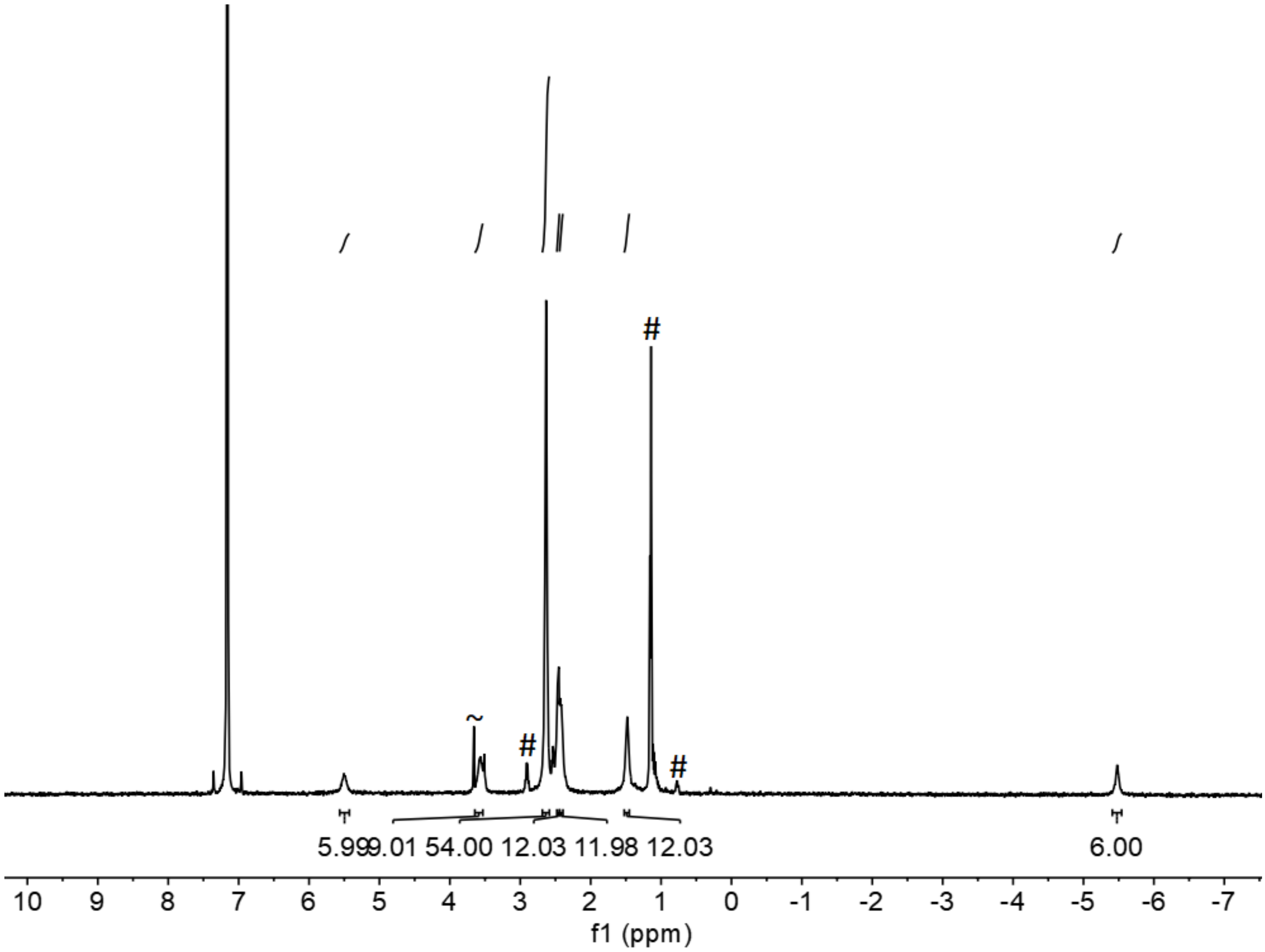

Supplementary Figure 87. Zoom-in of the ^{1}H NMR (D_6-benzene, 298 K) spectrum of the crude reaction mixture resulting from the reaction of $[U^{IV}(Tren^{TIPS})Cl]$ with **11**. Integrations represent the four ^{1}H environments of **12**, highlighting that this is the sole product in this reaction: δ 5.50 (6H, s, CH_2), 3.51 (9H, s, Pr^i-C*H*), 2.63 (54H, s, Pr^i-CH_3), 2.47 (12H, s, *C*H_2-cryptand), 2.44 (12H, t, *C*H_2-cryptand), 1.48 (12H, s, *C*H_2-cryptand), −5.48 (6H, s, CH_2) ppm. The tilde (~) at ~3.60 ppm denotes 2.2.2-cryptand; # = $Tren^{TIPS}H_3$.

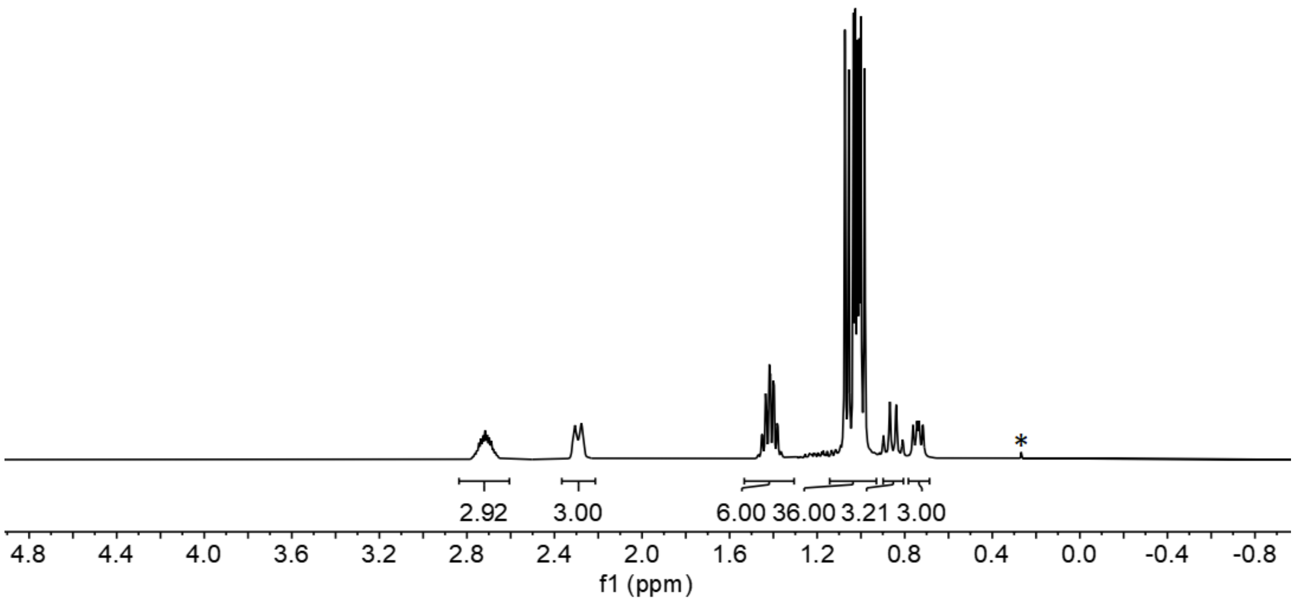

Supplementary Figure 88. ^{1}H NMR (D_6-benzene, 298 K) spectrum of **13**. * = trace silicon grease.

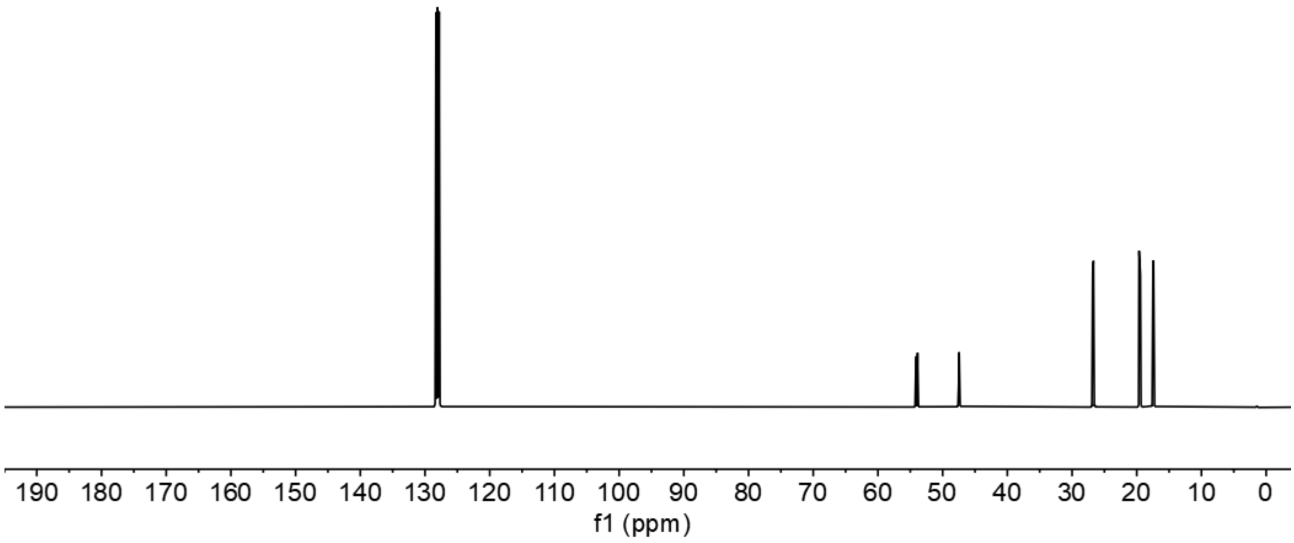

Supplementary Figure 89. $^{13}C\{^{1}H\}$ NMR (D_6-benzene, 298 K) spectrum of **13**.

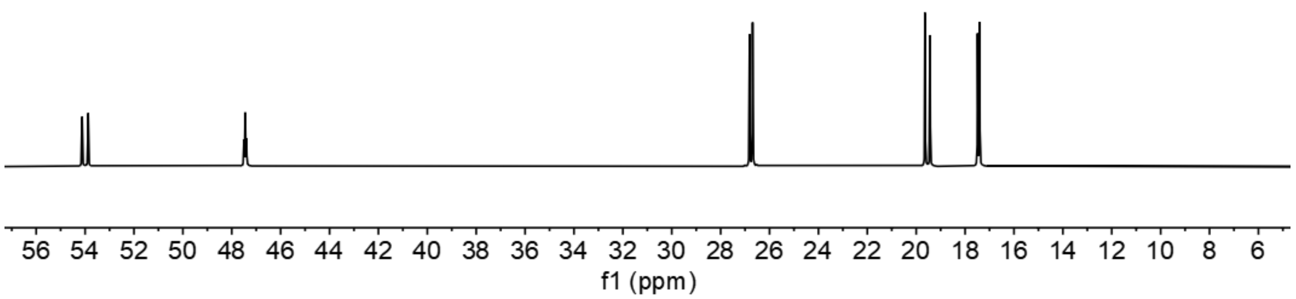

Supplementary Figure 90. Zoom-in of the $^{13}C\{^1H\}$ NMR (D_6-benzene, 298 K) spectrum of **13**.

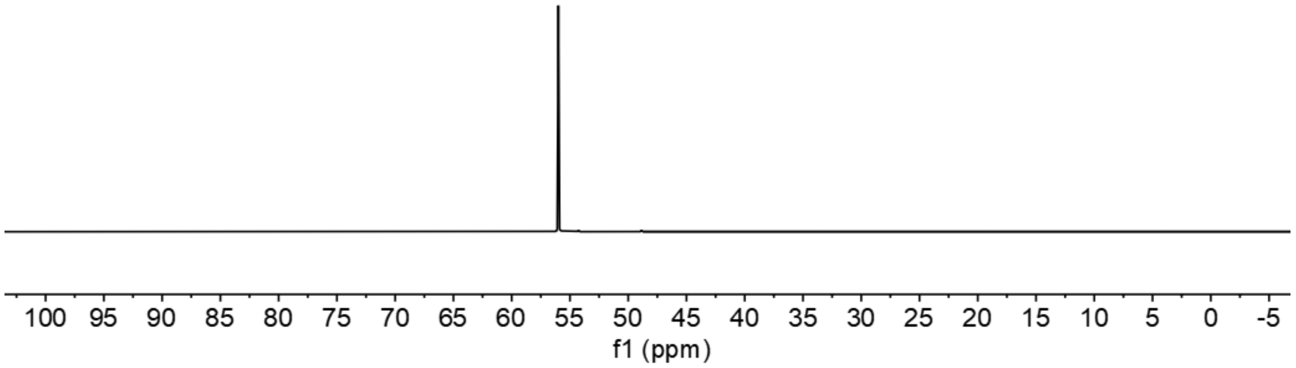

Supplementary Figure 91. $^{31}P\{^1H\}$ NMR (D_6-benzene, 298 K) spectrum of **13**.

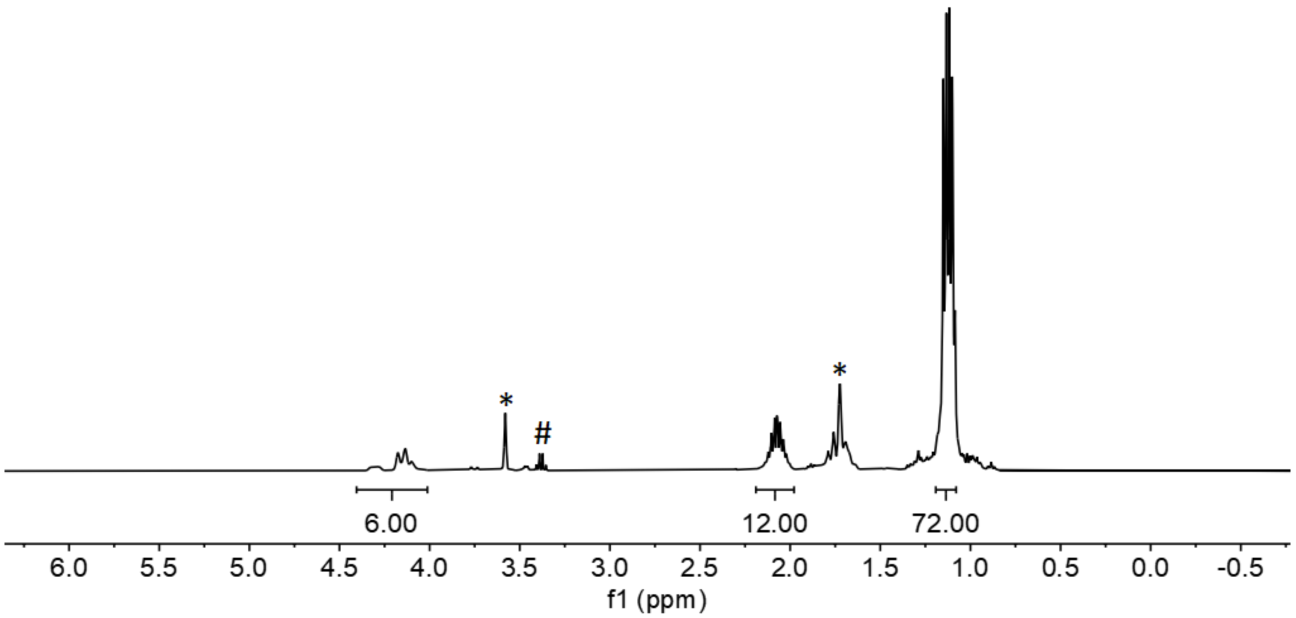

Supplementary Figure 92. ^{1}H NMR (D_8-THF, 298 K) spectrum of **14**. The hash (#) at ~3.38 denotes trace Et_2O; * = THF solvent. The resonance attributed to the 12 protons of CH_2-Cy could not be definitively assigned or integrated due to overlapping in the region 1.83−1.61 ppm with peaks from the D_8-THF solvent used for the NMR experiments.

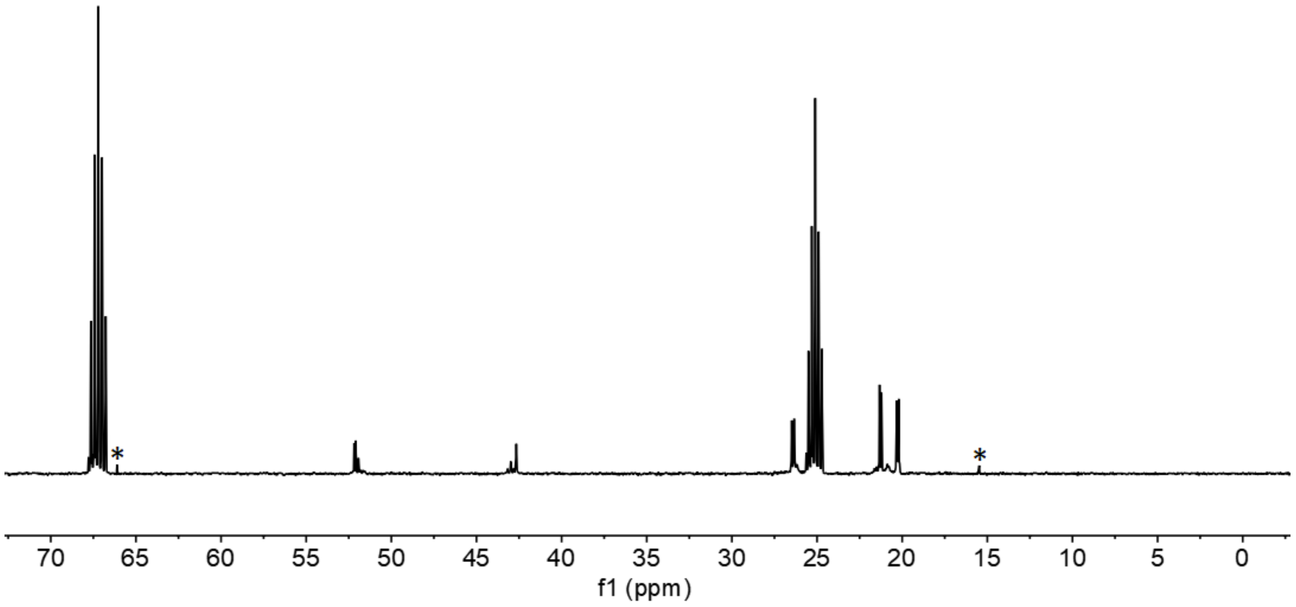

Supplementary Figure 93. $^{13}C\{^{1}H\}$ NMR (D_8-THF, 298 K) spectrum of **14**. The asterisks (*) at ~56.14 and 15.49 ppm denotes trace Et_2O.

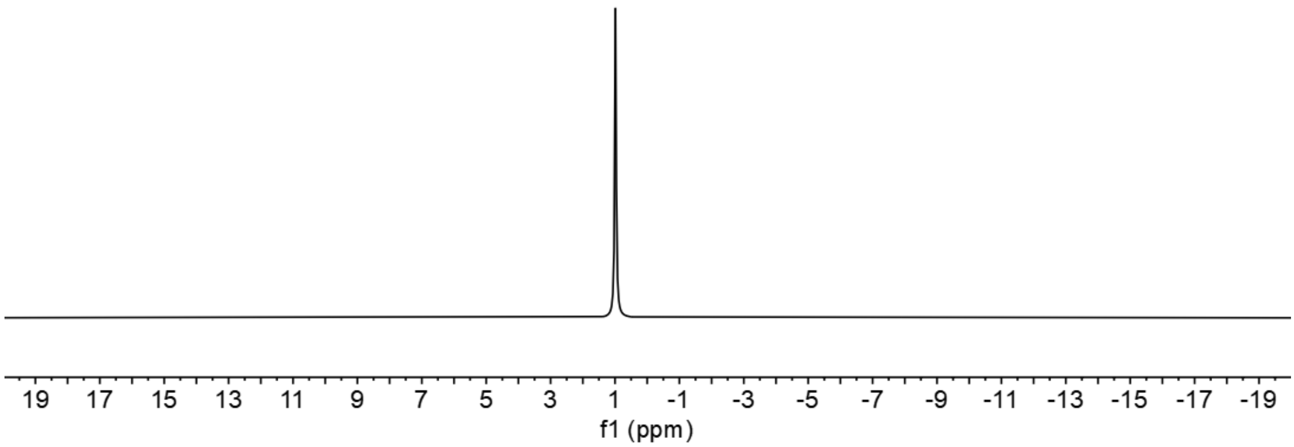

Supplementary Figure 94. $^{7}Li\{^{1}H\}$ NMR (D_8-THF, 298 K) spectrum of **14**.

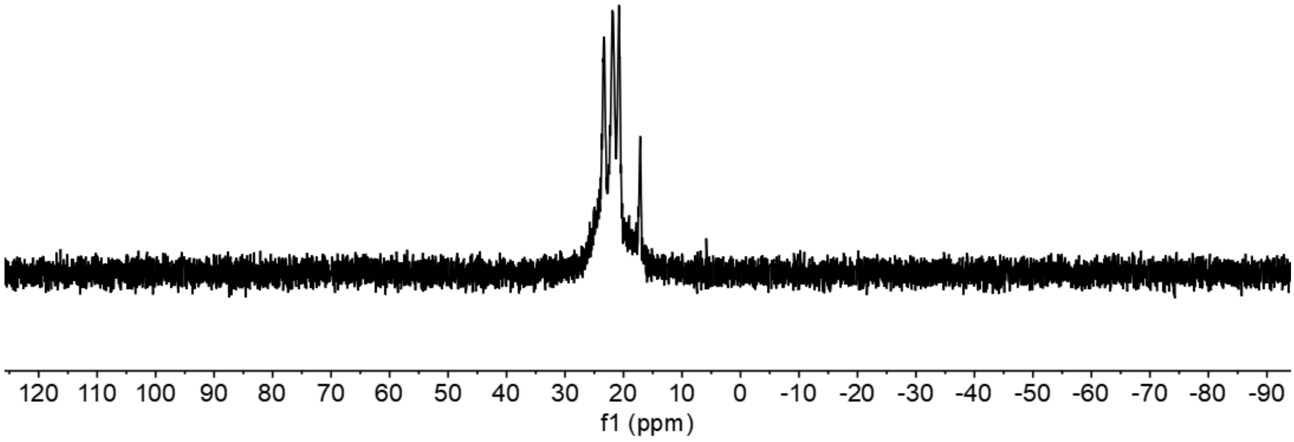

Supplementary Figure 95. $^{31}P\{^{1}H\}$ NMR (D_8-THF, 298 K) spectrum of **14**.

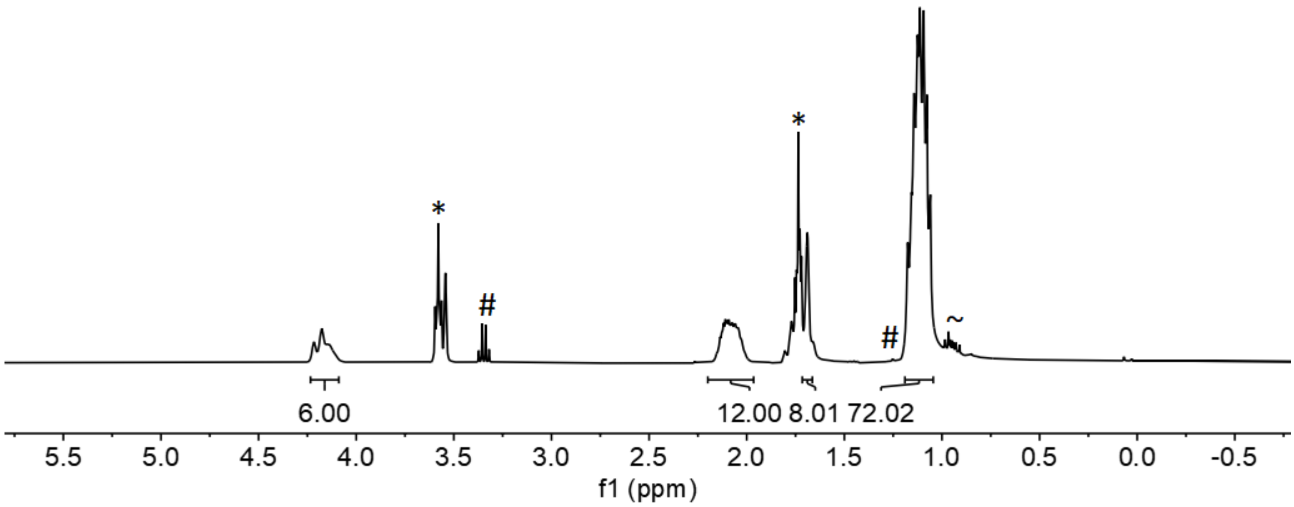

Supplementary Figure 96. ^{1}H NMR (D_8-THF, 298 K) spectrum of **15**. The hash (#) at ~3.38 and 1.12 ppm denotes trace Et_2O; * = THF solvent; ~ = residual **13**. The resonances attributed to the 8 protons of CH_2-THF, and 12 protons of CH_2-Cy, could not be definitively assigned or integrated due to overlapping in the regions ~3.58 and 1.78–1.72 ppm with peaks from the D_8-THF solvent used for the NMR experiments.

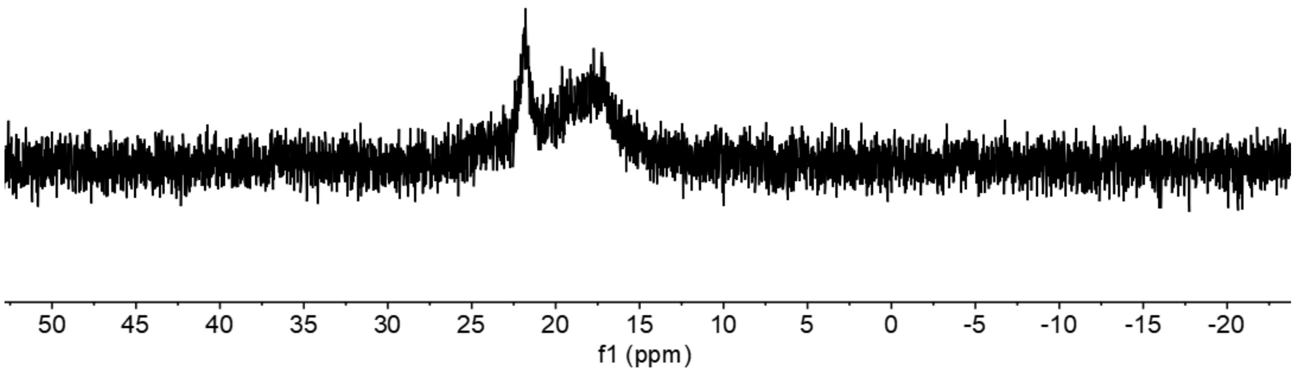

Supplementary Figure 97. $^{31}P\{^{1}H\}$ NMR (D_8-THF, 298 K) spectrum of **15**.

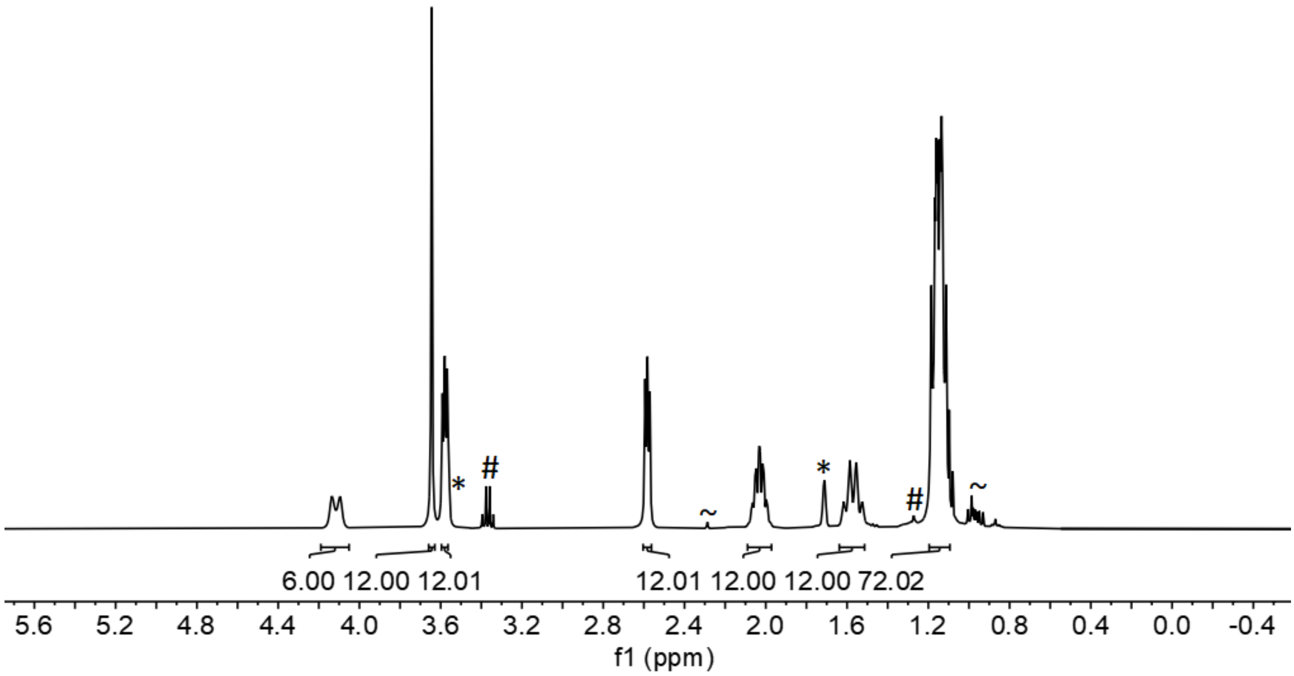

Supplementary Figure 98. ^{1}H NMR (D_8-THF, 298 K) spectrum of **16**. The hash (#) at ~3.38 and 1.12 ppm denotes trace Et_2O; * = THF solvent; ~ = residual **13**.

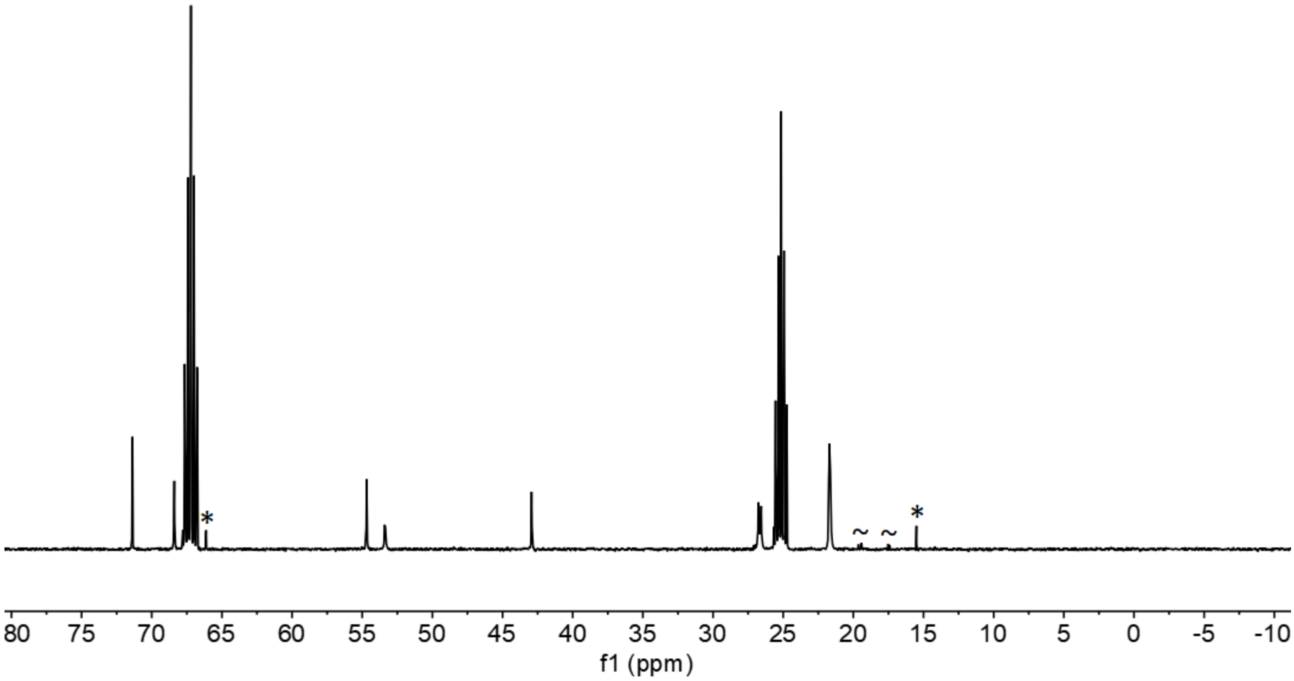

Supplementary Figure 99. $^{13}C\{^{1}H\}$ NMR (D_8-THF, 298 K) spectrum of **16**. The asterisks (*) at ~56.14 and 15.49 ppm denotes trace Et_2O; ~ = residual **13**.

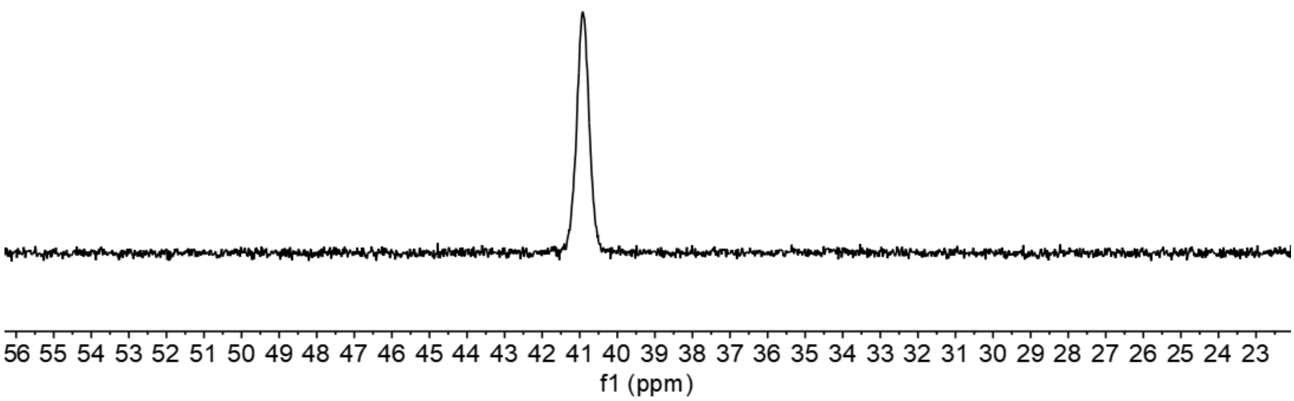

Supplementary Figure 100. $^{31}P\{^{1}H\}$ NMR (D_8-THF, 298 K) spectrum of **16**.

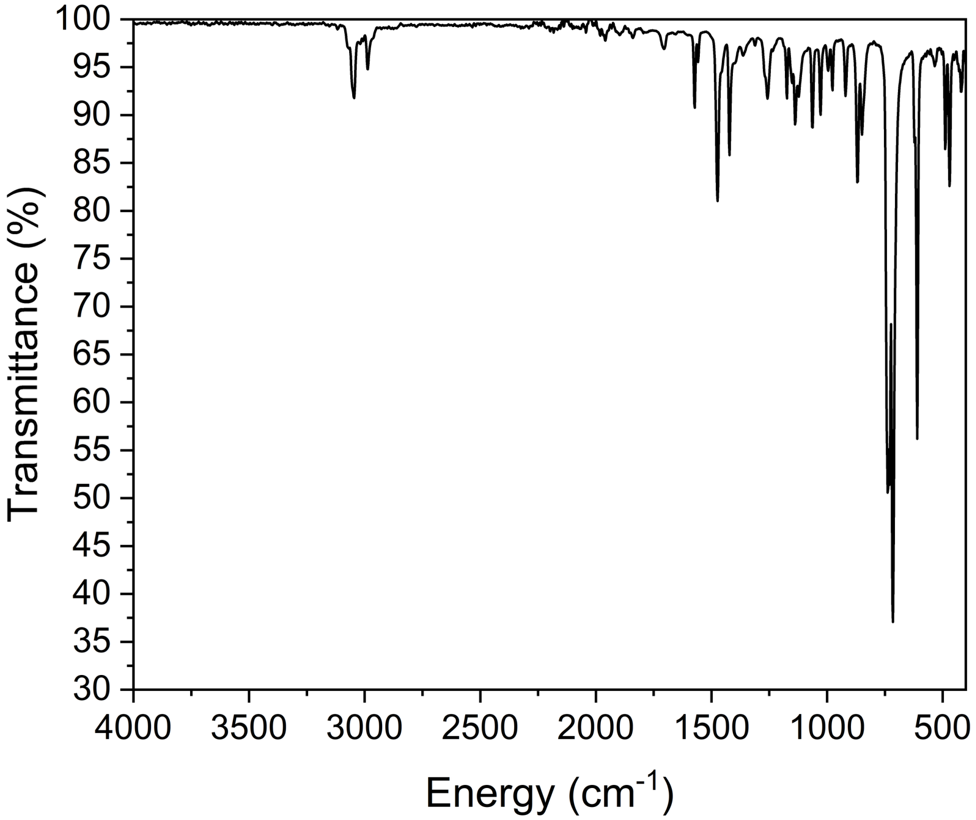

Supplementary Figure 101. ATR-IR spectrum of $AgBPh_4$.

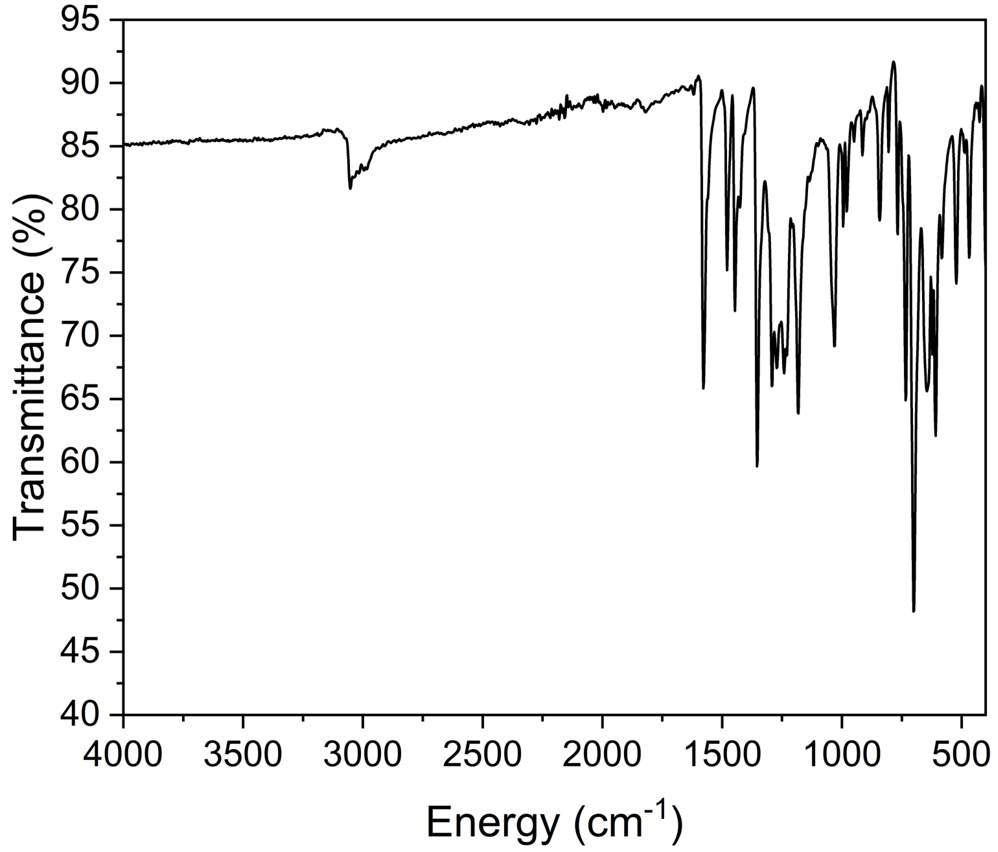

Supplementary Figure 102. ATR-IR spectrum of CPh_3BPh_4.

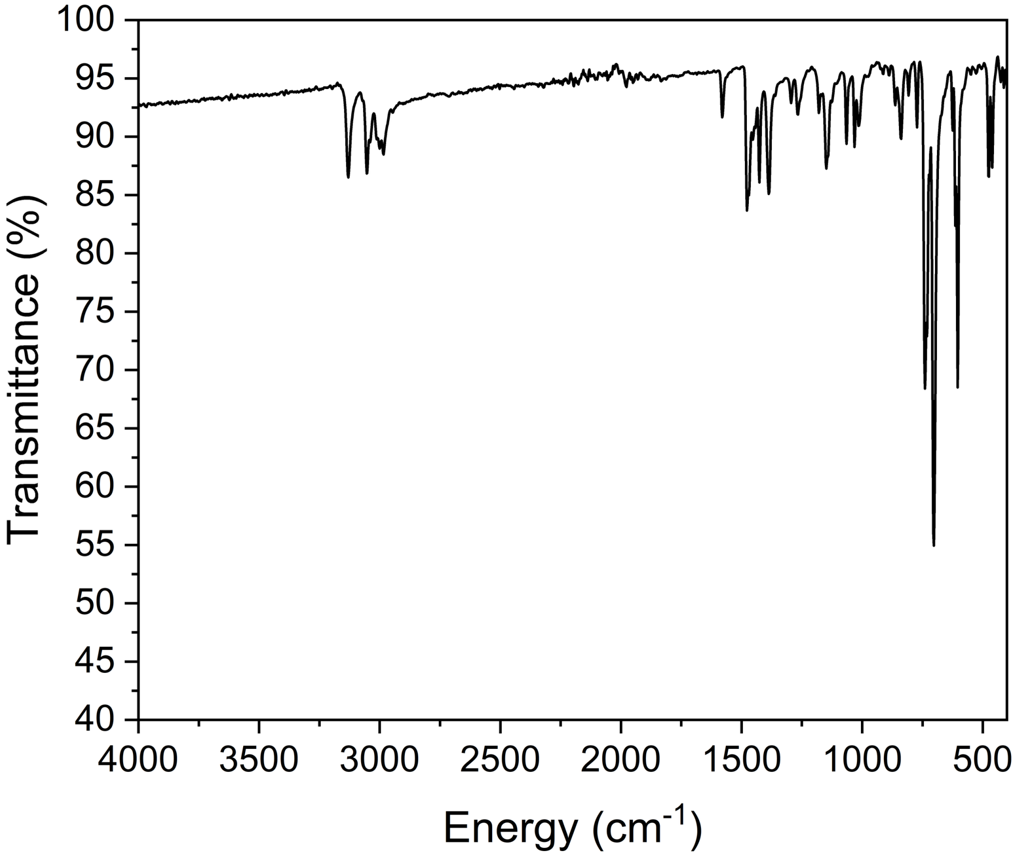

Supplementary Figure 103. ATR-IR spectrum of $[HNEt_3][BPh_4]$.

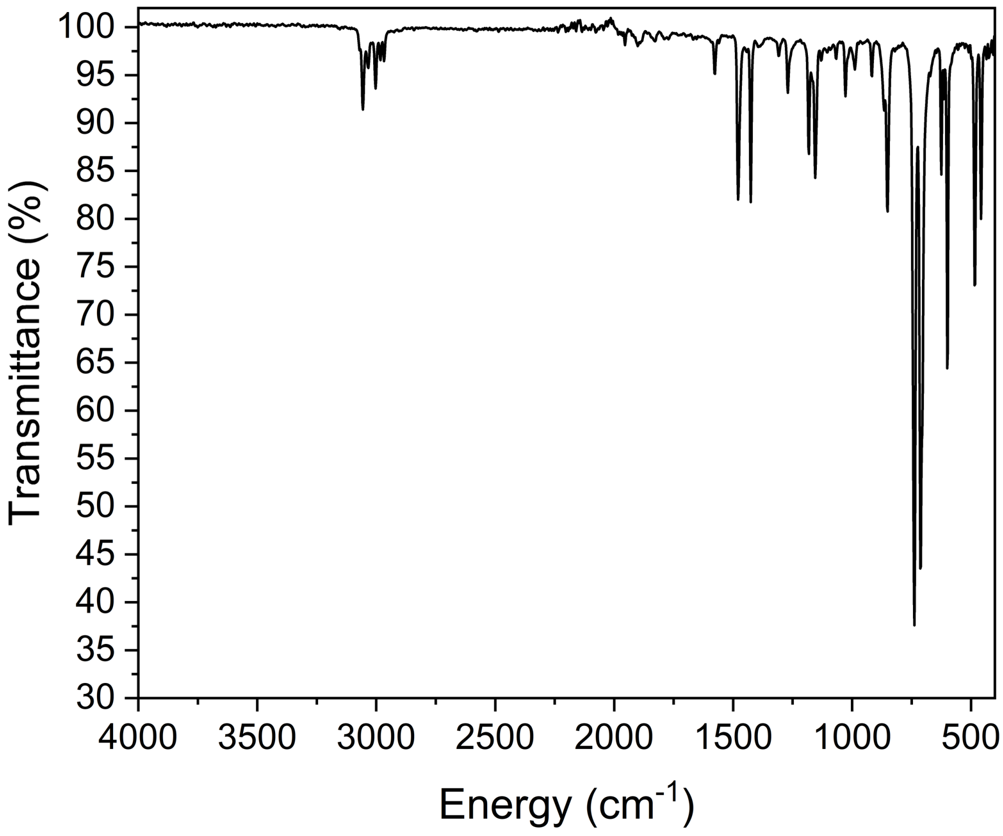

Supplementary Figure 104. ATR-IR spectrum of $TlBPh_4$.

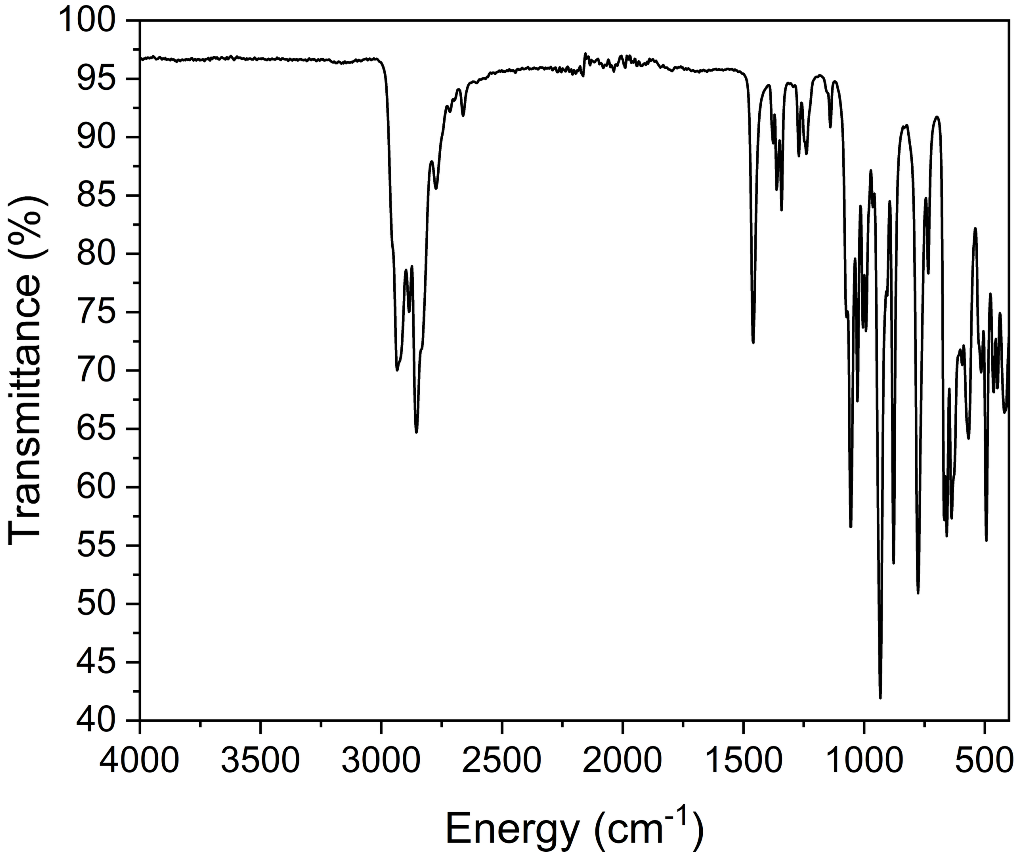

Supplementary Figure 105. ATR-IR spectrum of $Tren^{TIPS}Li_3$.

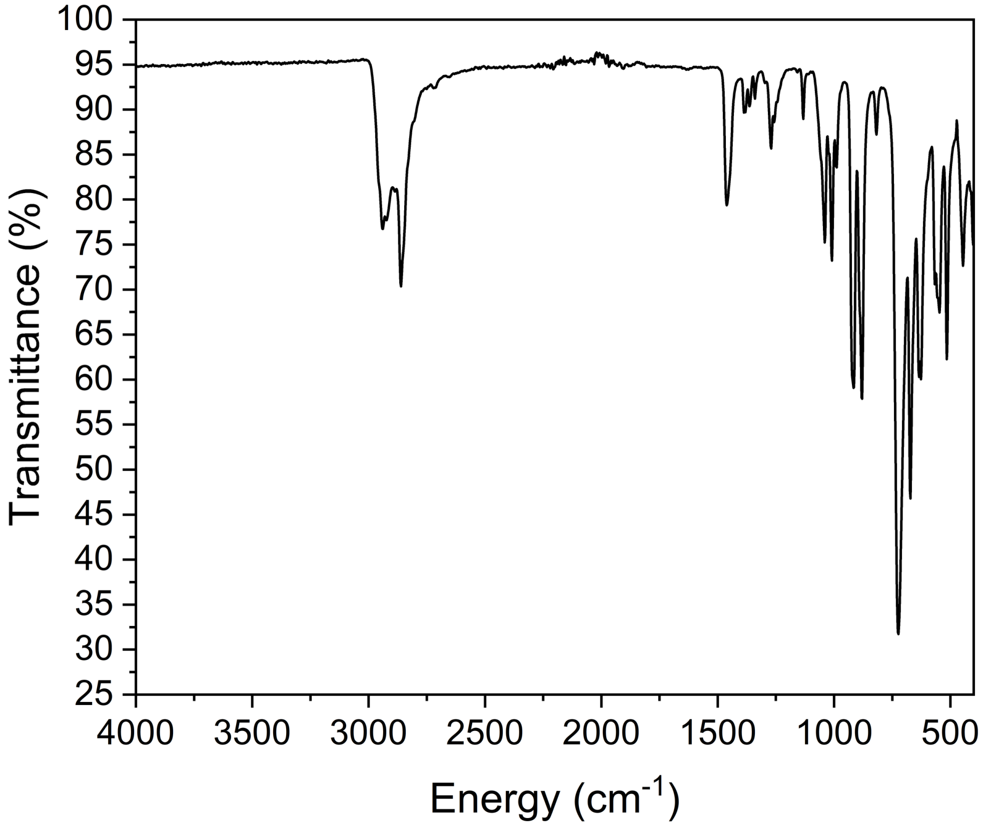

Supplementary Figure 106. ATR-IR spectrum of [U^{IV}($Tren^{TIPS}$)Cl].

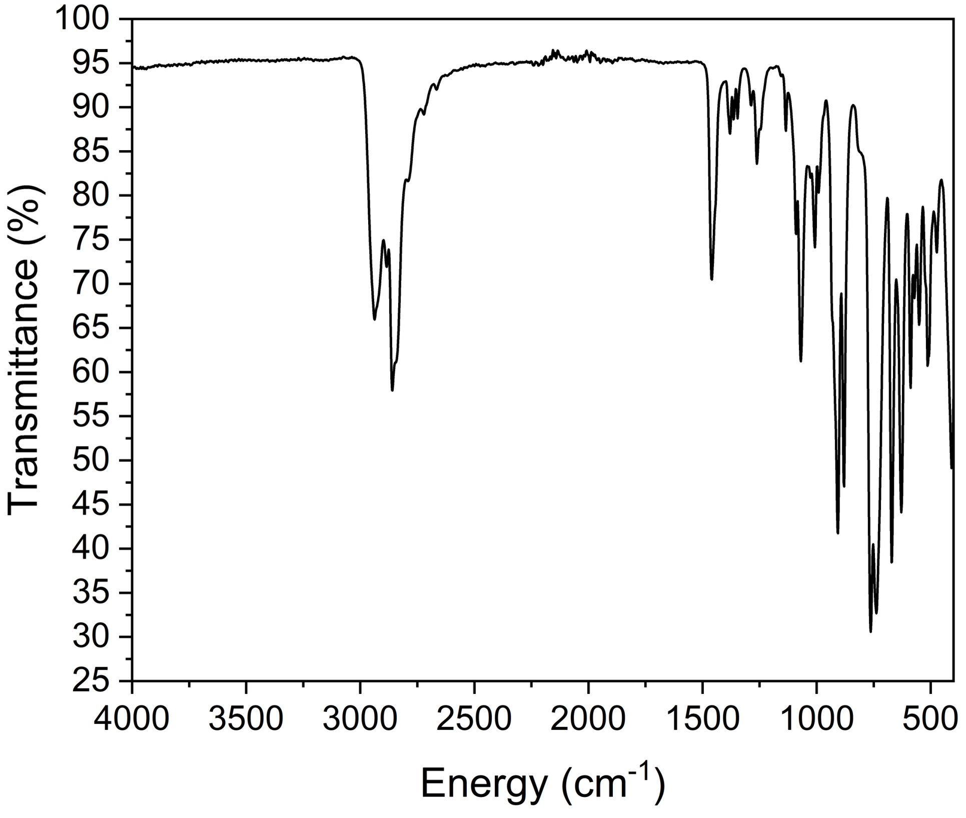

Supplementary Figure 107. ATR-IR spectrum of [U{N($CH_2CH_2NSiPr^i_3$)$_2$(CH_2CH_2Si-$Pr^i_2CHMeCH_2$)}].

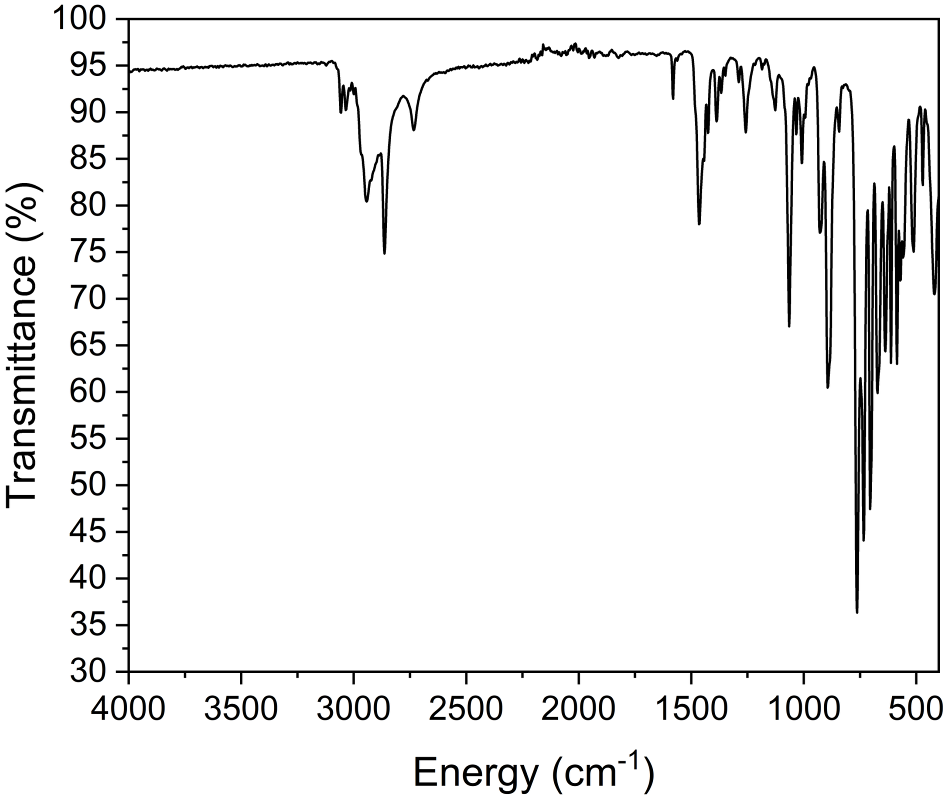

Supplementary Figure 108. ATR-IR spectrum of $[U^{IV}(Tren^{TIPS})(THF)][BPh_4]$.

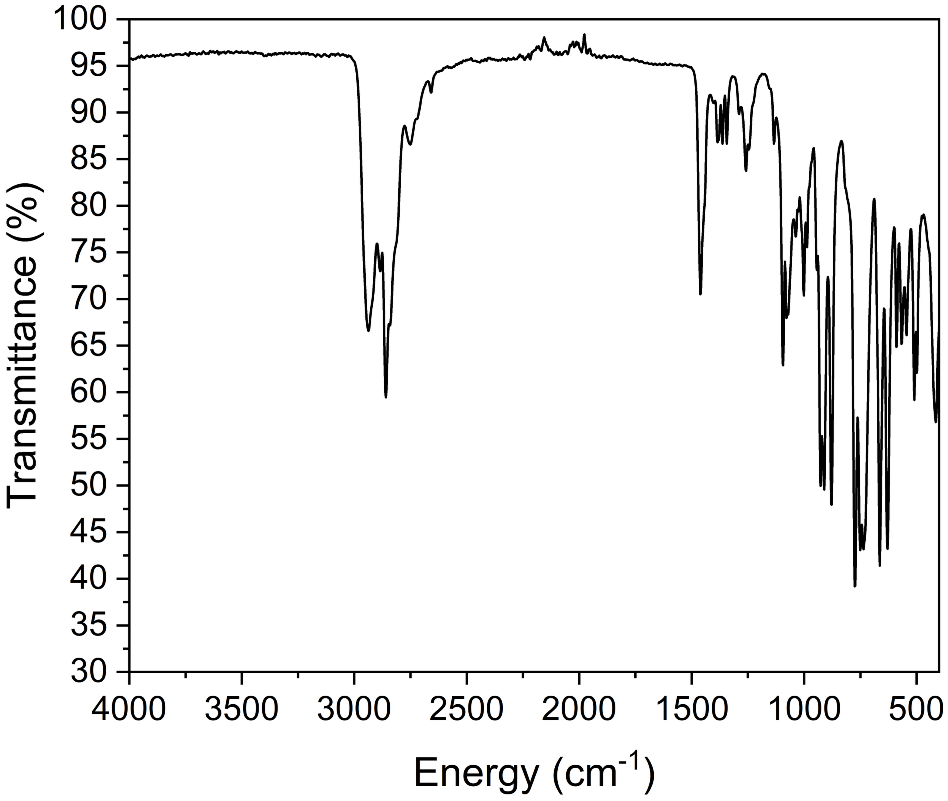

Supplementary Figure 109. ATR-IR spectrum of **1**.

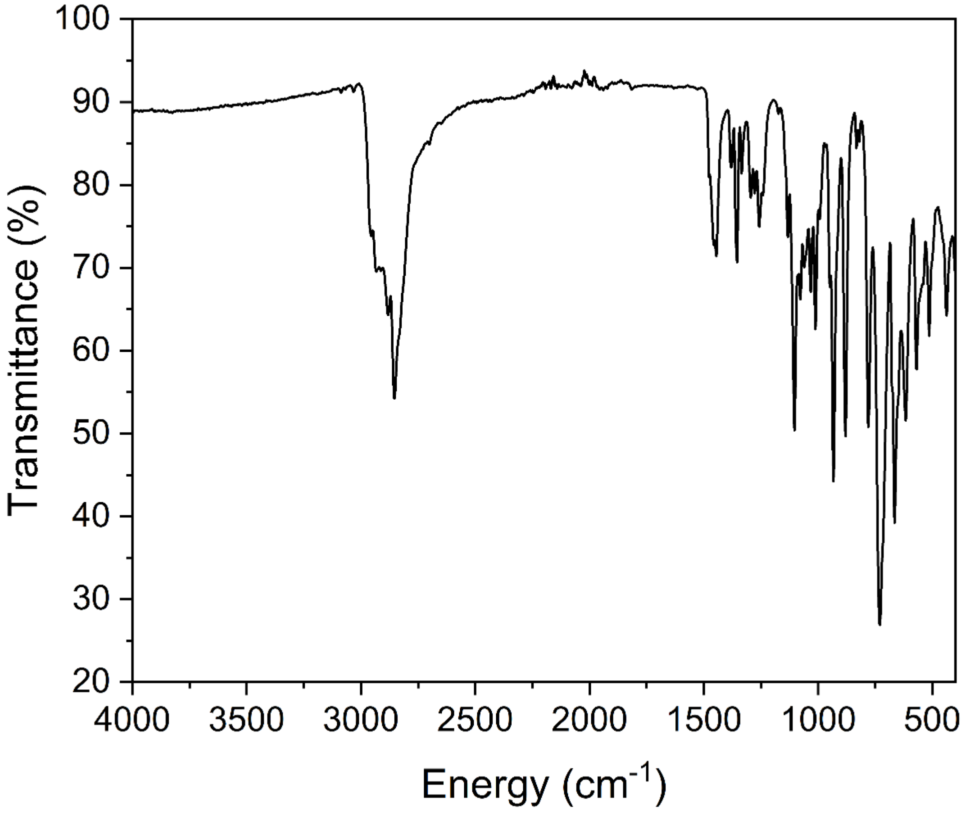

Supplementary Figure 110. ATR-IR spectrum of 9:1 **6a**:**6b**.

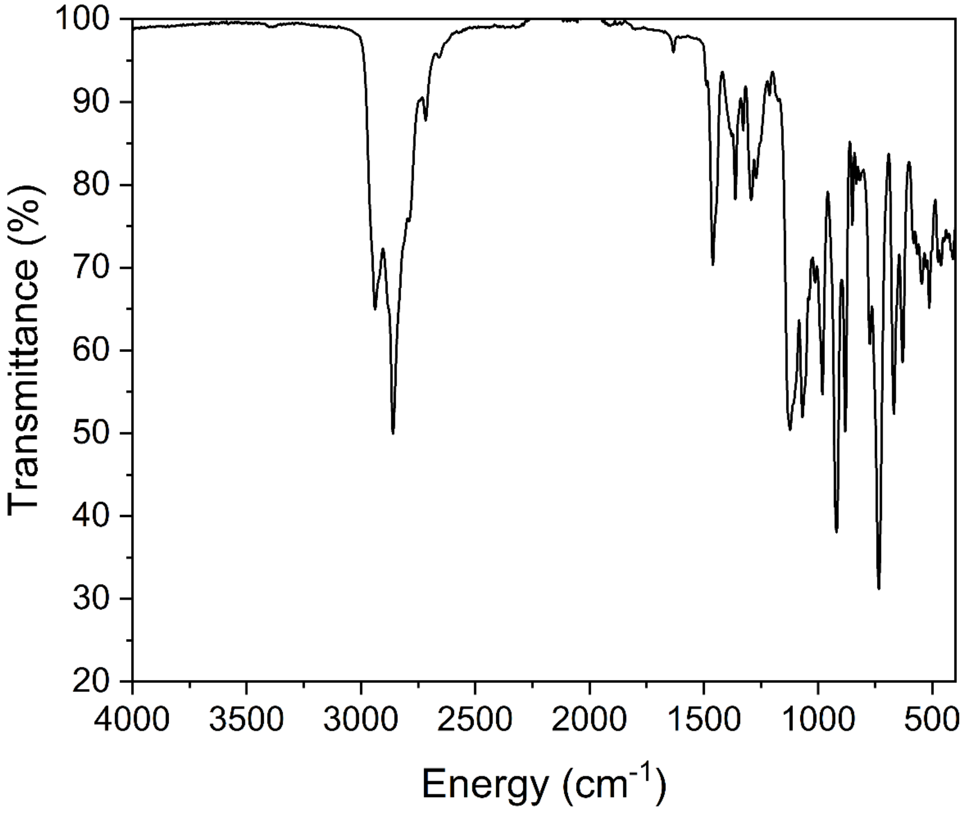

Supplementary Figure 111. ATR-IR spectrum of **8**.

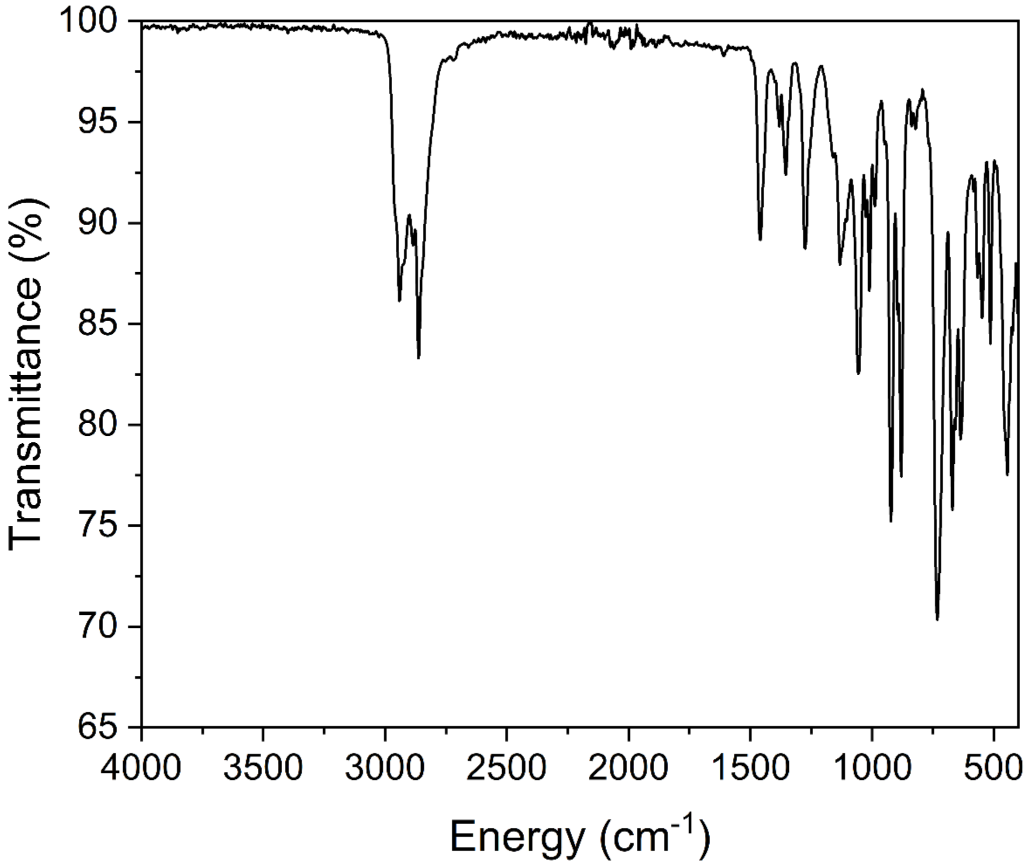

Supplementary Figure 112. ATR-IR spectrum of **9**.

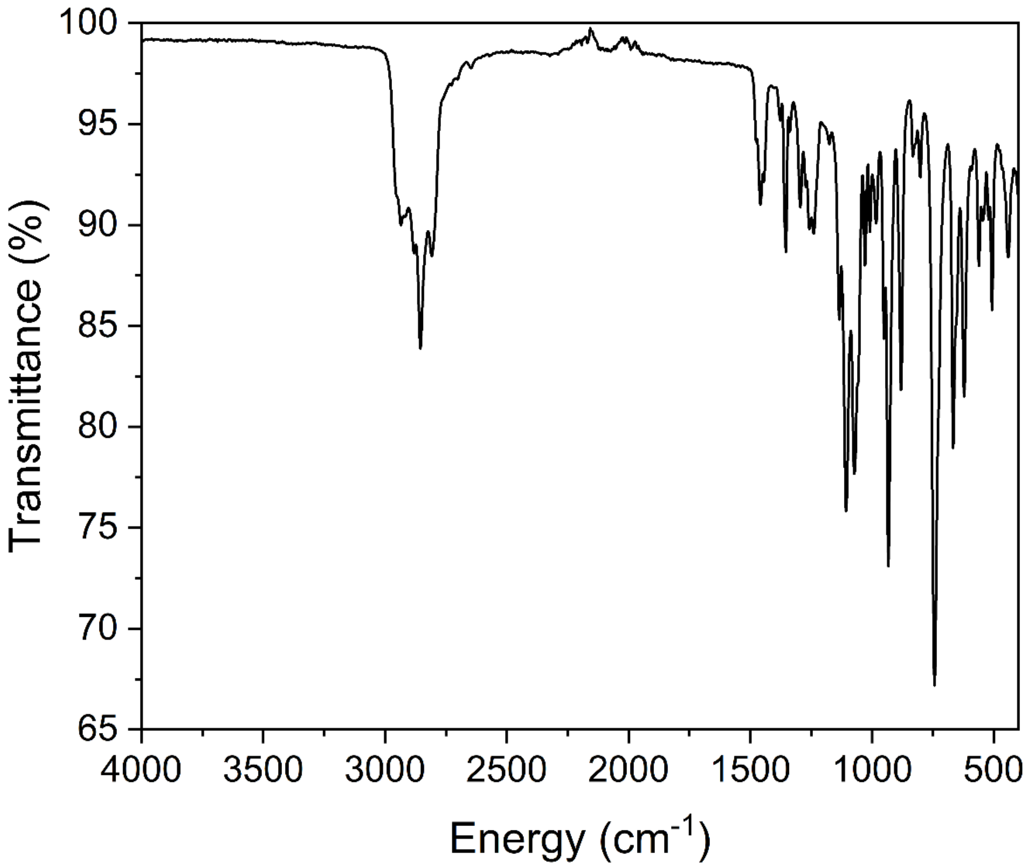

Supplementary Figure 113. ATR-IR spectrum of **12**.

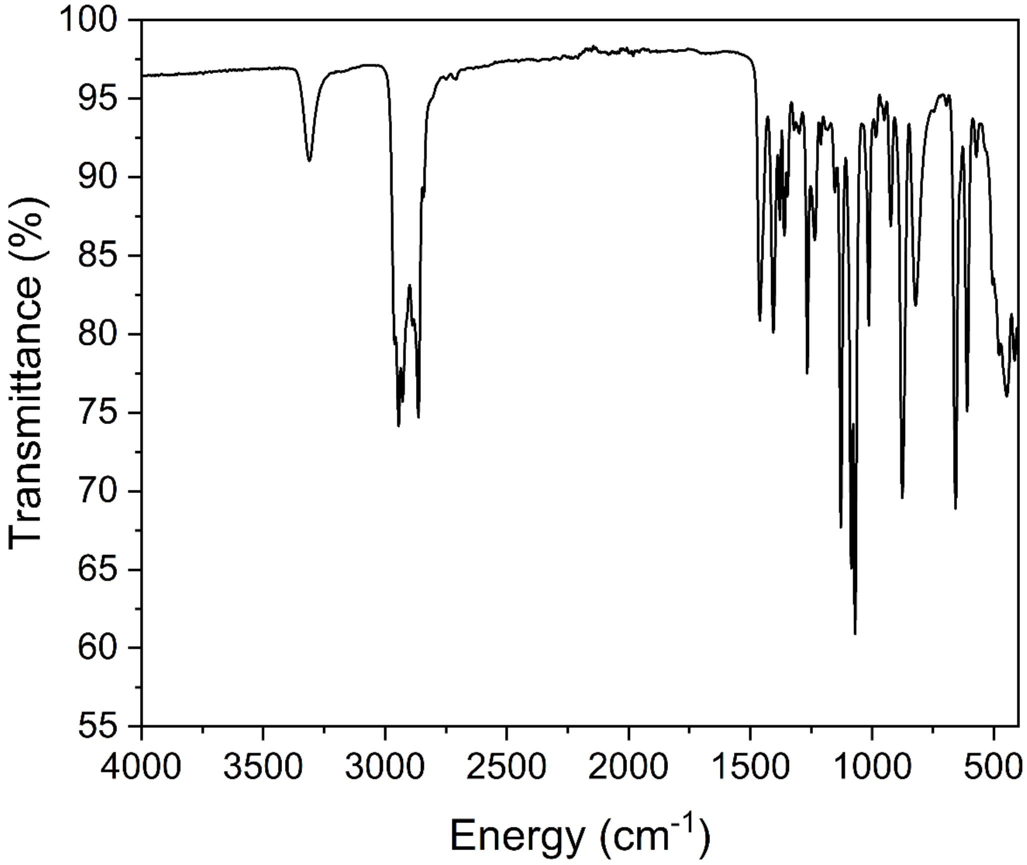

Supplementary Figure 114. ATR-IR spectrum of **13**.

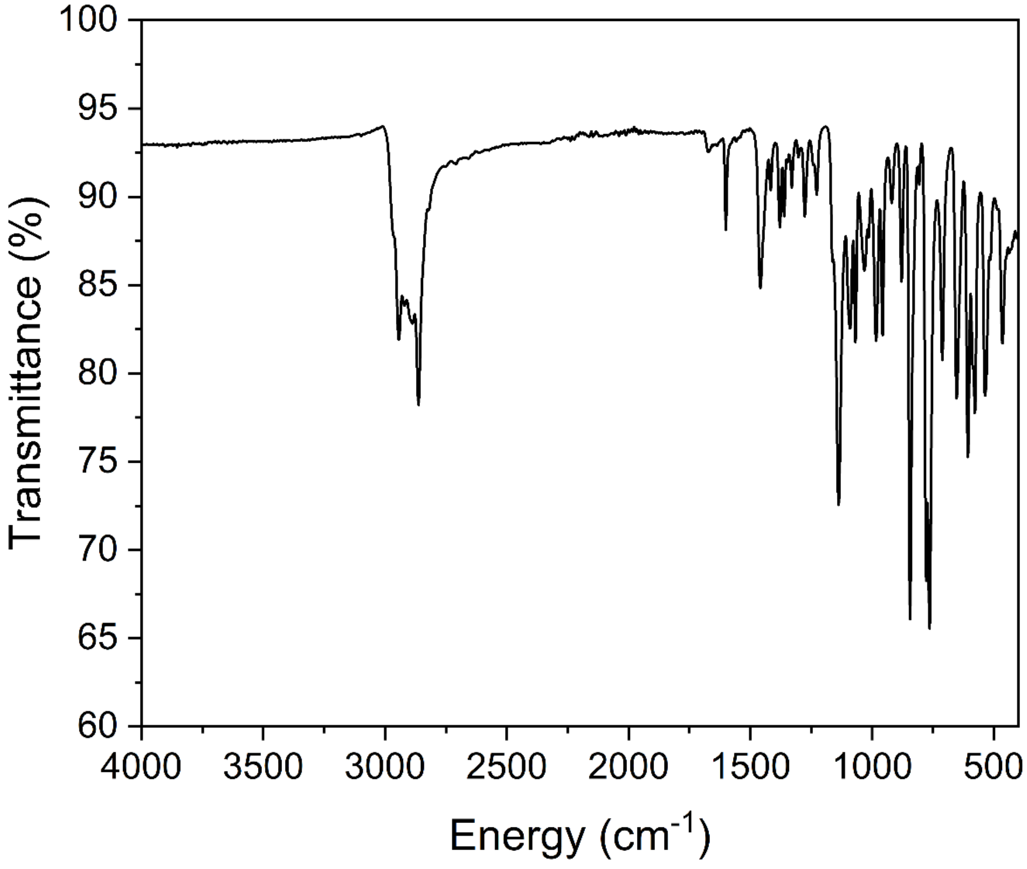

Supplementary Figure 115. ATR-IR spectrum of **14**.

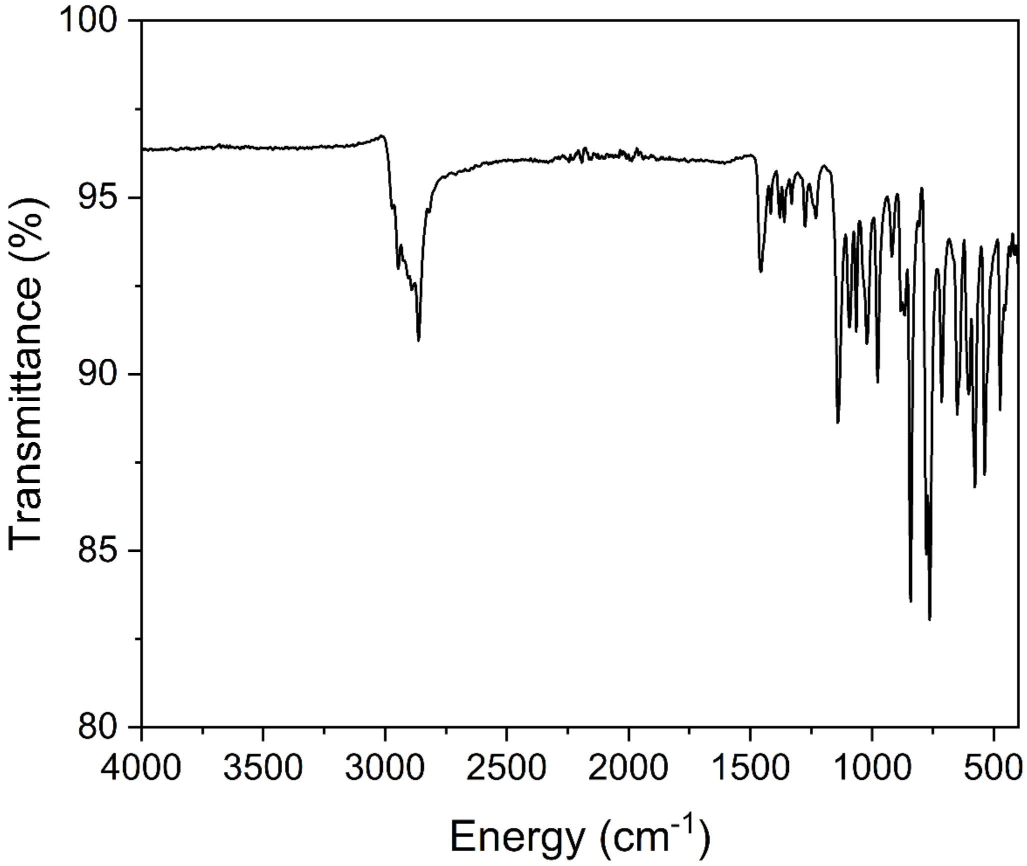

Supplementary Figure 116. ATR-IR spectrum of **15**.

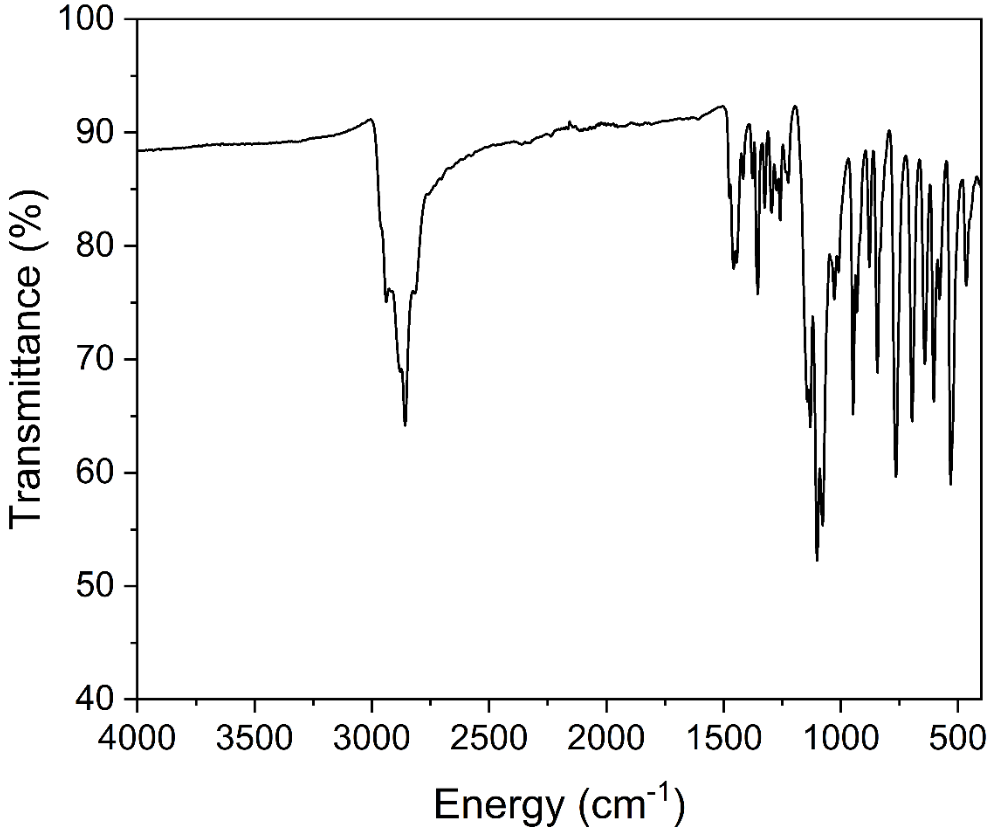

Supplementary Figure 117. ATR-IR spectrum of **16**.

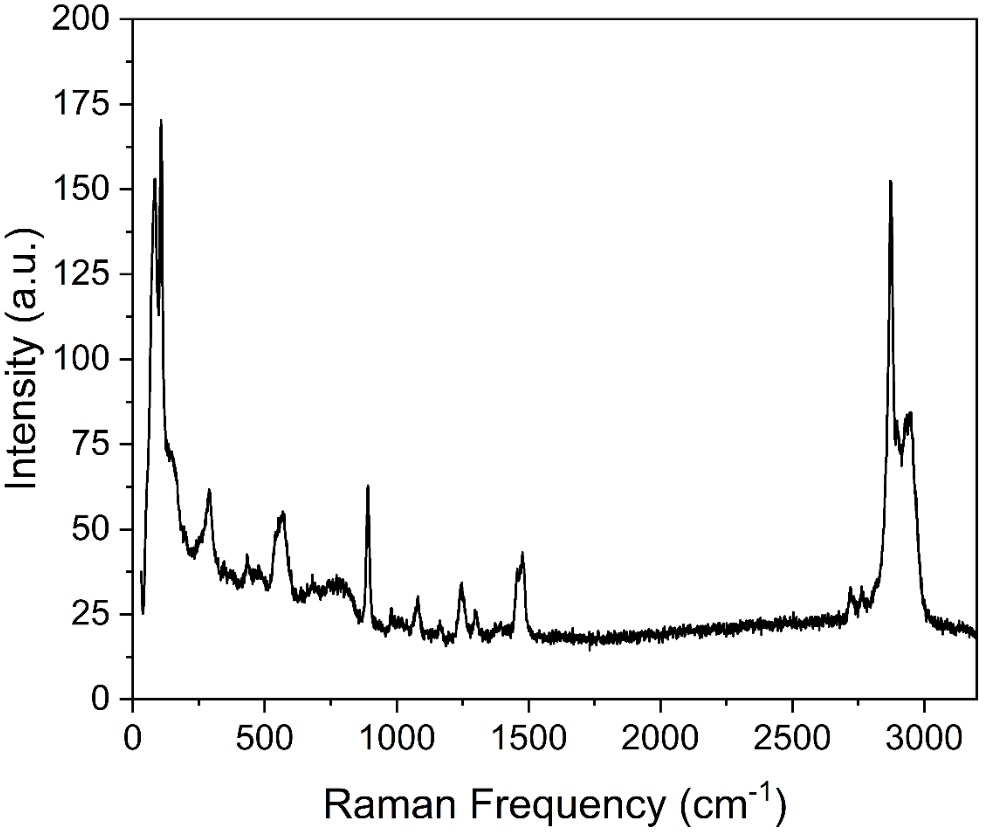

Supplementary Figure 118. Raman spectrum of **8** using a 638 nm laser.

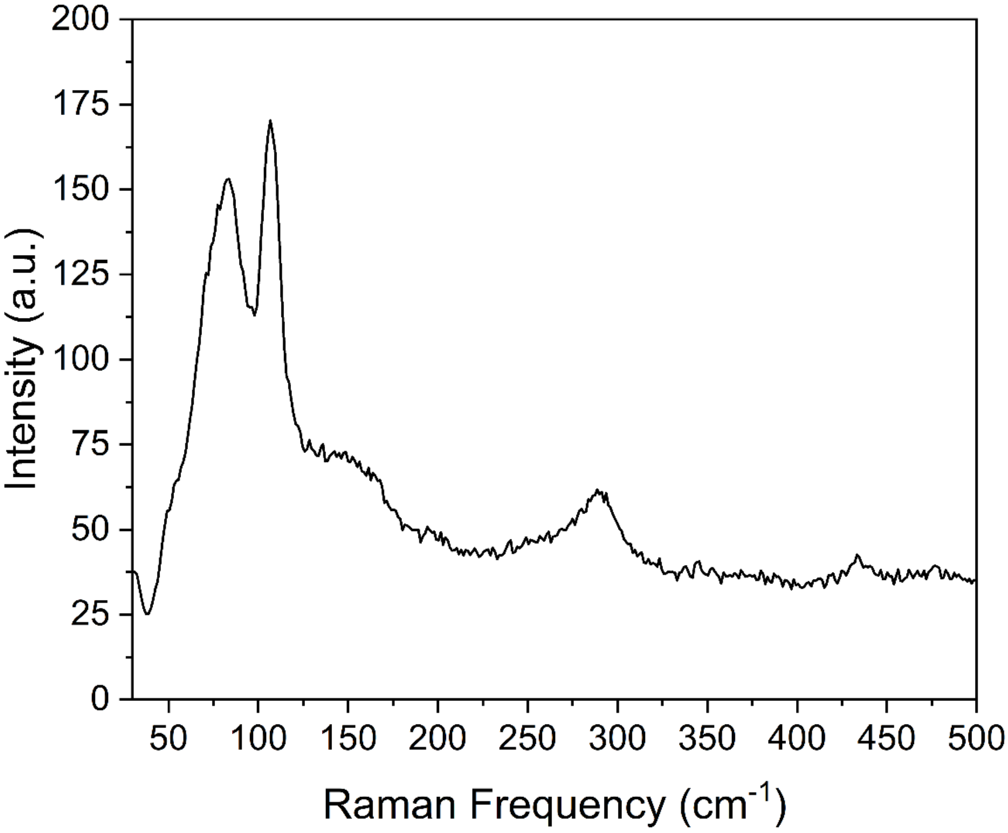

Supplementary Figure 119. Zoom-in of the Raman spectrum of **8** using a 638 nm laser.

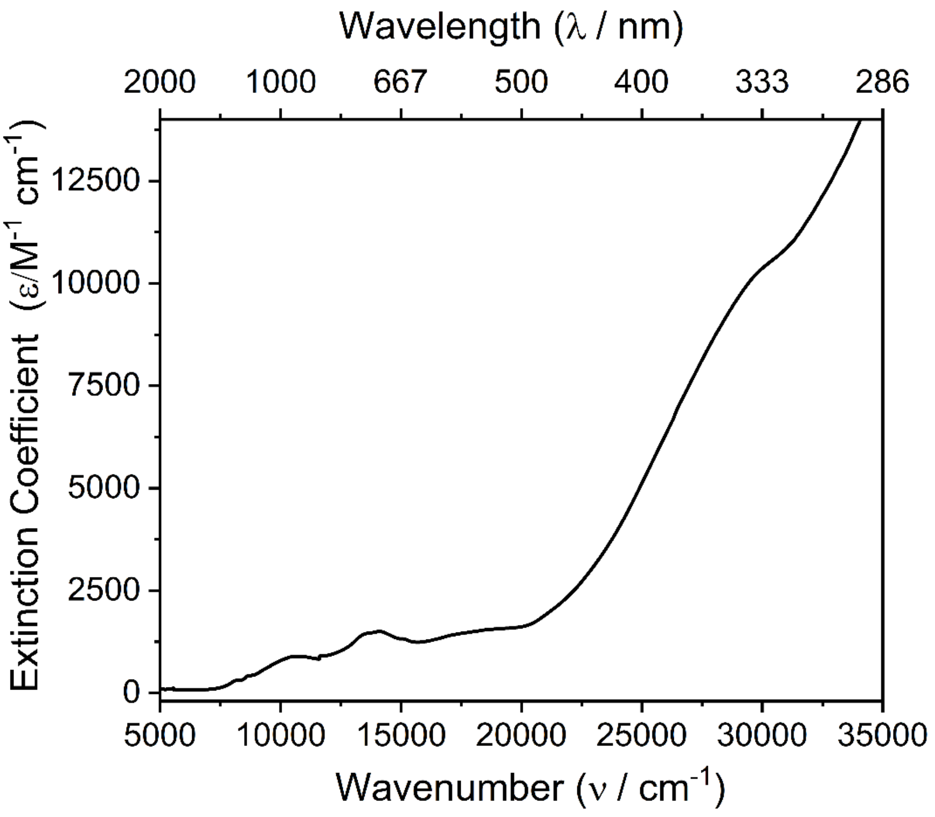

Supplementary Figure 120. UV/Vis/NIR spectra of 9:1 **6a**:**6b** in THF (2 mM).

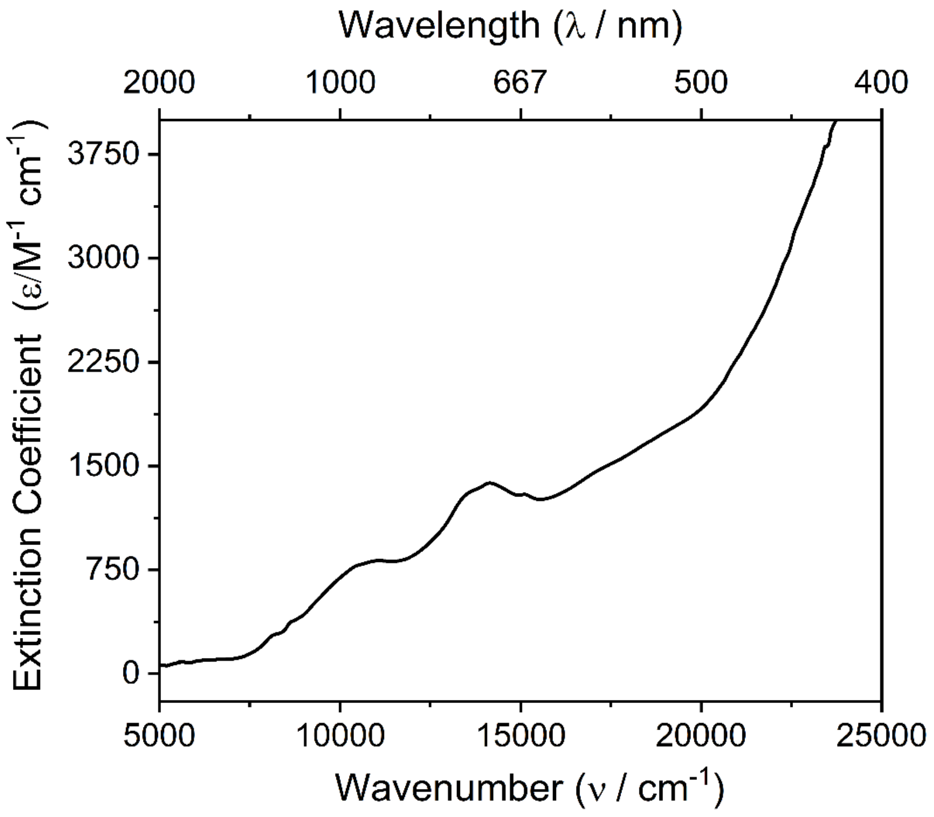

Supplementary Figure 121. UV/Vis/NIR spectra of 9:1 **6a**:**6b** in THF (10 mM).

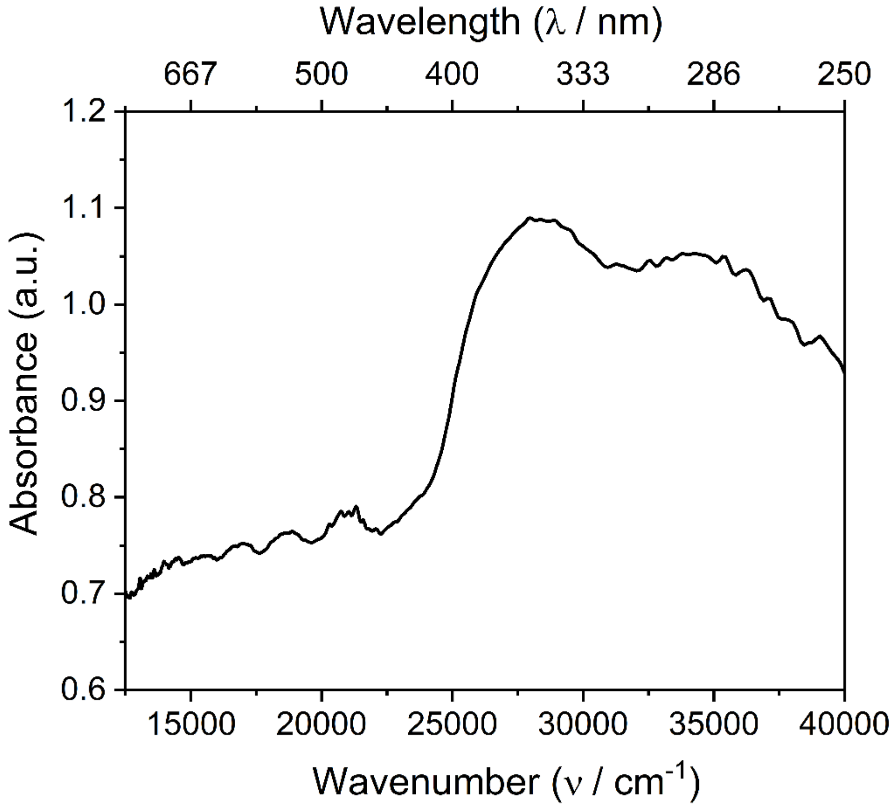

Supplementary Figure 122. Solid-state UV/Vis/NIR spectra of powdered 9:1 **6a**:**6b**.

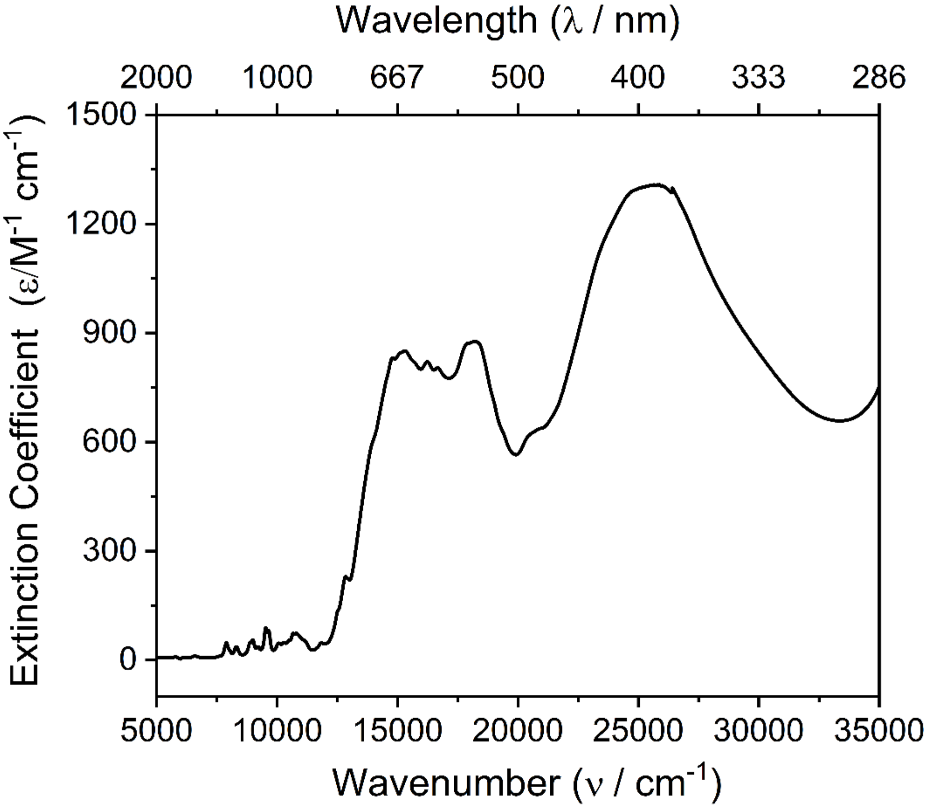

Supplementary Figure 123. UV/Vis/NIR spectra of **12** in benzene (18.53 mM).

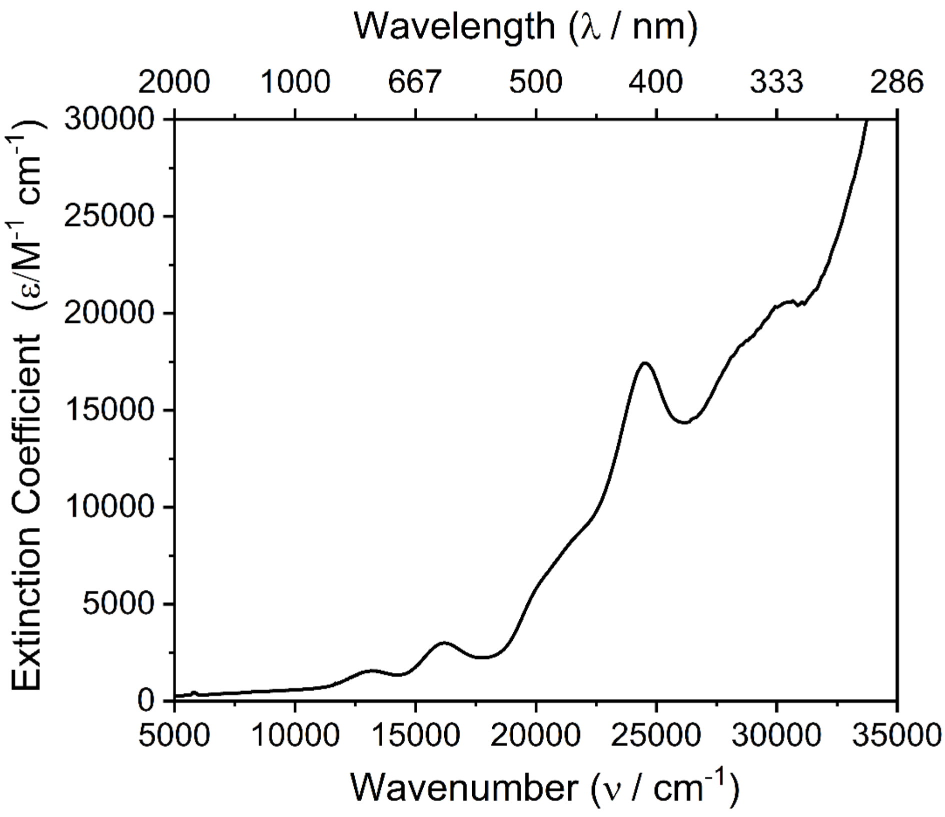

Supplementary Figure 124. UV/Vis/NIR spectra of **16** in THF (1 mM).

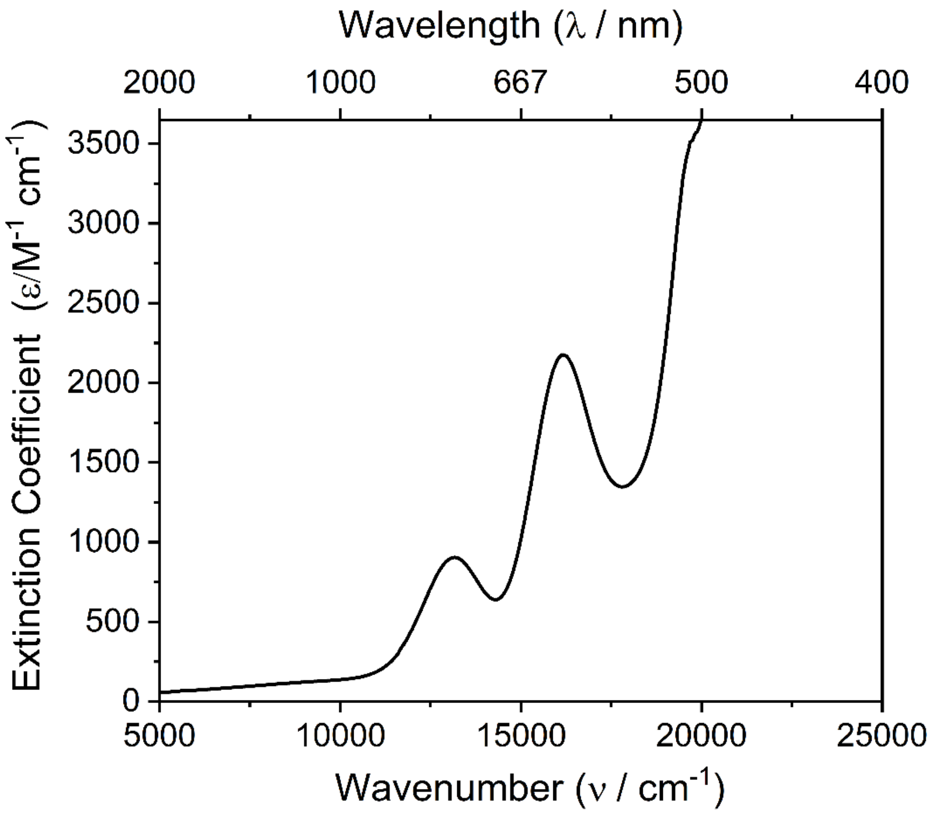

Supplementary Figure 125. UV/Vis/NIR spectra of **16** in THF (10 mM).

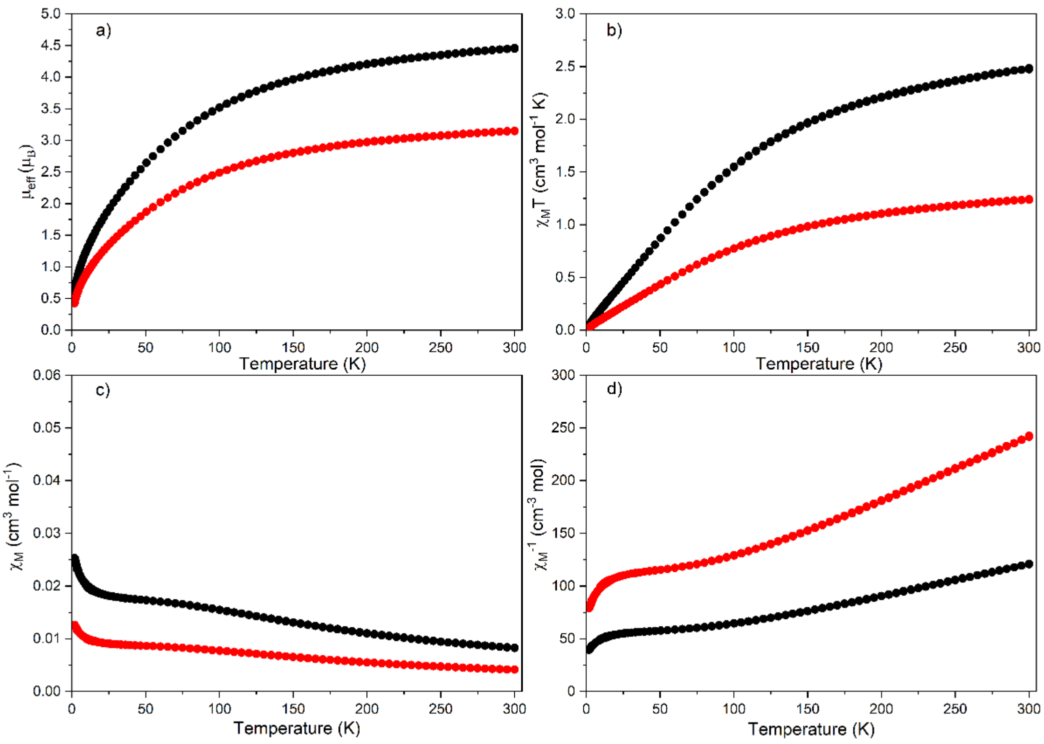

Supplementary Figure 126. Variable-temperature SQUID magnetometry for 9:1 **6a:6b** (per molecule: black; per U: red) over the temperature range 300-1.8 K in an external 0.5 T field: a) μ_{eff} vs T; b) χ_MT vs T; c) χ_M vs T; d) χ_M^{-1} vs T. The lines are a guide to the eye only.

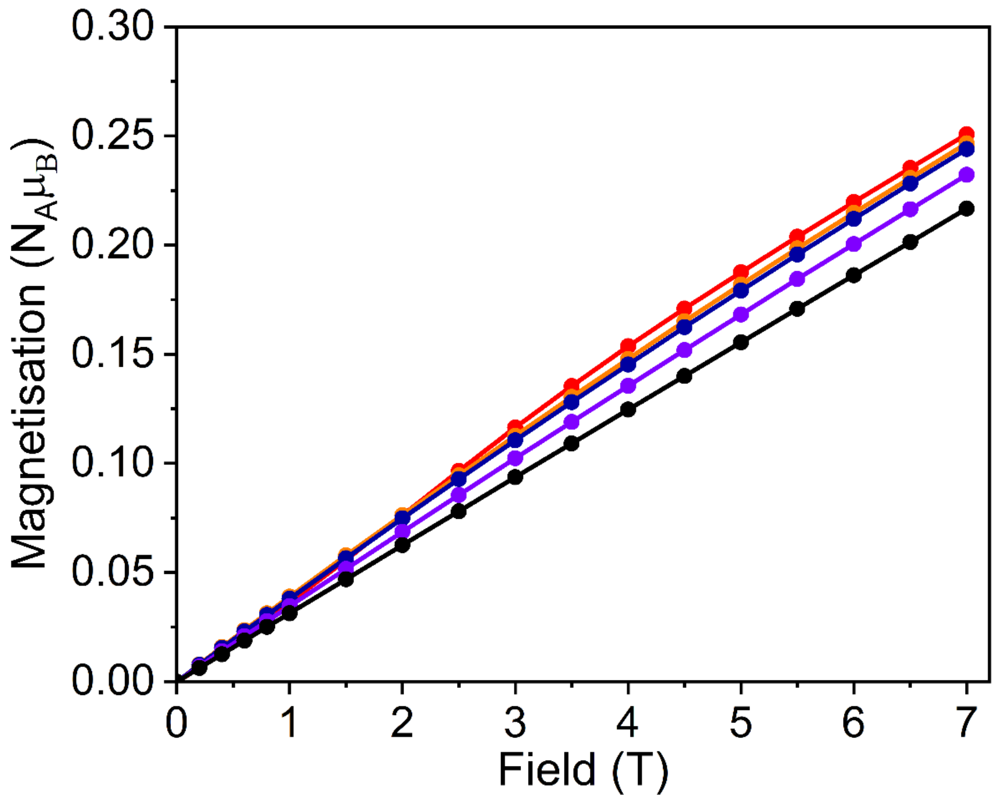

Supplementary Figure 127. Magnetisation vs Field data for 9:1 **6a**:**6b** at 2 (red), 4 (orange), 5 (blue), 10 (purple), and 20 K (black).

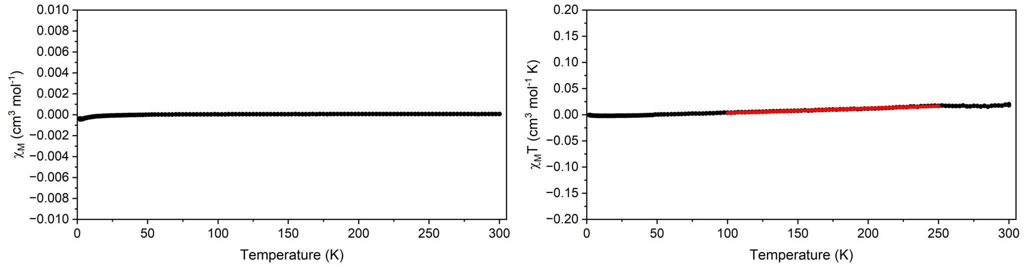

Supplementary Figure 128. Variable-temperature SQUID magnetometry of **16** at 0.1 T plotting χ_M (cm^3 mol^{-1}) vs Temperature (K) over the temperature range 1.8 to 300 K (left) and $\chi_M T$ (cm^3 mol^{-1} K) vs Temperature (K) over the temperature range 1.8 to 300 K (right). The red line represents a linear regression (250-100 K) whereby a χ_{TIP} value of 8.76 x 10^{-5} cm^3 mol^{-1} can be extracted.

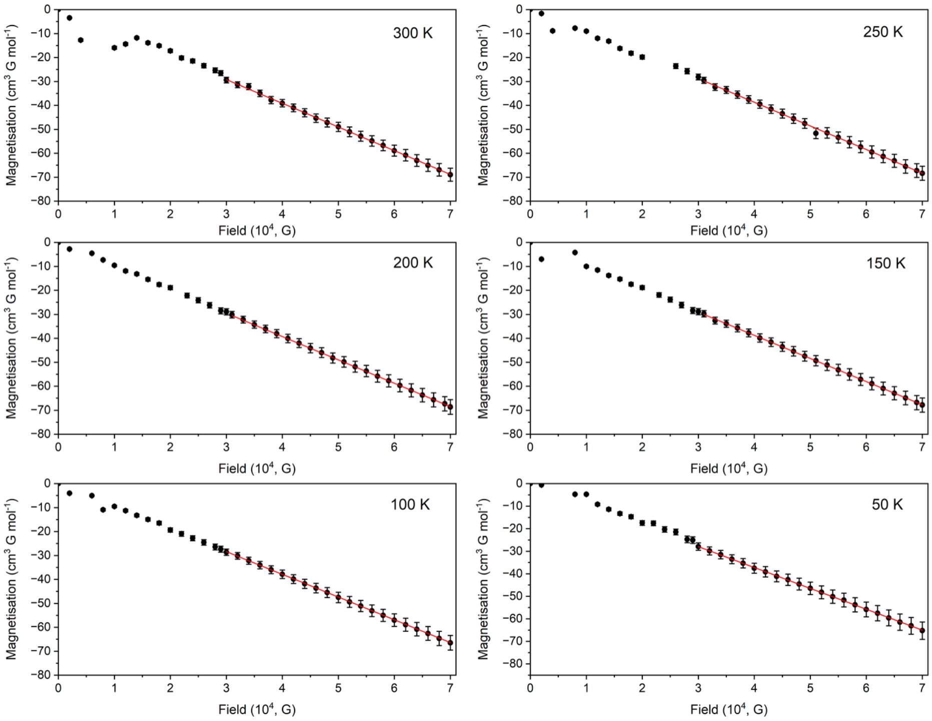

Supplementary Figure 129. Magnetisation vs Field data for **16** at 300 (top left: R^2 = 0.999; average error = 3.93 %), 250 (top right: R^2 = 0.999; average error = 4.28 %), 200 (middle left: R^2 = 0.999; average error = 4.41 %), 150 (middle right: R^2 = 0.999; average error = 4.36 %), 100 (bottom left: R^2 = 0.998; average error = 4.60 %) and 50 (bottom right: R^2 = 0.998; average error = 5.91%) K from 0-7 Tesla. The straight lines are linear regressions of the data points over the range 30,000 Gauss to 70,000 Gauss (10,000 Gauss = 1 Tesla). Errors derive from the standard deviation of the sampling distribution of the points used (sample population divided by the square root of the number of samples) to compute the reported moment represented as a percentage.

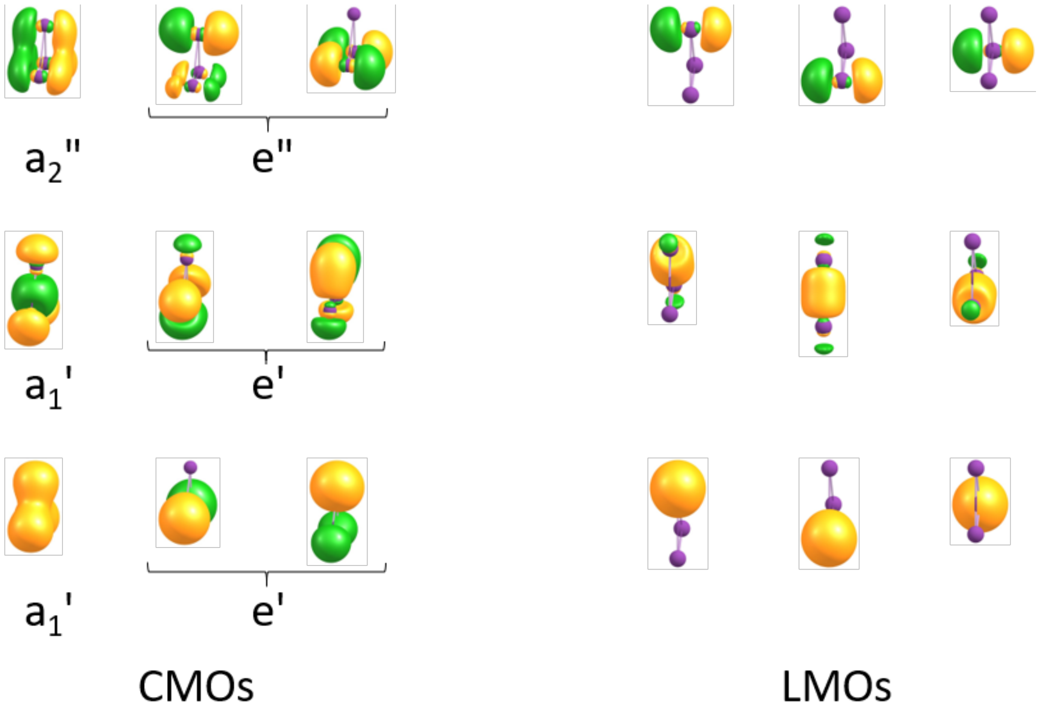

Supplementary Figure 130. Canonical MOs of Bi_3^{3-}, left, and localised MOs from a Pipek-Mezey localisation procedure, right. Contours are drawn at 0.05 a.u.

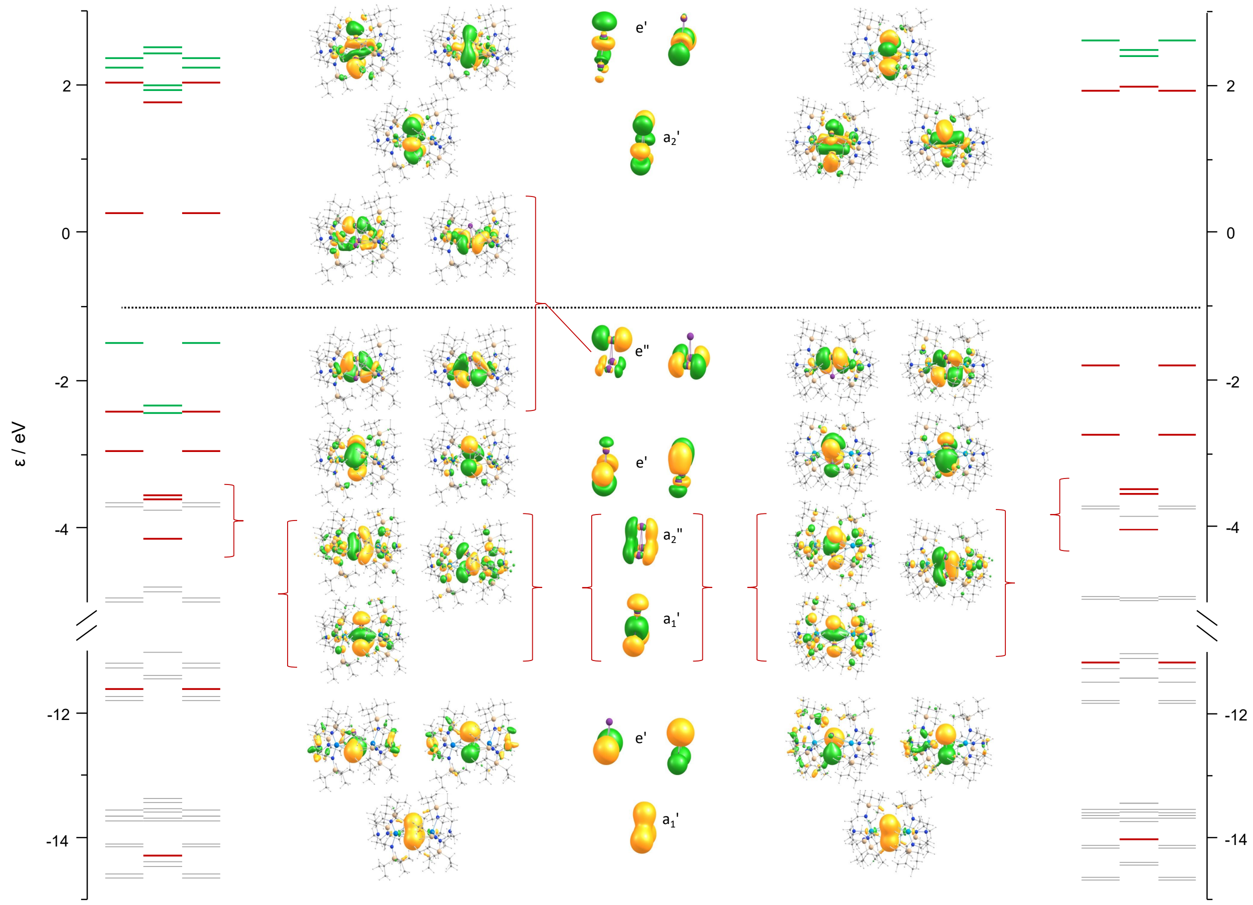

Supplementary Figure 131. Energies and shapes of occupied canonical molecular orbitals of 6a', left, *cyclo*-Bi_3^{3-}, centre, and 6a'(Th), right. For **6a'** only the majority spin orbitals are shown for simplicity. In the energy level diagram, the molecular orbitals that are depicted are represented by red bars, the four MOs containing the 5f electrons of the two U atoms in **6a'** are green bars, and the other orbitals are grey bars. Contours are drawn at ±0.025 a.u. (±0.05 a.u. for bare *cyclo*-Bi_3^{3-}).

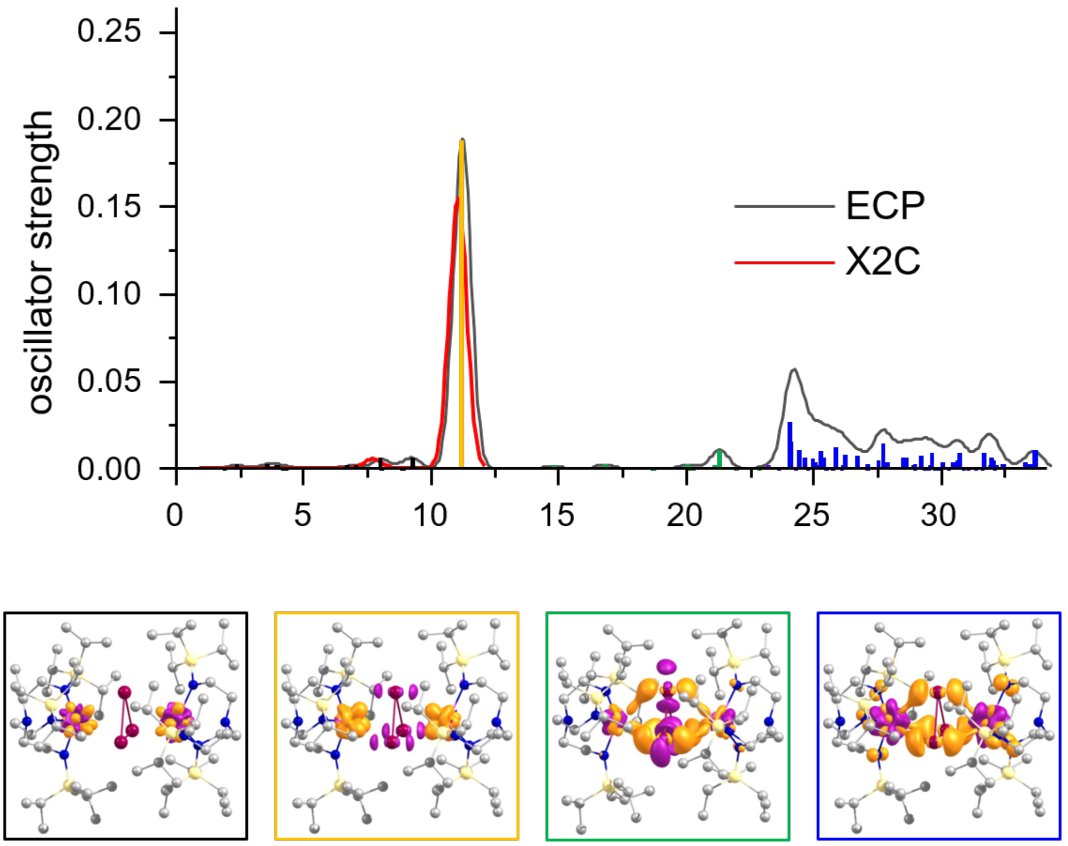

Supplementary Figure 132. Lowest spin-conserving excitations for **6a'** with TDDFT(PBE0). With ECPs, 50 excitations are calculated for both irrep a and irrep e, with X2C 10 per irrep, see also Tables S12 and S13. For ECPs, both individual excitations and superimposed Gaussians (FWHM=800cm^{-1}) are shown, for X2C only the latter. Composite excess density for the ground and excited states are plotted in orange and purple, respectively. Contours are drawn with ±0.001 a.u., and each colour denotes a group of excitations that constitute a composite that is energetically well separated from the other groups. The difference density plots provide an aggregated representation of the electron transfer for each excitation group containing weighted contributions of each excitation within the group by its oscillator strength.

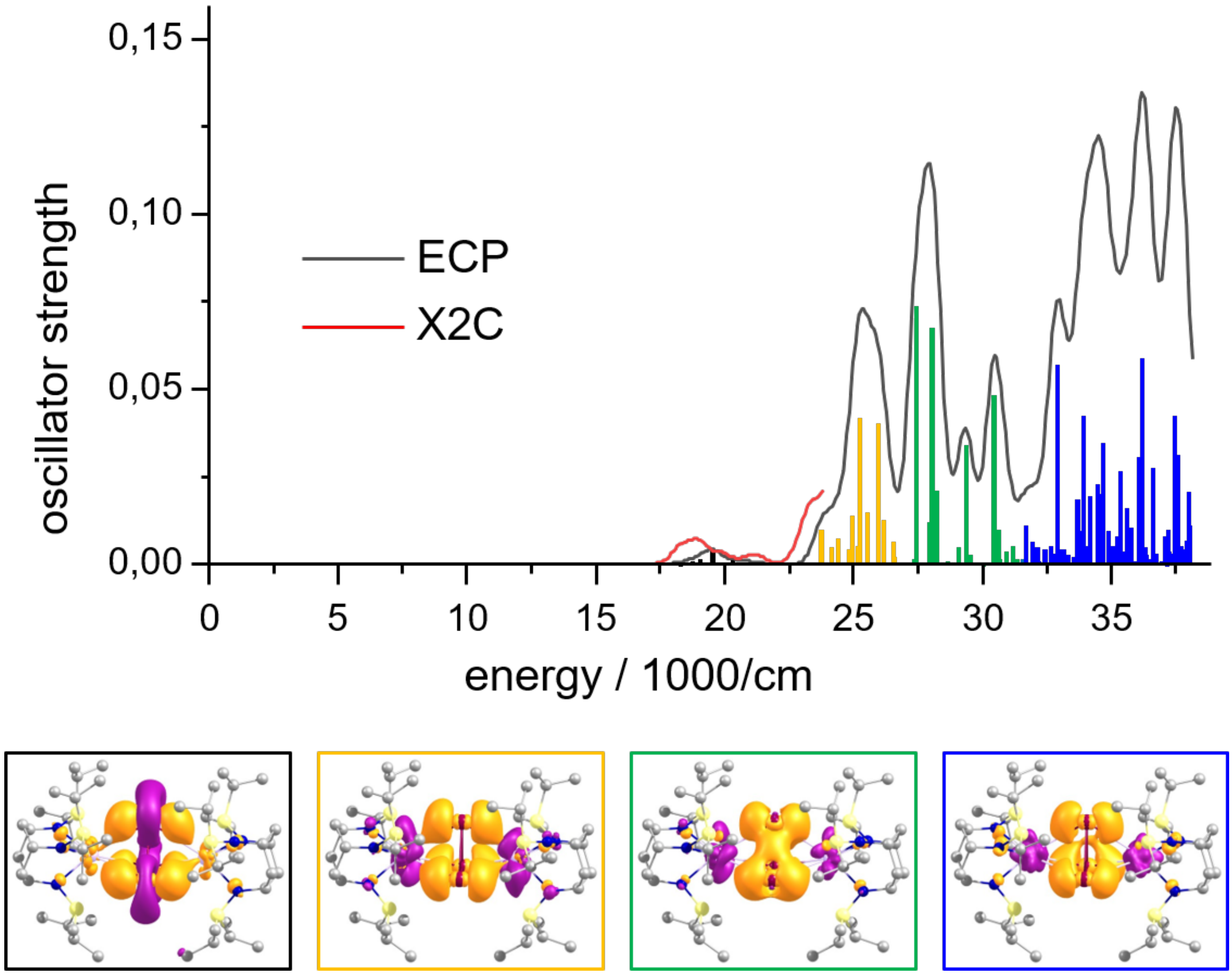

Supplementary Figure 133. Lowest 100 (with ECPs) and lowest 20 (X2C) spin-conserving excitations for **16'**.

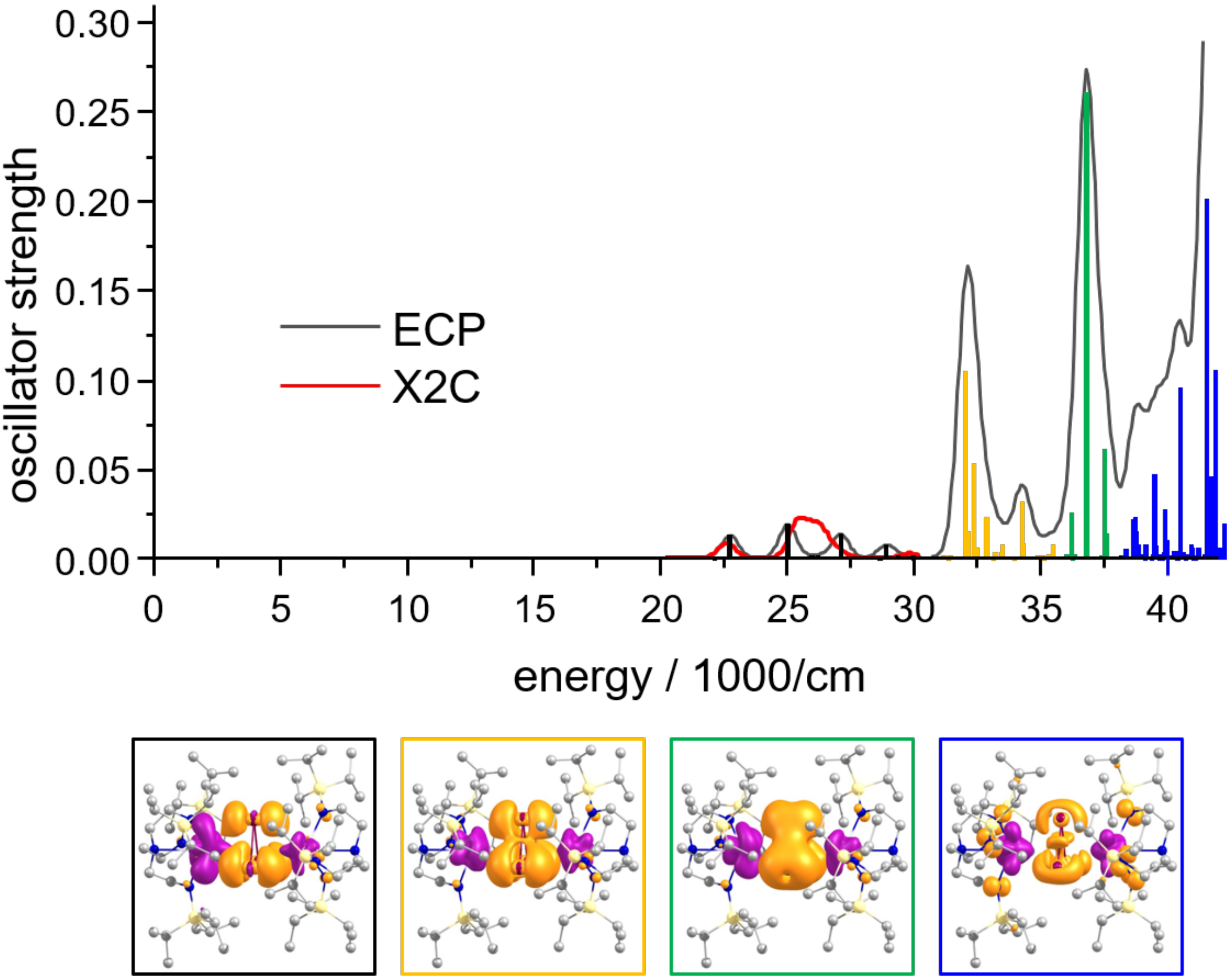

Supplementary Figure 134. Lowest spin-conserving excitations for **6a'(Th)**.

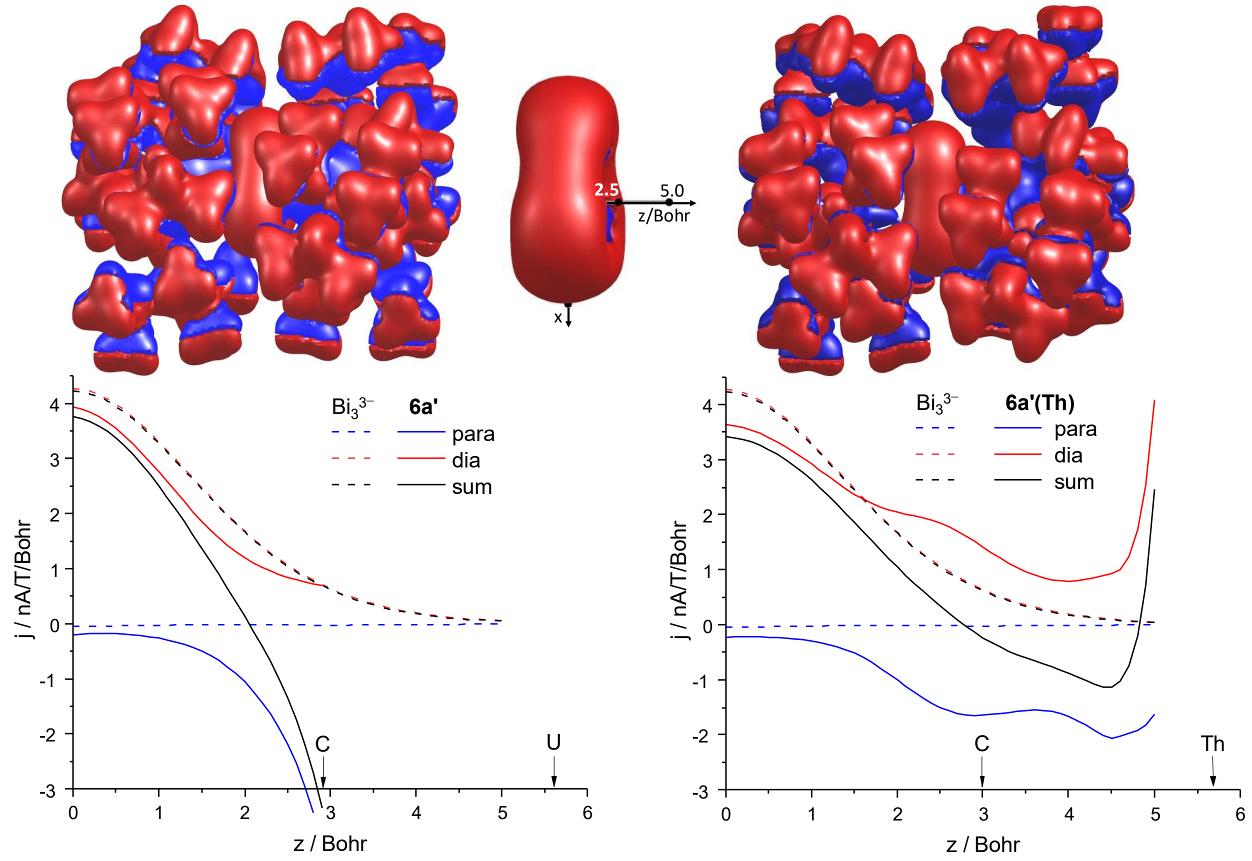

Supplementary Figure 135. Magnetic field-induced ring current densities and profiles for **6a'**, *cyclo*-Bi_3^{3-}, and **6a'(Th)** induced by a magnetic field in the z-direction. Top: ring current densities for **6a'**, *cyclo*-Bi_3^{3-}, and **6a'(Th)**. Regions with diatropic current density are depicted in red, those with paratropic current density are in blue. Contours are drawn at 0.02 a.u. Bottom: current profiles for **6a'** and **6a'(Th)**. The current profiles result from integration in the x- and y- directions for the *cyclo*-Bi_3^{3-} unit (dotted) and for the whole structures **6a'/6a'(Th)** (straight). Diatropic (red) and paratropic (blue) contributions are plotted separately as well as in total (black). “Th” and “U” mark the positions of those ions above the ring plane, “C” is the C atom closest to the ring plane.

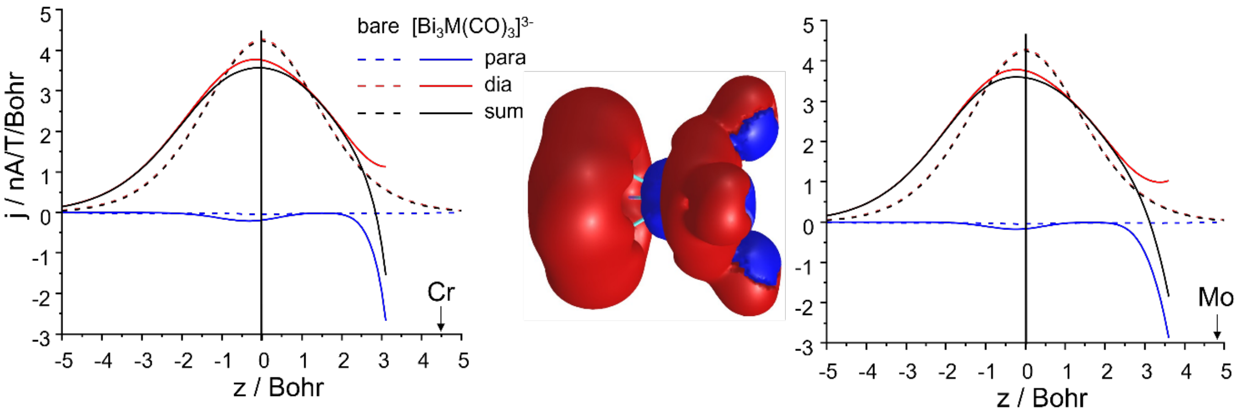

Supplementary Figure 136. Ring current profiles for $[(\eta^3\text{-}cyclo\text{-}Bi_3)M(CO)_3]^{3-}$ (M = Cr, left; Mo, right) and ring current density for M = Mo (middle). Current profiles result from the integration in x- and y- direction for Cr and Mo (straight lines) and for comparison for *cyclo*-Bi_3^{3-} (dotted). Diatropic and paratropic contributions are plotted separately (red/blue) as well as in total (black). "Cr" and "Mo" mark the position of the transition metal atoms above the ring plane. In the current density plot, middle, regions with diatropic current density are marked in red, those with paratropic current density in blue. Contours are drawn at 0.02 a.u.

Supplementary Tables

Supplementary Table 1. Comparison of CHN values between different single crystals of 6a:6b.

Entry	6a:6b-1	6a:6b-2	6a:6b-3	6a:6b-4
Solvent in lattice	Benzene	none	none	none
_cell_length_a	17.79750(10)	16.80211(15)	16.80211(15)	16.8952(2)
_cell_length_b	17.79750(10)	16.80211(15)	16.80211(15)	16.8952(2)
_cell_length_c	22.2147(2)	75.1435(8)	75.1435(8)	75.1366(11)
_cell_angle_alpha	90	90	90	90
_cell_angle_beta	90	90	90	90
_cell_angle_gamma	120	120	120	120
_cell_volume	6093.81(9)	18371.7(4)	18371.7(4)	18574.1(5)
_cell_formula_units_Z	2	6	6	6
UBiU	11%	22%	48%	26%
UBi_3U	89%	78%	52%	74%
Solvent used in reaction	Benzene	Toluene	Et_2O	DME
Molecular mass (g mol^{-1})	2851.25	2651.10	2542.43	2634.38
Expected C%	40.44	38.06	39.68	38.30
Expected H%	7.00	7.07	7.37	7.12
Expected N%	4.91	5.28	5.51	5.32
Found C%	40.28	38.26	40.22	38.77
Found H%	7.76	6.73	7.52	7.87
Found N%	4.65	4.95	5.66	4.87

Supplementary Table 2. Experimental X-ray crystallographic details for 6a-6b-1, 6a-6b-2, and 6a-6b-3.

	**6a6b-1**	**6a6b-2**	**6a6b-3**
Formula	$C_{102}H_{204}Bi_{2.77}KN_{10}O_6Si_6U_2$	$C_{84}H_{186}Bi_{2.56}KN_{10}O_6Si_6U_2$	$C_{84}H_{186}Bi_{2.03}KN_{10}O_6Si_6U_2$
Fw, g mol^{-1}	2929.365	2650.70	2540.30
Cryst size, mm	0.159 × 0.134 × 0.082	0.11 × 0.1 × 0.08	0.17 × 0.1 × 0.06
Crystal system	hexagonal	trigonal	trigonal
Space group	$P6_3$/m	R-3c	R-3c
Collection Temperature (K)	150(2)	100(2)	150(2)
a, (Å)	17.79750(10)	16.80211(15)	16.83021(17)
b, (Å)	17.79750(10)	16.80211(15)	16.83021(17)
c, (Å)	22.2147(2)	75.1435(8)	75.0665(9)
α, (°)	90	90	90
β, (°)	90	90	90
γ, (°)	120	120	120
V, (Å^3)	6093.81(9)	18371.7(4)	18414.3(4)
Z	2.00004	6.00012	6.00012
ρ_{calc} g cm-^{3}	1.596	1.438	1.374
μ, mm^{-1}	16.486	6.435	5.666
No. of reflections measured	56587	106966	122697
No. of unique reflections, R_{int}	3975, 0.0490	4874, 0.0611	6840, 0.0791
No. of reflections with $F^2 > 2\sigma(F^2)$	3817	4707	5451
Transmission coefficient range	0.221-0.715	0.635-0.794	0.611-1.000
R, R_w^a ($F^2 > 2\sigma(F^2)$)	0.0474, 0.1143	0.0897, 0.1994	0.0800, 0.1626
R, R_w^a (all data)	0.0489, 0.1154	0.0921, 0.2004	0.1075, 0.1706
S^a	0.9612	1.285	1.249
Parameters, Restraints	311, 735	220, 340	230, 303
Max.,min. difference map, e Å^{-3}	2.170, -1.211	4.178, -4.020	1.667, -3.934

^{a} Conventional $R = \Sigma||F_o| - |F_c||/\Sigma|F_o|$; $R_w = [\Sigma w(F_o^2 - F_c^2)^2/\Sigma w(F_o^2)^2]^{1/2}$;
$S = [\Sigma w(F_o^2 - F_c^2)^2/\text{no. data} - \text{no. params})]^{1/2}$ for all data.

Supplementary Table 3. Experimental X-ray crystallographic details for 6a-6b-4, 8, and 9.

	**6a6b-4**	**8**	**9**
Formula	$C_{84}H_{186}Bi_{2.47}KN_{10}O_6Si_6U_2$	$C_{66}H_{150}Bi_2N_8Si_6U_2$	$C_{33}H_{76}N_4OSi_3U$
Fw, g mol^{-1}	2632.24	2118.49	867.27
Cryst size, mm	0.15 × 0.11 × 0.09	0.08 × 0.07 × 0.06	0.13 × 0.11 × 0.06
Crystal system	trigonal	triclinic	monoclinic
Space group	*R*-3c	*P*-1	$P2_1$
Collection Temperature (K)	150(2)	100(2)	100(2)
a, (Å)	16.8952(2)	12.8845(3)	8.37440(10)
b, (Å)	16.8952(2)	13.2395(4)	20.5088(2)
c, (Å)	75.1366(11)	15.7529(7)	12.29690(10)
α, (°)	90	65.555(4)	90
β, (°)	90	89.384(3)	109.3970(10)
γ, (°)	120	61.012(3)	90
V, (Å^3)	18574.1(5)	2078.17(15)	1992.10(4)
Z	6.00012	1	2
ρ_{calc} g cm^{-3}	1.412	1.693	1.446
μ, mm^{-1}	6.240	20.106	12.542
No. of reflections measured	30724	7573	27716
No. of unique reflections, R_{int}	4577, 0.0516	7573, 0.0865	8381, 0.0319
No. of reflections with $F^2 > 2\sigma(F^2)$	4221	7184	8185
Transmission coefficient range	0.523-1.000	0.396-0.568	0.297-0.666
R, $R_w{}^a$ ($F^2 > 2\sigma(F^2)$)	0.0981, 0.2344	0.0947, 0.2819	0.0281, 0.0757
R, $R_w{}^a$ (all data)	0.1034, 0.2363	0.0972, 0.2829	0.0285, 0.0761
S^a	1.331	1.144	1.099
Parameters, Restraints	219, 344	419, 596	724, 1935
Max.,min. difference map, e Å^{-3}	5.853, -6.026	8.380, -3.875	1.292, -1.957

^{a} Conventional $R = \Sigma||F_o| - |F_c||/\Sigma|F_o|$; $R_w = [\Sigma w(F_o^2 - F_c^2)^2/\Sigma w(F_o^2)^2]^{1/2}$;
$S = [\Sigma w(F_o^2 - F_c^2)^2/\text{no. data} - \text{no. params})]^{1/2}$ for all data.

Supplementary Table 4. Experimental X-ray crystallographic details for 10, 12, and 14.

	**10**	**12**	**14**
Formula	$C_{80}H_{178}N_8O_2Si_6U_2$	$C_{53.40}H_{116.60}ClKN_6O_6Si_3U$	$C_{48}H_{102}Cl_3LiN_6P_6Th_2$
Fw, g mol^{-1}	1928.89	1335.77	1526.54
Cryst size, mm	0.198 × 0.183 × 0.096	0.378 × 0.241 × 0.148	0.226 × 0.146 × 0.141
Crystal system	triclinic	triclinic	monoclinic
Space group	*P*-1	*P*-1	$P2_1/n$
Collection Temperature (K)	100(2)	100(2)	150(2)
a, (Å)	11.5167(2)	21.2805(3)	12.9203(3)
b, (Å)	14.5555(3)	22.1436(4)	25.8854(5)
c, (Å)	15.1606(2)	38.8599(6)	19.7391(6)
α, (°)	94.441(2)	83.7610(10)	90
β, (°)	107.7450(10)	86.9810(10)	93.422(2)
γ, (°)	96.421(2)	68.387(2)	90
V, (Å^3)	2388.54(7)	16922.0(5)	6589.9(3)
Z	1	10	4
ρ_{calc} g cm^{-3}	1.341	1.311	1.539
μ, mm^{-1}	10.514	2.598	17.204
No. of reflections measured	49135	312554	74698
No. of unique reflections, R_{int}	10033, 0.0614	87371, 0.1287	11663, 0.0698
No. of reflections with $F^2 > 2\sigma(F^2)$	9389	54857	9669
Transmission coefficient range	0.272-0.851	0.049-0.930	0.102-0.428
R, $R_w{}^a$ ($F^2 > 2\sigma(F^2)$)	0.0507, 0.1362	0.0948, 0.2248	0.0948, 0.2684
R, $R_w{}^a$ (all data)	0.0528, 0.1386	0.1471, 0.2503	0.1056, 0.2808
S^a	1.100	1.050	1.042
Parameters, Restraints	465, 0	3374, 884	605, 1080
Max.,min. difference map, e Å^{-3}	5.076, -5.010	6.866, -3.367	2.197, -1.549

^{a} Conventional $R = \Sigma||F_o| - |F_c||/\Sigma|F_o|$; $R_w = [\Sigma w(F_o^2 - F_c^2)^2/\Sigma w(F_o^2)^2]^{1/2}$;
$S = [\Sigma w(F_o^2 - F_c^2)^2/\text{no. data} - \text{no. params})]^{1/2}$ for all data.

Supplementary Table 5. Experimental X-ray crystallographic details for 15 and 16.

	**15**	**16**
Formula	$C_{64}H_{138}Cl_4MgN_6O_4P_6Th_2$	$C_{73}H_{151}Bi_3KN_8O_7P_6Th_2$
Fw, g mol^{-1}	1871.81	2568.95
Cryst size, mm	0.16 × 0.12 × 0.09	0.11 × 0.09 × 0.08
Crystal system	monoclinic	orthorhombic
Space group	$P2_1/n$	*P*ccn
Collection Temperature (K)	150(2)	150(2)
a, (Å)	12.1371(2)	25.1158(3)
b, (Å)	18.9093(3)	26.9587(4)
c, (Å)	18.3813(3)	28.5737(3)
α, (°)	90	90
β, (°)	92.547(2)	90
γ, (°)	90	90
V, (Å^3)	4214.42(12)	19346.9(4)
Z	2	8
ρ_{calc} g cm-^{3}	1.475	1.764
μ, mm^{-1}	3.816	21.988
No. of reflections measured	32133	68218
No. of unique reflections, R_{int}	10730, 0.0285	19160, 0.0520
No. of reflections with $F^2 > 2\sigma(F^2)$	9007	16358
Transmission coefficient range	0.69236-1.00000	0.46773-1.00000
R, R_w^a ($F^2 > 2\sigma(F^2)$)	0.0267, 0.0551	0.0480, 0.1213
R, R_w^a (all data)	0.0376, 0.0577	0.0571, 0.1279
S^a	1.045	1.030
Parameters, Restraints	553, 583	1060, 431
Max.,min. difference map, e Å^{-3}	1.174, -0.425	1.520, -3.111

^{a} Conventional $R = \Sigma||F_o| - |F_c||/\Sigma|F_o|$; $R_w = [\Sigma w(F_o^2 - F_c^2)^2/\Sigma w(F_o^2)^2]^{1/2}$;
$S = [\Sigma w(F_o^2 - F_c^2)^2/\text{no. data} - \text{no. params})]^{1/2}$ for all data.

Supplementary Table 6. Sample information for those used in SQUID magnetometry measurements.

**Entry**	**6a:6b**	**17**
Chemical formula	$C_{84}H_{186}N_{10}O_6KSi_6Bi_{2.8}U_2{\bullet}(C_6H_6)_2$	$C_{66}H_{138}Bi_3KN_8O_6P_6Th_2$
Molecular mass (g mol^{-1})	2899.27	2455.84
Sample mass (mg)	31.5	31.1
Eicosane mass (mg)	14.0	21.5

Supplementary Table 7. Selected data for the magnetic properties of 16.

**Entry**	**16**
χ_D (300 K, whole molecule)[a]	–1079.599E-06
R^2 value	0.9999
χ_D (300 K, 1/3 molecule)	–359.866E-06
χ_D (300 K, per Bi)	–46.120E-06
Bi Pascal's constant[b]	–25.000E-06
Factor change vs expected	1.84479
Expected χ_D (whole molecule)	–1016.240E-06
χ_D per 1/3 molecule	–338.747E-06
χ_D $C_{66}H_{138}KN_8O_6P_6Th_2$	–941.240E-06
χ_D per 1/3 of $C_{66}H_{138}KN_8O_6P_6Th_2$	–313.747E-06
χ_D Increase vs expected (%)	5.86874
Λ (χ_D(expected) – χ_D(Found)[c]	63.359E-06

[a] χ is given in units of cm^3 mol^{-1}.
[b] This value is quoted as being uncertain in Pascal's Tables, but by comparing data in Pascal's constants would introduce an error of <0.18%.
[c]When using χ_D = the molecular weight multiplied by -0.5×10^{-6} cm^3 mol^{-1} rather than the addition of Pascal's constants, the χ_D increase vs expected = 5.77727% and Λ = 75.290E-06 cm^3 mol^{-1}.

Supplementary Table 8. Bi-Bi distances (in pm) in Bi_3^{3-} for the triangular (D_{3h}) and the bent (C_{2v}) structure, as well as angle in the bent structure. D3-BJ and COSMO are used for all calculations. Settings concerning functional and relativity were varied as specified in the first two columns. 1c/2c denotes the usage of the one- or the two-component formalism. Employed bases: def2-TZVP for ECP, x2c-TZVPall-2c for X2C). The numerical integration in DFT was done with fine grids (gridsize 5 plus weight derivatives).

functional	relativity	dist(D_{3h})	dist(C_{2v})	angle(C_{2v})
PBE	1c-ECP	308.7	292.5	116.3
PBE	1c-X2C	308.1	291.7	117.6
PBE	2c-ECP	314.6	297.7	118.3
PBE	2c-X2C	314.5	297.3	120.9
PBE0	1c-ECP	304.5	287.5	115.1
PBE0	1c-X2C	303.9	286.7	116.1
PBE0	2c-ECP	309.6	292.8	116.9
PBE0	2c-X2C	309.2	292.3	118.3

Supplementary Table 9. Data for Bi_3^{3-} at different levels of theory (see also Supplementary Table 8) for the 1c-ECP/PBE structure parameters (entry "N" in column geo-opt) as well as for the structure parameters optimised at the corresponding level (entry "Y" in column geo-opt). ΔE denotes the energy of the bent relative to the triangular structure, E(S,T) are the lowest excitation energies, for the one-component calculations separately listed for singlet and triplet, J is the induced magnetic current, and NICS0 to NICS3 are the nucleus-independent chemical shifts in ppm 0, 1, 2, 3 Bohr above the ring centre.

functional	relativity	Geo-opt	ΔE/kJ/mol	E(S,T)/eV	J/nA/T	NICS / ppm			
						0	1	2	3
PBE	1c-ECP	Y	10	1.55,1.17	16.2	–40.9	–32.1	–17.5	–8.7
PBE	1c-X2C	N	8	1.69,1.27	16.2	–41.5	–32.8	–18.0	–8.9
PBE	1c-X2C	Y	8	1.70,1.27	16.2	–41.6	–32.8	–18.0	–8.9
PBE	2c-ECP	N	–14	0.95	-	-	-	-	-
PBE	2c-ECP	Y	–14	0.89	-	-	-	-	-
PBE	2c-X2C	N	–17	1.00	-	–38.5	–29.1	–14.3	–6.4
PBE	2c-X2C	Y	–17	0.94	-	–37.2	–28.5	–14.4	–6.4
PBE0	1c-ECP	N	29	1.68,1.26	16.8	–42.3	–33.3	–18.2	–8.9
PBE0	1c-ECP	Y	28	1.71,1.30	17.0	–42.7	–33.4	–18.0	–8.8
PBE0	1c-X2C	N	29	1.81,1.37	16.8	–42.9	–33.9	–18.7	–9.2
PBE0	1c-X2C	Y	28	1.86,1.41	17.0	–43.4	–34.1	–18.6	–9.2
PBE0	2c-ECP	N	-2	1.02	-	-	-	-	-
PBE0	2c-ECP	Y	-2	1.01	-	-	-	-	-
PBE0	2c-X2C	N	-5	1.07	-	–38.8	–29.4	–14.5	–6.5
PBE0	2c-X2C	Y	-5	1.06	-	–38.6	–29.3	–14.5	–6.5

Supplementary Table 10. Energies of the bent structure relative to the triangular structure in kJ mol^{-1} for the PBE/ECP structure parameters at levels PBE0/ECP, PBE/1c-X2C, PBE/2c-X2C, PBE0/2c-X2C. D3-BJ and COSMO are used throughout, DFT gridsize 5 plus weight derivatives; bases: def2-TZVP). See also Supplementary Table 8. In case of $(CH)_3^{3-}$ and $(CH)_3^{+}$ the first two columns contain the non-relativistic data.

	ECP		X2C			
	PBE	PBE0/ECP	PBE/1c-X2C	PBE/2c-x2c	PBE0/1c-X2C	PBE0/2c-X2C
Bi_3^{3-}	10	29	8	-17	29	-5
Po_3	33	64	35	4	66	21
Sb_3^{3-}	3	20	2	-3	22	15
Te_3	25	54	26	18	55	43
$(CH)_3^{3-}$	–537	–547	–537	–537	–547	–547
$(C_3H_2)^{2-}$+H^-	–618	–620	–619	–618	–621	–620
$(CH)_3^{+}$	412	418	412	412	418	418

Supplementary Table 11. Magnetically induced ring currents (total current and diatropic contribution) for rings with 6 π electrons 6a', 6a'(Th), 16', Bi_3^{3-}, Po_3, Sb_3^{3-}, Te_3, $(C_3H_3)^{3-}$, C_6H_6) and with 2 π-electrons (Bi_3^{+}, Sb_3^{+}, $(C_3H_3)^{+}$). In the first four columns, the integration boundaries for the ring currents in the z direction were set to ±10 Bohr for the bare rings, and to ±2 Bohr for the rings connected to the actinide ligands (larger values would lead to unwanted inclusion of atomic current contributions from the actinide atoms). In column, 'total, 2 Bohr', the integration boundaries are set to ±2 Bohr also for the bare rings. Structure parameters were obtained with PBE/ECP, for further settings see Supplementary Table 8. Nucleus-independent chemical shifts (NICS) are calculated at the ring centre and as well as 1, 2 and 3 Bohr above the plane, see also Supplementary Table 9.

	J / nA/T					NICS / ppm							
	PBE/ECP		PBE/1c-X2C			PBE/1c-X2C				PBE/2c-X2C			
	diat.	total	diat.	total	total, 2 Bohr	0	1	2	3	0	1	2	3
**6a'**	12.2	9.8	12.4	9.9	9.9	−41	−36	−22	+33	-	-	-	-
**6a'(Th)**	12.0	10.3	12.2	10.5	10.5	−38	−32	−19	+1	−32	−27	−15	-
**16'**	8.4	7.2	8.4	7.1	7.1	−29	−23	−6	+17	−22	−14	0	15
Bi_3^{3-}	16.4	16.2	16.4	16.2	12.9	−42	−33	−18	−9	−39	−29	−14	−6
Po_3	13.5	12.9	13.7	13.0	11.1	−40	−30	−14	−6	−34	−23	−9	−4
Sb_3^{3-}	16.2	15.9	15.7	15.3	12.4	−41	−31	−16	−8	−39	−29	−14	−6
Te_3	13.5	12.8	13.7	13.0	11.2	−40	−28	−12	−5	−37	−25	−10	−4
$(C_3H_3)^{3-}$	11.5	9.5	11.5	9.6	8.1	−37	−5	+7	+1	−37	−5	+7	+1
C_6H_6	16.7	11.8	16.7	11.8	9.8	−7	−9	−9	−7	−7	−9	−9	−7
Bi_3^{+}	17.6	17.3	17.9	17.5	13.5	−14	−16	−16	−11	+8	+5	0	−2
Sb_3^{+}	17.3	16.9	18.1	17.7	13.9	−15	−17	−17	−12	−8	−11	−13	−10
$(C_3H_3)^{+}$	12.0	10.5	11.8	10.4	9.6	−22	−28	−13	−5	−22	−27	−13	−5

Supplementary Table 12. Excitation energies E and (dimensionless) oscillator (Osc) strengths in the velocity representation for the lowest 50 spin-conserving excitations of irreps Γ= a, e for 6a' and 6a'(Th) in C_3 symmetry and the lowest 100 excitations for 16' in C_1 symmetry, obtained at (one-component) level PBE0/ECP for structure parameters of PBE/ECP with TDDFT.

**6a'**			**6a'(Th)**			**16'**	
Γ	E/cm^{-1}	Osc	Γ	E/cm^{-1}	Osc	E/cm^{-1}	Osc
a	1908.5	0.000116	e	21232.8	0.000081	18290.6	0.000173
e	2159.8	0.000035	a	21673.9	0.000010	18734.2	0.000593
a	2199.0	0.000070	e	22148.7	0.000101	19031.0	0.000991
e	2363.9	0.000979	a	22737.4	0.013090	19538.4	0.003608
a	3543.1	0.002024	e	25002.4	0.009402	20322.3	0.001061
a	3958.7	0.001432	e	25956.1	0.000504	21049.7	0.000557
e	4169.1	0.000086	a	26221.5	0.000972	23759.0	0.009590
e	4274.0	0.000288	a	26784.8	0.000002	24156.2	0.003966
a	5146.0	0.000040	e	27084.5	0.006944	24373.8	0.006597
a	5618.2	0.000013	a	28654.2	0.000133	24816.4	0.003484
e	6681.5	0.000556	e	28912.8	0.003558	24910.9	0.013003
e	7054.9	0.000462	a	29407.4	0.000809	25165.7	0.004460
a	8015.3	0.005236	a	29855.1	0.000000	25247.4	0.041278
e	8034.2	0.000071	e	29961.4	0.000087	25507.5	0.014186
e	8328.1	0.000005	a	29968.8	0.000814	25938.9	0.039700
a	8431.9	0.000011	e	31186.5	0.000452	25961.1	0.000539
e	8834.1	0.000231	e	31362.7	0.000258	26163.4	0.012077
e	8911.7	0.000303	e	31412.7	0.000416	26564.7	0.005722
e	9233.6	0.002791	a	31463.8	0.000821	26595.0	0.001286
a	9299.3	0.000011	a	32002.4	0.104239	27357.4	0.000759

a	11219.1	0.190039	a	32147.9	0.003118	27441.1	0.073332
a	14802.0	0.001548	e	32164.0	0.007174	27946.0	0.011509
a	16794.7	0.002400	e	32350.5	0.026130	28069.8	0.067048
e	17088.5	0.000007	a	32481.3	0.005689	28216.0	0.020750
a	17368.3	0.000008	e	32857.0	0.011456	28638.1	0.000523
e	18698.4	0.000158	e	33139.2	0.000358	29078.3	0.004409
e	20045.0	0.000865	e	33241.8	0.001347	29342.7	0.033315
e	20085.2	0.000103	e	33469.8	0.001377	29515.1	0.001891
a	20219.5	0.000320	a	33484.8	0.007502	30027.5	0.000118
a	20311.1	0.000142	e	33971.5	0.000051	30456.8	0.047636
e	21135.7	0.000481	a	34272.5	0.031240	30605.2	0.009609
a	21318.6	0.009889	e	34299.3	0.004335	30680.1	0.000916
e	21948.6	0.000054	e	34900.7	0.000594	30939.3	0.002946
a	22879.5	0.000314	e	34977.8	0.000459	31165.5	0.004616
e	23209.0	0.000636	a	35066.4	0.000956	31333.2	0.000950
e	23644.7	0.000166	e	35087.5	0.000575	31559.1	0.001177
a	24081.4	0.025808	a	35150.2	0.000744	31686.8	0.010672
e	24112.4	0.007349	a	35298.2	0.001793	31943.8	0.005993
a	24280.2	0.000071	e	35470.7	0.000446	32142.9	0.004318
a	24428.2	0.009914	a	35512.5	0.007730	32163.8	0.000700
e	24432.0	0.004381	a	36038.7	0.001636	32436.4	0.003879
a	24632.4	0.005639	a	36225.2	0.025279	32681.8	0.004624
a	24776.3	0.000104	e	36259.2	0.000137	32789.2	0.002758
e	24934.4	0.002391	a	36328.4	0.001810	32915.8	0.056301
e	25063.0	0.001598	a	36815.1	0.259581	33025.5	0.003787
a	25181.3	0.001388	e	37384.4	0.000641	33134.4	0.003449
e	25278.3	0.004568	e	37541.3	0.030179	33295.3	0.001986
e	25365.2	0.002857	e	37605.4	0.007017	33339.5	0.001116
a	25515.6	0.000312	a	38206.5	0.000842	33589.9	0.001567
a	25717.4	0.000074	a	38234.7	0.001218	33613.0	0.000363
e	25846.2	0.005713	e	38362.9	0.002364	33697.0	0.018164
e	26119.3	0.000162	a	38510.3	0.000048	33762.9	0.009047
a	26134.7	0.001009	e	38664.5	0.010593	33939.8	0.041875
e	26195.5	0.003900	a	38714.2	0.022272	34024.2	0.004719
a	26339.1	0.000002	a	38779.9	0.014235	34132.8	0.019265
e	26686.3	0.003368	e	38805.1	0.003142	34478.0	0.022090
a	27109.4	0.002134	a	38838.1	0.007228	34534.5	0.014655
e	27117.2	0.000134	a	38995.7	0.002044	34599.6	0.019797
e	27537.6	0.001857	a	39005.2	0.001278	34684.9	0.034138
a	27723.9	0.013973	a	39134.2	0.000312	34851.8	0.008782
e	27795.1	0.000740	e	39152.5	0.003750	34901.4	0.001701
a	27899.9	0.002748	a	39362.7	0.000436	34977.9	0.004834
a	28490.4	0.005636	a	39438.4	0.001755	35074.2	0.004607
e	28586.0	0.002964	e	39503.7	0.023043	35216.1	0.007393
a	28886.7	0.000906	a	39542.8	0.006473	35366.4	0.026042
a	28977.9	0.002468	e	39585.4	0.001272	35426.4	0.003032
e	28996.7	0.000359	e	39706.5	0.000938	35572.0	0.015520
e	29203.3	0.003421	a	39709.8	0.000605	35656.4	0.007925
a	29317.2	0.000120	a	39827.6	0.000279	35758.2	0.009893
a	29369.3	0.000653	e	39849.0	0.001582	35974.9	0.000467
e	29493.0	0.000167	e	39908.8	0.013369	36086.2	0.030291
a	29580.4	0.001081	e	40001.6	0.005251	36156.0	0.001817
a	29637.3	0.008242	e	40065.8	0.000771	36169.1	0.008219
e	29679.8	0.000035	a	40214.3	0.001606	36222.6	0.058184
a	29755.4	0.000139	a	40235.4	0.000010	36318.0	0.004345
e	29805.1	0.000156	e	40292.7	0.002045	36356.5	0.002301
a	29908.6	0.000574	a	40311.1	0.000068	36428.8	0.001188

e	30035.6	0.001579	e	40352.0	0.001802	36466.0	0.000706
e	30279.5	0.000173	e	40416.3	0.000287	36602.3	0.026885
e	30479.2	0.001661	a	40509.6	0.095424	36617.3	0.007107
e	30652.2	0.002129	e	40538.3	0.001712	36711.1	0.002449
e	30726.7	0.004057	e	40658.7	0.001319	36996.5	0.000435
a	30896.5	0.000529	a	40756.7	0.000682	37080.6	0.007339
e	31085.5	0.000067	e	40766.8	0.001175	37154.1	0.000105
e	31367.2	0.000045	a	40844.1	0.001130	37199.1	0.009253
e	31586.1	0.000289	e	40940.2	0.003821	37217.9	0.004710
a	31675.6	0.008557	a	40990.8	0.005455	37239.3	0.003174
e	31928.9	0.000621	e	41067.0	0.000073	37302.5	0.002434
a	31953.5	0.005587	a	41118.8	0.001383	37339.8	0.001627
e	32016.1	0.002024	e	41139.4	0.000187	37351.0	0.001544
a	32079.8	0.000308	e	41268.0	0.002773	37429.5	0.041758
a	32140.0	0.000748	e	41474.1	0.000817	37512.9	0.015142
e	32144.2	0.000417	a	41541.6	0.200252	37584.7	0.030969
e	32420.7	0.000952	e	41556.9	0.003471	37684.9	0.004925
a	32444.7	0.000016	e	41619.9	0.008747	37750.0	0.003545
a	33178.4	0.000020	a	41708.3	0.045196	37853.2	0.001432
a	33257.7	0.001463	a	41856.9	0.000010	37909.4	0.006208
a	33450.6	0.000903	a	41899.0	0.105220	37931.2	0.002267
a	33676.9	0.008093	a	42069.6	0.005687	37998.2	0.020340
a	33706.3	0.000694	a	42239.3	0.018820	38034.6	0.010509

Supplementary Table 13. Excitation energies E and (dimensionless) oscillator (Osc) strengths in the velocity and the length representation for the lowest 10 spin-conserving excitations of irreps Γ= a, e for 6a' and 6a'(Th) in C_3 symmetry and the lowest 100 spin-conserving excitations for 16' in C_1 symmetry, obtained at (one-component) level PBE0/X2C for structure parameters of PBE/ECP with TDDFT. Note that the excitations transforming according to irrep e are twofold degenerate.

	**6a'**			**6a'(Th)**		**16'**	
Γ	E/cm^{-1}	Osc	Γ	E/cm^{-1}	Osc	E/cm^{-1}	Osc
a	1944.0	0.000008	e	21260.5	0.000087	17773.0	0.000334
e	2137.3	0.000000	a	21778.7	0.000008	18334.2	0.005046
a	2253.7	0.000014	e	22137.4	0.000082	18675.3	0.000066
e	2354.7	0.000069	e	22473.5	0.000537	19009.0	0.006010
a	3455.1	0.000172	a	22647.7	0.006899	19818.5	0.001847
a	3895.0	0.000213	e	22693.0	0.000290	19866.6	0.000739
e	4076.7	0.000002	e	25405.5	0.009444	19900.7	0.000513
e	4175.6	0.000059	a	25963.0	0.001127	20650.7	0.001022
a	5048.3	0.000003	e	25983.7	0.000442	21200.7	0.002123
a	5572.1	0.000000	a	25991.3	0.007867	21272.9	0.000364
e	6483.5	0.000018	e	26089.0	0.001278	22703.4	0.001034
e	6850.0	0.000012	a	26367.1	0.003152	23033.1	0.000961
a	7689.2	0.005024	a	26477.5	0.003354	23137.4	0.006777
e	7722.3	0.000014	e	26497.6	0.002250	23315.2	0.000206
e	8058.8	0.000108	e	27016.8	0.000059	23398.3	0.005914
a	8127.9	0.000089	a	27104.2	0.002250	23631.8	0.005862
e	8640.3	0.000016	e	27202.6	0.000200	23806.7	0.000763
e	8716.4	0.000025	a	27512.5	0.000179	24146.8	0.004968
a	9569.6	0.000055	a	28697.0	0.000094	24225.9	0.016340
a	11061.2	0.154710	a	29859.2	0.002651	24346.8	0.000640

Supplementary References

1. King, D. M. et al. Synthesis and Structure of a Terminal Uranium Nitride Complex. *Science* **337**, 717-720 (2012).
2. Gardner, B. M. et al. Triamidoamine–Uranium(IV)-Stabilized Terminal Parent Phosphide and Phosphinidene Complexes. *Angew. Chem. Int. Ed*. **53**, 4484-4488 (2014).
3. Popovych, O. Conductometric Determination of Solubility and Solubility Products of Silver Salts in a Medium of Low Dielectric Constant. *Anal. Chem*. **38**, 117A (1966).
4. Straus, D. A., Zhang, C & Tilley, T. D. Trityl tetraphenylborate as a reagent in organometallic chemistry. *J. Organomet. Chem*., **369**, C13-Cl7 (1989).
5. Barker, B. J. & Sears, P. G. Conductance behavior of some ammonium and partially substituted ammonium tetraphenylborates in 3-methyl-2-oxazolidone and 3-*tert*-butyl-2-oxazolidone at 25°. *J. Phys. Chem.* **78**, 2687-2688 (1974).
6. Maria, L. et al. A novel samarium(II) complex bearing a dianionic bis(phenolate) cyclam ligand: synthesis, structure and electron-transfer reactions. *Dalton Trans*., **45**, 3778-3790 (2016).
7. Bergbreiter, D. E. & Killough, J. M. Reactions of potassium-graphite. *J. Am. Chem. Soc*. **100**, 2126-2134 (1978).
8. Ababei, R. et al. Making practical use of the pseudo-element concept: an efficient way to ternary intermetalloid clusters by an isoelectronic Pb^{-}-Bi combination. *Chem. Commun*. **48**, 11295-11297 (2012).
9. Xu, L. & Sevov, S. C. Heteroatomic Deltahedral Clusters of Main-Group Elements: Synthesis and Structure of the Zintl Ions $[In_4Bi_5]^{3-}$, $[InBi_3]^{2-}$, and $[GaBi_3]^{2-}$. *Inorg. Chem.* **39**, 5383-5389 (2000).
10. Lichtenberger, N. et al. Main Group Metal-Actinide Magnetic Coupling and Structural Response Upon U^{4+} Inclusion Into Bi, Tl/Bi, or Pb/Bi Cages. *J. Am. Chem. Soc.* **138**, 9033-9036 (2016).

11. Xu, L., Bobev, S., El-Bahraoui & Sevov, S. C. A Naked Diatomic Molecule of Bismuth, $[Bi_2]^{2-}$, with a Short Bi-Bi Bond: Synthesis and Structure. *J. Am. Chem. Soc.* **122**, 1838-1839 (2000).

12. Turculet, L. & Tilley , T. D. Synthesis and Reactivity of d^0 Alkyl, Silyl, and Hydride Complexes of Titanium and Zirconium Featuring an Aryl-Substituted Tripodal Triamido Ligand Derived from cis,cis-1,3,5-Triaminocyclohexane. *Organometallics* **2004**, *23*, 1542-1553.

13. Gascoin, F. & Sevov, S. C. Synthesis and Characterization of the “Metallic Salts” A_5Pn_4 (A = K, Rb, Cs and Pn = As, Sb, Bi) with Isolated Zigzag Tetramers of Pn_4^{4-} and an Extra Delocalized Electron. *Inorg. Chem.* **40**, 5177-5181 (2001).

14. Lochmann, L. & Trekoval, J. Lithium-potassium exchange in alkyllithium/potassium t-pentoxide systems: XIV. Interactions of alkoxides. *J. Organomet. Chem.* **326**, 1-7 (1987).

15. Patel, D. et al. Comments on reactions of oxide derivatives of uranium with hexachloropropene to give UCl_4. *New J. Chem.* **39**, 7559-7562 (2015).

16. Gardner, B. M., et al. The Role of 5f-Orbital Participation in Unexpected Inversion of the σ-Bond Metathesis Reactivity Trend of Triamidoamine Thorium(IV) and Uranium(IV) Alkyls. *Chem. Sci.* **5**, 2489-2497 (2014).

17. Lichtenberger, N., Franzke, Y. J., Massa, W., Weigend, F. & Dehnen, S. The Identity of “Ternary” A/Tl/Pb or K/Tl/Bi Solid Mixtures and Binary Zintl Anions Isolated From Their Solutions. *Chem. Eur. J.* **24***,* 12022-12030 (2018).

18. Sheldrick, G. M. SHELXT – Integrated space-group and crystal-structure determination. *Acta Cryst. Sect. A* **71**, 3-8 (2015).

19. Meurer, F. et al. Probing the Isolobal Relation Between Cp″NiP_3 and White Phosphorus by Experimental Charge Density Analysis. *Chem. Eur. J.* **30**, e202303762 (2024).

20. CrysAlisPRO version 39.46, Oxford Diffraction /Agilent Technologies UK Ltd, Yarnton, England.

21. Sheldrick, G. M. Crystal structure refinement with SHELXL. *Acta Cryst. Sect. C* **71**, 3-8 (2015).

22. Dolomanov, O. V., Bourhis, L. J., Gildea, R. J., Howard, J. A. K., Puschmann, H. OLEX2: a complete structure solution, refinement and analysis program. *J. Appl. Cryst.* **42**, 339-341 (2009).

23. Farrugia, L. J. WinGX and ORTEP for Windows: an update. *J. Appl. Cryst.* **45**, 849-854 (2012).

24. Persistence of Vision (TM) Raytracer, Persistence of Vision Pty. Ltd., Williamstown, Victoria, Australia.

25. Weinert, B., Müller, F. Harms, K., Clérac, R. & Dehnen, S. Origin and Location of Electrons and Protons during the Formation of Intermetalloid Clusters [Sm@Ga_3−xH_3−2×Bi_{10}+x]$^{3−}$ (x = 0, 1). *Angew. Chem. Int. Ed.* **53**, 11979-11983 (2014).

26. Pan, F. et al. Insights into Formation and Relationship of Multimetallic Clusters: On the Way toward Bi-Rich Nanostructures. *J. Am. Chem. Soc.* **143**, 7176-7188 (2021).

27. Schneider. H, Krahfuß, M. J. & Radius, U. To Rearrange or not to Rearrange: Reactivity of NHCs towards Chloro- and Hydrostannanes R_2SnCl_2 (R = Me, Ph) and Ph_3SnH. *Z. Anorg. Allg. Chem.* **642**, (22), 1282-1286 (2016).

28. TURBOMOLE Version 7.9, TURBOMOLE GmbH **2024**. TURBOMOLE is a development of University of Karlsruhe and Forschungszentrum Karlsruhe 1989-2007, TURBOMOLE GmbH since 2007; available from https://www.turbomole.org.

29. Perdew, J., Burke, K. & Ernzerhof, M. Generalized Gradient Approximation Made Simple. *Phys. Rev. Lett.* **77**, 3865-3868 (1996).

30. Weigend, F. & Ahlrichs, R. Balanced basis sets of split valence, triple zeta valence and quadruple zeta valence quality for H to Rn: Design and assessment of accuracy. *Phys. Chem. Chem. Phys.* **7**, 3297-3305 (2005).

31. Metz, B., Stoll, H. & Dolg, M. Small-core multiconfiguration-Dirac–Hartree–Fock-adjusted pseudopotentials for post-d main group elements: Application to PbH and PbO. *J. Chem. Phys.* **113**, 2563-2569 (2000).

32. Küchle, W., Dolg, M., Stoll, H. & Preuss, H. Energy-adjusted pseudopotentials for the actinides. Parameter sets and test calculations for thorium and thorium monoxide. *J. Chem. Phys.* **100**, 7535-7542 (1994).

33. Cao, X., Dolg, M. & Stoll, H. Valence basis sets for relativistic energy-consistent small-core actinide pseudopotentials. *J. Chem. Phys.* **118**, 487-496 (2003).

34. Pausch, A. Consistent Analytical Second Derivatives of the Kohn–Sham DFT Energy in the Framework of the Conductor-Like Screening Model through Gaussian Charge Distributions, *J. Chem. Theory Comput.* **20**, 3169-3183 (2024).

35. Treutler, O. & Ahlrichs, R. Efficient molecular numerical integration schemes. *J. Chem. Phys.* **102**, 346-354 (1995).

36. Eichkorn, K., Treutler, O., Öhm, H., Häser, M. & Ahlrichs, R. Auxiliary basis sets to approximate Coulomb potentials. *Chem. Phys. Lett.* **240**, 283-290 (1995).

37. Weigend, F. Accurate Coulomb-fitting basis sets for H to Rn. *Phys. Chem. Chem. Phys*. **8**, 1057-1065 (2006).

38. Sierka, M., Hogekamp, A. & Ahlrichs, R. Fast evaluation of the Coulomb potential for electron densities using multipole accelerated resolution of identity approximation. *J. Chem. Phys.* **118**, 9136-9148 (2003).

39. Reiter, K., Mack, F. & Weigend, F. Calculation of Magnetic Shielding Constants with meta-GGA Functionals Employing the Multipole-Accelerated Resolution of the Identity: Implementation and Assessment of Accuracy and Efficiency. *J. Chem. Theory Comput.* **14**, 191-197 (2018).

40. Jusélius, J., Sundholm, D. & Gauss, J. Calculation of current densities using gauge-including atomic orbitals. *J. Chem. Phys*. **121**, 3952-3963, (2004), https://github.com/qmcurrents/gimic.

41. Perdew, J. P., Burke, K. & Ernzerhof, M. Rationale for mixing exact exchange with density functional approximations. *J. Chem. Phys* **105**, 9982-9985 (1996).

42. Kühn, M. & Weigend, F. Phosphorescence lifetimes of organic light-emitting diodes from two-component time-dependent density functional theory. *J. Chem. Phys*. **141**, 224302 (2014).

43. Pipek, J. & Mezey, P. G. A fast intrinsic localization procedure applicable for ab initio and semiempirical linear combination of atomic orbital wave functions. *J. Chem. Phys*. **90**, 4916-4926 (1989).
