# Supplementary material for: All-metal aromaticity of cyclo-Bi33− in diuranium and dithorium inverse-sandwich-type complexes
Source: Nat Chem. 2026 Apr 20;18(7):1308–17. doi: 10.1038/s41557-026-02123-8 (PMC13322972; doi:10.1038/s41557-026-02123-8)
Supplement: Supplementary file 2 — Optimized Cartesian coordinates for 6a′, 6a′(Th), 6b′ and for 16′. [file 41557_2026_2123_MOESM2_ESM.pdf]

Supplementary Data File 1

Optimised Cartesian coordinates for 6a', 6a'(Th), 6b' and for 16' in Ångstrom. (PBE+D3-BJ, gridsize 5 plus weight derivatives; bases: def2-TZVP for Bi, N, def-TZVPP for U/Th, def2-SV(P) else)

6a', Energy/eV = –173378.7667531, lowest vibration frequency/cm^{-1} = 13

N	1.5459980	1.4593175	3.8924152
N	-2.0368051	0.6092148	3.8924152
N	0.4908070	-2.0685323	3.8924152
N	-0.0000000	-0.0000000	5.8683297
Si	3.2034006	1.9640114	3.5522985
Si	-3.3025840	1.7922206	3.5522985
Si	0.0991834	-3.7562320	3.5522985
U	-0.0000000	-0.0000000	2.9712271
C	2.7582099	4.1174851	1.6080766
C	3.3448029	3.8202134	2.9934197
C	5.3992233	1.2486365	1.8123461
C	2.7769067	4.8262584	4.0106069
C	5.5354959	2.7851881	5.1762933
C	3.9544658	0.8616162	2.1693051
C	4.2853216	1.8860608	5.1826514
C	3.8389064	-0.6528668	2.4018195
C	-1.3540539	3.6510239	2.4018195
C	4.6808926	0.4584482	5.5959220
C	-2.7234144	2.9938597	2.1693051
C	-3.7809626	4.0515463	1.8123461
C	0.9938240	2.0964609	5.1017189
C	2.1867417	-4.4474224	1.6080766
C	0.9134599	1.0881968	6.2454624
C	2.7912090	-4.8180009	4.0106069
C	-2.7374741	3.8245478	5.5959220
C	-4.9449517	0.3299373	1.6080766
C	1.6360004	-4.8067910	2.9934197
C	1.3186764	-1.9089073	5.1017189
C	-4.9808033	0.9865776	2.9934197
C	-3.7760374	2.7681670	5.1826514
C	0.4856761	-1.3351779	6.2454624
C	-1.2310514	-3.8554760	2.1693051
C	-1.3991360	0.2469811	6.2454624
C	-5.1797916	3.4012860	5.1762933
C	-2.3125004	-0.1875537	5.1017189
C	-2.4848525	-2.9981571	2.4018195
C	-5.5681157	-0.0082575	4.0106069
C	-1.6182608	-5.3001828	1.8123461
C	-0.5092842	-4.6542278	5.1826514
C	-0.3557042	-6.1864741	5.1762933
C	-1.9434185	-4.2829960	5.5959220
H	2.9462475	5.1780703	1.3105375
H	3.1866812	3.4694815	0.8155901
H	5.5034991	2.3304204	1.5758729

H	4.4474368	3.9895870	2.9341410
H	3.0242456	5.8743380	3.7139194
H	5.7521545	0.6795603	0.9203191
H	1.6571374	3.9574913	1.5999800
H	6.2409190	2.5062291	4.3618296
H	5.2886081	3.8603246	5.0454691
H	3.3095519	1.0887601	1.2846544
H	6.1030498	1.0164978	2.6440544
H	1.6665319	4.7574007	4.0547185
H	6.0965255	2.6901013	6.1381442
H	3.1642674	4.6735823	5.0428527
H	-1.0284168	4.2064068	1.4897973
H	4.1570635	-1.2125683	1.4897973
H	4.4717419	-1.0014509	3.2466794
H	5.4658986	0.0490684	4.9214652
H	3.6114565	2.2844895	5.9790094
H	-3.4645938	4.6417318	0.9203191
H	-0.5481500	2.9099269	2.6073103
H	-1.3685891	4.3733675	3.2466794
H	2.7941456	-0.9802516	2.6073103
H	5.0991721	0.4370698	6.6313861
H	1.5797005	2.9783017	5.4524677
H	-2.5976699	2.3217759	1.2846544
H	3.8241751	-0.2491577	5.5632758
H	-3.9318378	4.7771473	2.6440544
H	2.5987193	-3.4138687	1.5999800
H	-0.0333302	2.4886720	4.9066018
H	-4.7699528	3.6009599	1.5758729
H	3.0112167	-5.1405603	1.3105375
H	3.2867639	-3.8219593	4.0547185
H	1.9239838	0.6630295	6.4050813
H	1.4113185	-4.4944876	0.8155901
H	-4.5979997	1.0250061	0.8155901
H	-2.7754438	4.7090729	4.9214652
H	2.1719183	-1.2154712	4.9066018
H	3.5752031	-5.5562425	3.7139194
H	0.5908445	1.5684288	7.2057881
H	-1.6963106	3.4364116	5.5632758
H	-4.2558567	-0.5436226	1.5999800
H	-0.7118820	-3.4105360	1.2846544
H	2.4653073	-5.0771271	5.0428527
H	-5.2909176	4.1516798	4.3618296
H	1.7894347	-2.8572116	5.4524677
H	-2.9280996	4.1974777	6.6313861
H	-5.9574642	-0.0375100	1.3105375
H	-5.6788021	1.8567998	2.9341410
H	-1.5361923	1.3347041	6.4050813
H	1.2313653	-5.8463868	2.9341410
H	1.0628769	-1.2959008	7.2057881

H	-2.2459956	-1.9296753	2.6073103
H	-3.7841542	1.9853684	5.9790094
H	-5.3779588	3.9346953	6.1381442
H	-3.1286467	-2.9938384	1.4897973
H	-2.1385881	-1.2732008	4.9066018
H	-5.9874432	2.6499067	5.0454691
H	-0.3877915	-1.9977336	6.4050813
H	-3.3691352	-0.1210901	5.4524677
H	-1.6537215	-0.2725280	7.2057881
H	-0.7335463	-5.9313802	1.5758729
H	-4.9532958	-0.9354414	4.0547185
H	0.1726977	-4.2698579	5.9790094
H	-2.2875607	-5.3212921	0.9203191
H	-6.5994487	-0.3180954	3.7139194
H	-5.6295747	0.4035448	5.0428527
H	0.6988351	-6.5102313	5.0454691
H	-3.1031528	-3.3719166	3.2466794
H	-2.1712120	-5.7936451	2.6440544
H	-2.1278644	-3.1872539	5.5632758
H	-0.9500014	-6.6579089	4.3618296
H	-2.6904549	-4.7581413	4.9214652
H	-0.7185667	-6.6247966	6.1381442
H	-2.1710725	-4.6345475	6.6313861
N	2.0076962	0.7082643	-3.8914079
N	-1.6172230	1.3845838	-3.8914079
N	-0.3904732	-2.0928481	-3.8914079
N	0.0000000	0.0000000	-5.8759565
Si	3.7335811	0.4979419	-3.5736695
Si	-2.2980209	2.9844051	-3.5736695
Si	-1.4355602	-3.4823470	-3.5736695
U	0.0000000	0.0000000	-2.9774268
C	4.0800318	2.5356018	-1.5441349
C	-4.2359115	2.2656103	-1.5441349
C	0.1558797	-4.8012121	-1.5441349
C	4.5660993	2.1300538	-2.9398460
C	-4.1277304	2.8893311	-2.9398460
C	-0.4383689	-5.0193849	-2.9398460
C	5.4509284	-0.9474441	-1.7334651
C	-1.9049535	5.1943646	-1.7334651
C	-3.5459749	-4.2469204	-1.7334651
C	4.4297716	3.3256092	-3.8983779
C	-5.0949478	2.1734901	-3.8983779
C	0.6651762	-5.4990993	-3.8983779
C	6.2009605	0.3816401	-5.1708373
C	-3.4309902	5.1793693	-5.1708373
C	-2.7699703	-5.5610093	-5.1708373
C	3.9961743	-0.8374867	-2.2190697
C	-1.2728024	3.8795318	-2.2190697
C	-2.7233719	-3.0420451	-2.2190697

C	4.6949802	0.0642634	-5.2197097
C	-2.4031438	4.0338404	-5.2197097
C	-2.2918364	-4.0981038	-5.2197097
C	3.4330127	-2.2312368	-2.5420488
C	0.2158014	4.0886946	-2.5420488
C	-3.6488141	-1.8574578	-2.5420488
C	4.4890934	-1.3779376	-5.7139418
C	-1.0512177	4.5766378	-5.7139418
C	-3.4378757	-3.1987001	-5.7139418
C	1.7475077	1.5196176	-5.0960890
C	-2.1897813	0.7535773	-5.0960890
C	0.4422736	-2.2731949	-5.0960890
C	1.2722016	0.6360854	-6.2476070
C	-1.1869669	0.7837162	-6.2476070
C	-0.0852347	-1.4198016	-6.2476070
H	4.6201310	3.4391113	-1.1716625
H	-5.2884233	2.2815952	-1.1716625
H	0.6682923	-5.7207065	-1.1716625
H	4.2162913	1.7304154	-0.7914130
H	-3.6067293	2.7862077	-0.7914130
H	-0.6095620	-4.5166230	-0.7914130
H	5.8854001	0.0372134	-1.4542272
H	-2.9749278	5.0782993	-1.4542272
H	-2.9104723	-5.1155127	-1.4542272
H	5.6516196	1.8778876	-2.8655844
H	-4.4521082	3.9555023	-2.8655844
H	-1.1995114	-5.8333899	-2.8655844
H	5.0305046	4.1957114	-3.5384321
H	-6.1488450	2.2586891	-3.5384321
H	1.1183403	-6.4544005	-3.5384321
H	5.5143908	-1.6029248	-0.8321480
H	-1.3690218	5.5770649	-0.8321480
H	-4.1453690	-3.9741401	-0.8321480
H	2.9933988	2.7754899	-1.5612413
H	-3.9003441	1.2046145	-1.5612413
H	0.9069453	-3.9801043	-1.5612413
H	6.7179944	-0.2008029	-4.3751954
H	-3.1850968	5.9183553	-4.3751954
H	-3.5328976	-5.7175524	-4.3751954
H	6.4051303	1.4566817	-4.9787271
H	-4.4640885	4.8186647	-4.9787271
H	-1.9410418	-6.2753464	-4.9787271
H	3.3848679	-0.4393813	-1.3700669
H	-1.3119186	3.1510723	-1.3700669
H	-2.0729494	-2.7116910	-1.3700669
H	6.1111278	-1.3969616	-2.5105082
H	-1.8457596	5.9908727	-2.5105082
H	-4.2653681	-4.5939111	-2.5105082
H	3.3716279	3.6668450	-3.9559988

H	-4.8613949	1.0864930	-3.9559988
H	1.4897669	-4.7533379	-3.9559988
H	6.6943070	0.1188293	-6.1384451
H	-3.4500627	5.7380253	-6.1384451
H	-3.2442443	-5.8568545	-6.1384451
H	4.7663388	3.0951615	-4.9343112
H	-5.0636579	2.5801898	-4.9343112
H	0.2973190	-5.6753512	-4.9343112
H	0.7626005	4.4546631	-1.6401540
H	-4.2391516	-1.5669001	-1.6401540
H	3.4765511	-2.8877629	-1.6401540
H	4.0055654	-2.7369194	-3.3493709
H	0.3674591	4.8373811	-3.3493709
H	-4.3730244	-2.1004617	-3.3493709
H	5.0677467	-2.0985136	-5.0942016
H	-0.7165073	5.4380542	-5.0942016
H	-4.3512394	-3.3395406	-5.0942016
H	4.2507378	0.7427546	-5.9873403
H	-2.7686132	3.3098697	-5.9873403
H	-1.4821246	-4.0526242	-5.9873403
H	0.7216360	3.1455135	-2.8460997
H	-3.0849126	-0.9478017	-2.8460997
H	2.3632766	-2.1977119	-2.8460997
H	4.8419567	-1.4995538	-6.7665387
H	-1.1223266	4.9430344	-6.7665387
H	-3.7196300	-3.4434806	-6.7665387
H	2.6315377	2.1059892	-5.4415387
H	-3.1396090	1.2259838	-5.4415387
H	0.5080713	-3.3319731	-5.4415387
H	3.4255302	-1.6994508	-5.6746573
H	-0.2409975	3.8163216	-5.6746573
H	-3.1845327	-2.1168708	-5.6746573
H	0.9561353	2.2813263	-4.8947013
H	-2.4537541	-0.3126257	-4.8947013
H	1.4976189	-1.9687006	-4.8947013
H	2.0285976	-0.1555011	-6.4153579
H	-0.8796309	1.8345675	-6.4153579
H	-1.1489666	-1.6790665	-6.4153579
H	1.1681729	1.2143831	-7.2023158
H	-1.6357730	0.4044758	-7.2023158
H	0.4676002	-1.6188589	-7.2023158
Bi	0.7197941	1.5483827	-0.0082299
Bi	0.9810417	-1.3975513	-0.0082299
Bi	-1.7008358	-0.1508314	-0.0082299

6a'(Th), Energy/eV = –169611.14688, lowest vibration frequency/cm^{-1} = 11

N	1.5555604	1.5073636	3.9394224
N	-2.0831954	0.5934730	3.9394224

N	0.5276350	-2.1008367	3.9394224
N	-0.0000000	0.0000000	5.8955097
Si	3.1980919	2.0330190	3.5769293
Si	-3.3596921	1.7531194	3.5769293
Si	0.1616001	-3.7861384	3.5769293
Th	0.0000000	0.0000000	2.9938335
C	2.7560256	4.1494356	1.5876180
C	3.3135334	3.8812668	2.9912792
C	5.3990533	1.3056744	1.8542394
C	2.6854851	4.8725128	3.9871608
C	5.5152950	2.9023661	5.1988871
C	3.9526669	0.9180129	2.2044976
C	4.2821115	1.9809934	5.2050604
C	3.8356356	-0.5960235	2.4387447
C	-1.4016463	3.6197696	2.4387447
C	4.6980856	0.5585203	5.6159001
C	-2.7713559	2.9641035	2.2044976
C	-3.8302739	4.0228801	1.8542394
C	0.9882153	2.1164120	5.1544429
C	2.2155038	-4.4615060	1.5876180
C	0.9105459	1.0903238	6.2832208
C	2.8769773	-4.7619547	3.9871608
C	-2.8327356	3.7894013	5.6159001
C	-4.9715294	0.3120704	1.5876180
C	1.7045090	-4.8102375	2.9912792
C	1.3387589	-1.9140255	5.1544429
C	-5.0180424	0.9289707	2.9912792
C	-3.8566464	2.7179207	5.2050604
C	0.4889751	-1.3337178	6.2832208
C	-1.1813110	-3.8821164	2.2044976
C	-1.3995210	0.2433940	6.2832208
C	-5.2711703	3.3252026	5.1988871
C	-2.3269742	-0.2023865	5.1544429
C	-2.4339893	-3.0237461	2.4387447
C	-5.5624624	-0.1105581	3.9871608
C	-1.5687794	-5.3285545	1.8542394
C	-0.4254652	-4.6989141	5.2050604
C	-0.2441247	-6.2275687	5.1988871
C	-1.8653500	-4.3479216	5.6159001
H	2.9205577	5.2129040	1.2865788
H	3.2251599	3.5082243	0.8119319
H	5.5017409	2.3873892	1.6161697
H	4.4114800	4.0843907	2.9601279
H	2.8869722	5.9277746	3.6814693
H	5.7574304	0.7357424	0.9648991
H	1.6609054	3.9557368	1.5505083
H	6.2241062	2.6389213	4.3820325
H	5.2465822	3.9727831	5.0703927
H	3.3135737	1.1409831	1.3147067

H	6.0995016	1.0773959	2.6898695
H	1.5790924	4.7501245	4.0185329
H	6.0801823	2.8167023	6.1593849
H	3.0669177	4.7495049	5.0257959
H	-1.0864654	4.1963179	1.5366962
H	4.1773506	-1.1572523	1.5366962
H	4.4444186	-0.9428705	3.3015876
H	5.4883835	0.1609167	4.9405331
H	3.6007457	2.3665988	6.0013316
H	-3.5158868	4.6182098	0.9648991
H	-0.5877777	2.8789212	2.6135121
H	-1.4056595	4.3204147	3.3015876
H	2.7871078	-0.9304302	2.6135121
H	5.1161437	0.5401514	6.6514893
H	1.5617653	2.9982384	5.5246409
H	-2.6449072	2.2991475	1.3147067
H	3.8499545	-0.1596773	5.5809436
H	-3.9828030	4.7436254	2.6898695
H	2.5953158	-3.4162547	1.5505083
H	-0.0418481	2.5056629	4.9618279
H	-4.8184101	3.5709528	1.6161697
H	3.0542285	-5.1357292	1.2865788
H	3.3241823	-3.7425964	4.0185329
H	1.9235804	0.6677055	6.4338028
H	1.4256314	-4.5471826	0.8119319
H	-4.6507913	1.0389583	0.8119319
H	-2.8835497	4.6726212	4.9405331
H	2.1908918	-1.2165899	4.9618279
H	3.6901173	-5.4640785	3.6814693
H	0.5867693	1.5539626	7.2509584
H	-1.7866926	3.4139970	5.5809436
H	-4.2562212	-0.5394821	1.5505083
H	-0.6686665	-3.4401306	1.3147067
H	2.5797331	-5.0307811	5.0257959
H	-5.3974259	4.0707734	4.3820325
H	1.8156680	-2.8516476	5.5246409
H	-3.0258567	4.1606347	6.6514893
H	-5.9747862	-0.0771748	1.2865788
H	-5.7429261	1.7782584	2.9601279
H	-1.5400401	1.3320167	6.4338028
H	1.3314461	-5.8626491	2.9601279
H	1.0523865	-1.2851384	7.2509584
H	-2.1993301	-1.9484910	2.6135121
H	-3.8499075	1.9350378	6.0013316
H	-5.4794269	3.8572412	6.1593849
H	-3.0908852	-3.0390656	1.5366962
H	-2.1490437	-1.2890730	4.9618279
H	-6.0638222	2.5572819	5.0703927
H	-0.3835402	-1.9997222	6.4338028

H	-3.3774333	-0.1465908	5.5246409
H	-1.6391557	-0.2688242	7.2509584
H	-0.6833308	-5.9583420	1.6161697
H	-4.9032747	-1.0075281	4.0185329
H	0.2491618	-4.3016366	6.0013316
H	-2.2415436	-5.3539522	0.9648991
H	-6.5770895	-0.4636960	3.6814693
H	-5.6466507	0.2812762	5.0257959
H	0.8172400	-6.5300650	5.0703927
H	-3.0387591	-3.3775442	3.3015876
H	-2.1166986	-5.8210213	2.6898695
H	-2.0632619	-3.2543197	5.5809436
H	-0.8266802	-6.7096947	4.3820325
H	-2.6048339	-4.8335379	4.9405331
H	-0.6007554	-6.6739435	6.1593849
H	-2.0902870	-4.7007861	6.6514893
N	2.0708416	0.6697816	-3.9555260
N	-1.6154687	1.4585106	-3.9555260
N	-0.4553729	-2.1282923	-3.9555260
N	0.0000000	0.0000000	-5.9110836
Si	3.7851882	0.4545564	-3.5985245
Si	-2.2862515	3.0507909	-3.5985245
Si	-1.4989367	-3.5053473	-3.5985245
Th	-0.0000000	-0.0000000	-3.0302921
C	4.1080067	2.5199686	-1.5923560
C	-4.2363602	2.2976539	-1.5923560
C	0.1283535	-4.8176225	-1.5923560
C	4.6240775	2.0835894	-2.9687597
C	-4.1164801	2.9627739	-2.9687597
C	-0.5075974	-5.0463632	-2.9687597
C	5.4228233	-0.9764847	-1.6745205
C	-1.8657511	5.1845451	-1.6745205
C	-3.5570722	-4.2080604	-1.6745205
C	4.5205507	3.2596759	-3.9552338
C	-5.0832375	2.2850738	-3.9552338
C	0.5626868	-5.5447497	-3.9552338
C	6.2741150	0.2483437	-5.1514711
C	-3.3521295	5.3093711	-5.1514711
C	-2.9219855	-5.5577148	-5.1514711
C	3.9904158	-0.8679208	-2.2224345
C	-1.2435665	3.8897619	-2.2224345
C	-2.7468494	-3.0218411	-2.2224345
C	4.7589608	-0.0128377	-5.2247714
C	-2.3683626	4.1277998	-5.2247714
C	-2.3905982	-4.1149621	-5.2247714
C	3.4390439	-2.2634051	-2.5579027
C	0.2406444	4.1100020	-2.5579027
C	-3.6796883	-1.8465969	-2.5579027
C	4.5015790	-1.4453179	-5.7231653

C	-0.9991075	4.6211407	-5.7231653
C	-3.5024715	-3.1758228	-5.7231653
C	1.8153099	1.4709420	-5.1668107
C	-2.1815281	0.8366335	-5.1668107
C	0.3662182	-2.3075755	-5.1668107
C	1.2956499	0.5861428	-6.2987173
C	-1.1554395	0.8289944	-6.2987173
C	-0.1402105	-1.4151371	-6.2987173
H	4.6445920	3.4281337	-1.2258586
H	-5.2911469	2.3082679	-1.2258586
H	0.6465548	-5.7364015	-1.2258586
H	4.2232212	1.7292736	-0.8205164
H	-3.6092054	2.7927801	-0.8205164
H	-0.6140158	-4.5220536	-0.8205164
H	5.8436719	0.0092751	-1.3789207
H	-2.9298684	5.0561308	-1.3789207
H	-2.9138035	-5.0654059	-1.3789207
H	5.7052138	1.8227385	-2.8645689
H	-4.4311447	4.0294908	-2.8645689
H	-1.2740691	-5.8522293	-2.8645689
H	5.1156116	4.1348077	-3.5981157
H	-6.1386544	2.3628458	-3.5981157
H	1.0230427	-6.4976535	-3.5981157
H	5.4483696	-1.6298508	-0.7697949
H	-1.3126926	5.5333519	-0.7697949
H	-4.1356770	-3.9035011	-0.7697949
H	3.0229433	2.7630369	-1.6378810
H	-3.9043318	1.2364273	-1.6378810
H	0.8813885	-3.9994642	-1.6378810
H	6.7564891	-0.3601204	-4.3533477
H	-3.0663711	6.0313514	-4.3533477
H	-3.6901180	-5.6712310	-4.3533477
H	6.5135209	1.3135468	-4.9447116
H	-4.3943253	4.9841012	-4.9447116
H	-2.1191956	-6.2976480	-4.9447116
H	3.3436135	-0.4618035	-1.4034467
H	-1.2718732	3.1265560	-1.4034467
H	-2.0717403	-2.6647525	-1.4034467
H	6.1164864	-1.4267530	-2.4214008
H	-1.8226389	6.0104091	-2.4214008
H	-4.2938476	-4.5836561	-2.4214008
H	3.4661062	3.6044843	-4.0496634
H	-4.8546280	1.1994939	-4.0496634
H	1.3885218	-4.8039782	-4.0496634
H	6.7735941	-0.0229145	-6.1135799
H	-3.3669525	5.8775619	-6.1135799
H	-3.4066416	-5.8546474	-6.1135799
H	4.8842987	3.0047974	-4.9762286
H	-5.0443803	2.7275281	-4.9762286

H	0.1600815	-5.7323255	-4.9762286
H	0.8045367	4.4226150	-1.6469711
H	-4.2323653	-1.5145582	-1.6469711
H	3.4278286	-2.9080567	-1.6469711
H	4.0539996	-2.7814879	-3.3252236
H	0.3818394	4.9016106	-3.3252236
H	-4.4358390	-2.1201227	-3.3252236
H	5.0556874	-2.1875698	-5.1068322
H	-0.6333526	5.4721386	-5.1068322
H	-4.4223347	-3.2845688	-5.1068322
H	4.3505542	0.6827647	-5.9974191
H	-2.7665687	3.4263081	-5.9974191
H	-1.5839855	-4.1090728	-5.9974191
H	0.7383605	3.1841140	-2.9232443
H	-3.1267039	-0.9526181	-2.9232443
H	2.3883434	-2.2314960	-2.9232443
H	4.8463776	-1.5771573	-6.7771201
H	-1.0573305	4.9856648	-6.7771201
H	-3.7890471	-3.4085075	-6.7771201
H	2.7114312	2.0219311	-5.5375482
H	-3.1067593	1.3372027	-5.5375482
H	0.3953281	-3.3591339	-5.5375482
H	3.4269461	-1.7280725	-5.6797675
H	-0.2169184	3.8318586	-5.6797675
H	-3.2100277	-2.1037861	-5.6797675
H	1.0544286	2.2663136	-4.9690496
H	-2.4898995	-0.2199948	-4.9690496
H	1.4354708	-2.0463188	-4.9690496
H	2.0225448	-0.2350013	-6.4548038
H	-0.8077553	1.8690758	-6.4548038
H	-1.2147895	-1.6340745	-6.4548038
H	1.2038301	1.1490052	-7.2633412
H	-1.5969828	0.4680449	-7.2633412
H	0.3931526	-1.6170501	-7.2633412
Bi	0.7772104	1.5238122	-0.0202330
Bi	0.9310548	-1.4349900	-0.0202330
Bi	-1.7082653	-0.0888221	-0.0202330

6b', Energy/eV = –161691.8316492, lowest vibration frequency/cm^{-1} = 16

C	-4.8633045	3.7558484	-5.0119393
C	4.7596615	-0.0729041	-6.2971836
C	-1.7570966	1.8011754	-5.0162268
C	1.3435062	-1.0247686	-5.0359604
C	-5.5151469	1.3446043	-4.6205329
C	5.2919421	-1.7378952	-4.4774296
C	-5.0255860	2.6445338	-3.9601944
C	4.9395903	-0.2729754	-4.7820560
C	-2.1473907	1.2704135	-3.6253808
C	1.9634186	-0.7680963	-3.6525997

C	-2.4777781	-0.2262220	-3.6915028
C	2.2021844	-2.0959013	-2.9203911
C	-2.6815335	4.1288831	-2.5450888
C	2.9010361	2.0403187	-4.5722997
C	-1.2795816	4.0253721	-1.9259759
C	1.6189172	2.5978050	-3.9439420
C	-3.5800055	5.0584847	-1.7116019
C	3.9803011	3.1342543	-4.6281082
C	4.0181400	-5.5468236	-0.4047810
C	-3.5498105	-4.0219709	-3.5208249
C	-5.2311017	2.2218609	-0.6977353
C	5.2852097	1.4072948	-1.9260363
C	-1.2516564	-3.6871064	-2.5136919
C	1.6496441	-4.7073387	-0.2865015
C	-6.3438336	1.1751688	-0.7623883
C	6.3652119	0.4898642	-1.3554951
C	-5.2201232	-1.7444516	-1.5720494
C	5.2932938	-2.3337495	-0.2694473
C	-2.6523361	-4.2796759	-2.2984576
C	2.9340167	-4.9560455	0.5120916
C	-6.3347910	-1.2845749	-0.6321464
C	6.3699367	-1.3769472	0.2391814
C	4.7945928	-5.3729968	3.2041032
C	-4.8239632	-6.2417712	-0.7456295
C	-4.9923386	-4.7773547	-0.3041655
C	4.9614080	-3.9592066	2.6195552
C	-2.4813135	3.3148022	1.6639265
C	2.1883064	3.5772465	-0.3774267
C	-6.3333482	0.0571714	1.4325588
C	6.3650671	0.9371530	1.0586394
C	-5.4813591	-4.7052784	1.1522447
C	5.3035624	-2.9606618	3.7371835
C	-5.5129681	3.3163312	3.4975599
C	5.2767669	4.7581588	0.6944638
C	-2.1199004	-3.7822128	0.7175910
C	1.9753517	-2.7603274	2.4794626
C	-2.1422676	2.5092049	2.9245990
C	1.9574488	3.5475223	1.1396758
C	-5.2126025	-0.5197677	2.2979283
C	5.2880001	0.9689685	2.1414586
C	-1.7245253	-5.2508119	0.9536960
C	1.3696105	-3.8339944	3.3981695
C	-1.7531032	3.4485432	4.0801040
C	1.3413417	4.8745474	1.6120394
C	-5.0144835	2.0964997	4.2906533
C	4.9367046	4.2827964	2.1162150
C	-2.4535740	-3.0916065	2.0462869
C	2.2012114	-1.4583561	3.2604670
C	-4.8511890	2.4505124	5.7791734

C	4.7613629	5.4911078	3.0530089
C	-2.6574399	0.1429636	4.8624011
C	2.9123710	2.9381238	4.0250164
C	-1.2537093	-0.3317502	4.4579556
C	1.6274368	2.1210227	4.1989578
C	-3.5457000	-1.0501278	5.2539777
C	3.9951454	2.4339710	4.9936018
H	-4.0275798	3.5423492	-5.7152945
H	3.8603424	-0.6075927	-6.6778800
H	-5.7898434	3.8611447	-5.6278369
H	5.6385235	-0.4711857	-6.8608359
H	-0.8996372	1.2257311	-5.4363408
H	0.3997756	-1.6123572	-4.9424721
H	-1.4563461	2.8718763	-4.9951117
H	1.0941970	-0.0828329	-5.5728448
H	-2.5978992	1.7022688	-5.7398315
H	2.0290131	-1.6119453	-5.6895527
H	-4.6598778	4.7458383	-4.5490970
H	4.6479771	0.9977964	-6.5734184
H	-4.8107461	1.0014356	-5.4109095
H	4.5222890	-2.4287229	-4.8883160
H	-6.5094713	1.4863349	-5.1093586
H	6.2682015	-2.0269966	-4.9363456
H	-2.5754411	4.5790639	-3.5612686
H	2.6688805	1.7350949	-5.6215237
H	-3.3597441	-0.4396929	-4.3342469
H	2.8498977	-2.7873675	-3.5031076
H	-1.6155230	-0.7995903	-4.1029007
H	1.2346520	-2.6092830	-2.7317009
H	-0.5512392	3.5600631	-2.6217751
H	0.7928490	1.8580437	-3.9366800
H	-0.8816787	5.0322562	-1.6508351
H	1.2547735	3.5024496	-4.4882147
H	-3.1312949	6.0772423	-1.6228127
H	3.6357013	4.0080940	-5.2325134
H	-5.8323013	2.9801794	-3.2651976
H	5.8165898	0.3479317	-4.4758174
H	-5.6136831	0.5119581	-3.8893643
H	5.3572743	-1.9370819	-3.3849121
H	-1.2617315	1.3648638	-2.9488793
H	1.2140723	-0.2082094	-3.0361522
H	-4.5963717	5.1800395	-2.1516684
H	4.9368063	2.7780665	-5.0734089
H	-2.6882257	-0.6702371	-2.6907993
H	2.6900061	-1.9725351	-1.9241350
H	-3.0986476	-4.4517433	-4.4475303
H	3.6810205	-6.5119559	-0.8547772
H	-3.6795229	-2.9309622	-3.6969445
H	4.2402833	-4.8570599	-1.2500339

H	-1.2780513	3.4012625	-1.0042311
H	1.7921237	2.8886484	-2.8874730
H	-3.7075147	4.6670440	-0.6778382
H	4.2081756	3.5168936	-3.6076973
H	-4.5652756	-4.4663762	-3.4081066
H	4.9764608	-5.7444714	0.1268246
H	-6.4731809	0.8780056	-1.8225666
H	6.4527122	-0.3883530	-2.0265375
H	-5.5942614	3.1500866	-1.2015720
H	5.6624786	1.8140615	-2.8952693
H	1.2949158	-5.6374946	-0.7927422
H	-0.8541340	-3.9401121	-3.5265358
H	-5.0724272	-0.9651491	-2.3598030
H	5.1834481	-2.2054490	-1.3749411
H	-1.2511684	-2.5777317	-2.4207533
H	1.8150544	-3.9407028	-1.0711704
H	-5.5780439	-2.6482785	-2.1218411
H	5.6741938	-3.3749539	-0.1353816
H	-7.3241838	1.5808859	-0.3994752
H	7.3677761	0.9912406	-1.3153276
H	-7.3168900	-1.1811548	-1.1633229
H	7.3734145	-1.5895690	-0.2146421
H	-2.5437713	-5.3847794	-2.1826430
H	2.7082565	-5.7106330	1.3042325
H	-5.0765533	2.5126428	0.3705613
H	5.1757569	2.2990266	-1.2601804
H	-0.5223207	-4.0648136	-1.7678344
H	0.8195733	-4.3366964	0.3485090
H	-4.6224755	-6.3310116	-1.8351966
H	4.6916921	-6.1492735	2.4154794
H	-5.8019129	-4.3459703	-0.9404431
H	5.8401211	-3.9962380	1.9304396
H	-2.6940492	2.6698660	0.7799407
H	2.6765015	2.6535161	-0.7697599
H	-1.6226962	3.9612102	1.3698859
H	1.2178789	3.6681465	-0.9115247
H	-6.4564772	-2.0546726	0.1560160
H	6.4578436	-1.5181419	1.3353774
H	-5.7473337	-6.8329330	-0.5292854
H	5.6756385	-5.6526304	3.8320463
H	-3.3649578	3.9748548	1.8055009
H	2.8325384	4.4287994	-0.6888804
H	-6.4631726	1.1233403	1.7069916
H	6.4481677	1.9579330	0.6338439
H	-3.9849082	-6.7426619	-0.2128134
H	3.8953240	-5.4440170	3.8566439
H	-5.6115563	3.1003102	2.4107404
H	5.3424641	3.9146063	-0.0277711
H	-7.3110777	-0.4626148	1.6094107

H	7.3696425	0.6547611	1.4699738
H	-6.4731237	-5.2047283	1.2732526
H	6.2808066	-3.2064173	4.2187320
H	-1.2349974	-3.2415347	0.2988491
H	1.2223616	-2.5166631	1.6867616
H	-4.8139726	4.1767740	3.5958185
H	4.5008798	5.4566569	0.3090920
H	5.3618616	-1.9142117	3.3644739
H	-5.5851457	-3.6570275	1.5099011
H	-1.4237482	-5.7672517	0.0155060
H	1.1310339	-4.7754543	2.8556746
H	-1.2533006	1.8823622	2.6648419
H	1.2086899	2.7341468	1.3203027
H	-6.5090020	3.6620013	3.8664888
H	6.2498335	5.3056857	0.6692879
H	-5.0556078	-1.5890020	2.0121191
H	5.1820780	-0.0538946	2.5807158
H	-0.9005959	4.1059814	3.7900585
H	0.3949834	5.0881266	1.0611978
H	-2.5968133	4.1189721	4.3612052
H	2.0267837	5.7332428	1.4258103
H	-4.7742484	-5.2159055	1.8435107
H	4.5326548	-2.9772811	4.5396271
H	-2.5625329	-5.8307318	1.4027896
H	2.0596481	-4.0938541	4.2337000
H	-2.6569493	-2.0016487	1.9306060
H	2.6773288	-0.6525920	2.6526611
H	-5.5688054	-0.5514148	3.3559340
H	5.6660738	1.6054591	2.9775198
H	-5.8169706	1.3223349	4.2352103
H	5.8187707	3.7088576	2.4916086
H	-0.8654454	-5.3234322	1.6605102
H	0.4221467	-3.4685211	3.8600109
H	-3.3404679	-3.5374684	2.5472656
H	2.8533262	-1.6084035	4.1490010
H	-1.4451990	2.8947570	4.9943632
H	1.0980720	4.8695061	2.6976524
H	1.2298417	-1.0491822	3.6132273
H	-1.5955510	-3.1663606	2.7533065
H	-5.7799851	2.9244089	6.1811655
H	5.6373179	6.1814863	2.9835809
H	-4.0199416	3.1716749	5.9459095
H	3.8574955	6.0864506	2.7916498
H	-1.2510556	-0.8097694	3.4526291
H	1.7920103	1.0612216	3.9151841
H	-0.5294745	0.5072442	4.4091633
H	0.7985491	2.4935591	3.5634740
H	-3.6701895	-1.7513516	4.3990094
H	4.2189586	1.3585379	4.8121181

H	-4.5635725	-0.7376051	5.5820488
H	4.9529166	2.9949973	4.9035709
H	-2.5531473	0.7993228	5.7595092
H	2.6859295	3.9997550	4.2885860
H	-4.6406820	1.5549660	6.4031208
H	4.6613973	5.1909242	4.1184075
H	-0.8511397	-1.0774278	5.1857966
H	1.2715499	2.1362173	5.2574015
H	-3.0896957	-1.6322903	6.0908074
H	3.6564726	2.5196197	6.0545382
Bi	-0.0034564	0.0020200	-0.0143460
N	-3.9956355	1.6760515	-1.2759376
N	4.0142354	0.6788990	-2.0420687
N	5.9633351	0.0158116	-0.0192224
N	-5.9497802	-0.0157881	0.0113558
N	-3.9822094	-1.9623738	-0.8113048
N	4.0211512	-2.0738109	0.4191567
N	-3.9812847	0.2591806	2.1080786
N	4.0139618	1.4310951	1.5732960
Si	-3.4770748	2.3847669	-2.8041479
Si	3.4717429	0.4115881	-3.6988255
Si	-3.4511257	-3.6362897	-0.6587442
Si	3.4896549	-3.3768247	1.4820956
Si	-3.4637768	1.2331316	3.4829543
Si	3.4723816	2.9979541	2.1746370
U	3.1343555	0.0111760	-0.0175907
U	-3.1072878	-0.0061827	0.0044237

16', Energy/eV = –155704.7594800, lowest vibration frequency/cm^{-1} = 7

C	-2.2744684	-1.3487774	-4.7934484
C	2.3457114	-2.1352186	-4.7031120
C	3.5884042	0.0202465	-4.3142543
C	-3.7281078	0.6331536	-4.2147157
C	-2.5921741	3.9615615	-2.8358895
C	-3.6075920	-0.8882005	-4.1818051
C	3.6064567	-1.4917048	-4.1015256
C	3.0971303	3.3543385	-3.0222080
C	-4.9049466	4.6014448	-1.9834534
C	1.8905327	4.9896578	-1.5186775
C	-3.8125462	3.5268154	-2.0107682
C	3.1406753	4.1146102	-1.6956143
C	5.4660573	-4.0341863	-1.8467385
C	-5.7470669	-3.3583038	-3.1789934
C	6.3850966	0.7797089	-1.7093992
C	-4.2769600	-3.2601797	-2.7499634
C	4.0553808	-3.7321842	-2.3578021
C	-6.4472114	1.0841776	-1.1318099
C	6.1117895	-0.7046290	-1.3572598
C	-1.7105559	4.8813616	0.5474743

C	5.8614701	1.7719020	-0.6462527
C	-6.1029284	-0.4181581	-1.2855610
C	2.9823013	-4.5544993	-1.6318913
C	4.7278254	5.0705721	0.9397835
C	-3.9765130	-4.1820460	-1.5674288
C	-3.1211102	4.3035411	0.7515179
C	-5.9159792	1.7079839	0.1836776
C	6.7244543	-0.9970919	0.0371498
C	3.7310399	3.9361569	1.1986872
C	6.4811593	1.4109163	0.7269572
C	-6.6413138	-1.1819646	-0.0484088
C	2.4960948	4.4050663	1.9829237
C	-6.4682470	0.8930452	1.3774704
C	6.2076085	-0.0529482	1.1536935
C	-3.3423769	3.9050051	2.2098520
C	-6.1341559	-0.6191043	1.3038071
C	4.0498643	-3.6655720	1.9898404
C	-4.1886121	-3.9380804	1.9969161
C	4.4458206	-2.6697390	3.0832809
C	5.9365214	-2.7626204	3.4319019
C	4.0189599	1.4459564	3.9962739
C	-3.9057204	-3.0707495	3.2202976
C	-3.5951288	1.0139752	4.3035444
C	3.9574720	-0.0636425	4.2298150
C	-4.0749908	-0.4380941	4.3341626
C	-5.5322196	-0.5447502	4.8040803
C	-2.6159635	-3.5182347	3.9258244
C	2.7154976	-0.4420033	5.0522416
H	-2.1822528	-1.0091353	-5.8517061
H	2.2778385	-1.9267007	-5.7963435
H	3.4611191	0.2731786	-5.3927341
H	-3.5902341	1.0241110	-5.2499654
H	-2.8727988	4.1134580	-3.9045087
H	-1.4196149	-0.9275199	-4.2148737
H	-2.1630752	-2.4559089	-4.7856570
H	2.9944314	4.0614098	-3.8784859
H	-2.9467314	1.0933851	-3.5677148
H	2.3214339	-3.2395717	-4.5711892
H	-5.2492846	4.8393904	-3.0183004
H	1.4335903	-1.7205903	-4.2147016
H	4.5196480	0.4996263	-3.9505603
H	2.7419481	0.4767384	-3.7511336
H	4.5156335	-1.9338870	-4.5748193
H	1.7436868	5.6507763	-2.4047682
H	-4.4510370	-1.3362839	-4.7567532
H	-1.7859920	3.1944990	-2.7934884
H	-2.1647021	4.9207119	-2.4675799
H	2.2301327	2.6544350	-3.0401940
H	-4.7181367	0.9708251	-3.8417457

H	4.0119665	2.7449329	-3.1726469
H	6.2462451	-3.5325910	-2.4595016
H	-4.2319015	2.6234534	-2.4996899
H	-4.5344797	5.5523652	-1.5385257
H	-5.9960569	-2.6625996	-4.0107088
H	4.0494406	4.7591205	-1.6781183
H	5.9283360	1.0213399	-2.6918251
H	4.0210268	-3.9893751	-3.4400826
H	-5.7936284	4.2778928	-1.3981426
H	-3.6363204	-3.5714952	-3.6075404
H	1.9512229	5.6430608	-0.6221706
H	0.9803472	4.3557928	-1.4102880
H	-6.0508391	1.6466322	-2.0032431
H	7.4879357	0.9176536	-1.8096991
H	5.5937682	-3.6887198	-0.7989375
H	-1.5394874	5.2335338	-0.4920213
H	6.6576875	-1.3300515	-2.1039400
H	-5.9924639	-4.3920855	-3.5211737
H	5.6664427	-5.1316138	-1.8674311
H	-6.6512236	-0.7894226	-2.1838666
H	-7.5584820	1.1886836	-1.1503074
H	3.1985162	-5.6476166	-1.6981178
H	6.2273818	2.7902154	-0.9309668
H	4.2740582	5.8866120	0.3334252
H	-6.4227693	-3.1252899	-2.3273962
H	-0.9391310	4.1065339	0.7638191
H	5.6335229	4.7132184	0.4013480
H	-1.5348165	5.7464142	1.2294046
H	-6.3366541	2.7414118	0.2680051
H	-3.8794394	5.0667373	0.4653453
H	-4.2004614	-5.2463080	-1.8154190
H	1.9724961	-4.3686292	-2.0587916
H	1.9397189	5.2064269	1.4480282
H	2.9211710	-4.2751028	-0.5576584
H	-2.9119890	-4.1048771	-1.2537969
H	7.8323037	-0.8819331	-0.0359494
H	5.0643932	5.5277990	1.9009666
H	7.5862837	1.5574188	0.6697141
H	-7.7560580	-1.1240479	-0.0530193
H	-4.5979956	-3.9022957	-0.6911378
H	6.5172800	-2.0511072	0.3180363
H	-6.3595336	-2.2543402	-0.1260901
H	1.7875177	3.5610904	2.1447107
H	-7.5778117	1.0123606	1.4003378
H	6.0965118	2.1035407	1.5052126
H	4.2366775	3.1677375	1.8216967
H	2.7913833	4.8108652	2.9791592
H	-2.6378417	3.0935023	2.4997054
H	-3.1684542	4.7745747	2.8861019

H	-4.3726922	3.5263650	2.3778793
H	-6.0651167	1.3152976	2.3221789
H	6.8107590	-0.2496466	2.0726122
H	4.5661707	-3.4189560	1.0360495
H	-5.1357417	-3.6424317	1.4980543
H	-6.7070842	-1.1372339	2.1106237
H	-3.3699742	-3.8277247	1.2509164
H	4.9448901	1.7388639	3.4581053
H	6.5634959	-2.6010910	2.5281920
H	2.9573064	-3.6225474	1.7844679
H	4.3239625	-4.7110455	2.2647660
H	-4.1857526	1.6043549	3.5716029
H	-4.2570509	-5.0157029	2.2757034
H	-2.5307894	1.0793279	3.9863443
H	3.1517844	1.7766237	3.3795733
H	6.1900285	-3.7723698	3.8341466
H	4.8751773	-0.3903558	4.7720344
H	-6.2043741	0.0594041	4.1571786
H	-4.7609000	-3.1467463	3.9309612
H	3.9837022	2.0004829	4.9629988
H	6.2409299	-2.0121308	4.1948172
H	3.8490323	-2.8951098	3.9973241
H	-3.7031131	1.4999935	5.3016830
H	-1.7462819	-3.4177824	3.2365817
H	-5.9072177	-1.5920405	4.7978654
H	-3.4322884	-1.0027917	5.0491261
H	-2.6866981	-4.5842045	4.2462500
H	1.7897287	-0.1493834	4.5052914
H	-5.6409932	-0.1567836	5.8449791
H	-2.3933357	-2.9107747	4.8309263
H	2.6531843	-1.5351661	5.2489749
H	2.7176241	0.0774368	6.0390376
Bi	-0.0254874	0.5400050	-1.5586965
Bi	0.0150557	-1.8870509	0.2547113
Bi	0.0321225	0.8906630	1.4517527
N	4.6859079	-1.0048099	-1.3678074
N	-4.6679986	-0.6308764	-1.4267700
N	4.4079161	1.7377836	-0.5953371
N	-4.4604012	1.7353160	0.2062853
N	4.7890288	-0.2713170	1.4056340
N	-4.7029971	-0.8403026	1.4604184
P	-3.6636594	-1.5129390	-2.4010771
P	3.6129540	-1.8875986	-2.2616117
P	-3.3087110	2.7899829	-0.3527338
P	3.2447161	2.8773406	-0.2851617
P	3.8521198	-0.9585975	2.5777562
P	-3.6873540	-1.2748338	2.6921679
Th	2.9592790	-0.0421261	-0.0698155
Th	-2.9421887	-0.0733091	0.0869535

Optimised Cartesian coordinates for the tri-anionic three-membered systems in Ångstrom (PBE+D3-BJ, gridsize 5 plus weight derivatives; bases: def2-TZVP).

Bi_3^{3-}, bent: E/eV = –17535.4258778, lowest frq./cm^{-1} = 42.20

Bi	2.4845662	0.0000000	-0.5144536
Bi	-2.4845662	0.0000000	-0.5144536
Bi	0.0000000	0.0000000	1.0289072

Bi_3^{3-}, linear: E/eV = –17534.4071376, lowest frq./cm^{-1} = –116.08

Bi	0.0000000	0.0000000	0.0000000
Bi	0.0000000	0.0000000	3.0270158
Bi	0.0000000	0.0000000	-3.0270158

Bi_3^{3-}, triangle: E/eV = –17535.5269082, lowest frq./cm^{-1} = 92.09

Bi	0.8910676	-1.5433744	0.0000000
Bi	0.8910676	1.5433744	0.0000000
Bi	-1.7821353	0.0000000	0.0000000

$C_3H_3^{3-}$, bent: E/eV = –3158.3522948, lowest frq./cm^{-1} = 490.62

C	1.3110397	0.0000000	-0.6600023
C	0.0000000	0.0000000	-0.0769202
C	-1.3110397	0.0000000	-0.6600023
H	2.0707507	0.0000000	0.1683021
H	0.0000000	0.0000000	1.0603207
H	-2.0707507	0.0000000	0.1683021

$C_3H_3^{3-}$, triangle: E/eV = –3152.7866507, lowest frq./cm^{-1} = –1536.51

C	0.8782848	0.0000000	0.0000000
C	-0.4391424	0.7606169	0.0000000
C	-0.4391424	-0.7606169	0.0000000
H	1.9664513	0.0000000	0.0000000
H	-0.9832257	1.7029968	0.0000000
H	-0.9832257	-1.7029968	0.0000000

$C_3H_2^{2-}$, bent: E/eV = –3141.5054265, lowest frq./cm^{-1} = 424.21

C	1.3186593	-0.0000000	-0.6154048
C	0.0000306	0.0000000	-0.4697991
C	-1.3185731	0.0000000	-0.6152559
H	2.0611045	0.0000000	0.1868146
H	-2.0612213	-0.0000000	0.1867446

Po_3, bent: E/eV = –19418.7395030, lowest frq./cm^{-1} = 45.49

Po	2.3178343	0.0000000	-0.5209029
Po	-2.3178343	0.0000000	-0.5209029
Po	0.0000000	0.0000000	1.0418059

Po_3, linear: E/eV = –19417.5957949, lowest frq./cm^{-1} = –83.53

Po	0.0000000	0.0000000	-0.0000000
Po	0.0000000	0.0000000	2.8852887
Po	0.0000000	0.0000000	-2.8852887

Po_3, triangle: E/eV = –19419.0857363, lowest frq./cm^{-1} = 120.31

Po	0.8490493	-1.4705965	0.0000000
Po	0.8490493	1.4705965	0.0000000
Po	-1.6980985	0.0000000	0.0000000

Sb_3^{3-}, bent: E/eV = –19623.7062496, lowest frq./cm^{-1} = 66.71

Sb	2.3398172	0.0000000	-0.4877541

Sb -2.3398172 0.0000000 -0.4877541
Sb 0.0000000 0.0000000 0.9755082
Sb_3^{3-}, linear: E/eV = –19622.5318490, lowest frq./cm^{-1} = 187.98 (triplet)
Sb 0.0000000 0.0000000 0.0000000
Sb 0.0000000 0.0000000 2.7813427
Sb 0.0000000 0.0000000 -2.7813427
Sb_3^{3-}, triangle: E/eV = –19623.7394641, lowest frq./cm^{-1} = 127.47
Sb 0.8423887 -1.4590600 0.0000000
Sb 0.8423887 1.4590600 0.0000000
Sb -1.6847774 0.0000000 0.0000000
Te_3, bent: E/eV = –21881.4263716, lowest frq./cm^{-1} = 67.06
Te 2.1901916 0.0000000 -0.4784449
Te -2.1901916 0.0000000 -0.4784449
Te 0.0000000 0.0000000 0.9568897
Te_3, linear: E/eV = –21880.0142923, lowest frq./cm^{-1} = –139.52
Te 0.0000000 0.0000000 0.0000000
Te 0.0000000 0.0000000 2.7293202
Te 0.0000000 0.0000000 -2.7293202
Te_3, triangle: E/eV = –21881.6851715, lowest frq./cm^{-1} = 167.81
Te 0.7986343 -1.3832752 0.0000000
Te 0.7986343 1.3832752 0.0000000
Te -1.5972686 0.0000000 0.0000000

Optimised Cartesian coordinates for the mono-cationic three-membered systems in Ångstrom (PBE+D3-BJ, gridsize 5 plus weight derivatives; bases: def2-TZVP)
Bi_3^{+}, linear: E/eV = –17520.9932838, lowest frq./cm^{-1} = 12.16 (triplet)
Bi 0.0000000 0.0000000 0.0000000
Bi 0.0000000 0.0000000 2.7361427
Bi 0.0000000 0.0000000 -2.7361427
Bi_3+, triangle: E/eV = –17522.9623726, lowest frq./cm^{-1} = 122.96
Bi 0.8342234 -1.4449173 0.0000000
Bi 0.8342234 1.4449173 0.0000000
Bi -1.6684468 0.0000000 0.0000000
$C_3H_3^{+}$, bent: E/eV = –3144.7389474, lowest frq./cm^{-1} = 82.05 (triplet)
C 1.1661955 0.0000000 -0.4842008
C 0.0000000 0.0000000 0.2161372
C -1.1661955 0.0000000 -0.4842008
H 2.2423784 0.0000000 -0.2873181
H 0.0000000 0.0000000 1.3269006
H -2.2423784 0.0000000 -0.2873181
$C_3H_3^{+}$, triangle: E/eV = –3149.0094177, lowest frq./cm^{-1} = 706.63
C 0.7881949 0.0000000 0.0000000
C -0.3940974 0.6825968 0.0000000
C -0.3940974 -0.6825968 0.0000000
H 1.8759376 0.0000000 0.0000000
H -0.9379688 1.6246097 0.0000000
H -0.9379688 -1.6246097 0.0000000
Sb_3^{+}, linear: E/eV = –19608.6484957, lowest frq./cm^{-1} = 18.67 (triplet)
Sb 0.0000000 0.0000000 0.0000000

Sb	0.0000000	0.0000000	2.5847455
Sb	0.0000000	0.0000000	-2.5847455

Sb_3^+, triangle: E/eV = –19610.5745148, lowest frq./cm^{-1} = 173.40

Sb	0.7901394	-1.3685617	0.0000000
Sb	0.7901394	1.3685617	0.0000000
Sb	-1.5802789	0.0000000	0.0000000

***Computed frequencies for* 6a', 6b', and 16'**

6a'

mode	symmetry	wave number	IR intensity	selection rules	
		cm**(-1)	km/mol	IR	RAMAN
1		-0.00	0.00000	-	-
2		-0.00	0.00000	-	-
3		-0.00	0.00000	-	-
4		0.00	0.00000	-	-
5		0.00	0.00000	-	-
6		0.00	0.00000	-	-
7	a	13.32	0.00034	YES	YES
8	e	24.71	0.07319	YES	YES
9	e	24.71	0.07319	YES	YES
10	e	25.95	0.07405	YES	YES
11	e	25.95	0.07405	YES	YES
12	a	26.68	0.06515	YES	YES
13	e	30.84	0.02531	YES	YES
14	e	30.84	0.02531	YES	YES
15	a	38.90	0.00079	YES	YES
16	e	43.86	0.00370	YES	YES
17	e	43.86	0.00370	YES	YES
18	e	46.38	0.01462	YES	YES
19	e	46.38	0.01462	YES	YES
20	a	48.54	0.05898	YES	YES
21	a	49.75	0.04555	YES	YES
22	e	52.41	0.01367	YES	YES
23	e	52.41	0.01367	YES	YES
24	a	52.62	0.13993	YES	YES
25	e	58.24	0.00758	YES	YES
26	e	58.24	0.00758	YES	YES
27	a	58.83	0.15290	YES	YES
28	a	61.25	0.07105	YES	YES
29	e	64.13	0.00271	YES	YES
30	e	64.13	0.00271	YES	YES
31	a	68.15	0.00561	YES	YES
32	e	69.59	0.01999	YES	YES
33	e	69.59	0.01999	YES	YES
34	a	72.74	0.12857	YES	YES
35	e	74.98	0.01441	YES	YES
36	e	74.98	0.01441	YES	YES
37	e	82.24	0.00125	YES	YES
38	e	82.24	0.00125	YES	YES

39	e	83.74	0.01978	YES	YES
40	e	83.74	0.01978	YES	YES
41	a	86.72	0.07888	YES	YES
42	a	88.33	1.32050	YES	YES
43	e	90.46	0.76444	YES	YES
44	e	90.46	0.76444	YES	YES
45	a	93.00	0.23317	YES	YES
46	e	93.76	0.09907	YES	YES
47	e	93.76	0.09907	YES	YES
48	e	96.64	0.33662	YES	YES
49	e	96.64	0.33662	YES	YES
50	a	96.82	0.49570	YES	YES
51	a	103.38	0.19657	YES	YES
52	a	104.47	1.78977	YES	YES
53	e	104.69	0.96475	YES	YES
54	e	104.69	0.96475	YES	YES
55	a	113.11	0.03928	YES	YES
56	e	113.62	0.28792	YES	YES
57	e	113.62	0.28792	YES	YES
58	e	115.94	0.08415	YES	YES
59	e	115.94	0.08415	YES	YES
60	a	116.33	5.35307	YES	YES
61	e	121.42	0.43493	YES	YES
62	e	121.42	0.43493	YES	YES
63	a	125.87	0.07891	YES	YES
64	e	126.40	8.34147	YES	YES
65	e	126.40	8.34147	YES	YES
66	a	126.95	6.04602	YES	YES
67	e	129.39	0.52209	YES	YES
68	e	129.39	0.52209	YES	YES
69	a	136.24	0.54586	YES	YES
70	e	137.09	0.11911	YES	YES
71	e	137.09	0.11911	YES	YES
72	a	140.25	1.13059	YES	YES
73	e	141.90	0.11140	YES	YES
74	e	141.90	0.11140	YES	YES
75	e	146.87	3.84600	YES	YES
76	e	146.87	3.84600	YES	YES
77	a	149.19	12.66343	YES	YES
78	e	150.74	2.95912	YES	YES
79	e	150.74	2.95912	YES	YES
80	a	151.62	0.58217	YES	YES
81	a	154.84	1.78771	YES	YES
82	a	158.81	7.02360	YES	YES
83	a	167.90	0.30834	YES	YES
84	e	169.07	0.29895	YES	YES
85	e	169.07	0.29895	YES	YES
86	e	171.93	0.62146	YES	YES
87	e	171.93	0.62146	YES	YES

88	a	173.12	7.70359	YES	YES
89	a	174.51	2.52673	YES	YES
90	e	180.59	2.01943	YES	YES
91	e	180.59	2.01943	YES	YES
92	e	183.56	2.49834	YES	YES
93	e	183.56	2.49834	YES	YES
94	a	187.09	6.06275	YES	YES
95	a	188.96	2.75491	YES	YES
96	e	192.06	5.71578	YES	YES
97	e	192.06	5.71578	YES	YES
98	e	197.70	9.09696	YES	YES
99	e	197.70	9.09696	YES	YES
100	a	209.27	0.11637	YES	YES
101	e	211.19	4.79092	YES	YES
102	e	211.19	4.79092	YES	YES
103	a	212.34	0.65821	YES	YES
104	e	214.03	1.61553	YES	YES
105	e	214.03	1.61553	YES	YES
106	a	215.37	11.91441	YES	YES
107	e	216.36	1.72310	YES	YES
108	e	216.36	1.72310	YES	YES
109	a	223.75	5.93221	YES	YES
110	e	230.30	0.43750	YES	YES
111	e	230.30	0.43750	YES	YES
112	a	230.49	0.46385	YES	YES
113	e	231.27	0.05720	YES	YES
114	e	231.27	0.05720	YES	YES
115	e	243.93	6.69330	YES	YES
116	e	243.93	6.69330	YES	YES
117	e	248.35	2.45170	YES	YES
118	e	248.35	2.45170	YES	YES
119	a	250.08	2.51801	YES	YES
120	a	252.11	0.01813	YES	YES
121	e	252.73	0.61833	YES	YES
122	e	252.73	0.61833	YES	YES
123	a	253.10	0.03489	YES	YES
124	e	254.20	2.18970	YES	YES
125	e	254.20	2.18970	YES	YES
126	e	260.58	4.76258	YES	YES
127	e	260.58	4.76258	YES	YES
128	a	260.65	5.66413	YES	YES
129	a	262.74	24.22939	YES	YES
130	e	262.89	0.24920	YES	YES
131	e	262.89	0.24920	YES	YES
132	a	264.40	3.74257	YES	YES
133	a	265.99	1.49027	YES	YES
134	e	270.99	0.64026	YES	YES
135	e	270.99	0.64026	YES	YES
136	a	272.52	0.87161	YES	YES

137	a	272.67	0.62011	YES	YES
138	e	273.26	0.26376	YES	YES
139	e	273.26	0.26376	YES	YES
140	e	273.96	0.20197	YES	YES
141	e	273.96	0.20197	YES	YES
142	e	275.03	0.09389	YES	YES
143	e	275.03	0.09389	YES	YES
144	a	275.56	1.14581	YES	YES
145	a	278.45	0.01206	YES	YES
146	e	278.93	0.78090	YES	YES
147	e	278.93	0.78090	YES	YES
148	a	280.15	0.00001	YES	YES
149	e	280.75	0.40943	YES	YES
150	e	280.75	0.40943	YES	YES
151	a	284.67	2.43122	YES	YES
152	a	286.13	2.15399	YES	YES
153	e	290.18	0.34208	YES	YES
154	e	290.18	0.34208	YES	YES
155	a	290.97	1.59514	YES	YES
156	e	296.64	0.14868	YES	YES
157	e	296.64	0.14868	YES	YES
158	e	298.16	0.68082	YES	YES
159	e	298.16	0.68082	YES	YES
160	a	299.49	0.02206	YES	YES
161	a	303.88	0.15694	YES	YES
162	e	316.80	0.01781	YES	YES
163	e	316.80	0.01781	YES	YES
164	e	317.23	0.48254	YES	YES
165	e	317.23	0.48254	YES	YES
166	a	321.18	4.08824	YES	YES
167	a	322.09	1.28492	YES	YES
168	e	328.12	1.42729	YES	YES
169	e	328.12	1.42729	YES	YES
170	e	334.41	2.24836	YES	YES
171	e	334.41	2.24836	YES	YES
172	a	348.75	16.08494	YES	YES
173	a	350.07	4.11361	YES	YES
174	e	362.75	6.72735	YES	YES
175	e	362.75	6.72735	YES	YES
176	e	364.86	10.09548	YES	YES
177	e	364.86	10.09548	YES	YES
178	a	387.70	4.09487	YES	YES
179	a	389.10	1.07078	YES	YES
180	e	389.98	0.23071	YES	YES
181	e	389.98	0.23071	YES	YES
182	e	390.18	9.86613	YES	YES
183	e	390.18	9.86613	YES	YES
184	a	390.83	0.37640	YES	YES
185	a	392.03	0.39573	YES	YES

186	e	405.58	1.29379	YES	YES
187	e	405.58	1.29379	YES	YES
188	e	407.11	0.68924	YES	YES
189	e	407.11	0.68924	YES	YES
190	e	417.74	0.32327	YES	YES
191	e	417.74	0.32327	YES	YES
192	a	418.09	0.50169	YES	YES
193	e	419.78	1.81214	YES	YES
194	e	419.78	1.81214	YES	YES
195	a	419.80	0.06763	YES	YES
196	a	421.69	56.85774	YES	YES
197	a	422.57	8.23170	YES	YES
198	e	441.27	2.40418	YES	YES
199	e	441.27	2.40418	YES	YES
200	e	441.45	3.00839	YES	YES
201	e	441.45	3.00839	YES	YES
202	a	501.55	0.04553	YES	YES
203	e	502.75	13.27040	YES	YES
204	e	502.75	13.27040	YES	YES
205	a	503.06	0.11613	YES	YES
206	e	503.73	25.04565	YES	YES
207	e	503.73	25.04565	YES	YES
208	e	525.72	0.51254	YES	YES
209	e	525.72	0.51254	YES	YES
210	e	525.87	2.24285	YES	YES
211	e	525.87	2.24285	YES	YES
212	a	531.56	2.75131	YES	YES
213	a	532.89	2.78088	YES	YES
214	e	537.96	0.74616	YES	YES
215	e	537.96	0.74616	YES	YES
216	e	538.11	16.04340	YES	YES
217	e	538.11	16.04340	YES	YES
218	a	551.71	47.29762	YES	YES
219	a	553.98	32.43445	YES	YES
220	e	597.71	17.88976	YES	YES
221	e	597.71	17.88976	YES	YES
222	a	598.14	45.16105	YES	YES
223	e	605.81	20.12613	YES	YES
224	e	605.81	20.12613	YES	YES
225	a	606.28	48.51136	YES	YES
226	e	653.35	8.64367	YES	YES
227	e	653.35	8.64367	YES	YES
228	a	653.95	279.51369	YES	YES
229	e	655.01	17.63894	YES	YES
230	e	655.01	17.63894	YES	YES
231	a	656.28	25.20935	YES	YES
232	a	702.84	250.62475	YES	YES
233	a	710.76	0.81716	YES	YES
234	e	725.10	72.75019	YES	YES

235	e	725.10	72.75019	YES	YES
236	e	730.30	777.83980	YES	YES
237	e	730.30	777.83980	YES	YES
238	a	768.08	1055.72924	YES	YES
239	a	788.69	4.65404	YES	YES
240	e	873.82	27.10404	YES	YES
241	e	873.82	27.10404	YES	YES
242	e	876.89	58.68660	YES	YES
243	e	876.89	58.68660	YES	YES
244	a	884.84	13.14201	YES	YES
245	e	885.10	20.00097	YES	YES
246	e	885.10	20.00097	YES	YES
247	a	886.91	0.23863	YES	YES
248	e	887.06	23.75000	YES	YES
249	e	887.06	23.75000	YES	YES
250	a	887.21	36.11627	YES	YES
251	e	887.77	1.58000	YES	YES
252	e	887.77	1.58000	YES	YES
253	a	888.41	5.31186	YES	YES
254	e	889.71	6.17667	YES	YES
255	e	889.71	6.17667	YES	YES
256	a	890.56	2.23533	YES	YES
257	a	893.01	0.78767	YES	YES
258	e	893.72	1.75605	YES	YES
259	e	893.72	1.75605	YES	YES
260	a	893.80	0.52106	YES	YES
261	e	894.48	9.86182	YES	YES
262	e	894.48	9.86182	YES	YES
263	a	895.15	3.06123	YES	YES
264	e	897.00	5.41057	YES	YES
265	e	897.00	5.41057	YES	YES
266	a	897.11	16.07910	YES	YES
267	e	898.14	23.83232	YES	YES
268	e	898.14	23.83232	YES	YES
269	a	898.81	0.96054	YES	YES
270	e	901.81	2.87238	YES	YES
271	e	901.81	2.87238	YES	YES
272	a	901.99	3.28969	YES	YES
273	e	903.50	0.53887	YES	YES
274	e	903.50	0.53887	YES	YES
275	a	903.72	2.46378	YES	YES
276	e	905.50	6.88220	YES	YES
277	e	905.50	6.88220	YES	YES
278	a	905.55	0.17936	YES	YES
279	e	908.02	0.73254	YES	YES
280	e	908.02	0.73254	YES	YES
281	a	908.03	3.16883	YES	YES
282	e	922.81	5.67038	YES	YES
283	e	922.81	5.67038	YES	YES

284	e	923.85	163.58239	YES	YES
285	e	923.85	163.58239	YES	YES
286	e	957.67	1.53628	YES	YES
287	e	957.67	1.53628	YES	YES
288	a	957.77	0.00005	YES	YES
289	e	957.99	0.19978	YES	YES
290	e	957.99	0.19978	YES	YES
291	a	958.00	0.28696	YES	YES
292	a	958.48	0.37242	YES	YES
293	e	958.62	0.12054	YES	YES
294	e	958.62	0.12054	YES	YES
295	e	959.51	6.10910	YES	YES
296	e	959.51	6.10910	YES	YES
297	a	959.67	0.82226	YES	YES
298	e	959.93	0.45064	YES	YES
299	e	959.93	0.45064	YES	YES
300	a	959.95	1.88669	YES	YES
301	a	963.59	1.42877	YES	YES
302	e	963.70	0.58464	YES	YES
303	e	963.70	0.58464	YES	YES
304	e	976.84	0.79225	YES	YES
305	e	976.84	0.79225	YES	YES
306	a	976.88	0.84553	YES	YES
307	a	979.85	0.92235	YES	YES
308	e	979.93	0.44165	YES	YES
309	e	979.93	0.44165	YES	YES
310	a	984.82	13.41732	YES	YES
311	e	985.16	26.97239	YES	YES
312	e	985.16	26.97239	YES	YES
313	a	985.21	19.99692	YES	YES
314	e	985.77	3.94833	YES	YES
315	e	985.77	3.94833	YES	YES
316	a	999.29	1.44175	YES	YES
317	a	1001.31	1.30025	YES	YES
318	e	1004.00	28.38582	YES	YES
319	e	1004.00	28.38582	YES	YES
320	a	1004.19	9.94253	YES	YES
321	e	1008.60	34.89822	YES	YES
322	e	1008.60	34.89822	YES	YES
323	a	1009.44	6.53734	YES	YES
324	e	1018.25	13.28956	YES	YES
325	e	1018.25	13.28956	YES	YES
326	e	1019.41	24.33202	YES	YES
327	e	1019.41	24.33202	YES	YES
328	a	1040.64	49.32968	YES	YES
329	a	1044.46	47.25029	YES	YES
330	a	1052.31	125.89111	YES	YES
331	a	1052.56	2.48219	YES	YES
332	e	1052.61	11.60179	YES	YES

333	e	1052.61	11.60179	YES	YES
334	e	1054.22	16.86108	YES	YES
335	e	1054.22	16.86108	YES	YES
336	e	1078.54	0.02308	YES	YES
337	e	1078.54	0.02308	YES	YES
338	a	1078.61	15.66817	YES	YES
339	e	1079.23	0.28923	YES	YES
340	e	1079.23	0.28923	YES	YES
341	a	1079.33	0.37460	YES	YES
342	e	1090.40	6.03456	YES	YES
343	e	1090.40	6.03456	YES	YES
344	a	1090.53	3.23379	YES	YES
345	e	1090.61	4.60296	YES	YES
346	e	1090.61	4.60296	YES	YES
347	a	1090.68	0.03485	YES	YES
348	e	1097.97	1.40535	YES	YES
349	e	1097.97	1.40535	YES	YES
350	a	1098.14	3.17830	YES	YES
351	e	1098.46	1.27925	YES	YES
352	e	1098.46	1.27925	YES	YES
353	a	1098.57	7.21481	YES	YES
354	e	1140.38	5.81333	YES	YES
355	e	1140.38	5.81333	YES	YES
356	e	1141.36	8.14797	YES	YES
357	e	1141.36	8.14797	YES	YES
358	a	1148.03	0.02246	YES	YES
359	e	1148.32	0.57365	YES	YES
360	e	1148.32	0.57365	YES	YES
361	a	1148.77	0.06970	YES	YES
362	e	1149.48	0.10154	YES	YES
363	e	1149.48	0.10154	YES	YES
364	a	1150.32	0.15990	YES	YES
365	e	1150.75	0.12350	YES	YES
366	e	1150.75	0.12350	YES	YES
367	a	1151.06	0.47109	YES	YES
368	e	1151.34	0.06634	YES	YES
369	e	1151.34	0.06634	YES	YES
370	a	1153.99	0.00941	YES	YES
371	e	1154.15	1.57252	YES	YES
372	e	1154.15	1.57252	YES	YES
373	a	1155.58	0.26625	YES	YES
374	e	1155.65	0.37370	YES	YES
375	e	1155.65	0.37370	YES	YES
376	a	1205.81	0.01587	YES	YES
377	a	1207.13	0.12678	YES	YES
378	e	1216.03	0.42247	YES	YES
379	e	1216.03	0.42247	YES	YES
380	e	1216.22	0.17141	YES	YES
381	e	1216.22	0.17141	YES	YES

382	a	1216.36	7.07167	YES	YES
383	a	1216.60	0.64451	YES	YES
384	a	1217.78	1.77098	YES	YES
385	e	1217.97	2.05954	YES	YES
386	e	1217.97	2.05954	YES	YES
387	e	1219.32	3.20253	YES	YES
388	e	1219.32	3.20253	YES	YES
389	a	1219.37	0.13542	YES	YES
390	e	1230.06	11.61953	YES	YES
391	e	1230.06	11.61953	YES	YES
392	e	1231.20	7.70936	YES	YES
393	e	1231.20	7.70936	YES	YES
394	a	1233.41	15.29129	YES	YES
395	e	1234.55	17.07371	YES	YES
396	e	1234.55	17.07371	YES	YES
397	a	1239.18	16.08753	YES	YES
398	e	1239.62	13.38335	YES	YES
399	e	1239.62	13.38335	YES	YES
400	a	1253.01	16.77095	YES	YES
401	a	1253.17	4.19576	YES	YES
402	e	1266.39	4.34879	YES	YES
403	e	1266.39	4.34879	YES	YES
404	e	1266.54	20.99718	YES	YES
405	e	1266.54	20.99718	YES	YES
406	a	1283.77	1.14114	YES	YES
407	e	1283.92	5.21571	YES	YES
408	e	1283.92	5.21571	YES	YES
409	a	1284.69	3.44161	YES	YES
410	e	1285.09	0.24547	YES	YES
411	e	1285.09	0.24547	YES	YES
412	e	1286.93	0.08007	YES	YES
413	e	1286.93	0.08007	YES	YES
414	a	1287.11	0.01852	YES	YES
415	e	1287.39	6.37788	YES	YES
416	e	1287.39	6.37788	YES	YES
417	a	1287.85	0.55491	YES	YES
418	e	1287.97	3.82262	YES	YES
419	e	1287.97	3.82262	YES	YES
420	a	1288.36	3.40108	YES	YES
421	e	1288.77	1.06633	YES	YES
422	e	1288.77	1.06633	YES	YES
423	a	1289.19	0.84478	YES	YES
424	e	1312.08	0.64578	YES	YES
425	e	1312.08	0.64578	YES	YES
426	e	1312.82	0.51168	YES	YES
427	e	1312.82	0.51168	YES	YES
428	a	1317.95	104.41051	YES	YES
429	a	1319.07	22.65162	YES	YES
430	e	1339.58	0.71999	YES	YES

431	e	1339.58	0.71999	YES	YES
432	a	1339.76	0.59024	YES	YES
433	e	1340.72	0.13238	YES	YES
434	e	1340.72	0.13238	YES	YES
435	a	1340.76	0.24783	YES	YES
436	a	1345.69	0.26587	YES	YES
437	a	1345.96	0.02287	YES	YES
438	e	1346.21	1.89491	YES	YES
439	e	1346.21	1.89491	YES	YES
440	e	1346.70	7.32717	YES	YES
441	e	1346.70	7.32717	YES	YES
442	a	1347.55	14.48258	YES	YES
443	e	1347.95	1.64400	YES	YES
444	e	1347.95	1.64400	YES	YES
445	e	1349.01	1.76320	YES	YES
446	e	1349.01	1.76320	YES	YES
447	a	1349.17	6.70333	YES	YES
448	a	1350.10	3.74612	YES	YES
449	a	1350.32	0.95903	YES	YES
450	e	1351.56	1.42732	YES	YES
451	e	1351.56	1.42732	YES	YES
452	e	1352.29	1.30366	YES	YES
453	e	1352.29	1.30366	YES	YES
454	a	1358.97	0.50413	YES	YES
455	e	1359.32	0.49377	YES	YES
456	e	1359.32	0.49377	YES	YES
457	a	1360.15	0.47525	YES	YES
458	e	1360.93	1.12525	YES	YES
459	e	1360.93	1.12525	YES	YES
460	e	1363.17	3.26249	YES	YES
461	e	1363.17	3.26249	YES	YES
462	e	1364.09	3.08736	YES	YES
463	e	1364.09	3.08736	YES	YES
464	a	1364.94	1.30297	YES	YES
465	a	1366.49	0.00904	YES	YES
466	a	1368.18	2.37042	YES	YES
467	e	1369.20	3.38021	YES	YES
468	e	1369.20	3.38021	YES	YES
469	a	1369.89	2.03737	YES	YES
470	e	1370.96	5.25946	YES	YES
471	e	1370.96	5.25946	YES	YES
472	e	1406.97	3.70140	YES	YES
473	e	1406.97	3.70140	YES	YES
474	a	1407.13	4.36778	YES	YES
475	e	1410.24	0.61270	YES	YES
476	e	1410.24	0.61270	YES	YES
477	a	1410.70	7.71215	YES	YES
478	e	1411.40	4.09421	YES	YES
479	e	1411.40	4.09421	YES	YES

480	e	1412.71	2.71140	YES	YES
481	e	1412.71	2.71140	YES	YES
482	a	1415.38	0.49948	YES	YES
483	e	1416.40	1.30635	YES	YES
484	e	1416.40	1.30635	YES	YES
485	a	1418.42	0.31600	YES	YES
486	a	1419.32	4.61023	YES	YES
487	e	1419.79	10.92753	YES	YES
488	e	1419.79	10.92753	YES	YES
489	e	1420.47	8.03239	YES	YES
490	e	1420.47	8.03239	YES	YES
491	a	1421.22	47.31890	YES	YES
492	e	1421.32	4.84690	YES	YES
493	e	1421.32	4.84690	YES	YES
494	a	1421.35	0.49486	YES	YES
495	a	1422.69	3.20200	YES	YES
496	e	1423.49	19.15681	YES	YES
497	e	1423.49	19.15681	YES	YES
498	a	1424.34	4.73681	YES	YES
499	e	1425.58	3.46823	YES	YES
500	e	1425.58	3.46823	YES	YES
501	e	1426.07	2.45721	YES	YES
502	e	1426.07	2.45721	YES	YES
503	a	1426.11	0.43966	YES	YES
504	a	1426.26	4.89540	YES	YES
505	e	1426.31	0.42453	YES	YES
506	e	1426.31	0.42453	YES	YES
507	a	1427.36	2.49723	YES	YES
508	e	1428.03	5.71352	YES	YES
509	e	1428.03	5.71352	YES	YES
510	a	1428.25	31.78920	YES	YES
511	e	1429.40	2.74226	YES	YES
512	e	1429.40	2.74226	YES	YES
513	a	1430.32	10.23689	YES	YES
514	a	1430.72	1.06464	YES	YES
515	e	1430.97	0.93826	YES	YES
516	e	1430.97	0.93826	YES	YES
517	a	1431.55	33.54265	YES	YES
518	e	1431.66	3.79613	YES	YES
519	e	1431.66	3.79613	YES	YES
520	e	1435.07	27.59414	YES	YES
521	e	1435.07	27.59414	YES	YES
522	a	1435.23	0.49348	YES	YES
523	e	1436.30	15.58313	YES	YES
524	e	1436.30	15.58313	YES	YES
525	a	1436.88	2.05081	YES	YES
526	e	1437.69	2.28968	YES	YES
527	e	1437.69	2.28968	YES	YES
528	a	1437.89	26.38204	YES	YES

529	e	1438.95	1.60666	YES	YES
530	e	1438.95	1.60666	YES	YES
531	a	1439.52	2.10078	YES	YES
532	e	1439.85	5.95038	YES	YES
533	e	1439.85	5.95038	YES	YES
534	a	1440.42	0.14054	YES	YES
535	e	1442.17	24.18241	YES	YES
536	e	1442.17	24.18241	YES	YES
537	a	1442.56	7.63913	YES	YES
538	a	1444.00	0.36497	YES	YES
539	e	1444.15	11.25129	YES	YES
540	e	1444.15	11.25129	YES	YES
541	a	1444.89	4.28405	YES	YES
542	a	1446.20	0.50279	YES	YES
543	e	1446.59	9.09079	YES	YES
544	e	1446.59	9.09079	YES	YES
545	e	1446.86	2.48426	YES	YES
546	e	1446.86	2.48426	YES	YES
547	a	1447.43	3.13048	YES	YES
548	e	1447.86	2.64421	YES	YES
549	e	1447.86	2.64421	YES	YES
550	e	1450.05	12.87987	YES	YES
551	e	1450.05	12.87987	YES	YES
552	a	1451.07	0.01517	YES	YES
553	e	1451.14	4.52186	YES	YES
554	e	1451.14	4.52186	YES	YES
555	a	1451.85	0.02632	YES	YES
556	e	2869.77	13.81004	YES	YES
557	e	2869.77	13.81004	YES	YES
558	e	2870.43	12.93348	YES	YES
559	e	2870.43	12.93348	YES	YES
560	a	2876.18	473.12313	YES	YES
561	a	2876.65	100.93348	YES	YES
562	a	2887.08	0.18599	YES	YES
563	e	2887.23	2.82753	YES	YES
564	e	2887.23	2.82753	YES	YES
565	a	2900.89	0.72869	YES	YES
566	e	2901.01	1.27049	YES	YES
567	e	2901.01	1.27049	YES	YES
568	a	2906.10	185.11357	YES	YES
569	e	2906.46	36.20489	YES	YES
570	e	2906.46	36.20489	YES	YES
571	a	2909.58	149.40967	YES	YES
572	e	2909.97	36.89524	YES	YES
573	e	2909.97	36.89524	YES	YES
574	a	2912.79	11.31932	YES	YES
575	e	2912.85	51.62054	YES	YES
576	e	2912.85	51.62054	YES	YES
577	e	2913.07	30.71668	YES	YES

578	e	2913.07	30.71668	YES	YES
579	a	2913.10	76.12189	YES	YES
580	a	2913.49	55.80560	YES	YES
581	e	2913.53	47.43502	YES	YES
582	e	2913.53	47.43502	YES	YES
583	e	2913.68	31.18798	YES	YES
584	e	2913.68	31.18798	YES	YES
585	e	2913.68	139.31420	YES	YES
586	e	2913.68	139.31420	YES	YES
587	a	2913.69	56.33909	YES	YES
588	a	2913.83	16.92422	YES	YES
589	e	2914.51	109.58964	YES	YES
590	e	2914.51	109.58964	YES	YES
591	a	2914.90	78.34605	YES	YES
592	e	2915.47	85.93600	YES	YES
593	e	2915.47	85.93600	YES	YES
594	a	2915.60	57.94454	YES	YES
595	a	2916.73	36.38186	YES	YES
596	e	2916.76	29.58377	YES	YES
597	e	2916.76	29.58377	YES	YES
598	a	2918.26	7.72383	YES	YES
599	e	2918.47	39.98254	YES	YES
600	e	2918.47	39.98254	YES	YES
601	e	2919.74	19.28536	YES	YES
602	e	2919.74	19.28536	YES	YES
603	a	2919.75	0.32374	YES	YES
604	e	2919.89	37.39708	YES	YES
605	e	2919.89	37.39708	YES	YES
606	a	2919.95	0.59661	YES	YES
607	a	2920.94	62.12631	YES	YES
608	e	2921.27	39.59937	YES	YES
609	e	2921.27	39.59937	YES	YES
610	a	2921.53	42.27283	YES	YES
611	e	2921.54	17.74198	YES	YES
612	e	2921.54	17.74198	YES	YES
613	a	2921.96	96.01626	YES	YES
614	e	2922.07	7.16116	YES	YES
615	e	2922.07	7.16116	YES	YES
616	e	2923.31	80.43128	YES	YES
617	e	2923.31	80.43128	YES	YES
618	a	2923.67	24.62027	YES	YES
619	e	2927.42	108.57378	YES	YES
620	e	2927.42	108.57378	YES	YES
621	a	2927.64	2.75238	YES	YES
622	e	2944.20	55.26290	YES	YES
623	e	2944.20	55.26290	YES	YES
624	a	2944.56	57.28124	YES	YES
625	e	2949.18	54.64688	YES	YES
626	e	2949.18	54.64688	YES	YES

627	a	2949.51	51.08168	YES	YES
628	e	2986.45	9.72719	YES	YES
629	e	2986.45	9.72719	YES	YES
630	a	2986.57	44.71165	YES	YES
631	e	2988.67	18.43484	YES	YES
632	e	2988.67	18.43484	YES	YES
633	a	2988.71	1.52926	YES	YES
634	e	2988.94	35.57519	YES	YES
635	e	2988.94	35.57519	YES	YES
636	a	2989.04	47.85706	YES	YES
637	e	2989.19	1.86499	YES	YES
638	e	2989.19	1.86499	YES	YES
639	a	2989.29	101.45878	YES	YES
640	e	2994.31	5.39455	YES	YES
641	e	2994.31	5.39455	YES	YES
642	a	2994.42	175.66523	YES	YES
643	e	2994.80	19.84089	YES	YES
644	e	2994.80	19.84089	YES	YES
645	a	2994.90	17.00179	YES	YES
646	e	2994.92	0.14797	YES	YES
647	e	2994.92	0.14797	YES	YES
648	a	2995.03	135.02046	YES	YES
649	e	2996.02	0.74529	YES	YES
650	e	2996.02	0.74529	YES	YES
651	a	2996.06	68.32466	YES	YES
652	e	2998.82	43.60400	YES	YES
653	e	2998.82	43.60400	YES	YES
654	a	2998.86	4.39304	YES	YES
655	a	2999.54	9.68393	YES	YES
656	e	2999.55	6.85422	YES	YES
657	e	2999.55	6.85422	YES	YES
658	e	3000.35	0.38502	YES	YES
659	e	3000.35	0.38502	YES	YES
660	a	3000.37	9.37034	YES	YES
661	e	3001.41	70.15250	YES	YES
662	e	3001.41	70.15250	YES	YES
663	a	3001.57	1.23014	YES	YES
664	a	3021.71	40.43483	YES	YES
665	e	3021.73	16.88357	YES	YES
666	e	3021.73	16.88357	YES	YES
667	a	3021.86	14.55395	YES	YES
668	e	3021.88	33.02645	YES	YES
669	e	3021.88	33.02645	YES	YES
670	e	3023.46	9.69175	YES	YES
671	e	3023.46	9.69175	YES	YES
672	a	3023.47	0.09989	YES	YES
673	e	3024.53	34.91951	YES	YES
674	e	3024.53	34.91951	YES	YES
675	a	3024.70	38.42136	YES	YES

676	a	3027.86	7.80735	YES	YES
677	e	3027.89	12.14376	YES	YES
678	e	3027.89	12.14376	YES	YES
679	a	3029.23	0.12994	YES	YES
680	a	3029.89	8.99411	YES	YES
681	e	3030.11	1.36214	YES	YES
682	e	3030.11	1.36214	YES	YES
683	e	3031.19	46.93789	YES	YES
684	e	3031.19	46.93789	YES	YES
685	a	3032.20	13.86866	YES	YES
686	e	3032.72	83.99148	YES	YES
687	e	3032.72	83.99148	YES	YES
688	a	3033.07	0.33502	YES	YES
689	a	3034.38	3.56634	YES	YES
690	e	3035.36	3.03233	YES	YES
691	e	3035.36	3.03233	YES	YES
692	e	3037.64	4.84525	YES	YES
693	e	3037.64	4.84525	YES	YES
694	a	3038.35	0.02895	YES	YES
695	e	3038.40	28.88926	YES	YES
696	e	3038.40	28.88926	YES	YES
697	a	3041.75	0.79124	YES	YES
698	e	3041.79	25.68861	YES	YES
699	e	3041.79	25.68861	YES	YES
700	e	3044.91	8.10763	YES	YES
701	e	3044.91	8.10763	YES	YES
702	a	3044.92	7.30687	YES	YES
703	a	3058.40	5.40363	YES	YES
704	e	3058.42	6.21537	YES	YES
705	e	3058.42	6.21537	YES	YES

6b'

mode	symmetry	wave number	IR intensity	selection rules	
		cm**(-1)	km/mol	IR	RAMAN
1		-0.00	0.00000	-	-
2		-0.00	0.00000	-	-
3		-0.00	0.00000	-	-
4		0.00	0.00000	-	-
5		0.00	0.00000	-	-
6		0.00	0.00000	-	-
7	a	16.14	0.04286	YES	YES
8	a	23.75	0.11910	YES	YES
9	a	25.87	0.02139	YES	YES
10	a	26.81	0.15203	YES	YES
11	a	27.76	0.12931	YES	YES
12	a	29.90	0.12660	YES	YES
13	a	35.02	0.10707	YES	YES
14	a	37.42	0.12639	YES	YES
15	a	39.39	0.03426	YES	YES

16	a	40.64	0.03583	YES	YES
17	a	41.77	0.02768	YES	YES
18	a	43.70	0.01011	YES	YES
19	a	45.20	0.01145	YES	YES
20	a	46.80	0.08130	YES	YES
21	a	50.30	0.13516	YES	YES
22	a	51.66	0.05853	YES	YES
23	a	53.58	0.25959	YES	YES
24	a	54.14	0.16095	YES	YES
25	a	54.55	0.13794	YES	YES
26	a	61.45	0.06287	YES	YES
27	a	62.14	0.10236	YES	YES
28	a	62.92	0.12873	YES	YES
29	a	63.51	0.05409	YES	YES
30	a	69.72	0.59780	YES	YES
31	a	71.05	0.40403	YES	YES
32	a	72.40	0.17938	YES	YES
33	a	73.49	0.21793	YES	YES
34	a	76.15	0.19888	YES	YES
35	a	81.53	0.41339	YES	YES
36	a	81.97	0.06311	YES	YES
37	a	82.12	0.52747	YES	YES
38	a	83.15	0.32265	YES	YES
39	a	87.00	1.39706	YES	YES
40	a	87.66	1.64692	YES	YES
41	a	88.41	1.69736	YES	YES
42	a	89.96	0.82577	YES	YES
43	a	91.25	0.62869	YES	YES
44	a	93.58	0.98240	YES	YES
45	a	96.04	0.17493	YES	YES
46	a	96.89	0.30252	YES	YES
47	a	99.31	0.18107	YES	YES
48	a	100.53	0.10757	YES	YES
49	a	101.20	0.06659	YES	YES
50	a	102.65	0.24436	YES	YES
51	a	103.72	0.51014	YES	YES
52	a	105.60	0.41360	YES	YES
53	a	106.86	0.41234	YES	YES
54	a	112.48	1.80430	YES	YES
55	a	114.86	1.72415	YES	YES
56	a	116.00	0.00617	YES	YES
57	a	116.86	0.37381	YES	YES
58	a	118.74	0.57114	YES	YES
59	a	119.39	0.42492	YES	YES
60	a	123.52	2.42119	YES	YES
61	a	130.04	0.83850	YES	YES
62	a	130.91	0.86426	YES	YES
63	a	133.91	0.50987	YES	YES
64	a	135.15	0.37197	YES	YES

65	a	135.41	0.11768	YES	YES
66	a	138.23	1.54901	YES	YES
67	a	139.76	1.70973	YES	YES
68	a	139.89	1.40263	YES	YES
69	a	146.70	0.00777	YES	YES
70	a	149.11	1.92288	YES	YES
71	a	149.91	2.78864	YES	YES
72	a	150.51	1.33964	YES	YES
73	a	151.46	4.28474	YES	YES
74	a	152.34	3.88134	YES	YES
75	a	154.10	5.70927	YES	YES
76	a	155.02	0.73860	YES	YES
77	a	159.46	0.14577	YES	YES
78	a	161.00	0.29163	YES	YES
79	a	162.46	6.01354	YES	YES
80	a	164.46	1.79853	YES	YES
81	a	166.73	1.80578	YES	YES
82	a	167.60	0.74387	YES	YES
83	a	171.12	0.43077	YES	YES
84	a	171.90	0.01251	YES	YES
85	a	174.51	0.46416	YES	YES
86	a	175.88	2.53599	YES	YES
87	a	178.19	1.06920	YES	YES
88	a	185.86	4.93071	YES	YES
89	a	190.76	5.52276	YES	YES
90	a	191.38	0.48879	YES	YES
91	a	193.29	1.54782	YES	YES
92	a	199.55	0.59931	YES	YES
93	a	200.83	2.14415	YES	YES
94	a	201.65	2.28330	YES	YES
95	a	202.96	2.68900	YES	YES
96	a	204.76	4.14387	YES	YES
97	a	205.50	2.91457	YES	YES
98	a	208.55	5.20010	YES	YES
99	a	210.49	6.16786	YES	YES
100	a	212.29	1.51831	YES	YES
101	a	213.65	3.04057	YES	YES
102	a	218.17	2.44803	YES	YES
103	a	220.23	1.47915	YES	YES
104	a	220.74	3.28660	YES	YES
105	a	221.61	6.16444	YES	YES
106	a	223.99	0.46655	YES	YES
107	a	224.54	2.62634	YES	YES
108	a	224.99	1.07468	YES	YES
109	a	231.72	0.77589	YES	YES
110	a	232.29	1.20912	YES	YES
111	a	234.45	2.54668	YES	YES
112	a	239.50	0.46839	YES	YES
113	a	241.85	0.22801	YES	YES

114	a	243.20	0.30177	YES	YES
115	a	249.06	0.11127	YES	YES
116	a	251.68	0.38174	YES	YES
117	a	254.59	1.63270	YES	YES
118	a	254.84	0.49429	YES	YES
119	a	256.41	0.92672	YES	YES
120	a	256.85	1.28172	YES	YES
121	a	261.59	1.53591	YES	YES
122	a	262.32	0.29795	YES	YES
123	a	262.77	2.37520	YES	YES
124	a	263.83	9.14626	YES	YES
125	a	265.23	12.96000	YES	YES
126	a	265.94	7.12223	YES	YES
127	a	266.85	10.00564	YES	YES
128	a	268.80	1.01127	YES	YES
129	a	269.70	0.52084	YES	YES
130	a	270.37	0.03180	YES	YES
131	a	272.96	1.03811	YES	YES
132	a	273.78	0.30454	YES	YES
133	a	274.34	15.45929	YES	YES
134	a	275.41	6.72262	YES	YES
135	a	275.81	1.24090	YES	YES
136	a	276.25	0.07917	YES	YES
137	a	276.61	0.63458	YES	YES
138	a	277.74	1.38101	YES	YES
139	a	278.41	0.38649	YES	YES
140	a	279.36	0.54552	YES	YES
141	a	280.33	0.04313	YES	YES
142	a	281.45	1.46991	YES	YES
143	a	283.14	1.21561	YES	YES
144	a	283.42	1.02609	YES	YES
145	a	285.06	0.36452	YES	YES
146	a	285.82	0.04807	YES	YES
147	a	286.63	0.07875	YES	YES
148	a	291.00	1.84840	YES	YES
149	a	291.65	0.90776	YES	YES
150	a	291.96	0.96346	YES	YES
151	a	293.31	3.96620	YES	YES
152	a	293.97	4.70271	YES	YES
153	a	294.57	1.93208	YES	YES
154	a	296.65	3.01089	YES	YES
155	a	303.23	0.84873	YES	YES
156	a	311.81	0.43060	YES	YES
157	a	312.67	0.36629	YES	YES
158	a	314.85	0.30148	YES	YES
159	a	315.58	0.45033	YES	YES
160	a	320.70	3.01771	YES	YES
161	a	323.36	2.88119	YES	YES
162	a	329.72	1.55721	YES	YES

163	a	330.49	1.39676	YES	YES
164	a	334.30	0.32699	YES	YES
165	a	334.44	0.28315	YES	YES
166	a	348.09	20.60860	YES	YES
167	a	350.21	4.26402	YES	YES
168	a	362.91	7.51190	YES	YES
169	a	363.59	7.30079	YES	YES
170	a	366.22	14.57635	YES	YES
171	a	366.91	14.03845	YES	YES
172	a	386.63	0.16905	YES	YES
173	a	389.41	0.06569	YES	YES
174	a	389.83	4.45969	YES	YES
175	a	390.35	4.37780	YES	YES
176	a	391.23	1.97937	YES	YES
177	a	391.60	4.08672	YES	YES
178	a	392.10	5.52151	YES	YES
179	a	394.00	0.61498	YES	YES
180	a	403.39	1.03291	YES	YES
181	a	403.72	1.24101	YES	YES
182	a	405.24	2.08326	YES	YES
183	a	405.86	2.14767	YES	YES
184	a	412.46	1.73533	YES	YES
185	a	414.80	0.31554	YES	YES
186	a	415.17	0.28036	YES	YES
187	a	416.22	2.11979	YES	YES
188	a	416.90	0.77823	YES	YES
189	a	417.07	0.74449	YES	YES
190	a	423.13	25.75838	YES	YES
191	a	427.76	32.52460	YES	YES
192	a	437.32	7.78162	YES	YES
193	a	439.42	7.38951	YES	YES
194	a	441.45	6.97357	YES	YES
195	a	442.46	6.72922	YES	YES
196	a	500.93	0.58757	YES	YES
197	a	502.16	16.83667	YES	YES
198	a	502.19	6.37538	YES	YES
199	a	502.50	4.08791	YES	YES
200	a	502.91	33.72476	YES	YES
201	a	503.34	19.96875	YES	YES
202	a	529.10	3.02593	YES	YES
203	a	530.01	2.53531	YES	YES
204	a	530.41	4.30851	YES	YES
205	a	530.50	3.80243	YES	YES
206	a	534.29	15.23457	YES	YES
207	a	536.01	5.52651	YES	YES
208	a	543.33	11.06918	YES	YES
209	a	544.66	10.68857	YES	YES
210	a	546.93	11.34705	YES	YES
211	a	547.39	11.40758	YES	YES

212	a	554.28	88.60134	YES	YES
213	a	554.64	2.03722	YES	YES
214	a	609.57	29.16594	YES	YES
215	a	609.94	26.00852	YES	YES
216	a	610.34	25.27075	YES	YES
217	a	615.19	32.59136	YES	YES
218	a	615.70	27.84956	YES	YES
219	a	616.48	26.86895	YES	YES
220	a	658.69	8.85907	YES	YES
221	a	658.74	8.38223	YES	YES
222	a	659.22	221.93150	YES	YES
223	a	661.08	14.80315	YES	YES
224	a	661.25	14.41972	YES	YES
225	a	662.29	57.00174	YES	YES
226	a	704.79	80.82456	YES	YES
227	a	711.87	15.99207	YES	YES
228	a	726.99	154.94756	YES	YES
229	a	727.48	137.97727	YES	YES
230	a	732.26	751.11708	YES	YES
231	a	732.91	724.77700	YES	YES
232	a	769.22	971.27354	YES	YES
233	a	790.27	13.80372	YES	YES
234	a	880.92	32.93609	YES	YES
235	a	881.49	33.30353	YES	YES
236	a	883.37	58.85162	YES	YES
237	a	883.85	18.90898	YES	YES
238	a	883.99	18.71002	YES	YES
239	a	884.25	49.86683	YES	YES
240	a	884.75	5.46298	YES	YES
241	a	884.96	18.79867	YES	YES
242	a	885.22	5.96578	YES	YES
243	a	885.83	3.98320	YES	YES
244	a	886.42	11.97554	YES	YES
245	a	886.84	13.11356	YES	YES
246	a	887.14	13.70669	YES	YES
247	a	887.60	10.01206	YES	YES
248	a	888.76	6.66440	YES	YES
249	a	889.12	6.15351	YES	YES
250	a	891.92	4.04394	YES	YES
251	a	892.95	4.98310	YES	YES
252	a	893.39	6.01718	YES	YES
253	a	893.57	5.12301	YES	YES
254	a	893.95	12.12765	YES	YES
255	a	895.22	8.73993	YES	YES
256	a	895.73	8.00705	YES	YES
257	a	897.50	7.57730	YES	YES
258	a	897.91	2.73520	YES	YES
259	a	898.06	16.62000	YES	YES
260	a	899.70	6.13481	YES	YES

261	a	900.41	3.05889	YES	YES
262	a	900.91	8.05021	YES	YES
263	a	901.00	0.31501	YES	YES
264	a	901.07	6.80562	YES	YES
265	a	901.18	1.07568	YES	YES
266	a	901.72	2.93681	YES	YES
267	a	901.96	5.54172	YES	YES
268	a	902.57	5.65870	YES	YES
269	a	903.80	4.55462	YES	YES
270	a	903.94	5.96154	YES	YES
271	a	904.83	4.66308	YES	YES
272	a	905.80	2.99601	YES	YES
273	a	908.39	2.76341	YES	YES
274	a	908.62	2.98490	YES	YES
275	a	908.90	2.19500	YES	YES
276	a	920.12	67.47931	YES	YES
277	a	921.02	56.54400	YES	YES
278	a	923.00	124.20144	YES	YES
279	a	923.35	118.00571	YES	YES
280	a	957.44	0.11733	YES	YES
281	a	957.90	0.23741	YES	YES
282	a	957.97	0.90019	YES	YES
283	a	958.27	0.44182	YES	YES
284	a	958.31	0.34132	YES	YES
285	a	958.45	1.79646	YES	YES
286	a	958.56	1.40387	YES	YES
287	a	958.72	1.84788	YES	YES
288	a	959.71	2.18259	YES	YES
289	a	961.83	0.49731	YES	YES
290	a	962.31	0.44433	YES	YES
291	a	962.65	1.07223	YES	YES
292	a	963.42	0.42518	YES	YES
293	a	964.11	1.96026	YES	YES
294	a	964.46	0.04572	YES	YES
295	a	964.60	1.44345	YES	YES
296	a	964.77	0.26206	YES	YES
297	a	966.50	1.04894	YES	YES
298	a	976.11	8.17861	YES	YES
299	a	976.89	11.48647	YES	YES
300	a	977.16	9.52716	YES	YES
301	a	978.17	4.36701	YES	YES
302	a	978.43	3.27835	YES	YES
303	a	979.47	1.25195	YES	YES
304	a	981.28	16.45271	YES	YES
305	a	982.16	15.66991	YES	YES
306	a	982.59	9.07983	YES	YES
307	a	983.06	2.99965	YES	YES
308	a	984.87	24.92531	YES	YES
309	a	984.93	24.99267	YES	YES

310	a	1000.63	0.15328	YES	YES
311	a	1006.21	4.85912	YES	YES
312	a	1006.69	19.92322	YES	YES
313	a	1007.06	13.74531	YES	YES
314	a	1007.99	20.30033	YES	YES
315	a	1009.16	20.39717	YES	YES
316	a	1009.38	30.68741	YES	YES
317	a	1011.25	21.33674	YES	YES
318	a	1021.74	5.49486	YES	YES
319	a	1022.01	23.95480	YES	YES
320	a	1022.31	4.89528	YES	YES
321	a	1022.53	29.41546	YES	YES
322	a	1049.15	6.74039	YES	YES
323	a	1052.03	7.18751	YES	YES
324	a	1060.32	182.00194	YES	YES
325	a	1060.60	24.56945	YES	YES
326	a	1060.82	35.80904	YES	YES
327	a	1061.84	108.21897	YES	YES
328	a	1062.25	26.24960	YES	YES
329	a	1062.73	25.48192	YES	YES
330	a	1081.37	1.36567	YES	YES
331	a	1081.56	7.92001	YES	YES
332	a	1082.04	15.89022	YES	YES
333	a	1083.07	1.10685	YES	YES
334	a	1083.23	2.88417	YES	YES
335	a	1083.52	3.42309	YES	YES
336	a	1091.47	4.96977	YES	YES
337	a	1091.50	5.76473	YES	YES
338	a	1091.87	3.30578	YES	YES
339	a	1091.93	3.86863	YES	YES
340	a	1092.56	3.62863	YES	YES
341	a	1093.03	3.89903	YES	YES
342	a	1100.62	2.32704	YES	YES
343	a	1100.81	2.22764	YES	YES
344	a	1101.07	9.22067	YES	YES
345	a	1101.30	1.99057	YES	YES
346	a	1101.53	2.00015	YES	YES
347	a	1101.82	3.42986	YES	YES
348	a	1137.62	5.46493	YES	YES
349	a	1138.03	1.37114	YES	YES
350	a	1138.08	13.33756	YES	YES
351	a	1138.43	9.44629	YES	YES
352	a	1148.02	0.11534	YES	YES
353	a	1148.22	0.09173	YES	YES
354	a	1148.38	0.15713	YES	YES
355	a	1149.12	0.28624	YES	YES
356	a	1149.55	0.87870	YES	YES
357	a	1149.75	0.74520	YES	YES
358	a	1149.79	1.14167	YES	YES

359	a	1150.16	0.12752	YES	YES
360	a	1150.35	0.01725	YES	YES
361	a	1151.77	0.32160	YES	YES
362	a	1152.15	0.48000	YES	YES
363	a	1152.17	0.30234	YES	YES
364	a	1152.36	0.27166	YES	YES
365	a	1152.47	0.10369	YES	YES
366	a	1152.75	0.12191	YES	YES
367	a	1154.12	0.21334	YES	YES
368	a	1154.47	0.19711	YES	YES
369	a	1154.99	0.19371	YES	YES
370	a	1203.75	0.01079	YES	YES
371	a	1205.33	0.09331	YES	YES
372	a	1210.43	2.18317	YES	YES
373	a	1210.81	2.99302	YES	YES
374	a	1211.28	3.46253	YES	YES
375	a	1214.81	0.12867	YES	YES
376	a	1214.94	1.58459	YES	YES
377	a	1215.55	1.36735	YES	YES
378	a	1216.31	2.36695	YES	YES
379	a	1217.32	1.76098	YES	YES
380	a	1218.28	1.21329	YES	YES
381	a	1219.26	3.09228	YES	YES
382	a	1219.69	2.41987	YES	YES
383	a	1219.72	2.15517	YES	YES
384	a	1228.98	4.88284	YES	YES
385	a	1229.34	4.65602	YES	YES
386	a	1230.66	4.58688	YES	YES
387	a	1231.12	5.09522	YES	YES
388	a	1236.21	9.86702	YES	YES
389	a	1237.00	20.48771	YES	YES
390	a	1238.14	19.25652	YES	YES
391	a	1242.76	8.79550	YES	YES
392	a	1243.07	12.96205	YES	YES
393	a	1244.76	11.20380	YES	YES
394	a	1253.79	10.83147	YES	YES
395	a	1257.91	16.45528	YES	YES
396	a	1259.99	10.08918	YES	YES
397	a	1260.47	10.48567	YES	YES
398	a	1261.00	13.71281	YES	YES
399	a	1261.38	13.39170	YES	YES
400	a	1284.67	3.74679	YES	YES
401	a	1284.83	6.51318	YES	YES
402	a	1285.22	3.01014	YES	YES
403	a	1286.02	0.43157	YES	YES
404	a	1286.17	1.39744	YES	YES
405	a	1286.26	1.30765	YES	YES
406	a	1288.15	0.64204	YES	YES
407	a	1288.21	0.15138	YES	YES

408	a	1288.42	0.85895	YES	YES
409	a	1288.95	3.93334	YES	YES
410	a	1289.04	2.49585	YES	YES
411	a	1289.16	5.76023	YES	YES
412	a	1289.26	1.93290	YES	YES
413	a	1289.42	0.47523	YES	YES
414	a	1289.79	0.56384	YES	YES
415	a	1290.25	2.64869	YES	YES
416	a	1291.27	6.47971	YES	YES
417	a	1292.31	3.84025	YES	YES
418	a	1311.54	1.78118	YES	YES
419	a	1312.95	0.61995	YES	YES
420	a	1314.82	0.59907	YES	YES
421	a	1315.70	0.50303	YES	YES
422	a	1319.62	78.37930	YES	YES
423	a	1322.84	59.92541	YES	YES
424	a	1340.25	0.17612	YES	YES
425	a	1340.43	0.04415	YES	YES
426	a	1340.64	0.10766	YES	YES
427	a	1340.96	0.06603	YES	YES
428	a	1341.13	0.40123	YES	YES
429	a	1341.61	0.01637	YES	YES
430	a	1344.21	3.23225	YES	YES
431	a	1344.45	1.96637	YES	YES
432	a	1344.56	3.96724	YES	YES
433	a	1345.88	2.07633	YES	YES
434	a	1346.18	2.40764	YES	YES
435	a	1346.80	3.40192	YES	YES
436	a	1347.83	14.49978	YES	YES
437	a	1348.66	9.79158	YES	YES
438	a	1349.19	2.04766	YES	YES
439	a	1349.28	1.53623	YES	YES
440	a	1349.53	2.53796	YES	YES
441	a	1349.58	1.53052	YES	YES
442	a	1349.98	0.54187	YES	YES
443	a	1350.58	3.14140	YES	YES
444	a	1350.90	0.72201	YES	YES
445	a	1351.76	1.51468	YES	YES
446	a	1354.46	2.52440	YES	YES
447	a	1355.13	2.62284	YES	YES
448	a	1359.99	0.83149	YES	YES
449	a	1360.04	0.91215	YES	YES
450	a	1360.39	1.31526	YES	YES
451	a	1360.82	2.50114	YES	YES
452	a	1360.88	1.11217	YES	YES
453	a	1361.28	1.11887	YES	YES
454	a	1364.29	3.73609	YES	YES
455	a	1364.42	5.32765	YES	YES
456	a	1365.63	1.67378	YES	YES

457	a	1365.90	1.34935	YES	YES
458	a	1366.99	0.06276	YES	YES
459	a	1367.39	0.02340	YES	YES
460	a	1369.89	7.83990	YES	YES
461	a	1370.92	4.31849	YES	YES
462	a	1371.56	4.15952	YES	YES
463	a	1372.15	0.10181	YES	YES
464	a	1373.24	3.56315	YES	YES
465	a	1373.95	3.88384	YES	YES
466	a	1404.26	0.54853	YES	YES
467	a	1404.99	0.30140	YES	YES
468	a	1406.04	0.08068	YES	YES
469	a	1406.45	1.35384	YES	YES
470	a	1407.56	0.75006	YES	YES
471	a	1409.77	0.57550	YES	YES
472	a	1412.92	4.92192	YES	YES
473	a	1413.10	0.50940	YES	YES
474	a	1414.19	3.42276	YES	YES
475	a	1414.93	0.85695	YES	YES
476	a	1417.44	6.79090	YES	YES
477	a	1417.97	4.09904	YES	YES
478	a	1418.24	1.05022	YES	YES
479	a	1418.28	3.35986	YES	YES
480	a	1419.48	0.48927	YES	YES
481	a	1419.64	1.94949	YES	YES
482	a	1419.89	3.42911	YES	YES
483	a	1420.02	13.36438	YES	YES
484	a	1420.67	4.45555	YES	YES
485	a	1421.36	1.86926	YES	YES
486	a	1421.62	1.10088	YES	YES
487	a	1422.73	3.36534	YES	YES
488	a	1423.05	4.27026	YES	YES
489	a	1423.24	3.39069	YES	YES
490	a	1424.97	2.86259	YES	YES
491	a	1425.27	0.39021	YES	YES
492	a	1425.64	5.42244	YES	YES
493	a	1425.89	4.75873	YES	YES
494	a	1425.91	5.51712	YES	YES
495	a	1426.17	3.69856	YES	YES
496	a	1427.17	1.08736	YES	YES
497	a	1427.30	2.16443	YES	YES
498	a	1427.53	3.24425	YES	YES
499	a	1427.61	2.18350	YES	YES
500	a	1427.79	3.70350	YES	YES
501	a	1428.64	9.51094	YES	YES
502	a	1428.95	2.97623	YES	YES
503	a	1429.03	0.93141	YES	YES
504	a	1429.44	4.98901	YES	YES
505	a	1431.34	1.07967	YES	YES

506	a	1431.44	0.98995	YES	YES
507	a	1431.99	39.62395	YES	YES
508	a	1432.42	2.89353	YES	YES
509	a	1432.66	1.98672	YES	YES
510	a	1432.80	3.39236	YES	YES
511	a	1434.27	15.70228	YES	YES
512	a	1434.32	16.03520	YES	YES
513	a	1435.17	3.37929	YES	YES
514	a	1435.28	6.68821	YES	YES
515	a	1435.63	33.95705	YES	YES
516	a	1436.69	2.13116	YES	YES
517	a	1437.60	0.98859	YES	YES
518	a	1437.91	4.30998	YES	YES
519	a	1438.03	5.09400	YES	YES
520	a	1438.65	3.06833	YES	YES
521	a	1438.92	0.46392	YES	YES
522	a	1439.15	1.05840	YES	YES
523	a	1440.18	0.24350	YES	YES
524	a	1440.87	3.93200	YES	YES
525	a	1440.93	2.45873	YES	YES
526	a	1441.67	8.03495	YES	YES
527	a	1441.80	9.10186	YES	YES
528	a	1442.01	9.26563	YES	YES
529	a	1442.97	2.34436	YES	YES
530	a	1443.31	0.28039	YES	YES
531	a	1443.44	0.89958	YES	YES
532	a	1443.83	0.68329	YES	YES
533	a	1444.75	21.17110	YES	YES
534	a	1444.98	20.12024	YES	YES
535	a	1446.62	3.46619	YES	YES
536	a	1447.19	4.31187	YES	YES
537	a	1447.41	4.03256	YES	YES
538	a	1448.02	0.19190	YES	YES
539	a	1448.61	0.63753	YES	YES
540	a	1448.88	0.25602	YES	YES
541	a	1449.85	0.62287	YES	YES
542	a	1450.24	0.63524	YES	YES
543	a	1450.40	2.16722	YES	YES
544	a	1450.83	2.55335	YES	YES
545	a	1450.95	2.47548	YES	YES
546	a	1451.69	0.98794	YES	YES
547	a	1452.62	16.51278	YES	YES
548	a	1452.81	14.99136	YES	YES
549	a	1453.87	1.99346	YES	YES
550	a	2864.74	6.63532	YES	YES
551	a	2864.83	6.48765	YES	YES
552	a	2867.56	7.56594	YES	YES
553	a	2867.72	7.39083	YES	YES
554	a	2871.55	270.74477	YES	YES

555	a	2873.94	220.42176	YES	YES
556	a	2875.84	7.35788	YES	YES
557	a	2875.96	4.92356	YES	YES
558	a	2877.34	5.78611	YES	YES
559	a	2890.86	110.92242	YES	YES
560	a	2891.25	142.67619	YES	YES
561	a	2891.98	157.79320	YES	YES
562	a	2894.68	66.89420	YES	YES
563	a	2894.96	128.23698	YES	YES
564	a	2896.06	127.61573	YES	YES
565	a	2904.23	3.39610	YES	YES
566	a	2905.15	3.29702	YES	YES
567	a	2905.74	2.78378	YES	YES
568	a	2910.39	75.13948	YES	YES
569	a	2910.53	62.45031	YES	YES
570	a	2910.92	38.41310	YES	YES
571	a	2912.82	42.57292	YES	YES
572	a	2912.96	57.97435	YES	YES
573	a	2913.05	60.25712	YES	YES
574	a	2913.32	69.10413	YES	YES
575	a	2913.40	58.93114	YES	YES
576	a	2913.48	17.05737	YES	YES
577	a	2913.53	84.30527	YES	YES
578	a	2913.66	47.62840	YES	YES
579	a	2913.72	35.76681	YES	YES
580	a	2914.03	103.67363	YES	YES
581	a	2914.24	90.33626	YES	YES
582	a	2914.47	106.00581	YES	YES
583	a	2914.79	83.77054	YES	YES
584	a	2914.90	76.76072	YES	YES
585	a	2915.15	53.66509	YES	YES
586	a	2916.83	40.98885	YES	YES
587	a	2917.28	41.84587	YES	YES
588	a	2917.39	47.48347	YES	YES
589	a	2917.84	135.15395	YES	YES
590	a	2918.00	24.25874	YES	YES
591	a	2918.03	23.59519	YES	YES
592	a	2918.67	31.75147	YES	YES
593	a	2918.73	32.54674	YES	YES
594	a	2919.11	27.58841	YES	YES
595	a	2921.51	25.78213	YES	YES
596	a	2922.00	10.85109	YES	YES
597	a	2922.08	19.77988	YES	YES
598	a	2922.29	14.08738	YES	YES
599	a	2922.40	32.19135	YES	YES
600	a	2922.82	27.35355	YES	YES
601	a	2924.40	25.27776	YES	YES
602	a	2924.47	12.15568	YES	YES
603	a	2924.81	11.52567	YES	YES

604	a	2924.90	24.32860	YES	YES
605	a	2925.30	4.25058	YES	YES
606	a	2925.33	21.00633	YES	YES
607	a	2925.59	42.59624	YES	YES
608	a	2926.30	12.49392	YES	YES
609	a	2926.36	14.97609	YES	YES
610	a	2927.01	42.49603	YES	YES
611	a	2927.16	33.32779	YES	YES
612	a	2927.28	42.31713	YES	YES
613	a	2929.10	101.45069	YES	YES
614	a	2929.11	27.87251	YES	YES
615	a	2929.22	60.52800	YES	YES
616	a	2929.29	76.72191	YES	YES
617	a	2929.76	15.24547	YES	YES
618	a	2929.88	101.26472	YES	YES
619	a	2930.21	193.76141	YES	YES
620	a	2930.28	165.25609	YES	YES
621	a	2930.89	19.36561	YES	YES
622	a	2987.31	24.12236	YES	YES
623	a	2987.47	25.24674	YES	YES
624	a	2987.51	15.45525	YES	YES
625	a	2987.53	33.06207	YES	YES
626	a	2987.63	21.78300	YES	YES
627	a	2988.13	22.39274	YES	YES
628	a	2991.24	40.66730	YES	YES
629	a	2991.45	28.74523	YES	YES
630	a	2991.55	122.51182	YES	YES
631	a	2993.69	73.26315	YES	YES
632	a	2993.77	43.90415	YES	YES
633	a	2993.98	64.59767	YES	YES
634	a	2994.01	24.38609	YES	YES
635	a	2994.11	114.38370	YES	YES
636	a	2994.19	6.66133	YES	YES
637	a	2994.93	9.95800	YES	YES
638	a	2995.08	10.53339	YES	YES
639	a	2995.57	29.97195	YES	YES
640	a	2995.70	16.08124	YES	YES
641	a	2995.78	18.92518	YES	YES
642	a	2995.91	89.83940	YES	YES
643	a	2996.93	13.46038	YES	YES
644	a	2996.99	13.15938	YES	YES
645	a	2997.15	13.04843	YES	YES
646	a	3001.05	4.35968	YES	YES
647	a	3001.14	4.56762	YES	YES
648	a	3001.44	4.47254	YES	YES
649	a	3002.04	15.14871	YES	YES
650	a	3002.28	3.45014	YES	YES
651	a	3002.71	15.31671	YES	YES
652	a	3002.95	25.89469	YES	YES

653	a	3004.15	16.58276	YES	YES
654	a	3004.74	18.83131	YES	YES
655	a	3019.26	23.86541	YES	YES
656	a	3019.36	25.34709	YES	YES
657	a	3019.41	27.67971	YES	YES
658	a	3020.66	1.79662	YES	YES
659	a	3021.39	12.67708	YES	YES
660	a	3021.57	2.83936	YES	YES
661	a	3022.23	14.20071	YES	YES
662	a	3022.36	19.36589	YES	YES
663	a	3023.19	5.67068	YES	YES
664	a	3023.35	7.50464	YES	YES
665	a	3023.56	7.24283	YES	YES
666	a	3023.91	40.25259	YES	YES
667	a	3024.26	40.81022	YES	YES
668	a	3024.54	25.60582	YES	YES
669	a	3024.84	17.51111	YES	YES
670	a	3025.87	36.32320	YES	YES
671	a	3026.12	41.19717	YES	YES
672	a	3027.28	25.72835	YES	YES
673	a	3027.45	14.48171	YES	YES
674	a	3027.70	22.80168	YES	YES
675	a	3027.78	22.53929	YES	YES
676	a	3028.35	6.22976	YES	YES
677	a	3028.40	4.72862	YES	YES
678	a	3028.79	9.62439	YES	YES
679	a	3029.04	5.34295	YES	YES
680	a	3029.19	4.50644	YES	YES
681	a	3029.28	7.41052	YES	YES
682	a	3035.07	8.31894	YES	YES
683	a	3035.33	4.00964	YES	YES
684	a	3035.56	15.76628	YES	YES
685	a	3035.63	21.14778	YES	YES
686	a	3035.68	6.40292	YES	YES
687	a	3035.81	35.10798	YES	YES
688	a	3035.83	11.11764	YES	YES
689	a	3036.09	21.55611	YES	YES
690	a	3036.20	25.51236	YES	YES
691	a	3037.86	12.03487	YES	YES
692	a	3038.32	11.73428	YES	YES
693	a	3038.84	12.31042	YES	YES
694	a	3058.49	4.06744	YES	YES
695	a	3059.03	4.20529	YES	YES
696	a	3059.71	4.10291	YES	YES
697	a	3077.59	3.27636	YES	YES
698	a	3077.72	3.55273	YES	YES
699	a	3078.02	3.52188	YES	YES

mode	symmetry	wave number	IR intensity	selection rules	
		cm**(-1)	km/mol	IR	RAMAN
1		-0.00	0.00000	-	-
2		-0.00	0.00000	-	-
3		0.00	0.00000	-	-
4		0.00	0.00000	-	-
5		0.00	0.00000	-	-
6		0.00	0.00000	-	-
7	a	7.32	0.07252	YES	YES
8	a	10.72	0.06592	YES	YES
9	a	14.29	0.05591	YES	YES
10	a	23.29	0.06326	YES	YES
11	a	24.29	0.04379	YES	YES
12	a	25.67	0.06657	YES	YES
13	a	28.64	0.02640	YES	YES
14	a	30.67	0.05386	YES	YES
15	a	34.43	0.20015	YES	YES
16	a	35.21	0.05587	YES	YES
17	a	38.41	0.03002	YES	YES
18	a	39.69	0.02746	YES	YES
19	a	41.51	0.02489	YES	YES
20	a	41.75	0.09116	YES	YES
21	a	43.18	0.05955	YES	YES
22	a	45.16	0.00819	YES	YES
23	a	45.64	0.06160	YES	YES
24	a	46.80	0.02762	YES	YES
25	a	53.05	0.17419	YES	YES
26	a	54.21	0.07067	YES	YES
27	a	56.41	0.03426	YES	YES
28	a	59.28	0.17764	YES	YES
29	a	61.90	0.08951	YES	YES
30	a	62.89	0.18188	YES	YES
31	a	63.85	0.25909	YES	YES
32	a	68.17	0.01227	YES	YES
33	a	68.35	0.20072	YES	YES
34	a	70.38	0.07495	YES	YES
35	a	73.58	0.06169	YES	YES
36	a	74.31	0.08364	YES	YES
37	a	78.13	0.19684	YES	YES
38	a	80.00	0.29088	YES	YES
39	a	80.15	0.75869	YES	YES
40	a	82.60	0.39912	YES	YES
41	a	85.77	0.19634	YES	YES
42	a	88.58	1.22747	YES	YES
43	a	92.69	0.73619	YES	YES
44	a	93.41	1.30838	YES	YES
45	a	95.28	1.64247	YES	YES
46	a	96.89	2.90915	YES	YES
47	a	98.72	0.84978	YES	YES

48	a	102.09	1.12905	YES	YES
49	a	104.72	0.18619	YES	YES
50	a	106.78	0.48437	YES	YES
51	a	108.09	0.27204	YES	YES
52	a	109.39	1.12654	YES	YES
53	a	109.93	1.54210	YES	YES
54	a	112.36	1.18675	YES	YES
55	a	127.41	2.19599	YES	YES
56	a	131.11	3.28094	YES	YES
57	a	136.13	0.45623	YES	YES
58	a	137.34	1.39020	YES	YES
59	a	140.31	0.11089	YES	YES
60	a	141.45	0.22357	YES	YES
61	a	142.40	1.13163	YES	YES
62	a	148.63	1.91989	YES	YES
63	a	150.00	1.70952	YES	YES
64	a	168.38	30.33363	YES	YES
65	a	170.65	3.18308	YES	YES
66	a	181.07	5.96017	YES	YES
67	a	183.71	4.17023	YES	YES
68	a	184.23	6.80703	YES	YES
69	a	185.91	3.08813	YES	YES
70	a	187.02	5.61872	YES	YES
71	a	188.23	1.91475	YES	YES
72	a	194.15	0.90128	YES	YES
73	a	194.34	1.90962	YES	YES
74	a	197.81	0.21696	YES	YES
75	a	202.91	1.74884	YES	YES
76	a	207.66	2.03758	YES	YES
77	a	210.73	0.21572	YES	YES
78	a	212.51	0.77269	YES	YES
79	a	214.13	1.56057	YES	YES
80	a	216.07	0.10335	YES	YES
81	a	218.26	0.35473	YES	YES
82	a	219.18	0.32235	YES	YES
83	a	219.73	0.89677	YES	YES
84	a	226.50	0.16269	YES	YES
85	a	227.91	0.74905	YES	YES
86	a	230.22	3.34961	YES	YES
87	a	233.16	1.54351	YES	YES
88	a	234.58	2.31813	YES	YES
89	a	238.96	1.08128	YES	YES
90	a	239.25	1.86753	YES	YES
91	a	242.33	6.04896	YES	YES
92	a	244.56	0.94394	YES	YES
93	a	245.39	1.82940	YES	YES
94	a	248.38	2.38302	YES	YES
95	a	249.15	0.41567	YES	YES
96	a	252.53	2.71392	YES	YES

97	a	253.62	5.04271	YES	YES
98	a	257.34	2.33152	YES	YES
99	a	258.17	1.07294	YES	YES
100	a	259.62	2.70240	YES	YES
101	a	259.79	0.34500	YES	YES
102	a	262.83	1.79953	YES	YES
103	a	263.10	1.81356	YES	YES
104	a	268.33	6.17853	YES	YES
105	a	268.64	4.88119	YES	YES
106	a	277.62	4.72092	YES	YES
107	a	279.61	8.36962	YES	YES
108	a	280.26	1.98287	YES	YES
109	a	283.27	0.91714	YES	YES
110	a	285.88	10.24482	YES	YES
111	a	287.50	8.25681	YES	YES
112	a	306.63	4.86740	YES	YES
113	a	319.94	1.18019	YES	YES
114	a	322.06	3.96102	YES	YES
115	a	322.29	1.64697	YES	YES
116	a	325.32	1.34478	YES	YES
117	a	326.15	8.38267	YES	YES
118	a	329.26	2.14327	YES	YES
119	a	331.92	5.46720	YES	YES
120	a	336.18	5.30842	YES	YES
121	a	337.07	4.72299	YES	YES
122	a	347.36	0.67852	YES	YES
123	a	351.03	0.94063	YES	YES
124	a	357.47	0.64688	YES	YES
125	a	360.66	0.69236	YES	YES
126	a	362.53	0.94009	YES	YES
127	a	363.71	1.35806	YES	YES
128	a	378.88	0.85059	YES	YES
129	a	382.47	0.81596	YES	YES
130	a	392.48	2.80591	YES	YES
131	a	397.78	0.68954	YES	YES
132	a	401.28	4.14960	YES	YES
133	a	408.28	3.23469	YES	YES
134	a	415.45	1.40376	YES	YES
135	a	418.67	2.28772	YES	YES
136	a	419.87	3.37693	YES	YES
137	a	421.84	0.92013	YES	YES
138	a	427.86	5.22772	YES	YES
139	a	430.53	11.65230	YES	YES
140	a	433.17	0.81187	YES	YES
141	a	435.47	0.94911	YES	YES
142	a	441.81	10.29293	YES	YES
143	a	442.62	4.76926	YES	YES
144	a	446.71	11.99960	YES	YES
145	a	448.50	5.65037	YES	YES

146	a	452.26	9.09095	YES	YES
147	a	454.65	10.89528	YES	YES
148	a	508.75	24.94851	YES	YES
149	a	510.26	37.97968	YES	YES
150	a	512.54	28.48442	YES	YES
151	a	522.19	25.52699	YES	YES
152	a	524.00	141.44671	YES	YES
153	a	527.24	20.82352	YES	YES
154	a	563.62	5.40154	YES	YES
155	a	565.49	5.29859	YES	YES
156	a	566.54	9.85810	YES	YES
157	a	569.56	6.13702	YES	YES
158	a	578.94	6.60863	YES	YES
159	a	581.15	5.79974	YES	YES
160	a	593.16	23.00273	YES	YES
161	a	596.54	11.24915	YES	YES
162	a	598.13	52.36582	YES	YES
163	a	598.89	3.92362	YES	YES
164	a	611.14	18.04454	YES	YES
165	a	615.97	0.67729	YES	YES
166	a	618.63	56.56954	YES	YES
167	a	623.06	67.16392	YES	YES
168	a	632.79	39.64143	YES	YES
169	a	635.85	41.06243	YES	YES
170	a	669.00	157.68956	YES	YES
171	a	673.05	2.84648	YES	YES
172	a	739.99	27.99264	YES	YES
173	a	740.45	75.08286	YES	YES
174	a	741.87	91.38542	YES	YES
175	a	744.50	73.59331	YES	YES
176	a	802.99	0.65692	YES	YES
177	a	804.30	1.53318	YES	YES
178	a	839.99	24.19776	YES	YES
179	a	841.73	22.59949	YES	YES
180	a	842.81	21.44981	YES	YES
181	a	847.42	18.88585	YES	YES
182	a	879.66	0.03479	YES	YES
183	a	881.04	2.03471	YES	YES
184	a	882.67	1.07263	YES	YES
185	a	883.51	1.32127	YES	YES
186	a	883.68	8.16913	YES	YES
187	a	884.02	9.30331	YES	YES
188	a	885.40	2.07096	YES	YES
189	a	885.72	5.84860	YES	YES
190	a	887.09	12.53763	YES	YES
191	a	887.13	5.71774	YES	YES
192	a	888.60	7.49288	YES	YES
193	a	889.89	10.71238	YES	YES
194	a	900.79	2.22576	YES	YES

195	a	902.89	7.53764	YES	YES
196	a	903.64	7.37590	YES	YES
197	a	905.06	1.19244	YES	YES
198	a	905.37	1.72613	YES	YES
199	a	905.54	1.97259	YES	YES
200	a	906.58	1.21185	YES	YES
201	a	906.79	0.58465	YES	YES
202	a	907.44	4.12612	YES	YES
203	a	907.70	1.84931	YES	YES
204	a	907.72	1.94032	YES	YES
205	a	910.89	2.01367	YES	YES
206	a	911.52	5.06369	YES	YES
207	a	911.93	2.17474	YES	YES
208	a	913.84	2.24482	YES	YES
209	a	914.53	2.82104	YES	YES
210	a	944.85	0.29914	YES	YES
211	a	945.06	0.16623	YES	YES
212	a	945.96	0.20877	YES	YES
213	a	946.10	0.65895	YES	YES
214	a	947.11	0.28348	YES	YES
215	a	948.57	0.54673	YES	YES
216	a	948.63	0.43925	YES	YES
217	a	948.82	0.45056	YES	YES
218	a	948.88	0.27465	YES	YES
219	a	949.22	0.34450	YES	YES
220	a	949.36	0.59937	YES	YES
221	a	949.85	0.14173	YES	YES
222	a	975.99	0.84865	YES	YES
223	a	977.37	1.74434	YES	YES
224	a	983.91	6.54919	YES	YES
225	a	990.23	15.29874	YES	YES
226	a	990.86	2.67048	YES	YES
227	a	992.14	13.50104	YES	YES
228	a	992.24	9.39361	YES	YES
229	a	993.47	16.17634	YES	YES
230	a	1004.55	4.18252	YES	YES
231	a	1008.89	3.62315	YES	YES
232	a	1011.23	7.67719	YES	YES
233	a	1012.12	1.04633	YES	YES
234	a	1017.57	18.33014	YES	YES
235	a	1022.11	21.72806	YES	YES
236	a	1024.23	0.05167	YES	YES
237	a	1026.40	0.30651	YES	YES
238	a	1034.76	1.84174	YES	YES
239	a	1035.73	4.79662	YES	YES
240	a	1040.63	1.32918	YES	YES
241	a	1041.36	0.91430	YES	YES
242	a	1041.74	0.37893	YES	YES
243	a	1042.64	0.35854	YES	YES

244	a	1091.84	0.51348	YES	YES
245	a	1093.54	1.42355	YES	YES
246	a	1094.06	0.65611	YES	YES
247	a	1095.08	3.39516	YES	YES
248	a	1095.34	0.95558	YES	YES
249	a	1096.65	1.80047	YES	YES
250	a	1098.85	3.72523	YES	YES
251	a	1101.61	4.06890	YES	YES
252	a	1104.85	3.53467	YES	YES
253	a	1107.81	7.32427	YES	YES
254	a	1108.82	6.68238	YES	YES
255	a	1109.52	4.10906	YES	YES
256	a	1110.02	1.91580	YES	YES
257	a	1110.22	13.90674	YES	YES
258	a	1112.04	21.56066	YES	YES
259	a	1113.30	37.95871	YES	YES
260	a	1117.16	46.39968	YES	YES
261	a	1120.78	46.45005	YES	YES
262	a	1145.84	0.37939	YES	YES
263	a	1147.49	1.80806	YES	YES
264	a	1148.00	0.64461	YES	YES
265	a	1149.90	1.33161	YES	YES
266	a	1151.56	4.43155	YES	YES
267	a	1151.59	0.10503	YES	YES
268	a	1151.91	1.41757	YES	YES
269	a	1153.23	5.51630	YES	YES
270	a	1153.60	5.73337	YES	YES
271	a	1155.69	0.48538	YES	YES
272	a	1156.35	10.90373	YES	YES
273	a	1157.58	3.83079	YES	YES
274	a	1172.15	509.83183	YES	YES
275	a	1174.16	156.48533	YES	YES
276	a	1200.16	12.67298	YES	YES
277	a	1205.34	16.81858	YES	YES
278	a	1205.71	18.99856	YES	YES
279	a	1206.69	26.83168	YES	YES
280	a	1207.54	31.44153	YES	YES
281	a	1208.20	22.18275	YES	YES
282	a	1219.13	6.18105	YES	YES
283	a	1220.36	0.15881	YES	YES
284	a	1221.40	8.07171	YES	YES
285	a	1221.88	1.64075	YES	YES
286	a	1223.03	6.99060	YES	YES
287	a	1224.05	1.78335	YES	YES
288	a	1225.21	5.26438	YES	YES
289	a	1225.44	3.65469	YES	YES
290	a	1226.99	6.38629	YES	YES
291	a	1227.41	1.18542	YES	YES
292	a	1228.00	3.56444	YES	YES

293	a	1231.77	7.08778	YES	YES
294	a	1255.81	10.74453	YES	YES
295	a	1258.57	8.54656	YES	YES
296	a	1260.20	11.13162	YES	YES
297	a	1260.26	11.07731	YES	YES
298	a	1281.56	0.33290	YES	YES
299	a	1281.80	0.57872	YES	YES
300	a	1282.14	0.25998	YES	YES
301	a	1283.10	0.03266	YES	YES
302	a	1286.15	0.05211	YES	YES
303	a	1286.21	0.53095	YES	YES
304	a	1287.89	0.01104	YES	YES
305	a	1288.74	0.17178	YES	YES
306	a	1288.93	0.32287	YES	YES
307	a	1292.05	0.22641	YES	YES
308	a	1292.94	2.48239	YES	YES
309	a	1293.37	0.86295	YES	YES
310	a	1304.25	0.06826	YES	YES
311	a	1305.64	5.91740	YES	YES
312	a	1312.20	155.79505	YES	YES
313	a	1312.59	0.36839	YES	YES
314	a	1322.33	1.56906	YES	YES
315	a	1322.66	1.53135	YES	YES
316	a	1323.24	1.28176	YES	YES
317	a	1324.10	1.67906	YES	YES
318	a	1331.44	0.05970	YES	YES
319	a	1332.15	0.10286	YES	YES
320	a	1332.62	0.12636	YES	YES
321	a	1333.92	0.10764	YES	YES
322	a	1340.28	3.11323	YES	YES
323	a	1341.37	1.93844	YES	YES
324	a	1342.21	0.73507	YES	YES
325	a	1342.62	2.73742	YES	YES
326	a	1342.80	3.68860	YES	YES
327	a	1343.46	2.86803	YES	YES
328	a	1343.61	4.19182	YES	YES
329	a	1344.31	2.18845	YES	YES
330	a	1344.54	0.67608	YES	YES
331	a	1345.85	1.17593	YES	YES
332	a	1346.80	1.53617	YES	YES
333	a	1347.08	4.23790	YES	YES
334	a	1356.06	1.81768	YES	YES
335	a	1357.07	1.22786	YES	YES
336	a	1358.58	2.46149	YES	YES
337	a	1358.97	1.19960	YES	YES
338	a	1359.83	3.00971	YES	YES
339	a	1360.18	1.70972	YES	YES
340	a	1360.73	2.37182	YES	YES
341	a	1361.53	2.54277	YES	YES

342	a	1363.56	4.48513	YES	YES
343	a	1364.67	1.32427	YES	YES
344	a	1364.89	7.96502	YES	YES
345	a	1365.66	2.22897	YES	YES
346	a	1391.52	4.91183	YES	YES
347	a	1391.87	6.13198	YES	YES
348	a	1393.81	2.76142	YES	YES
349	a	1394.28	3.43805	YES	YES
350	a	1409.68	2.85066	YES	YES
351	a	1410.72	7.94987	YES	YES
352	a	1412.31	2.00750	YES	YES
353	a	1412.55	2.49581	YES	YES
354	a	1413.94	3.02619	YES	YES
355	a	1414.34	9.50137	YES	YES
356	a	1415.56	4.37215	YES	YES
357	a	1416.02	2.43985	YES	YES
358	a	1416.26	2.42576	YES	YES
359	a	1417.50	2.47077	YES	YES
360	a	1418.61	4.45987	YES	YES
361	a	1418.94	2.36368	YES	YES
362	a	1419.49	0.31610	YES	YES
363	a	1420.17	0.23714	YES	YES
364	a	1420.98	3.69371	YES	YES
365	a	1421.44	5.34696	YES	YES
366	a	1421.70	6.71064	YES	YES
367	a	1421.83	0.81234	YES	YES
368	a	1423.01	0.48719	YES	YES
369	a	1424.05	1.23535	YES	YES
370	a	1424.48	2.16620	YES	YES
371	a	1424.85	0.21044	YES	YES
372	a	1424.92	1.99728	YES	YES
373	a	1426.12	3.39483	YES	YES
374	a	1427.09	3.41941	YES	YES
375	a	1427.69	0.71361	YES	YES
376	a	1430.79	3.52019	YES	YES
377	a	1431.24	6.27743	YES	YES
378	a	1431.60	3.25185	YES	YES
379	a	1431.74	0.62084	YES	YES
380	a	1432.39	3.36994	YES	YES
381	a	1432.96	0.66522	YES	YES
382	a	1434.21	6.18188	YES	YES
383	a	1434.71	8.67757	YES	YES
384	a	1436.10	7.67106	YES	YES
385	a	1436.13	13.86204	YES	YES
386	a	1436.49	13.71613	YES	YES
387	a	1437.66	0.91584	YES	YES
388	a	1438.24	6.45512	YES	YES
389	a	1438.53	10.04101	YES	YES
390	a	1438.78	3.58777	YES	YES

391	a	1439.12	2.35542	YES	YES
392	a	1441.58	0.47489	YES	YES
393	a	1442.69	10.93423	YES	YES
394	a	1442.97	15.63120	YES	YES
395	a	1443.25	1.64316	YES	YES
396	a	1443.56	12.70309	YES	YES
397	a	1448.69	12.31416	YES	YES
398	a	1449.62	5.60251	YES	YES
399	a	1450.11	15.27366	YES	YES
400	a	2866.84	79.14134	YES	YES
401	a	2868.77	71.04884	YES	YES
402	a	2890.55	44.73709	YES	YES
403	a	2893.24	49.43005	YES	YES
404	a	2894.97	30.33882	YES	YES
405	a	2901.82	24.03317	YES	YES
406	a	2919.02	29.81853	YES	YES
407	a	2919.38	44.06529	YES	YES
408	a	2920.19	38.04292	YES	YES
409	a	2920.49	15.96961	YES	YES
410	a	2921.98	77.30753	YES	YES
411	a	2922.83	12.24326	YES	YES
412	a	2922.95	51.25691	YES	YES
413	a	2923.28	51.13576	YES	YES
414	a	2923.34	28.13131	YES	YES
415	a	2923.73	42.87569	YES	YES
416	a	2924.10	30.28899	YES	YES
417	a	2924.45	47.48915	YES	YES
418	a	2924.73	11.80859	YES	YES
419	a	2924.83	23.15531	YES	YES
420	a	2924.99	7.77203	YES	YES
421	a	2925.46	30.40235	YES	YES
422	a	2925.70	67.04259	YES	YES
423	a	2925.78	6.46248	YES	YES
424	a	2926.34	17.48240	YES	YES
425	a	2927.21	18.75275	YES	YES
426	a	2927.27	58.55501	YES	YES
427	a	2927.42	25.83987	YES	YES
428	a	2927.71	5.25381	YES	YES
429	a	2927.94	19.43419	YES	YES
430	a	2928.68	167.96894	YES	YES
431	a	2928.70	13.01522	YES	YES
432	a	2929.91	172.73734	YES	YES
433	a	2930.23	71.32576	YES	YES
434	a	2930.94	83.66401	YES	YES
435	a	2931.43	86.35993	YES	YES
436	a	2932.28	69.51356	YES	YES
437	a	2936.35	22.20836	YES	YES
438	a	2938.65	465.37655	YES	YES
439	a	2940.00	256.90718	YES	YES

440	a	2940.40	39.16744	YES	YES
441	a	2942.03	39.76187	YES	YES
442	a	2942.23	70.83950	YES	YES
443	a	2943.42	25.60711	YES	YES
444	a	2950.01	49.10666	YES	YES
445	a	2952.71	35.10819	YES	YES
446	a	2982.72	9.24303	YES	YES
447	a	2991.89	24.51266	YES	YES
448	a	2996.64	10.46220	YES	YES
449	a	2996.89	2.02561	YES	YES
450	a	2998.66	2.67804	YES	YES
451	a	2999.34	0.51903	YES	YES
452	a	2999.97	48.17396	YES	YES
453	a	3000.58	6.94441	YES	YES
454	a	3000.89	5.78274	YES	YES
455	a	3001.24	17.42719	YES	YES
456	a	3001.34	5.58627	YES	YES
457	a	3001.46	6.69096	YES	YES
458	a	3001.68	37.50692	YES	YES
459	a	3002.00	23.72739	YES	YES
460	a	3002.08	6.15350	YES	YES
461	a	3002.47	34.59189	YES	YES
462	a	3002.70	67.03126	YES	YES
463	a	3003.83	62.91804	YES	YES
464	a	3004.06	43.68343	YES	YES
465	a	3006.84	16.24746	YES	YES
466	a	3007.19	12.11637	YES	YES
467	a	3007.24	44.77886	YES	YES
468	a	3008.70	36.24207	YES	YES
469	a	3008.98	3.39350	YES	YES
470	a	3009.63	24.32917	YES	YES
471	a	3009.98	45.46159	YES	YES
472	a	3010.81	14.59888	YES	YES
473	a	3011.44	2.46352	YES	YES
474	a	3011.85	23.02795	YES	YES
475	a	3012.34	30.62959	YES	YES
476	a	3014.14	19.96906	YES	YES
477	a	3014.50	56.94885	YES	YES
478	a	3018.72	14.14095	YES	YES
479	a	3023.07	7.43332	YES	YES
480	a	3023.10	17.53557	YES	YES
481	a	3023.38	15.13502	YES	YES
482	a	3023.80	6.38267	YES	YES
483	a	3023.98	13.78657	YES	YES
484	a	3027.11	18.53768	YES	YES
485	a	3031.17	18.00239	YES	YES
486	a	3033.11	6.14645	YES	YES
487	a	3037.06	15.41308	YES	YES
488	a	3037.98	13.59324	YES	YES

489	a	3038.69	20.30135	YES	YES
490	a	3038.92	8.64879	YES	YES
491	a	3040.41	15.73461	YES	YES
492	a	3040.90	9.05945	YES	YES
493	a	3042.72	8.62119	YES	YES
494	a	3043.17	7.60739	YES	YES
495	a	3043.47	8.03879	YES	YES
496	a	3045.79	2.44728	YES	YES
497	a	3049.37	15.40567	YES	YES
498	a	3049.74	8.39082	YES	YES
499	a	3052.25	6.38359	YES	YES
500	a	3055.74	6.57894	YES	YES
501	a	3058.13	8.64318	YES	YES
